# A nomenclator of extant and fossil taxa of the Melanopsidae (Gastropoda, Cerithioidea)

**DOI:** 10.3897/zookeys.602.8136

**Published:** 2016-07-05

**Authors:** Thomas A. Neubauer

**Affiliations:** 1 Geological-Paleontological Department, Natural History Museum Vienna, 1010 Vienna, Austria

**Keywords:** Non-marine snails, catalogue, nomenclature, taxonomy, www-references

## Abstract

This nomenclator provides details on all published names in the family-, genus-, and species-group, as well as for a few infrasubspecific names introduced for, or attributed to, the family Melanopsidae. It includes nomenclaturally valid names, as well as junior homonyms, junior objective synonyms, nomina nuda, common incorrect subsequent spellings, and as far as possible discussion on the current status in taxonomy. The catalogue encompasses three family-group names, 79 genus-group names, and 1381 species-group names. All of them are given in their original combination and spelling (except mandatory corrections requested by the Code), along with their original source. For each family- and genus-group name, the original classification and the type genus and type species, respectively, are given. Data provided for species-group taxa are type locality, type horizon (for fossil taxa), and type specimens, as far as available.

## Introduction

The family Melanopsidae (Caenogastropoda: Cerithioidea) is one of the most diverse groups of non-marine gastropods in Earth history (Strong et al. 2008). Today, the family occurs in southern to eastern Europe, northern Africa, and the Middle East, as well as New Zealand and some south Pacific islands (Bănărescu 1990, Glaubrecht 1993, 1996, Altaba 1998, Strong et al. 2008). Its record dates back at least into the late Cretaceous of Europe (Bandel 2000, Neubauer et al. 2016a). While the fossil record of New Zealand species is fairly restricted, the record for Europe, northern Africa and the Middle East yielded many hundreds of species-group taxa. Early species were pure brackish-water dwellers, which they remained until the early Miocene, when they started to conquer freshwater (Glaubrecht 1996, Neubauer et al. 2016a). This change in life style coincided with a series of adaptive radiations in the middle and late Miocene, producing hundreds of species, and forming the basis for the modern clades and present diversity and distribution (Neubauer et al. 2016a).

The first detailed listing of names of living “Melanidae” – at that time the Melanopsidae were considered a subfamily of that group – was published by Brot (1874–1879). Soon later, Bourguignat (1884) followed with his “Histoire des mélaniens du système européen”, in which he described numerous new species, but few of them are currently still accepted. Pallary (1926a, b) provided a list of all published names of fossil and recent Melanopsidae, yet without any comments. Wenz (1929) summarized all Cenozoic (Paleocene–Pleistocene) names and gave extensive synonymy lists. Besides, there are papers on the fossil and recent Melanopsidae of Italy (Pantanelli 1886b) and Spain (Pallary 1924, Azpeitia Moros 1929). In addition, Pallary (1916a, b, 1920, 1925) provided a detailed account of nomenclatural mistakes concerning this family and introduced a great many of replacement names for existing homonyms.

A comprehensive annotated list of melanopsid names is, however, entirely missing. This catalogue presents information for all published names in the family-, genus- and species-group, as well as for a few infrasubspecific names. Discussed are nomenclaturally valid, invalid (e.g., junior objective synonyms, junior homonyms) and unavailable names (e.g., nomina nuda, misspellings), following the rules of the fourth edition of the International Code of Zoological Nomenclature (ICZN 1999, henceforth referred to as “the Code”), incorporating later added amendments (see http://www.nhm.ac.uk/hosted-sites/iczn/code/index.jsp). To facilitate comparison, the general outline of the present work follows the excellent nomenclator of Valvatidae by Haszprunar (2014).

## Part I: Supraspecific taxa in Melanopsidae

The supraspecific phylogenetic relationships within the Melanopsidae are poorly resolved (Strong et al. 2011). Most of the subfamilies, genera and subgenera were established for particular morphological characteristics, most of which have been later shown to be highly variable and, moreover, polyphyletic (e.g., Neubauer et al. 2014a). Few of them are currently used in taxonomy.

The fossil Stomatopsinae Stache, 1889 (type genus: *Stomatopsis* Stache in Sandberger, 1871), originally introduced as subfamily of the “Melaniidae” (although Stache 1889 used the suffix “-idae”) but considered as a junior synonym of the Melanopsidae by Bouchet and Rocroi (2005: 248), are not included here. Their systematic position is highly uncertain and requires a more detailed re-examination.

### Part IA. Family-group names

#### AMPHIMELANIINAE

Taxon classificationAnimaliaSorbeoconchaMelanopsidae

P. Fischer & Crosse, 1891

##### Original source.

Fischer and Crosse 1880–1902: 312.

##### Original classification.

Subfamily of Melaniidae.

##### Type genus.

*Amphimelania* P. Fischer, 1885 (junior objective synonym of *Holandriana* Bourguignat, 1884).

##### Remarks.

Considered a junior synonym of the Melanopsidae by Bouchet and Rocroi (2005: 248).

#### FAGOTIINAE

Taxon classificationAnimaliaSorbeoconchaMelanopsidae

Starobogatov in Starobogatov et al., 1992

##### Original source.

Starobogatov et al. 1992: 58.

##### Original classification.

Subfamily of Melanopsidae.

##### Type genus.

*Fagotia* Bourguignat, 1884.

##### Remarks.

Considered a junior synonym of the Melanopsidae by Bouchet and Rocroi (2005: 248).

#### MELANOPSIDAE

Taxon classificationAnimaliaSorbeoconchaMelanopsidae

H. Adams & A. Adams, 1854

##### Original source.

Adams and Adams 1853–1858: 309.

##### Original classification.

Subfamily of Melaniidae.

##### Type genus.

*Melanopsis* Férussac in Férussac & Férussac, 1807.

### Part IB. Genus-group names

In the following list, not all genus-group names are accompanied by a type species. Especially Bourguignat, who introduced the greatest number of melanopsid (sub)genera, rarely designated type species. Before 1931, a type species fixation was not a requirement for being available (ICZN 1999, Art. 12, 13.3). Original classifications of genus-level taxa are omitted for genera that were introduced without clear family classification. Purely fossil genera are marked by a dagger.

#### Aciculariana

Taxon classificationAnimaliaSorbeoconchaMelanopsidae

Bourguignat, 1884

##### Original source.

Bourguignat 1884: 51.

##### Original classification.

Subgenus of *Microcolpia*.

##### Remarks.

Bourguignat (1884) apparently intended to base this subgenus on *Melanopsis acicularis*. If originally or subsequently designated as type species, *Aciculariana* would be an objective synonym of *Microcolpia*.

#### Acroxiana

Taxon classificationAnimaliaSorbeoconchaMelanopsidae

Bourguignat, 1884

##### Original source.

Bourguignat 1884: 32.

##### Original classification.

Subgenus of *Fagotia*.

#### Amphimelania

Taxon classificationAnimaliaSorbeoconchaMelanopsidae

P. Fischer, 1885 [invalid]

##### Original source.

Fischer and Crosse 1880–1902: 701.

##### Original classification.

Subgenus of *Melania*.

##### Type species.

*Melania holandrii* Pfeiffer, 1828, by original designation.

##### Remarks.

Replacement name for *Melanella* Swainson, 1840, non Bowdich, 1822. Junior objective synonym of *Holandriana* Bourguignat, 1884, with the same type species.

#### Asmena

Taxon classificationAnimaliaSorbeoconchaMelanopsidae

Gistel, 1848 [invalid]

##### Original source.

Gistel 1848: xi.

##### Remarks.

Unnecessary substitute name for *Melanopsis* Férussac in Férussac & Férussac, 1807.

#### † Battistiana

Taxon classificationAnimaliaSorbeoconchaMelanopsidae

Pallary, 1920 [invalid]

##### Original source.

Pallary 1920b: 108.

##### Original classification.

Genus of Melanopsidae.

##### Type species.

† *Buccinum fossile* Gmelin, 1791, by typification of replaced name.

##### Remarks.

Established as a replacement name for *Pannonia* Pallary, 1916, wrongly assumed by Pallary (1920b) to be preoccupied by *Pannona* Lörenthey, 1902, and in fact a junior homonym of *Pannonia* Dollfus, 1912.

#### Belusiana

Taxon classificationAnimaliaSorbeoconchaMelanopsidae

Bourguignat, 1884

##### Original source.

Bourguignat 1884: 75.

##### Original classification.

Subgenus of *Melanopsis*.

#### † Boistelia

Taxon classificationAnimaliaSorbeoconchaMelanopsidae

Cossmann, 1909

##### Original source.

Cossmann 1909: 183.

##### Original classification.

Section of *Melanopsis*.

##### Type species.

† *Melanoptychia paradoxa* Brusina, 1892, by original designation.

#### Buccinoidiana

Taxon classificationAnimaliaSorbeoconchaMelanopsidae

Bourguignat, 1884

##### Original source.

Bourguignat 1884: 73.

##### Original classification.

Subgenus of *Melanopsis*.

#### Caledomelanopsis

Taxon classificationAnimaliaSorbeoconchaMelanopsidae

Germain, 1934 [unavailable]

##### Original source.

Germain 1934: 146.

##### Original classification.

Genus of Melanopsidae.

##### Remarks.

Germain (1934) neither provided a diagnosis, nor did he indicate a type species.

#### † Calodiona

Taxon classificationAnimaliaSorbeoconchaMelanopsidae

Stefanescu, 1896

##### Original source.

Stefanescu 1896: 126, 131.

##### Original classification.

Subgenus of *Melanopsis*.

##### Type species.

† *Melanopsis bergeroni* Stefanescu, 1896, by monotypy.

#### † Campylostylus

Taxon classificationAnimaliaSorbeoconchaMelanopsidae

Sandberger, 1870

##### Original source.

Sandberger 1870–1875: 90.

##### Original classification.

Subgenus of *Melanopsis*.

##### Type species.

† *Melanopsis galloprovincialis* Mathéron, 1843, by monotypy.

#### † Canthidomus

Taxon classificationAnimaliaSorbeoconchaMelanopsidae

Swainson, 1840

##### Original source.

Swainson 1840: 202, 342.

##### Original classification.

Subgenus of *Melanopsis*.

##### Type species.

† *Melanopsis bouei* Férussac, 1823, by subsequent designation by Herrmannsen (1846: 167).

#### Cariosiana

Taxon classificationAnimaliaSorbeoconchaMelanopsidae

Bourguignat, 1877

##### Original source.

Bourguignat 1877: 88.

##### Original classification.

Subgenus of *Melanopsis*.

##### Type species.

*Murex cariosus* Linnaeus, 1767, by original designation.

#### Ceneona

Taxon classificationAnimaliaSorbeoconchaMelanopsidae

Gistel, 1848 [invalid]

##### Original source.

Gistel 1848: 169.

##### Remarks.

Unnecessary substitute name for *Melanopsis* Férussac in Férussac & Férussac, 1807.

#### Charpentieriana

Taxon classificationAnimaliaSorbeoconchaMelanopsidae

Bourguignat, 1884

##### Original source.

Bourguignat 1884: 75.

##### Original classification.

Subgenus of *Melanopsis*.

#### Costatiana

Taxon classificationAnimaliaSorbeoconchaMelanopsidae

Bourguignat, 1884 

##### Original source.

Bourguignat 1884: 76.

##### Original classification.

Subgenus of *Melanopsis*.

#### Couphaniana

Taxon classificationAnimaliaSorbeoconchaMelanopsidae

Bourguignat, 1884

##### Original source.

Bourguignat 1884: 74.

##### Original classification.

Subgenus of *Melanopsis*.

##### Type species.

*Melanopsis coupha* Bourguignat, 1884, by monotypy.

#### Crassiana

Taxon classificationAnimaliaSorbeoconchaMelanopsidae

Bourguignat, 1884 [invalid]

##### Original source.

Bourguignat 1884: 8.

##### Original classification.

Subgenus of *Melanella*.

##### Remarks.

Junior homonym of *Crassiana* Servain, 1882 (Bivalvia, Unionidae).

#### † Duabiana

Taxon classificationAnimaliaSorbeoconchaMelanopsidae

Starobogatov & Anistratenko in Anistratenko, 1993

##### Original source.

Anistratenko 1993: 69, textfig. 1.

##### Original classification.

Subgenus of *Melanopsis*.

##### Type species.

† *Melanopsis cylindrica* Anistratenko, 1993, by original designation.

#### Dufouriana

Taxon classificationAnimaliaSorbeoconchaMelanopsidae

Bourguignat, 1884

##### Original source.

Bourguignat 1884: 75.

##### Original classification.

Subgenus of *Melanopsis*.

#### Esperiana

Taxon classificationAnimaliaSorbeoconchaMelanopsidae

Bourguignat, 1877

##### Original source.

Bourguignat 1877: 88.

##### Original classification.

Subgenus of *Melanopsis*.

##### Type species.

*Melanopsis esperi* Férussac, 1823, by original designation.

#### Eumorphiana

Taxon classificationAnimaliaSorbeoconchaMelanopsidae

Bourguignat, 1884

##### Original source.

Bourguignat 1884: 76.

##### Original classification.

Subgenus of *Melanopsis*.

#### Fagotia

Taxon classificationAnimaliaSorbeoconchaMelanopsidae

Bourguignat, 1884 [invalid]

##### Original source.

Bourguignat 1884: 30.

##### Original classification.

Subgenus of *Fagotia*.

##### Type species.

*Melanopsis esperi* Férussac, 1823, by subsequent designation by Wenz (1939: 690).

##### Remarks.

Wenz (1939) declared that *Melanopsis esperi* was the type species by monotypy, but 22 nominal species were originally included in *Fagotia*. Junior objective synonym of *Esperiana*.

#### Fagotiana

Taxon classificationAnimaliaSorbeoconchaMelanopsidae

Bourguignat, 1884

##### Original source.

Bourguignat 1884: 7.

##### Original classification.

Subgenus of *Melanella*.

##### Type species.

*Melanella fagotiana* Bourguignat, 1884, by tautonymy.

#### Graellsiana

Taxon classificationAnimaliaSorbeoconchaMelanopsidae

Bourguignat, 1884

##### Original source.

Bourguignat 1884: 75.

##### Original classification.

Subgenus of *Melanopsis*.

#### † Handmannia

Taxon classificationAnimaliaSorbeoconchaMelanopsidae

Cossmann, 1889 [invalid]

##### Original source.

Cossmann 1889: 1101.

##### Type species.

† *Melanopsis pygmaea* Hörnes, 1856, by original designation.

##### Remarks.

Established as a substitute name for *Homalia* Handmann, 1887, which Cossmann (1899), despite being aware of the difference, treated as a junior homonym of “*Homala* Schumacher” (actually *Omala* Schumacher, 1817, unjustified emendation to “*Homala*” by Agassiz 1847).

#### Holandriana

Taxon classificationAnimaliaSorbeoconchaMelanopsidae

Bourguignat, 1884

##### Original source.

Bourguignat 1884: 7.

##### Original classification.

Subgenus of *Melanella*.

##### Type species.

*Melania holandrii* Pfeiffer, 1828, by subsequent designation by Welter-Schultes (2012: 36).

#### † Homalia

Taxon classificationAnimaliaSorbeoconchaMelanopsidae

Handmann, 1887

##### Original source.

Handmann 1887: 12.

##### Original classification.

Subgenus of *Melanopsis*.

#### † Hyphantria

Taxon classificationAnimaliaSorbeoconchaMelanopsidae

Handmann, 1887 [invalid]

##### Original source.

Handmann 1887: 37.

##### Original classification.

Subgenus of *Melanopsis*.

##### Remarks.

Junior homonym of *Hyphantria* Harris, 1841 (Lepidoptera).

#### Laevigatiana

Taxon classificationAnimaliaSorbeoconchaMelanopsidae

Bourguignat, 1884

##### Original source.

Bourguignat 1884: 8.

##### Original classification.

Subgenus of *Melanella*.

#### Letourneuxiana

Taxon classificationAnimaliaSorbeoconchaMelanopsidae

Bourguignat, 1884 [invalid]

##### Original source.

Bourguignat 1884: 32.

##### Original classification.

Subgenus of *Fagotia*.

##### Remarks.

Bourguignat (1884) introduced three different divisions called *Letourneuxiana*, one for *Fagotia*, one for *Melanella* and one for *Melanopsis*. All are junior homonyms of *Letourneuxiana* Silva e Castro, 1883 (Unionidae).

#### Letourneuxiana

Taxon classificationAnimaliaSorbeoconchaMelanopsidae

Bourguignat, 1884 [invalid]

##### Original source.

Bourguignat 1884: 8.

##### Original classification.

Subgenus of *Melanella*.

##### Remarks.

Junior homonym of *Letourneuxiana* Silva e Castro, 1883 (Unionidae).

#### Letourneuxiana

Taxon classificationAnimaliaSorbeoconchaMelanopsidae

Bourguignat, 1884 [invalid]

##### Original source.

Bourguignat 1884: 75.

##### Original classification.

Subgenus of *Melanopsis*.

##### Type species.

*Melanopsis letourneuxi* Bourguignat, 1884, by monotypy.

##### Remarks.

Junior homonym of *Letourneuxiana* Silva e Castro, 1883 (Unionidae).

#### Locardiana

Taxon classificationAnimaliaSorbeoconchaMelanopsidae

Bourguignat, 1884

##### Original source.

Bourguignat 1884: 32.

##### Original classification.

Subgenus of *Fagotia*.

##### Type species.

*Fagotia locardiana* Bourguignat, 1884, by tautonymy.

#### Lorcaniana

Taxon classificationAnimaliaSorbeoconchaMelanopsidae

Bourguignat, 1884

##### Original source.

Bourguignat 1884: 74.

##### Original classification.

Subgenus of *Melanopsis*.

#### Lortetiana

Taxon classificationAnimaliaSorbeoconchaMelanopsidae

Bourguignat, 1884

##### Original source.

Bourguignat 1884: 76.

##### Original classification.

Subgenus of *Melanopsis*.

##### Type species.

*Melanopsis lortetiana* Locard, 1883, by tautonymy.

#### Lyrcea

Taxon classificationAnimaliaSorbeoconchaMelanopsidae

H. Adams & A. Adams, 1854

##### Original source.

Adams and Adams 1853–1858: 310.

##### Original classification.

Subgenus of *Melanopsis*.

##### Type species.

*Melanopsis dufourii* Férussac, 1822, by subsequent designation by Cossmann (1909: 174).

##### Remarks.

“*Lyrcaea*” as mentioned in Wenz (1929: 2647) and many other authors is an incorrect subsequent spelling.

#### † Macrospira

Taxon classificationAnimaliaSorbeoconchaMelanopsidae

Sandberger, 1872 [invalid]

##### Original source.

Sandberger 1870–1875: 222, 248.

##### Original classification.

Subgenus of *Melanopsis*.

##### Remarks.

Junior homonym of *Macrospira* Guilding in Swainson, 1840. Rovereto (1899: 109) introduced *Stilospirula* as replacement name.

#### Maresiana

Taxon classificationAnimaliaSorbeoconchaMelanopsidae

Bourguignat, 1884

##### Original source.

Bourguignat 1884: 77.

##### Original classification.

Subgenus of *Melanopsis*.

#### † Martinia

Taxon classificationAnimaliaSorbeoconchaMelanopsidae

Handmann, 1887 [invalid]

##### Original source.

Handmann 1887: 19.

##### Original classification.

Subgenus of *Melanopsis*.

##### Type species.

† *Melanopsis martiniana* Férussac, 1823 [objective synonym of *Melanopsis fossilis* (Gmelin, 1791)], by subsequent designation by Cossmann (1909: 174).

##### Remarks.

Junior homonym of *Martinia* M’Coy in M’Coy & Griffith, 1844 (Brachiopoda).

#### † Megalonoda

Taxon classificationAnimaliaSorbeoconchaMelanopsidae

Kollmann, 1984

##### Original source.

Kollmann 1984: 56.

##### Original classification.

Genus of Melanopsidae.

##### Type species.

† *Purpuroidea reussi* Hörnes, 1856, by original designation.

#### Melafusus

Taxon classificationAnimaliaSorbeoconchaMelanopsidae

Swainson, 1840

##### Original source.

Swainson 1840: 201, 341.

##### Original classification.

Subgenus of *Melanopsis*.

##### Remarks.

Briefly described but no species included.

#### Melanella

Taxon classificationAnimaliaSorbeoconchaMelanopsidae

Swainson, 1840 [invalid]

##### Original source.

Swainson 1840: 199, 341.

##### Original classification.

Subgenus of *Melania*.

##### Remarks.

No originally included species. Junior homonym of *Melanella* Bowdich, 1822. Fischer (1885: 701) introduced *Amphimelania* as replacement name, which is invalid, too, because it is a junior objective synonym of *Holandriana*, with the same type species.

#### Melanithes

Taxon classificationAnimaliaSorbeoconchaMelanopsidae

Swainson, 1840

##### Original source.

Swainson 1840: 202, 341.

##### Original classification.

Subgenus of *Melanopsis*.

##### Remarks.

Based on some of Férussac’s (1823) fossil (Eocene to Pliocene) *Melanopsis* species but no type species was designated.

#### Melanopsis

Taxon classificationAnimaliaSorbeoconchaMelanopsidae

J.B.L.d’A. de Férussac in J.B.L. d’A. de Férussac & A.E.J.P.J.F. d’A. de Férussac, 1807

##### Original source.

Férussac and Férussac 1807: 70.

##### Type species.

*Melania costata* Olivier, 1804, by subsequent designation by Gray (1847: 153).

##### Remarks.

Correct authority is denoted on p. xii of Férussac and Férussac (1807).

#### † Melanoptychia

Taxon classificationAnimaliaSorbeoconchaMelanopsidae

Neumayr, 1880

##### Original source.

Neumayr 1880c: 480.

##### Original classification.

Genus of Melanopsidae.

##### Type species.

† *Melanoptychia bittneri* Neumayr, 1880, by subsequent designation by Cossmann (1909: 182).

#### † Melanosteira

Taxon classificationAnimaliaSorbeoconchaMelanopsidae

Andreae, 1893

##### Original source.

Andreae 1893: 172.

##### Original classification.

Genus of Melanopsidae.

##### Type species.

† *Melanopsis aetolica* Neumayr, 1876, by subsequent designation by Cossmann (1909: 179).

##### Remarks.

The name is not available from Oppenheim (1891), who used the vernacular “Melanosteiren”. In a book review of that paper, Andreae (1893) Latinized the name and made it thereby available.

#### † Melanostira

Taxon classificationAnimaliaSorbeoconchaMelanopsidae

Cossmann, 1909 [invalid]

##### Original source.

Cossmann 1909: 179.

##### Remarks.

Unjustified emendation and therefore junior objective synonym of *Melanosteira* Andreae, 1893.

#### Mesopotamia

Taxon classificationAnimaliaSorbeoconchaMelanopsidae

Pallary, 1939 [unavailable]

##### Original source.

Pallary 1939: 98.

##### Original classification.

Subgenus of *Melanopsis*.

##### Remarks.

Unavailable because no type species was designated (Art. 13.3).

#### Microcolpia

Taxon classificationAnimaliaSorbeoconchaMelanopsidae

Bourguignat, 1884

##### Original source.

Bourguignat 1884: 49.

##### Original classification.

Genus of Melaniidae (note that Bourguignat actually only gave the vernacular “Mélaniens”).

##### Type species.

*Melanopsis acicularis* Férussac, 1823, by subsequent designation by Cossmann (1909: 159).

##### Remarks.

“*Microcalpia*” as mentioned in Fischer (1886: 705) is an incorrect subsequent spelling.

#### Mingreliciana

Taxon classificationAnimaliaSorbeoconchaMelanopsidae

Bourguignat, 1884

##### Original source.

Bourguignat 1884: 74.

##### Original classification.

Subgenus of *Melanopsis*.

##### Type species.

*Melanopsis mingrelica* Mousson, 1863, by subsequent designation by Izzatullaev and Starobogatov (1984: 1476).

#### Myosotidiana

Taxon classificationAnimaliaSorbeoconchaMelanopsidae

Bourguignat, 1884

##### Original source.

Bourguignat 1884: 74.

##### Original classification.

Subgenus of *Melanopsis*.

##### Type species.

*Melanopsis myosotidaea* Bourguignat, 1884, by monotypy.

#### Nodosiana

Taxon classificationAnimaliaSorbeoconchaMelanopsidae

Bourguignat, 1884

##### Original source.

Bourguignat 1884: 76.

##### Original classification.

Subgenus of *Melanopsis*.

##### Type species.

*Melanopsis nodosa* Férussac, 1822, by monotypy.

#### Olivieriana

Taxon classificationAnimaliaSorbeoconchaMelanopsidae

Bourguignat, 1884

##### Original source.

Bourguignat 1884: 74.

##### Original classification.

Subgenus of *Melanopsis*.

#### Pakaurangia

Taxon classificationAnimaliaSorbeoconchaMelanopsidae

Finlay, 1926

##### Original source.

Finlay 1926: 380, 476.

##### Original classification.

Subgenus of *Zemelanopsis*.

##### Type species.

*Melanopsis waitaraensis* Marwick, 1926, by original designation.

##### Remarks.

Finlay (1926) included also another species, the fossil *Coptochetus zelandicus* Marshall, 1918 (p. 265, pl. 22, fig. 13), in this subgenus. That species is, however, not a Melanopsidae but belongs to the marine genus *Exilia* Conrad, 1860 after Beu and Maxwell (1990: 414) and is consequently not treated in this catalogue. Finally, Bouchet and Poppe (1995: 503) synonymized *Pakaurangia* with the marine deep-water genus *Calliotectum* (Volutidae).

#### † Pannonia

Taxon classificationAnimaliaSorbeoconchaMelanopsidae

Pallary, 1916 [invalid]

##### Original source.

Pallary 1916: 76.

##### Original classification.

Subgenus of *Melanopsis*.

##### Type species.

† *Buccinum fossile* Gmelin, 1791, by original designation.

##### Remarks.

Replacement name for *Martinia* Handmann, 1887, non M’Coy in M’Coy & Griffith, 1844. Junior homonym of *Pannonia* Dollfus, 1912 (see *Battistiana*).

#### Parreyssiana

Taxon classificationAnimaliaSorbeoconchaMelanopsidae

Bourguignat, 1884

##### Original source.

Bourguignat 1884: 76.

##### Original classification.

Subgenus of *Melanopsis*.

##### Type species.

*Melanopsis parreyssi* Philippi, 1847, by monotypy.

#### † Pauluccia

Taxon classificationAnimaliaSorbeoconchaMelanopsidae

Brusina, 1902

##### Original source.

Brusina 1902: viii.

##### Original classification.

Subgenus of *Melanopsis*.

#### Pechaudiana

Taxon classificationAnimaliaSorbeoconchaMelanopsidae

Bourguignat, 1884

##### Original source.

Bourguignat 1884: 77.

##### Original classification.

Subgenus of *Melanopsis*.

#### Potamactebiana

Taxon classificationAnimaliaSorbeoconchaMelanopsidae

Bourguignat, 1884

##### Original source.

Bourguignat 1884: 52.

##### Original classification.

Subgenus of *Microcolpia*.

##### Type species.

*Melanopsis potamactebia* Bourguignat, 1870, by subsequent designation by Anistratenko and Anistratenko (2001: 150).

#### Praeclariana

Taxon classificationAnimaliaSorbeoconchaMelanopsidae

Bourguignat, 1884

##### Original source.

Bourguignat 1884: 51.

##### Original classification.

Subgenus of *Microcolpia*.

##### Type species.

*Microcolpia praeclara* Bourguignat, 1884, by monotypy.

#### Praemorsiana

Taxon classificationAnimaliaSorbeoconchaMelanopsidae

Bourguignat, 1877

##### Original source.

Bourguignat 1877: 88.

##### Original classification.

Subgenus of *Melanopsis*.

##### Type species.

*Buccinum praemorsum* Linnaeus, 1758, by original designation.

#### Pseudhemisinus

Taxon classificationAnimaliaSorbeoconchaMelanopsidae

Nevill, 1884 [invalid]

##### Original source.

Nevill 1884: 214.

##### Original classification.

Subgenus of *Melanopsis*.

##### Type species.

*Melanopsis esperi* Férussac, 1823, by original designation.

##### Remarks.

Junior objective synonym of *Esperiana* Bourguignat, 1876, with the same type species.

#### † Pseudofagotia

Taxon classificationAnimaliaSorbeoconchaMelanopsidae

Anistratenko, 1993

##### Original source.

Anistratenko 1993: 72.

##### Original classification.

Genus of Fagotiinae.

##### Type species.

† *Pseudofagotia lineata* Anistratenko, 1993, by original designation.

#### Pyramidaliana

Taxon classificationAnimaliaSorbeoconchaMelanopsidae

Bourguignat, 1884

##### Original source.

Bourguignat 1884: 51.

##### Original classification.

Subgenus of *Microcolpia*.

#### † Sasykiana

Taxon classificationAnimaliaSorbeoconchaMelanopsidae

Gozhik in Gozhik & Datsenko, 2007

##### Original source.

Gozhik and Datsenko 2007: 98.

##### Original classification.

Subgenus of *Fagotia*.

##### Type species.

† Fagotia (Sasykiana) plena Gozhik in Gozhik & Datsenko, 2007, by original designation.

#### Saulcyana

Taxon classificationAnimaliaSorbeoconchaMelanopsidae

Bourguignat, 1884

##### Original source.

Bourguignat 1884: 75.

##### Original classification.

Subgenus of *Melanopsis*.

#### Scalariana

Taxon classificationAnimaliaSorbeoconchaMelanopsidae

Bourguignat, 1884

##### Original source.

Bourguignat 1884: 74.

##### Original classification.

Subgenus of *Melanopsis*.

#### Seignettiana

Taxon classificationAnimaliaSorbeoconchaMelanopsidae

Bourguignat, 1884

##### Original source.

Bourguignat 1884: 74.

##### Original classification.

Subgenus of *Melanopsis*.

#### Servainiana

Taxon classificationAnimaliaSorbeoconchaMelanopsidae

Bourguignat, 1884

##### Original source.

Bourguignat 1884: 51.

##### Original classification.

Subgenus of *Microcolpia*.

#### Sesteriana

Taxon classificationAnimaliaSorbeoconchaMelanopsidae

Bourguignat, 1884

##### Original source.

Bourguignat 1884: 75.

##### Original classification.

Subgenus of *Melanopsis*.

#### Sistaniana

Taxon classificationAnimaliaSorbeoconchaMelanopsidae

Izzatullaev & Starobogatov, 1984

##### Original source.

Izzatullaev and Starobogatov 1984: 1476.

##### Original classification.

Subgenus of *Melanopsis*.

##### Type species.

*Melanopsis sistanica* Izzatullaev & Starobogatov, 1984, by original designation.

#### Smendovia

Taxon classificationAnimaliaSorbeoconchaMelanopsidae

Tournouër, 1882

##### Original source.

Tournouër 1882: 59.

##### Original classification.

Genus of Melanopsinae.

##### Type species.

† *Melanopsis thomasi* Tournouër, 1877, by original designation.

#### Speciosiana

Taxon classificationAnimaliaSorbeoconchaMelanopsidae

Bourguignat, 1884

##### Original source.

Bourguignat 1884: 8.

##### Original classification.

Subgenus of *Melanella*.

##### Type species.

*Melanella speciosa* Bourguignat, 1884, by monotypy.

#### † Spiridionia

Taxon classificationAnimaliaSorbeoconchaMelanopsidae

Cossmann, 1909

##### Original source.

Cossmann 1909: 178.

##### Original classification.

Subgenus of *Melanopsis*.

##### Type species.

† *Melanopsis austriaca* Handmann, 1882, by original designation.

##### Remarks.

Replacement name for *Hyphantria* Handmann, 1887, non Harris, 1841 (Lepidoptera).

#### † Stilospirula

Taxon classificationAnimaliaSorbeoconchaMelanopsidae

Rovereto, 1899

##### Original source.

Rovereto 1899: 109.

##### Original classification.

Genus of “Melaniidae”.

##### Type species.

† *Melanopsis proboscidea* Deshayes, 1862, by subsequent designation by Cossmann (1909: 172; note that Cossmann made an unjustified emendation by changing the name to “*Stylospirula*”).

##### Remarks.

Replacement name for *Macrospira* Sandberger, 1872, non Guilding in Swainson, 1840.

#### † Stylospirula

Taxon classificationAnimaliaSorbeoconchaMelanopsidae

Cossmann, 1899 [invalid]

##### Original source.

Cossmann 1899: 147.

##### Remarks.

Unjustified emendation and therefore junior objective synonym of *Stilospirula* Rovereto, 1899.

#### † Turripontica

Taxon classificationAnimaliaSorbeoconchaMelanopsidae

Anistratenko, 1993

##### Original source.

Anistratenko 1993: 75.

##### Original classification.

Genus of Fagotiinae.

##### Type species.

† *Turripontica aciculina* Anistratenko, 1993, by original designation.

#### Villeserriana

Taxon classificationAnimaliaSorbeoconchaMelanopsidae

Bourguignat, 1884

##### Original source.

Bourguignat 1884: 51.

##### Original classification.

Subgenus of *Microcolpia*.

##### Type species.

*Microcolpia villeserriana* Bourguignat, 1884, by tautonymy.

#### Zemelanopsis

Taxon classificationAnimaliaSorbeoconchaMelanopsidae

Finlay, 1926

##### Original source.

Finlay 1926: 380, 476.

##### Type species.

*Melanopsis trifasciata* Gray, 1843, by original designation.

## Part II: Species-group and infrasubspecific names

The number of living melanopsid species-group taxa accepted in taxonomy today ranges between 25 and 50 (Strong et al. 2008); the IUCN Red List includes 36 species. Fossil species are far more numerous, with over 450 accepted names (Neubauer et al. 2016a). Beyond that, a plethora of homonyms and synonyms have been described in the Melanopsidae. The catalogue includes taxa initially described in this family and subsequently classified in other families and vice versa. It comprises a total of 1381 names, of which 71 are unavailable (mostly nomina nuda), 252 are available but invalid (junior homonyms and junior objective synonyms) and 15 are unresolved (requiring the action of a First Reviser).

Not treated by the present work are taxa not described for, and currently not considered to belong to, the Melanopsidae but classified therein previously. In the 19^th^ century, many species introduced in the group that nowadays is understood as the family Melanopsidae, especially regarding varieties of *Holandriana holandrii* (Pfeiffer, 1828), have been classified within the genus *Melania* Lamarck, 1799 (e.g., Rossmässler 1838–1844, Brusina 1866, Kobelt 1881, Westerlund 1886, 1898). In order to avoid mixing with “real” *Melania* (which is a junior objective synonym of *Thiara* Röding, 1798, the type species of the family Thiaridae), only such *Melania* species-group taxa attributed to the *Holandriana holandrii* species-complex, or those that have been claimed to be related to that group, are included herein. Further taxa that are not included are: species introduced within the genera *Faunus* Montfort, 1810, *Hemisinus* Swainson, 1840, *Dicolpus* Philippi, 1887 [invalid: junior homonym of *Dicolpus* Gerstaecker, 1884 (Insecta)], *Loxotrema* Gabb, 1868, and *Boggsia* Olsson, 1929 that have been occasionally classified in the Melanopsidae (e.g., Brot 1874–1879, Cossmann 1909, Squires 1998, 1999, Squires and Saul 1997) but are not considered to belong there today; the Bullia (Bulliopsis) species described by Conrad (1830, 1862) from middle Miocene deposits of Maryland, USA (*Bulliopsis integra* Conrad, 1862, *Bulliopsis marylandica* Conrad, 1862, and *Bulliopsis quadrata* Conrad, 1830) that he later considered melanopsids (Conrad 1866) but which belong to the marine Nassariidae after Allmon (1990); Paleocene species of the Stomatopsinae Stache, 1889 (genera *Stomatopsis* Stache in Sandberger, 1871, *Stomatopsella* Stache, 1889, and *Megastomopsis* Stache, 1889) which was considered a subfamily of Melanopsidae by Bouchet & Rocroi (2005) but probably does not belong there; species introduced in the genus *Coptostylus* Sandberger, 1872, which has occasionally been classified within the Melanopsidae, but is currently assigned to the Thiaridae (Pacaud and Le Renard 1995: 156).

Basic sources used for the present assembly were the catalogues of Brot (1874–1879), Bourguignat (1884), Pallary (1926a, b) and Wenz (1929), as well as internet resources such as the Biodiversity Heritage Library (http://biodiversitylibrary.org/), the Global Names Index (http://gni.globalnames.org/) and the Worldwide Mollusc Species Data Base (http://www.bagniliggia.it/WMSD/WMSDhome.htm). However, many names listed in those online repositories are obvious misspellings deriving from automatic digitization procedures that have not been critically reviewed (compare discussion in Haszprunar 2014). Such errors are not included here because they do not come from the published literature. Some of the names currently listed in those databases could neither be traced back to any publication, nor could they be linked to an evident misspelling. These names are on purpose excluded from the present paper to avoid introducing potential misspellings or nomina nuda into the literature. Nevertheless, I will be grateful to receive information on names presently not included here, as well as corrections, explanations and additions.

Some of the publications consulted for this work display problematic cases, regarding their actual publication dates or the nomenclatural or taxonomic concepts applied therein. These issues require careful examinations, which are provided below.

### *Melanopsis* species introduced by André Etienne Justin Pascal Joseph Francois d’Audebard de Férussac

Férussac described several new species of *Melanopsis* in his “Histoire naturelle générale et particulière des mollusques terrestres et fluviatiles”, as well as in the “Monographie des espèces vivantes et fossiles du genre mélanopside”. The problem is to determine the exact publication dates of those works and, therefore, when and in which work which species was described.

The “Histoire naturelle” is an extremely comprehensive work, containing two volumes of text and 210 plates, which were published between March 1819 and August 1851 in 35 “livraisons”. It has been subject of intensive debates regarding the exact publication dates – for details see Kennard (1942a–c). The work was commenced by Férussac, who issued the first 28 livraisons between 1819 and 1832. After his death in 1836, Gérard Paul Deshayes finished the work by adding another seven livraisons between 1839 and 1851, including new explanations for the plates. The work includes two plates of “Mélanopsides fossiles”. The first plate was published in livraison 15 on 13 April 1822, but its captions came along with livraison 17, which was published on 2 November 1822 (Kennard 1842b: 106). The second plate appeared in livraison 21 on 27 September 1823 including its captions (Kennard 1842a: 16).

To increase confusion, there is a disagreement between the original captions provided by Férussac and the ones supplied by Deshayes with livraison 29 in 1839 (Table 1; see also Kennard 1842b: 106). Deshayes made several corrections and synonymizations and even introduced two new names in the altered captions for plate 2, i.e., *Melanopsis subcarinata* and Melanopsis nodosa var. longa. While the original captions for plate 2 are available to me, I could not find the captions for plate 1. Both plates were also published par for par in Férussac’s (1823) monograph on the Melanopsidae. The captions for plate 2 are the same in both works (except minor differences regarding the details provided), so we can assume the same for plate 1. However, the exact wording of the original captions of 1822 needs to be seen in the future, in order to finally ascertain the availability of the associated names. A different problem appears for plate 2. While the year of publication of the species illustrated there is undoubtedly 1823, the original publication itself is uncertain – either the livraison 21 of the “Histoire naturelle” [27 September 1823] or the “Monographie” [exact date unknown]. This matter could unfortunately not be solved.

**Table 1.** Férussac’s original figure legends of the two plates of “Mélanopsides fossiles” (1822/1823) in the “Histoire naturelle”, compared with the altered legends supplied by Deshayes in 1839 when finalizing the work. Note that Férussac’s original figure legends for plate 1 are unavailable to me and thus based on the legends for plate 7 in Férussac (1823), which are supposed to be identical (see text for details).

**Plate**	**Férussac’s original legend (1822/1823)**	**Deshayes’ altered legend (1839)**
1	Fig. 1, 2. *Melanopsis Buccinoidea*, var γ) antiqua: *inflata*. Des environs d’Epernay. *Melanopsis fusiformis*, Sowerby, *Min. conch*., tab. 232, fig. 1, 5, 7	Fig. 1–7. **Melanopsis fusiformis**, Sowerby. Diverses variétés d’Épernay, de Cuiseaux et d’Angleterre
	Fig. 3. La même plus jeune, du lieu dit les Rozières, près d’Épemay	
	Fig. 4. *MelanopsisBuccinoidea*? var. ε) *Minuta*; *fossilis*. De Cuiseaux, près St.-Amour, dans le bassin de la Saône. *Nota*. Les tours de spire sont un peu trop détachés dans la figure	
	Fig. 5. *MelanopsisBuccinoidea*, var. γ) antiqua: *elongata*. Des environs d’Epernay. *Melanopsis fusiformis*, Sowerby, loc. cit., fig. 2, 3, 6	
	Fig. 6. La même, de l’île de Wight. Sowerby, *id.*	
	Fig. 7. La même plus âgée, des environs d’Epernay	
	Fig. 8. *MelanopsisBuccinoidea*, var. α). *Fossilis*. de Sestos	8–11. — **buccinoides**, Férussac. Des îles de la Grèce et d’Italie
	Fig. 9. *MelanopsisBuccinoidea*, var. γ) antiqua: *inflata*. Du dépôt situé entre St.-Germinini et Carsoli; Italie	
	Fig. 10. *MelanopsisBuccinoidea*, var. α). *fossilis*. De l’île de Rhodes	
	Fig. 11. *MelanopsisBuccinoidea*, var. δ). *fossilis*. Des dépôts situés entre St.-Germini et Carsoli, et entre Otricoli et le Vigne	
	Fig. 12. *Melanopsis incerta*? var. de Sestos	12. — **incerta**, Fér. De Sestos
	Fig. 13. *Melanopsis nodosa*. Du dépôt situé entre Otricoli et le Vigne, route de Rome à Foligno	13. — **nodosa**, Férussac. Otricoli
	Fig. 14. *Melanopsis costata*, de Sestos	14–15. — **costata**, Fér. De Sestos
	Fig. 15. La même plus petite, de Sestos	
	Fig. 16. *MelanopsisDufourii*, var. ε). *Fossilis*, *maxima*. Des environs de Dax	16. — **Dufourii**, Fér. Dax
2	Fig. 1. *Melanopsis buccinoidea*, var. *a*) *Bulimus antidiluviamus*, Poiret.	Fig. 1, 2, 4, 5. **Melanopsis buccinoidea**, Férussac. Variétés de Carsoli, Italie
	Fig. 2. Id. var. γ) *Antiquua*. Du dépôt situé entre Saint-Germini et Carsoli	
	Fig. 3. Id. même var. Plus âgée. Du même lieu	3. — **subcarinata**, Desh. d’Italie
	Fig. 4. Id	
	Fig. 5. *MelanopsisDufourii*, var. *a.* De Dax	
	Fig. 6. *Melanopsis incerta*, var. *a*). Jeune de Sestos	6. — **Audebardi**, Desh. De Sestos. (Confondue par Férus. avec l’*incerta*)
	Fig. 7. *Melanopsis atra*, fossile? De l’île de Luçon	7. — **atra**, Fèrus. De Luçon
	Fig. 8. *Melanopsis nodosa*, var. *a*) *cylindracea*. Dans une roche calcaire d’Athènes	8. — **nodosa**, Fèrus. Var. *longa* d’Athènes
	Fig. 9. *Melanopsis Bouei*. De la Moravie	9–10. — **Bouei**, Fèrussac. De la Moravie
	Fig. 10. Id. var.	
	Fig. 11, 13. *Melanopsis Martiniana*. Grand exemplaires	11–13. — **martiniana**, Féruss. De la Moravie
	Fig. 12. La même coquille; individus moins âgés	

### Validity of names mentioned in Férussac’s and Deshayes’ works

Apart from the uncertainties detailed above, the inconsistent formatting of Férussac’s (1823) monograph led later authors to adopt names as valid that were certainly not intended as such by Férussac himself. He introduced several new *Melanopsis* species and varieties, latter of which he denoted by Greek letters followed by Latin descriptions. Quite often, he used the same Latin words to characterize different varieties and apparently did not mean to introduce available names (e.g., Férussac 1823: 154). New names (or new combinations) were always denoted by him with “nobis”. Table 2 summarizes the available species-group names and unnamed varieties introduced and described by Férussac (1823).

**Table 2.** Available species-group names (in italics) and unnamed varieties in Férussac (1823). Only the names in bold were first introduced in that work. The assigned status as Recent (R) or fossil (F) is based on Férussac’s concept of the taxon in question, whether or not this reflects the latest opinion. Abbreviations: Lam. – Lamarck; O. – Olivier.

Species	Variety	Infrasub-specific name	Remarks	Fossil/recent
*buccinoidea* O.				R+F
	α			R+F
	β			R
	γ (“Antiquua”)		If considered available, it was a junior objective synonym of *Melanopsis fusiformis* Sowerby, 1822	F
		*inflata*	Already available from Férussac’s “Histoire naturelle”	F
		*elongata*	Already available from Férussac’s “Histoire naturelle”	F
	δ			F
	ε			F
*dufourii*			Already available from Férussac’s “Histoire naturelle”	R+F
	α			F
	β			R
	γ			R
	δ			R
	ε			F
	ζ			R
	η			R
	θ			R
*martiniana*			Junior objective synonym of *Melanopsis fossilis* (Gmelin, 1791)	F
***incerta***				F
*costata* O.				R+F
	α (“*Fasciata*”)		Nomen nudum	?
***costellata***			Junior objective synonym of *Melanopsis cariosa* (Linnaeus, 1767)	R
	α			R
*nodosa*			Already available from Férussac’s “Histoire naturelle”	R+F
	α			F
***bouei***				F
***decussata***				R
***esperi***				R
***acicularis***				R+F
	α			R
	β			R
*atra* Lam.				R
*spinosa* Lam.				R

The variety *Melania buccinoidea* var. “γ) Antiquua” [sic] (Férussac 1823: 149) poses a special case. It appeared first in the legend of plate 1 of the “Mélanopsides fossiles” in the “Histoire naturelle” but – if the format was the same as in the monograph – not in italic font as are the other names. In the monograph, it is the only term on the subspecific level Férussac gave in small caps, a format he otherwise used only for vernacular names of species. Because of this and the fact that he did not add “nobis” it is unlikely that Férussac wanted to introduce a new name; rather, he may have intended to indicate its status as a fossil (Latin *antiqua* = old). If it had been introduced as a distinct taxon, the name would have been invalid as an objective junior synonym of *Melanopsis fusiformis* Sowerby, 1822, which Férussac listed in synonymy.

Férussac (1822) also introduced two additional names (*inflata*, *elongata*) in the captions of plate 1 of the “Mélanopsides fossiles”, ranked below var. γ [“Antiquua”]. In the monograph (1823) they are clearly marked with “nobis”. If “*antiquua*” [= *fusiformis*] was considered as a distinct taxon, these names would be of infrasubspecfic rank, which is not governed by the Code. Both names would have nonetheless become available as species-group names after Art. 45.6.4.1 at least from Pallary (1916: 77), who used them as valid species names. At that time, however, both of them were junior homonyms and thus invalid.

Pallary (1916: 77–78) considered several of the descriptive Latin terms as available names, i.e., “*Melanopsis antiqua* Férussac”, “*Melanopsis affinis* Férussac”, “*Melanopsis magna* Férussac”, “*Melanopsis minuta* Férussac”, “*Melanopsis parva* Férussac” and “*Melanopsis subtuberculata* Férussac”. Since Pallary (1916) clearly associated those names with illustrations in Férussac (1823), they became available thereby, but with the authority “Pallary, 1916”. This actually also includes *Melanopsis antiqua*, which had been an unavailable name until then.

### Names introduced by Jean-Pierre Sylvestre de Grateloup in 1828 and 1838

A similar problem as for Férussac’s monograph appears in works by Grateloup (1828, 1838). He labelled varieties with Latin letters, occasionally followed by Latin terms (e.g., “*olivula*”) and Latin descriptions and sometimes marked with “Nob.”. In his 1828-work he attributed the Latin terms *minor* and *major* to varieties of two different species. Apparently, most of these terms were meant as keywords rather than real names. This becomes obvious also from Grateloup (1838), where he introduced the new name *Melanopsis gibbosula* for specimens treated as “var. b. *minor*” in Grateloup (1828).

In order to bring stability to the problem, I propose to use only the Latin terms marked by Grateloup with “Nob.” as available names.

### The taxonomic concepts applied by Jules René Bourguignat, Paul Pallary, Jean-Marie Pérès and Ferdinand Starmühlner

**Figure 1.**
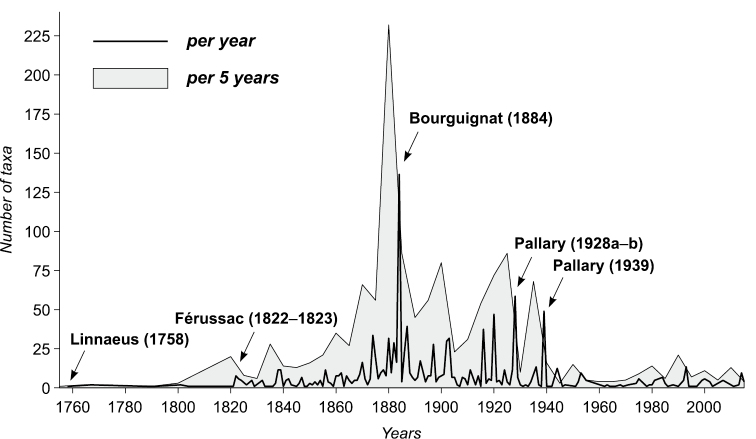
Numbers of melanopsid taxa described between 1758 and 2015 per year and per five years. The most important contributions, i.e., pioneer works and those with numerous new taxa described, are highlighted.

The works by Bourguignat and Pallary between 1853 and 1939 extended the list of melanopsid names enormously (Figure 1). Both authors applied very detailed taxonomic concepts and introduced dozens of species and varieties for even minor morphological deviations or differences in shell color. This procedure artificially inflated present-day melanopsid diversity, and few of those names are actually used today (Glaubrecht 2011). Nonetheless, most of the names are nomenclaturally available.

Unfortunately, Bourguignat and Pallary did not foresee that subspecies (as well as forma and variety names published before before 1961, Art. 10.2) would all become included by the Code in the species group, with the Principle of Homonymy applying throughout. Both malacologists introduced many varieties, such as *minor* and *major*, often several times for different species within the very same work. Pallary apparently considered some of the variety names he introduced as self-explanatory (e.g., Latin *minor* means “small”) and left them undescribed (see, e.g., Pallary 1904: 37). In order to avoid inflating the present catalogus with anyway unavailable and taxonomically hardly useful names, I chose not to include these nomina nuda.

Finally, Pérès (1938a, b, 1939, 1946) and Starmühlner (1970, 1973) applied elaborate systems to categorize morphologies and coloration patterns of recent melanopsids. Pérès introduced several “formae” and “modes” that he obviously did not use as valid names but rather as descriptive terms to fit existing species into his morphological concept (see, e.g., Pérès 1939: 135–137, Pérès 1946: 166). He even assigned “types” to his morpho-units (Pérès 1939: 137). Although hardly any of his new names have any meaning in nomenclature or taxonomy, the available names are included and discussed herein.

Starmühlner (1970, 1973) in a similar way classified New Caledonian specimens of *Melanopsis frustulum* according to form and size of the shell (“forma”), type and height of the spire (“modus”) and coloration (“coloratus”). None of the names used by Starmühlner are available in nomenclature because they were introduced after 1961 and are of infrasubspecific rank (Art. 10.2). They are not included in this catalogus.

### Note on the information provided

The catalogue lists all names in alphabetical order in the original spelling and combination, with the necessary amendments required by the Code. The status of taxa that are invalid, unavailable or unresolved is denoted in square brackets after the taxon name; those without status declaration are available and nomenclaturally valid, irrespective of their taxonomic status. The first description, or alternatively the basis of record for unavailable names, is always indicated. Taxa solely found as fossils are marked by a dagger. Taxon authorities attributed to a person other than one of the authors in the original source are only accepted as such if there is clear evidence that the description derived from that person (Art. 50.1.1). In such cases, the notation is given in accordance with Recommendation 51E. Information on type locality, type horizon (for fossils only) and type specimens are indicated as far as available. The exact spelling or phrase (given in quotes) provided for the type locality in the original source is denoted, along with an English translation if required. If the localities have been indicated indirectly (e.g., “same as for the previous species”), the phrase is given in square brackets. Old locality names have been matched with today’s geographic names as far as possible, mostly using the GeoNames geographical database (http://www.geonames.org/v3/). Places that could not be found on the map or where the matched name is uncertain are marked by a postposed question mark in the translation.

Note that Wenz (1929) in his Fossilium Catalogus quite often misstated type localities, which he indicated in spaced letters. For taxa where no type has been fixed and several localities have been mentioned in the original publication, he apparently preferred localities mentioned first or those indicated for illustrated specimens. After Art. 76.1 of the Code, in absence of a holotype or lectotype the type locality encompasses the localities of all syntypes. Illustrated specimens do not automatically qualify as holotypes or have any priority in this respect.

Information on type horizons follows the most recent age classifications found in the literature (e.g., Neubauer et al. 2015a, Georgopoulou et al. 2015 for the Neogene and Quaternary freshwater deposits of Europe). As far as possible, the present taxonomic status of a taxon is indicated, i.e., whether it has been considered a junior synonym by later authors. Data on type specimens are provided for all names where information is available, particularly for those published after 1999, when a type fixation became mandatory (Art. 16.4). If not indicated otherwise, the information provided regards the storage of the holotype. For names published by Spiridion Brusina and Petar S. Pavlović the inventory catalogues of the Croatian Natural History Museum in Zagreb (Milan et al. 1974) and the Natural History Museum in Belgrade (Milošević 1962) formed the basis. Note, however, that Milan et al. (1974) often wrongly indicated holotypes from syntype series, apparently considering illustrated specimens preferable to the rest of the material (see also discussion in Neubauer et al. 2016b). Milošević (1962) provided inventory numbers of the specimens illustrated by Pavlović; neither of both applied a type concept.

### Nomenclatural notes

The following list of species-group names comprises several recurrent nomenclatural issues. In order to save space, to avoid multiple elaborate repetitions of the same rules, and to prevent that the reader needs to consult the Code constantly, I refer in text to two nomenclatural notes which are defined as follows:

**Note 1**: Because of the Principle of Coordination (Art. 46), homonymy in the species-group does not depend on a taxon’s original rank in the species-group. This also encompasses variety and forma names published before 1961 (Art. 10.2). Many authors have been unaware that species names can constitute junior homonyms of subspecies, variety or forma names. Only in the case of simultaneously published names the taxon of higher rank takes precedence (Art. 24.1).

**Note 2**: Several names of Melanopsidae first occurred in synonymy lists of other names (see, e.g., Brot 1874–1879). According to Art. 11.6 of the Code, “a name which when first published in an available work was treated as a junior synonym of a name then used as valid is not thereby made available”. Some of the names, however, have been made indeed available following the provisions of Art. 11.6.1: “However, if such a name published as a junior synonym had been treated before 1961 as an available name and either adopted as the name of a taxon or treated as a senior homonym, it is made available thereby but dates from its first publication as a synonym [...]”.

#### † Melanopsis aaronsohni

Taxon classificationAnimaliaSorbeoconchaMelanopsidae

Blanckenhorn in Blanckenhorn & Oppenheim, 1927

##### Original source.

Blanckenhorn and Oppenheim 1927: 37, pl. 21, figs 14–15.

##### Type horizon.

Early Pleistocene.

##### Type locality.

“10 Minuten nördlich von der Station Djisr et-Medjâmi” (p. 34) [10 minutes north of the station Djisr et-Medjâmi (= ‘Erq el-Ahmar, also known as Gesher)], Israel.

#### † Melanopsis costata var. abbreviata

Taxon classificationAnimaliaSorbeoconchaMelanopsidae

Brusina, 1874

##### Original source.

Brusina 1874: 41, pl. 7, fig. 10.

##### Type horizon.

Cernikian, Pliocene.

##### Type locality.

“Podvinje (Čaplja) [Čaplja trench near Slavonski Brod]; Novska; Farkašić”, Croatia.

##### Types.

Milan et al. (1974: 89) indicated a holotype, but it is uncertain whether the specimen was the only one Brusina had at hand (holotype by monotypy, Art. 73.1.2). The specimen is stored in the Croatian Natural History Museum, Zagreb, coll. no. 3717-1357/1.

#### † Melanopsis abbreviata

Taxon classificationAnimaliaSorbeoconchaMelanopsidae

Pallary, 1916 [invalid]

##### Original source.

Pallary 1916: 81.

##### Type horizon.

Middle Lutetian, Eocene.

##### Type locality.

“Au Nord d’Albas” (Doncieux 1908: 204), France.

##### Remarks.

Replacement name for *Melanopsis brevis* Doncieux, 1908, non Sowerby, 1826. Junior homonym of *Melanopsis abbreviata* Brusina, 1874 (see *Melanopsis atacica* Wenz, 1928).

#### † Melanopsis abichi

Taxon classificationAnimaliaSorbeoconchaMelanopsidae

Calvert & Neumayr, 1880

##### Original source.

Calvert and Neumayr 1880: 376, pl. 2, figs 20–22.

##### Type horizon.

Late Sarmatian, Khersonian, late Miocene.

##### Type locality.

“Renkiöi” [north of İntepe], Turkey.

#### Melanopsis (Mesopotamia) mesopotamica var. abrahamiana

Taxon classificationAnimaliaSorbeoconchaMelanopsidae

Pallary, 1939

##### Original source.

Pallary 1939: 100, pl. 5, figs 1–2.

##### Type locality.

“‘Ain Arouss” [‘Ayn al ‘Arūs, near Tall Abyaḑ], Syria.

#### † “ Melanopsis martiniana abscissa

Taxon classificationAnimaliaSorbeoconchaMelanopsidae

Handmann” mentioned in Fischer (1996b: 20, fig. 2) [unavailable]

##### Horizon.

Pannonian, zone B–D, late Miocene.

##### Locality.

“Leobersdorf”, Austria.

##### Remarks.

Nomen nudum, based on an “in schedis” determination by Handmann (the corresponding label is illustrated in Fischer 1996b: 21, fig. 2).

#### † Melanopsis absyrtidum

Taxon classificationAnimaliaSorbeoconchaMelanopsidae

Stache in Sandberger, 1871

##### Original source.

Sandberger 1870–1875: 134, pl. 19, figs 13–13a.

##### Type horizon.

Cretaceous.

##### Type locality.

“Insel Unie bei Lussin” [Unije island], Croatia.

#### † Melanopsis (Canthidomus) acanthica

Taxon classificationAnimaliaSorbeoconchaMelanopsidae

Neumayr, 1869

##### Original source.

Neumayr 1869: 357, pl. 11, figs 6–7.

##### Type horizon.

Langhian, middle Miocene.

##### Type locality.

“Miocic” [Miočić], Croatia.

##### Types.

Illustrated syntypes are stored at the Geological Survey Austria, Vienna, coll. no. 1869/01/4/1-2.

#### † Melanopsis acanthicoides

Taxon classificationAnimaliaSorbeoconchaMelanopsidae

Hoernes, 1876

##### Original source.

Hoernes 1876: 14, figs 1–5.

##### Type horizon.

Late Sarmatian, Khersonian, late Miocene.

##### Type locality.

“Renkiöi” [north of İntepe], Turkey.

#### † Melanoptychia acanthicula

Taxon classificationAnimaliaSorbeoconchaMelanopsidae

Bourguignat, 1880

##### Original source.

Bourguignat 1880: 31.

##### Type horizon.

Langhian, middle Miocene.

##### Type locality.

“Vallée de la Cettina” [Cetina river valley], Croatia.

##### Remarks.

The name “*acanthinula*” as mentioned in Pallary (1926a: 74) is an incorrect subsequent spelling. Wenz (1929: 2650) considered the taxon as a junior synonym of *Melanopsis acanthica* Neumayr, 1869.

#### † Melanopsis (Martinia) martiniana var. accedens

Taxon classificationAnimaliaSorbeoconchaMelanopsidae

Handmann, 1887

##### Original source.

Handmann 1887: 24, pl. 3, figs 15–16.

##### Type horizon.

Pannonian, zone B–D, late Miocene.

##### Type locality.

“Leobersdorf”, Austria.

##### Remarks.

Wenz (1929: 2719) considered this taxon as a junior synonym of *Melanopsis fossilis* (Gmelin, 1791).

#### Melanopsis acicularis

Taxon classificationAnimaliaSorbeoconchaMelanopsidae

Férussac, 1823

##### Original source.

Férussac 1823: 160.

##### Type locality.

“La rivière de Laybach. [...] Les eaux thermales de Weslau, près de Vienne. [...] Le Danube, à Wissegrad et à Bude. [...] de l’île de Wight” [Ljubljanica river; in the thermal water of Vöslau near Vienna; in the Danube, at Visegrád and Budapest; from the Isle of Wight], Slovenia.

##### Remarks.

Partly a junior objective synonym of *Melanopsis subulata* Sowerby, 1822 (regarding the specimen of the Isle of Wight) and *Melanopsis daudebartii* [Prevost], 1821 (regarding the Vöslau material), which Férussac considered varieties of *Melanopsis acicularis* and listed in synonymy. Currently considered as a junior synonym or subspecies of *Microcolpia daudebartii* [Prevost], 1821, respectively (Welter-Schultes 2012: 36; Neubauer et al. 2014d).

#### Melanopsis aciculella

Taxon classificationAnimaliaSorbeoconchaMelanopsidae

Potiez & Michaud, 1838

##### Original source.

Potiez and Michaud 1838: 346, pl. 31, figs 3–4.

##### Type locality.

“Les rivières de la Carniole” [rivers of Carniola, a historical region that comprised parts of present-day Slovenia].

##### Remarks.

Starobogatov et al. (1992: 65) considered the species as a junior synonym of *Microcolpia cornea* (Pfeiffer, 1828).

#### † Turripontica aciculina

Taxon classificationAnimaliaSorbeoconchaMelanopsidae

Anistratenko, 1993

##### Original source.

Anistratenko 1993: 75, textfig. 3.

##### Type horizon.

Duab Beds, middle to late Kimmerian, Pliocene.

##### Type locality.

“Окр. с. Мокви, Очамчирский р-н” [near the village Mok’vi, Ochamchirskiy rayon], Georgia.

##### Types.

Schmalhausen Institute of Zoology of National Academy of Sciences of Ukraine, Kiev; no number indicated.

##### Remarks.

Type species of the genus *Turripontica* Anistratenko, 1993.

#### † Melanopsis acrolepta

Taxon classificationAnimaliaSorbeoconchaMelanopsidae

Fontannes, 1884

##### Original source.

Fontannes 1884: 29, pl. 4, figs 1–2.

##### Type horizon.

Early Rupelian, Oligocene.

##### Type locality.

“Barjac, Roméjac, Avéjan, Saint-Jean-de-Maruéjols, Célas, Issirac, Galès, près de Montclus (Gard)”, France.

#### Fagotia acroxia

Taxon classificationAnimaliaSorbeoconchaMelanopsidae

Bourguignat, 1884

##### Original source.

Bourguignat 1884: 48.

##### Type locality.

“Dans la rivière au-dessous de Krapina-Toeplitz, en Croatie” [in the river below Krapinske toplice], Croatia.

##### Remarks.

Note that Bourguignat denoted the authority as “Bourguignat, 1879”.

#### † Melanopsis acuminata

Taxon classificationAnimaliaSorbeoconchaMelanopsidae

Gümbel, 1861

##### Original source.

Gümbel 1861: 753.

##### Type horizon.

Chattian, Oligocene.

##### Type locality.

“In der oberen Leizach, an der Schlierach, im Sulzgraben, bei Pensberg, Rimselrain, im Höllbache, am hohen Peissenberge” [all localities are near Miesbach, southern Bavaria], Germany.

##### Remarks.

Gümbel attributed the authority to Sandberger, but there is no evidence that the description really derived from that author.

#### † Melanopsis bouei var. acuminata

Taxon classificationAnimaliaSorbeoconchaMelanopsidae

Handmann, 1882 [invalid]

##### Original source.

Handmann 1882: 557.

##### Type horizon.

Pannonian, zone D, late Miocene.

##### Type locality.

“Kottingbrunn [...] Ziegelei a”, Austria.

##### Remarks.

Junior homonym of *Melanopsis acuminata* Gümbel, 1861. Not included in the Fossilium Catalogus of Wenz (1929).

#### Melanopsis costata var. acuminata

Taxon classificationAnimaliaSorbeoconchaMelanopsidae

Bourguignat, 1884 [invalid]

##### Original source.

Bourguignat 1884: 140.

##### Type locality.

“Le Jourdain, à 4 kilomètres au-dessus de la Mer Morte” [Jordan river, 4 km north of the Dead Sea], Israel/Jordan.

##### Remarks.

Junior homonym of *Melanopsis acuminata* Gümbel, 1861.

#### † Melanopsis acuminata

Taxon classificationAnimaliaSorbeoconchaMelanopsidae

Pallary, 1901 [invalid]

##### Original source.

Pallary 1901a: 178, pl. 2, fig. 23.

##### Type horizon.

Plio-Pleistocene.

##### Type locality.

“Du puits Karoubi” [from the well Karoubi], Algeria.

##### Remarks.

Junior homonym of *Melanopsis acuminata* Gümbel, 1861. Wenz (1919a: 73) introduced *Melanopsis pallaryi* as replacement name (see also *Melanopsis raphidia* Pallary, 1920).

#### † Melanopsis acuminata

Taxon classificationAnimaliaSorbeoconchaMelanopsidae

Seninski, 1905 [invalid]

##### Original source.

Seninski 1905: 62, pl. 2, figs 3–4.

##### Type horizon.

Duab Beds, middle to late Kimmerian, Pliocene.

##### Type locality.

“Моквинскіе пласты, р. Дуабъ” [Mokvi layers at Duab river], Georgia.

##### Remarks.

Junior homonym of *Melanopsis acuminata* Gümbel, 1861. Wenz (1928a) introduced *Melanopsis seninskii* as replacement name.

#### † Melanopsis acuta

Taxon classificationAnimaliaSorbeoconchaMelanopsidae

Handmann, 1882

##### Original source.

Handmann 1882: 556.

##### Type horizon.

Pannonian, zone D, late Miocene.

##### Type locality.

“Kottingbrunn [...] Ziegelei a”, Austria.

#### Melanopsis acutespira

Taxon classificationAnimaliaSorbeoconchaMelanopsidae

Bourguignat, 1884

##### Original source.

Bourguignat 1884: 114.

##### Type locality.

“Dans l’aqueduc de la Palafanga, près Almazora (Espagne), aux environs de Mascara (Algérie)” [in the aqueduct Palafanga near Almazora (Spain), near Mascara (Algeria)].

##### Remarks.

Bourguignat introduced this species for formerly misidentified *Melanopsis dufourei* [sic] *graellsii* sensu Rossmässler, 1854 and *Melanopsis maroccana* sensu Paladilhe, 1875 as well as a part of the material of the variety *Melanopsis maroccana subgraellsiana* Bourguignat, 1864. He apparently overlooked that Paladilhe (1875) had not referred to *Melanopsis maroccana* but had introduced the new variety *Melanopsis maroccana zonatosubcostata*. Since there was never a holotype or lectotype defined, the name *acutespira* comprises the syntypes of all three works; it remains nomenclaturally valid as name for the two specimens illustrated by Rossmässler (1854) and Bourguignat (1864).

#### Melanopsis acutissima

Taxon classificationAnimaliaSorbeoconchaMelanopsidae

Gassies, 1871

##### Original source.

Gassies 1871: 197, pl. 6, fig. 13.

##### Type locality.

“Bélep (île Art)” [Bélep, Art Island], New Caledonia.

#### Melanopsis acutula

Taxon classificationAnimaliaSorbeoconchaMelanopsidae

Pallary, 1920

##### Original source.

Pallary 1920a: 31.

##### Type locality.

“Dans un bassin entre le Mellah et le pont; vers Dar Mahrès; [...] Bahlil (28 kil. au sud de Fès)” [in a basin between the Mellah and the bridge; toward Dar Mahres; Bhalil (28 km south of Fes)], Morocco.

#### Melanopsis adrarensis

Taxon classificationAnimaliaSorbeoconchaMelanopsidae

Pallary, 1912

##### Original source.

Pallary 1912: 16, figs 8–9.

##### Type locality.

“Dans les seguias des oasis du Touat, spécialement dans celles de l’Adrar” [in the irrigation channels of the oasis of Touat, especially those of Adrar], Algeria.

##### Remarks.

Given as “*adparensis*” on p. 16, but “*adrarensis*” in plate captions. Since Pallary clearly denoted it from the locality Adrar, the name must be “*adrarensis*” (Art. 32.5.1).

#### † Melanopsis aegea

Taxon classificationAnimaliaSorbeoconchaMelanopsidae

Tournouër, 1875

##### Original source.

Tournouër 1875: 76.

##### Type horizon.

Phoka to Elia Formation, Plio-Pleistocene.

##### Type locality.

“[Prope vicum Antimaki et prope civitatem Cos], in loco Hagios-Foukas” [near near the village Antimácheia and near the city of Kos, in the locality Ágios Fokás], Greece.

##### Remarks.

The name “*aegaea*” as mentioned in Tournouër (1876: 453), Wenz (1929: 2653) and Willmann (1981: 174) is an incorrect subsequent spelling.

#### † Melanopsis aegyptiaca

Taxon classificationAnimaliaSorbeoconchaMelanopsidae

Blanckenhorn, 1901

##### Original source.

Blanckenhorn 1901: 414, pl. 15, figs 15–16.

##### Type horizon.

Pleistocene.

##### Type locality.

“In der Fossilienbank an der Tewfik-Moschee bei Kairo. [...] Wadi Urag, Sanur, Moschasch, Raijade” [in the fossil deposits at the Tewfik Mosque near Cairo. In the wadis Urag (?), Sannūr, Moschasch (?), Raijade (?)], Egypt.

#### † Melanopsis aetolica

Taxon classificationAnimaliaSorbeoconchaMelanopsidae

Neumayr, 1876

##### Original source.

Neumayr 1876: 368.

##### Type horizon.

Gelasian, early Pleistocene.

##### Type locality.

“Stamna, nordwestlich von Missolunghi” [Stamná, NW of Mesolóngi], Greece.

##### Types.

Papp (1955: pl. 20, fig. 9) designated a specimen from the type material of Neumayr (1880a) as lectotype, but did not indicate whether it had been illustrated by Neumayr or not. A comparison of the images in both works was inconclusive. However, since it is likely that Neumayr (1880a) used the material reported in 1876 for the description, this designation is considered to be based on the original material and is thus valid. The specimen is stored in the Institute of Paleontology, University of Vienna; no number indicated.

##### Remarks.

The name became available from Neumayr (1876), where he briefly described the species. He nevertheless described the species as “new” in Neumayr (1880a: 126), providing a detailed description and illustrations (pl. 6, figs 13–17; note that the plate is inserted at the end of the previous article of the same volume).

#### † Melanopsis affinis

Taxon classificationAnimaliaSorbeoconchaMelanopsidae

Handmann, 1882

##### Original source.

Handmann 1882: 558.

##### Type horizon.

Pannonian, zone D, late Miocene.

##### Type locality.

“Kottingbrunn [...] Ziegelei a”, Austria.

##### Remarks.

Pallary (1916) considered the species a junior homonym of “*Melanopsis affinis* Férussac” and introduced *Melanopsis subaffinis* as replacement name. “*Melanopsis affinis* Férussac” is, however, not an available name (see below) and *Melanopsis subaffinis* is thus a junior objective synonym of *Melanopsis affinis* Handmann, 1882. Wenz (1929: 2674, 2681) synonymized both Handmann’s and Pallary’s species with *Melanopsis bouei* Férussac, 1823.

#### † Melanopsis affinis

Taxon classificationAnimaliaSorbeoconchaMelanopsidae

Pallary, 1916 [invalid]

##### Original source.

Pallary 1916: 78.

##### Type horizon.

Late Villafranchian, early Pleistocene.

##### Type locality.

“D’Italie” (Férussac 1823 had given the locality more precisely as “entre Otricoli et le Vigne, route de Rome à Foligno” []), Italy.

##### Remarks.

The name often appears as “*Melanopsis affinis* Férussac” in the literature (e.g., Wenz 1929: 2653; Esu and Girotti 1975: 251). It was first mentioned in Férussac (1814) as nomen nudum for a specimen from the Euphrates River. Later, Férussac (1823) listed the name in synonymy of *Melanopsis nodosa* Férussac, 1822. Unlike Pallary (1916) claimed and followed by Wenz (1929), *affinis* is not to be preferred over *nodosa*, because at that time *affinis* was not an available name – it has never been described, indicated or illustrated (as, for example, wrongly presumed by Esu and Girotti 1975) and so does not fulfill the requirements of availability (Art. 11 and 12; see also Art. 11.6). Pallary (1916) was the first to affiliate the name with an illustration, namely the one of *Melanopsis nodosa* provided by Férussac, 1823 (pl. 7, fig. 13), and made the name thereby available. The illustrated specimen is, however, the holotype (by monotypy) of *Melanopsis nodosa* Férussac, 1822 (Férussac used the same plates in both of his works and thus Pallary actually referred to the specimen illustrated as *Melanopsis nodosa* in the “Histoire naturelle”; see also introduction and Table 1 for details of Férussac’s publications). Consequently, *Melanopsis affinis* Pallary, 1916 is a junior objective synonym of *Melanopsis nodosa*. Moreover, the name is a junior homonym of *Melanopsis affinis* Handmann, 1882.

#### Melania afra

Taxon classificationAnimaliaSorbeoconchaMelanopsidae

Rossmässler, 1839

##### Original source.

Rossmässler 1838–1844: 38, pl. 50, fig. 665.

##### Type locality.

“Aus der Ringelsza bei Laibach” [Ringelsza (?) brook near Ljubljana], Slovenia.

##### Remarks.

Introduced in synonymy of *Melania holandri laevigata*. It was made available at least by Brusina (1867) who treated it as a valid name (see Note 2).

#### † Melanopsis matheroni var. agatensis

Taxon classificationAnimaliaSorbeoconchaMelanopsidae

Pantanelli, 1886

##### Original source.

Pantanelli 1886a: 68 or 1886b: 78, pl. 3, figs 1–4 (precedence not established).

##### Type horizon.

Tortonian–early Messinian, late Miocene.

##### Type locality.

“S. Valentino e S. Agata” [San Valentino and Sant’Agata Fossili], Italy.

##### Remarks.

Harzhauser et al. (2015: 9) considered the taxon as a junior synonym of *Melanopsis narzolina* d’Archiac in Viquesnel, 1846.

#### Melania holandrii var. agnata

Taxon classificationAnimaliaSorbeoconchaMelanopsidae

Pfeiffer, 1828

##### Original source.

Pfeiffer 1828: 47, pl. 8, fig. 8.

##### Type locality.

“In der Muhr” [in the river Mur], Austria or Slovenia.

##### Remarks.

Note that Bourguignat denoted the authority incorrectly as “Bourguignat, 1877”. The name “*aequata*” as mentioned in Clessin (1890: 676) is an incorrect subsequent spelling.

#### Melanella agnatella

Taxon classificationAnimaliaSorbeoconchaMelanopsidae

Bourguignat, 1884

##### Original source.

Bourguignat 1884: 14.

##### Type locality.

“Rivière près Zenica, en Bosnie” [river near Zenica], Bosnia and Herzegovina.

##### Remarks.

Appeared first as a nomen nudum in Servain (1884: 379).

#### “ Melanopsis agoroea

Taxon classificationAnimaliaSorbeoconchaMelanopsidae

” mentioned in Bourguignat (1884: 83) [unavailable]

##### Locality.

Not indicated.

##### Remarks.

Nomen nudum: Bourguignat (1884) listed the name in synonymy of *Melanopsis laevigata*, perhaps referring to an unused manuscript name. Probably the name was intended as “*agoraea*”, but the ligature was mixed up during typesetting.

#### Melanopsis ahuiri

Taxon classificationAnimaliaSorbeoconchaMelanopsidae

Ahuir Galindo, 2014

##### Original source.

Ahuir Galindo 2014b: 7, unnumbered figure.

##### Type locality.

“Around Guefait, at the Northeastern of Morocco”, Morocco.

##### Types.

Museo Malacologico di Cupra Marittima, Italy; no number indicated.

#### † Melanopsis ajkaensis

Taxon classificationAnimaliaSorbeoconchaMelanopsidae

Tausch, 1886

##### Original source.

Tausch 1886: 9, pl. 1, figs 35a–c.

##### Type horizon.

Late Santonian–early Campanian, late Cretaceous.

##### Type locality.

“Csingerthal bei Ajka” [Csinger valley near Ajka], Hungary.

#### † Melanopsis albasensis

Taxon classificationAnimaliaSorbeoconchaMelanopsidae

Wenz, 1919 [invalid]

##### Original source.

Wenz 1919a: 73.

##### Type horizon.

Middle Lutetian, Eocene.

##### Type locality.

“Au Nord d’Albas” (Doncieux 1908: 204), France.

##### Remarks.

Introduced as replacement name for the junior homonym *Melanopsis nodosa* Doncieux, 1908, non Férussac, 1822, for which Pallary (1916) had already introduced *Melanopsis doncieuxi* as replacement name. Thus, *Melanopsis albasensis* is a junior objective synonym of *Melanopsis doncieuxi* Pallary, 1916.

#### Melanopsis alepensis

Taxon classificationAnimaliaSorbeoconchaMelanopsidae

Pallary, 1939 [invalid]

##### Original source.

Pallary 1939: 86.

##### Remarks.

Unjustified emendation and therefore junior objective synonym of *Melanopsis alepi* Bourguignat, 1884.

#### Melanopsis alepi

Taxon classificationAnimaliaSorbeoconchaMelanopsidae

Bourguignat, 1884

##### Original source.

Bourguignat 1884: 119.

##### Type locality.

“Environs d’Alep” [surroundings of Aleppo], Syria.

##### Remarks.

Heller et al. (2005: 243) tentatively considered the species as a junior synonym of *Melanopsis costata* (Olivier, 1804).

#### Melanopsis algerica

Taxon classificationAnimaliaSorbeoconchaMelanopsidae

Pallary, 1904

##### Original source.

Pallary 1904: 35.

##### Type locality.

“Oran; [...] Mostaghanem” (Bourguignat 1864: caption of pl. 15; the exact localities where the illustrated specimens had been found are unknown), Algeria.

##### Remarks.

Introduced by indication of illustrations in Bourguignat (1864: pl. 15, figs 12–14, 17–18).

#### Melanopsis algericensis

Taxon classificationAnimaliaSorbeoconchaMelanopsidae

Pallary, 1922 [invalid]

##### Original source.

Pallary 1922: 207.

##### Remarks.

Unjustified emendation and therefore junior objective synonym of *Melanopsis algerica* Pallary, 1904.

#### † Melania (Campylostylus) allobroga

Taxon classificationAnimaliaSorbeoconchaMelanopsidae

Oppenheim, 1892

##### Original source.

Oppenheim 1892: 766, pl. 30, figs 5–7.

##### Type horizon.

Calcaire de Rognac, Maastrichtian, Cretaceous.

##### Type locality.

“Les Pennes, Valon du Duc bei Rognac” [Les Pennes, Valon du Duc near Rognac], France.

##### Remarks.

Originally the gender was incorrectly given as neutrum (“*allobrogum*”), but *Melania* is feminine.

#### † Melanopsis (Lyrcea) almerai

Taxon classificationAnimaliaSorbeoconchaMelanopsidae

Cossmann, 1909

##### Original source.

Cossmann 1909: 175.

##### Type horizon.

Eocene.

##### Type locality.

“Pyrénées catalanes”, Spain.

##### Remarks.

Cossmann (1909) gave a very brief description (“little keeled”) and made the name available.

#### † Melanopsis ancillaroides var. alpina

Taxon classificationAnimaliaSorbeoconchaMelanopsidae

Traub, 1938

##### Original source.

Traub 1938: 76, pl. 6, figs 8a–b.

##### Type horizon.

Paleocene.

##### Type locality.

“Aus Kch Fi1 [etwa 20 m unterhalb der großen betonierten Bachverbauung südöstlich von Kleinoiching am linken Ufer des Kroisbaches]” [from block Kch Fi1, ca. 20 m below the large concrete structure southeast of Kleinoiching, at the left bank of the Kroisbach brook], Austria.

#### † Melanopsis alutensis

Taxon classificationAnimaliaSorbeoconchaMelanopsidae

Stefanescu, 1896

##### Original source.

Stefanescu 1896: 128, pl. 11, figs 12–14.

##### Type horizon.

Early Pliocene.

##### Type locality.

“À Milcov, près de Slatina, dans la vallée de l’Oltu” [at Milcov, near Slatina, in the valley of the river Olt], Romania.

#### Melanopsis amabilis

Taxon classificationAnimaliaSorbeoconchaMelanopsidae

Pallary, 1928

##### Original source.

Pallary 1928a: 261, pl. 5, figs 19–20.

##### Type locality.

“Mechera Kredar, sur la route de Media à Larache; Bou Hellou (secteur ouest de Taza)” [Mechera Kredar (?), at the road from Mehdya to Larache; Bou Hellou, western part of prov. Taza], Morocco.

#### † Melanopsis hybostoma var. amaradica

Taxon classificationAnimaliaSorbeoconchaMelanopsidae

Fontannes, 1887

##### Original source.

Fontannes 1887: 337, pl. 26, figs 20–22.

##### Type horizon.

Early Cernikian, early Pliocene.

##### Type locality.

“Caprenu, val. Amaradii (Jud. Gorjiu)” [Căpreni], Romania.

#### † “ Melanopsis ambigua

Taxon classificationAnimaliaSorbeoconchaMelanopsidae

Gaudry, 1862” mentioned in Neumayr (1869: 372) [unavailable]

##### Horizon.

Pliocene?

##### Locality.

Not indicated.

##### Remarks.

Status unclear: the name was mentioned in Neumayr (1869: 372) and Pallary (1926a: 75), but the original description could not be found. Perhaps it is a lapsus calami of *Melanopsis anceps* Gaudry & P. Fischer in Gaudry, 1867.

#### Melanella amblya

Taxon classificationAnimaliaSorbeoconchaMelanopsidae

Bourguignat, 1884

##### Original source.

Bourguignat 1884: 24.

##### Type locality.

“La Save à Sissek, en Slavonie” [in the Sava river near Sisak in Slavonia], Croatia.

##### Remarks.

Note that Bourguignat denoted the authority as “Bourguignat, 1879”.

#### † Melanopsis americana

Taxon classificationAnimaliaSorbeoconchaMelanopsidae

White, 1883

##### Original source.

White 1883: 96, pl. 4, figs 9–10.

##### Type horizon.

Laramie Group, Cretaceous.

##### Type locality.

“Valley of South Platte River, Northeastern Colorado”, United States.

##### Remarks.

Probably not a Melanopsidae.

#### Melanopsis ammanensis

Taxon classificationAnimaliaSorbeoconchaMelanopsidae

Pallary, 1939 [invalid]

##### Original source.

Pallary 1939: 88, pl. 4, fig. 3.

##### Remarks.

Unjustified emendation and therefore junior objective synonym of *Melanopsis ammonis* Tristram, 1865.

#### Melanopsis ammonis

Taxon classificationAnimaliaSorbeoconchaMelanopsidae

Tristram, 1865

##### Original source.

Tristram 1865: 542.

##### Type locality.

“Streams at Heshbon and Ammon, east of Jordan”, Jordan.

#### “ Melanopsis amori

Taxon classificationAnimaliaSorbeoconchaMelanopsidae

” mentioned in Azpeitia Moros (1929: 40, 250) [unavailable]

##### Locality.

“Pedroche (arroyo de), en el partido judicial de Pozoblanco (Córdoba)” [Pedroche stream, district Pozoblanco, Córdoba], Spain.

##### Remarks.

Based on a manuscript name from Hidalgo and introduced in synonymy of *Melanopsis etrusca* (see Art. 11.6).

#### † Melanopsis amphora

Taxon classificationAnimaliaSorbeoconchaMelanopsidae

Oppenheim, 1890

##### Original source.

Oppenheim 1890a: 136, pl. 5, figs 2–2b.

##### Type horizon.

Ronca Beds, Bartonian, Eocene.

##### Type locality.

“Lovara di Tressino, Monte Pulli, Mussolon” [Lovara, Monte Pulli (near Valdagno), Muzzolon], Italy.

##### Remarks.

Wenz (1929: 2846) considered this taxon as a junior synonym of *Melanopsis vicentina* Oppenheim, 1890.

#### † Melanopsis ampla

Taxon classificationAnimaliaSorbeoconchaMelanopsidae

Pallary, 1920

##### Original source.

Pallary 1920b: 109.

##### Type horizon.

Middle Pannonian, late Miocene.

##### Type locality.

“Sulzlacke bei Margarethen nächst Oedenburg [...]. Tinnye bei Ofen” (Fuchs 1873: 20) [Sulzlacke near St. Margarethen (Burgenland, Austria); Tinnye (Hungary)].

##### Remarks.

Replacement name for *Melanopsis avellana* Fuchs, 1873, non Sandberger, 1870. Wenz (1929: 2760) considered the taxon as a junior synonym of *Melanopsis inermis* Handmann, 1882.

#### Melanopsis marocana [sic] var. ampla

Taxon classificationAnimaliaSorbeoconchaMelanopsidae

Pallary, 1920

##### Original source.

Pallary 1920c: 145.

##### Type locality.

“Tazouta”, Morocco.

##### Remarks.

This name, published in November 1920, is a junior homonym of *Melanopsis ampla* Pallary, 1920 [July].

#### † Melanopsis textilis var. ampullacea

Taxon classificationAnimaliaSorbeoconchaMelanopsidae

Handmann, 1887

##### Original source.

Handmann 1887: 16.

##### Type horizon.

Pannonian, zone B–D, late Miocene.

##### Type locality.

“Leobersdorf”, Austria.

#### Fagotia anatolica

Taxon classificationAnimaliaSorbeoconchaMelanopsidae

Bourguignat, 1884

##### Original source.

Bourguignat 1884: 43.

##### Type locality.

“Rivières près Ismidt (Anatolie)” [rivers near İzmit], Turkey.

##### Remarks.

Note that Bourguignat denoted the authority as “Bourguignat, 1880”. Starobogatov et al. (1992: 63) considered the species as a junior synonym of *Fagotia* [= *Esperiana*] *gallandi* Bourguignat, 1884.

#### † Melanopsis anceps

Taxon classificationAnimaliaSorbeoconchaMelanopsidae

Gaudry & P. Fischer in Gaudry, 1867

##### Original source.

Gaudry 1862–1867: 446, pl. 62, figs 1–6.

##### Type horizon.

Pliocene.

##### Type locality.

“Mégare” (p. 444), Greece.

#### Fagotia anceyana

Taxon classificationAnimaliaSorbeoconchaMelanopsidae

Bourguignat, 1884

##### Original source.

Bourguignat 1884: 36.

##### Type locality.

“Dans la Save au-dessous d’Agram, dans la rivière de Krapina (Croatie); enfin, dans le lac Sabandja près d’Ismidt (Anatolie)” [in the Sava river below Zagreb, in the river Krapina (Croatia); finally, in Lake Sapanca near İzmit (Turkey)].

##### Remarks.

Starobogatov et al. (1992: 60) considered the species as a junior synonym of *Fagotia* [= *Esperiana*] *esperi* (Férussac, 1823).

#### † Melanopsis ancillaroides

Taxon classificationAnimaliaSorbeoconchaMelanopsidae

Deshayes, 1825

##### Original source.

Deshayes 1824–1837: 121, pl. 15, figs 1–2.

##### Type horizon.

Cuisian, late Ypresian, Eocene.

##### Type locality.

“Les environs de Meaux” [surroundings of Meaux], France.

#### Melanopsis andersoni

Taxon classificationAnimaliaSorbeoconchaMelanopsidae

Pallary, 1923

##### Original source.

Pallary 1923: 41, [unnumbered plate], figs 1–2.

##### Type locality.

“Dans l’Ain-Touagha, à Fatnassa dans le Nefzaoua” [Ain Touagha (?) at Fatnassa in Nefzaoua], Tunisia.

#### † Melanopsis andrussowi

Taxon classificationAnimaliaSorbeoconchaMelanopsidae

Brusina, 1885

##### Original source.

Brusina 1885: 160.

##### Type horizon.

*Spaniodon* Beds, Karaganian, middle Miocene.

##### Type locality.

“Лопушны” (Sinzov 1884: 4) [Lăpuşna], Moldova.

##### Remarks.

Introduced for *Melanopsis lanzaeana* sensu Sinzov, 1884, non Brusina, 1874.

#### † Melanopsis angulata

Taxon classificationAnimaliaSorbeoconchaMelanopsidae

Neumayr, 1880

##### Original source.

Neumayr 1880c: 479, pl. 7, fig. 8.

##### Type horizon.

Langhian, middle Miocene.

##### Type locality.

“Žepj” [Džepi], Bosnia and Herzegovina.

##### Types.

The type material, with all specimens studied by Neumayr (1880), is lost. Neubauer et al. (2016c: 275) defined a neotype based on material from the type locality. The specimen is stored in the Geological-Paleontological Department, Natural History Museum Vienna, coll. no. 2014/0364/0012.

#### † Melanopsis angusta

Taxon classificationAnimaliaSorbeoconchaMelanopsidae

Pallary, 1916

##### Original source.

Pallary 1916: 82.

##### Type horizon.

Pannonian, zone D, late Miocene.

##### Type locality.

“Kottingbrunn [...] Ziegelei a” (Handmann 1882: 561), Austria.

##### Remarks.

Replacement name for *Melanopsis fusiformis* Handmann, 1882, non Sowerby, 1822. Wenz (1929: 2742) considered the taxon as a junior synonym of *Melanopsis haueri* Handmann, 1882.

#### † Melanopsis (Canthidomus) hybostoma anili

Taxon classificationAnimaliaSorbeoconchaMelanopsidae

Taner, 1997

##### Original source.

Taner 1997: 39.

##### Type horizon.

Early Romanian, Pliocene.

##### Type locality.

“Südöstlich vom Hügel Başaltı, 2,2 km W Musaköy, 15 km NE Çanakkale, W-Anatolien” [southeast of hill Başaltı, 2.2 km W of Musaköy, 15 km NE Çanakkale, W Anatolia], Turkey.

##### Types.

Geological-Paleontological Department, Natural History Museum Vienna, Austria, coll. no. 1996/0053/0001.

#### † Melanopsis anistratenkoi

Taxon classificationAnimaliaSorbeoconchaMelanopsidae

Neubauer, Harzhauser, Georgopoulou, Mandic & Kroh, 2014

##### Original source.

Neubauer et al. 2014a: 456.

##### Type horizon.

Duab Beds, middle to late Kimmerian, Pliocene.

##### Type locality.

“Окр. с. Мокви, Очамчирский р-н” (Anistratenko 1993: 69) [near the village Mok’vi, Ochamchirskiy rayon], Georgia.

##### Types.

Schmalhausen Institute of Zoology of National Academy of Sciences of Ukraine, Kiev, coll. no. 22/VI 1989.

##### Remarks.

Replacement name for the junior secondary homonym *Melanopsis cylindrica* Anistratenko, 1993, non *Lyrcea cylindrica* Stoliczka, 1862.

#### † Melanopsis anita

Taxon classificationAnimaliaSorbeoconchaMelanopsidae

Aldrich, 1886

##### Original source.

Aldrich 1886: 35, pl. 5, fig. 12.

##### Type horizon.

Early Eocene.

##### Type locality.

“Gregg’s Landing, Alabama”; Harris (1899, p. 77, pl. 10, fig. 11) designated a lectotype, United States.

##### Types.

Harris (1899: 77) designated a lectotype, which is stored in the collection of the National Museum of Natural History, Smithsonian Institution, Washington, DC, coll. no. 638955 (Palmer and Brann 1966: 755).

##### Remarks.

The taxon does not belong to the Melanopsidae after Allmon (1990: 63).

#### Melanopsis nodosa var. anodifera

Taxon classificationAnimaliaSorbeoconchaMelanopsidae

Cerulli-Irelli, 1914

##### Original source.

Cerulli-Irelli 1914: 185, pl. 15 (47), fig. 7.

##### Type locality.

“M. Mario”, Italy.

##### Remarks.

Girotti (1972: 232) considered the taxon as a junior synonym of “*Melanopsis affinis* Férussac”, which is not an available name.

#### † Bulimus antidiluvianus

Taxon classificationAnimaliaSorbeoconchaMelanopsidae

Poiret, 1801

##### Original source.

Poiret 1801: 36, 37.

##### Type horizon.

Sparnacian, early Ypresian, Eocene.

##### Type locality.

“Chemin de Soissons à Château-Thierry” [way of Soissons at Château-Thierry], France.

##### Remarks.

The name “*antediluviana*” as mentioned in Wenz (1929: 2658) is an incorrect subsequent spelling.

#### † Melanopsis antiqua

Taxon classificationAnimaliaSorbeoconchaMelanopsidae

Pallary, 1916

##### Original source.

Pallary 1916: 78.

##### Type horizon.

Late Villafranchian, Pleistocene.

##### Type locality.

“D’Italie” (Férussac 1823 had given the locality more precisely as “entre St.-Germini et Carsoli” [between San Gemini and Carsoli, Italy]).

##### Remarks.

The name “*Melanopsis antiqua* Férussac, 1823 has been used as valid name by several authors, but obviously was not intended as species-group name by Férussac (1823). The name became available from Pallary (1916) who associated the name with an illustration in Férussac (1823: pl. 8, fig. 2). See introduction for detailed discussion about the names used by Férussac (1823).

#### Melanopsis aperta

Taxon classificationAnimaliaSorbeoconchaMelanopsidae

Gassies, 1861

##### Original source.

Gassies 1861: 291, pl. 7, fig. 11.

##### Type locality.

“Jengen, dans les ruisseaux” [in streams at Hienghène], New Caledonia.

#### † Melanopsis bleicheri var. apicula

Taxon classificationAnimaliaSorbeoconchaMelanopsidae

Pallary, 1901

##### Original source.

Pallary 1901a: 178, pl. 2, fig. 26.

##### Type horizon.

Plio-Pleistocene.

##### Type locality.

“Puits Karoubi” [from the well Karoubi], Algeria.

#### Melanopsis graellsii var. apiculata

Taxon classificationAnimaliaSorbeoconchaMelanopsidae

Pallary, 1924

##### Original source.

Pallary 1924: 250, pl. 25, fig. 15.

##### Type locality.

“Almenara (entre Castellón et Valencia)” [Almenara, between Castellón and Valencia], Spain.

#### † Melanopsis turriformis applanata

Taxon classificationAnimaliaSorbeoconchaMelanopsidae

Schütt in Schütt & Ortal, 1993

##### Original source.

Schütt and Ortal 1993: 92, pl. 2, figs 21–23.

##### Type horizon.

Pleistocene, Mindel glacial epoch.

##### Type locality.

“Galilee, ‘Ubeidiya [El ‘Ubeidīya], 3 km SE of the Sea of Galilee”, Israel.

##### Types.

Paleontology Collection of the Hebrew University of Jerusalem; no number indicated.

#### Melanopsis cossoni var. aprica

Taxon classificationAnimaliaSorbeoconchaMelanopsidae

Bourguignat, 1884

##### Original source.

Bourguignat 1884: 112.

##### Type locality.

“Dans les Seguia des jardins de Miliana, premier ksar au nord de l’oasis d’Insalah, dans le Sahara” [in the irrigation channel (?) in the gardens of Miliana, first ksar (= fortified village, castle) north of the oasis of In Salah in the Sahara], Algeria.

#### † Melanopsis aquensis

Taxon classificationAnimaliaSorbeoconchaMelanopsidae

Grateloup, 1838

##### Original source.

Grateloup 1838: 139, pl. 4, figs 48–49.

##### Type horizon.

Burdigalian, early Miocene.

##### Type locality.

“Dax; Mandillot, à Saint-Paul”, France.

##### Remarks.

Wenz (1929) synonymized the name with “*Melanopsis major* Férussac, 1823”, which is not an available name.

#### † Melanopsis aquitanica

Taxon classificationAnimaliaSorbeoconchaMelanopsidae

Pallary, 1916

##### Original source.

Pallary 1916: 79.

##### Type horizon.

Burdigalian, early Miocene.

##### Type locality.

“Dax, St-Paul, Mandillot” (Grateloup 1840: captions of the plate “Mollusques terrestres et fluviatiles fossiles de Dax”), France.

##### Remarks.

Introduced for *Melanopsis dufourii* sensu Grateloup, 1840, non Férussac, 1822. Wenz (1929: 2662) considered the taxon as a junior synonym of “*Melanopsis major* Férussac, 1823”, which is not an available name.

#### Melanopsis scalaris var. arbalensis

Taxon classificationAnimaliaSorbeoconchaMelanopsidae

Pérès, 1939

##### Original source.

Pérès 1939: 144, pl. 4, figs 15, 19–20.

##### Type locality.

“Stations 3, 4, 1 3, 29, 35, 143. Oued Tizguit; [...] Station 109. Aïn Aghbal près d’Azrou” [Stations 3, 4, 1 3, 29, 35, 143 at Oued Tizguit; station 109 at Ain Aghbal near Azrou], Morocco.

#### † Melanopsis arcuata

Taxon classificationAnimaliaSorbeoconchaMelanopsidae

Brusina, 1878

##### Original source.

Brusina 1878: 348.

##### Type horizon.

Cernikian, Pliocene.

##### Type locality.

“Malino”, Croatia.

##### Types.

The syntypes are stored in the Croatian Natural History Museum, Zagreb; no number indicated (Milan et al. 1974: 85).

##### Remarks.

Pallary (1925) introduced *Melanopsis simulata* as replacement name for the “junior homonym” *Melanopsis arcuata* Brusina, 1878, non Matheron, 1842, which is not available name but an incorrect subsequent spelling of *Melanopsis armata* Matheron, 1842.

#### † Melanopsis armata

Taxon classificationAnimaliaSorbeoconchaMelanopsidae

Matheron, 1842

##### Original source.

Matheron 1842: 294, pl. 37, figs 12–14.

##### Type horizon.

Calcaire de Rognac, Maastrichtian, Cretaceous.

##### Type locality.

“Rognac et St. Victoret”, France.

##### Remarks.

The name “*arcuata*” as mentioned in Pallary (1925: 257) is an incorrect subsequent spelling. Based on this error, Pallary (1925) considered *Melanopsis arcuata* Brusina, 1897 a junior homonym and introduced the replacement name *Melanopsis simulata* (junior objective synonym). Sandberger (1871: 101) attributed the species to the genus *Paludomus* Swainson, 1840 (Paludomidae). After Calzada and Urquiola (1994: 152) this species belongs in the genus *Cosinia* Stache, 1880 (Paludomidae).

#### Melanopsis (Mesopotamia) aroussiana

Taxon classificationAnimaliaSorbeoconchaMelanopsidae

Pallary, 1939

##### Original source.

Pallary 1939: 100, pl. 5, figs 22–25, 56.

##### Type locality.

“‘Ain Arouss” [‘Ayn al ‘Arūs, near Tall Abyaḑ], Syria.

#### † Melanopsis arsinovi

Taxon classificationAnimaliaSorbeoconchaMelanopsidae

Brusina, 1902

##### Original source.

Brusina 1902: pl. 6, figs 2–4.

##### Type horizon.

Early Langhian, middle Miocene.

##### Type locality.

“Zvezdanski ključ” [village Zvezdan], Serbia.

##### Types.

The illustrated syntypes are stored in the Croatian Natural History Museum, Zagreb, coll. no. 2503-149/1-3 (Milan et al. 1974: 86).

#### Fagotia ascanica

Taxon classificationAnimaliaSorbeoconchaMelanopsidae

Bourguignat, 1884

##### Original source.

Bourguignat 1884: 39.

##### Type locality.

“Lac Sabandja” [Lake Sapanca], Turkey.

##### Remarks.

Note that Bourguignat denoted the authority as “Bourguignat, 1880”.

#### Melanopsis ascanica

Taxon classificationAnimaliaSorbeoconchaMelanopsidae

Bourguignat, 1884

##### Original source.

Bourguignat 1884: 96.

##### Type locality.

“Lac Sabandja, près d’Ismidt (Anatolie)” [Lake Sapanca near İzmit], Turkey.

##### Remarks.

Appeared first as a nomen nudum in Locard (1883a: 201). Note that Bourguignat denoted the authority as “Bourguignat, 1880”.

#### Melanopsis ashkhabadensis

Taxon classificationAnimaliaSorbeoconchaMelanopsidae

Izzatullaev & Starobogatov, 1984

##### Original source.

Izzatullaev and Starobogatov 1984: 1480, fig. 1 (14), fig. 3 (8).

##### Type locality.

“Ашхабад, горные быстро текущие ручьи - Ферюза, Гули и др.” [Ashgabat, fast mountain streams - Firyuza, Guli and others”], Turkmenistan.

##### Types.

Zoological Institute of Russian Academy of Sciences, St.-Petersburg; no number indicated.

#### † Melanopsis arbalensis f. assakaensis

Taxon classificationAnimaliaSorbeoconchaMelanopsidae

Jodot, 1965 [unavailable]

##### Original source.

Jodot 1965: 104, fig. 2.

##### Type horizon.

Quaternary.

##### Type locality.

“Oued Assaka” [Oued Asaca], Morocco.

##### Remarks.

Unavailable for two reasons: First, the original work lacks a verbal description of the taxon which is required for names published after 1930 (Art. 13.1.1). Second, the taxon was introduced after 1961 as “forma” which is deemed to be infrasubspecific after Art. 15.2.

#### † Melanopsis astathmeta

Taxon classificationAnimaliaSorbeoconchaMelanopsidae

Brusina, 1897

##### Original source.

Brusina 1897: 8, pl. 6, figs 13–16.

##### Type horizon.

Cernikian, Pliocene.

##### Type locality.

“Malino”, Croatia.

##### Types.

Milan et al. (1974: 86) indicated a holotype, but it is uncertain whether the specimen was the only one Brusina had at hand (holotype by monotypy, Art. 73.1.2). The specimen is stored in the Croatian Natural History Museum, Zagreb, coll. no. 3000-646.

#### † Melanopsis astrapaea

Taxon classificationAnimaliaSorbeoconchaMelanopsidae

Brusina, 1876

##### Original source.

Brusina 1876: 110.

##### Type horizon.

Early Langhian, middle Miocene.

##### Type locality.

Originally given as “Sinj” and later specified as “Župića potok” in Brusina (1884b), Croatia.

##### Types.

Milan et al. (1974: 86) stated that Brusina (1897) had indicated one of the specimens illustrated by him (pl. 4, fig. 1) as type. However, it is uncertain whether the specimen actually derives from the original type series. The specimen is stored in the Croatian Natural History Museum, Zagreb, coll. no. 2968-614/1-2.

#### Melanopsis praemorsa var. astropaliae

Taxon classificationAnimaliaSorbeoconchaMelanopsidae

Gambetta, 1929

##### Original source.

Gambetta 1929: 104.

##### Type locality.

“Stampalia” [Astypalaia Island], Greece.

#### † Melanopsis atacica

Taxon classificationAnimaliaSorbeoconchaMelanopsidae

Wenz, 1928

##### Original source.

Wenz 1928a: 119.

##### Type horizon.

Lutetian, Eocene.

##### Type locality.

“Au Nord d’Albas” (Doncieux 1908: 204), France.

##### Remarks.

Replacement name for *Melanopsis doncieuxi* Wenz, 1919, non Pallary, 1916, which in turn was introduced as replacement name for *Melanopsis brevis* Doncieux, 1908, non Sowerby, 1826 (see also *Melanopsis abbreviata* Pallary, 1916).

#### † Melanopsis (Lyrcaea) slavonica var. atanasiui

Taxon classificationAnimaliaSorbeoconchaMelanopsidae

Șoverth, 1953

##### Original source.

Șoverth 1953: 206, pl. 3, figs d-d’’.

##### Type horizon.

Romanian, Pliocene–early Pleistocene.

##### Type locality.

“Hurezanii-de-Sus - Hurezanii-de-jos”, Romania.

#### Melanopsis aterrima

Taxon classificationAnimaliaSorbeoconchaMelanopsidae

Bourguignat, 1884

##### Original source.

Bourguignat 1884: 127.

##### Type locality.

“Dans deux ou trois sources de la plaine de Jéricho (Syrie)” [in two or three sources of the plain of Jericho], Palestine.

##### Remarks.

Heller et al. (2005: 248) considered the species as a junior synonym of *Melanopsis saulcyi* Bourguignat, 1853.

#### Melanopsis chehirensis var. atra

Taxon classificationAnimaliaSorbeoconchaMelanopsidae

Pallary, 1939

##### Original source.

Pallary 1939: 94.

##### Type locality.

Not explicitly stated but probably the same as for the species (“Dans le source de Yeni Chehir, [...] entre Antioche et Alep, à l’intersection de la route d’Alexandrette” [at the source of Yenişehir, between Antakya and Aleppo, at the intersection of the road from İskenderun], Turkey).

#### Melanopsis atramentaria

Taxon classificationAnimaliaSorbeoconchaMelanopsidae

Pallary, 1939

##### Original source.

Pallary 1939: 91, pl. 5, fig. 29.

##### Type locality.

“D’Acharné, sur l’Oronte, entre Hama et Kalâat el Moudik” [from Acharne at the Orontes, between Hama and Qal’at al Maḑīq], Syria.

#### † ? Melanopsis attenuata

Taxon classificationAnimaliaSorbeoconchaMelanopsidae

Sowerby in Fitton, 1836

##### Original source.

Fitton 1836: 228, 346, pl. 22, fig. 5.

##### Type horizon.

Weald Clay, early Cretaceous.

##### Type locality.

“Punfield”, United Kingdom.

##### Remarks.

After Sandberger (1870: 57) this species belongs in the genus *Goniobasis* Lea, 1862 (Pleuroceridae).

#### Melanopsis nobilis var. attenuata

Taxon classificationAnimaliaSorbeoconchaMelanopsidae

Pallary, 1912 [invalid]

##### Original source.

Pallary 1912: 21, fig. 43.

##### Type locality.

“Des bords du Chott Djerid, à Tozeur” [banks of the Chott el Djérid at Tozeur], Tunisia.

##### Remarks.

Junior homonym of *Melanopsis attenuata* Sowerby in Fitton, 1836.

#### Melanopsis attenuata

Taxon classificationAnimaliaSorbeoconchaMelanopsidae

Pallary, 1920 [invalid]

##### Original source.

Pallary 1920a: 29.

##### Type locality.

“Tétouan”, Morocco.

##### Remarks.

Junior homonym of *Melanopsis attenuata* Sowerby in Fitton, 1836.

#### Melanopsis aurantiaca

Taxon classificationAnimaliaSorbeoconchaMelanopsidae

Gassies, 1874

##### Original source.

Gassies 1874: 383.

##### Type locality.

“Bourail et Nékété” [Bourail and Nakéty], New Caledonia.

#### † Melanopsis austriaca

Taxon classificationAnimaliaSorbeoconchaMelanopsidae

Handmann, 1882

##### Original source.

Handmann 1882: 560.

##### Type horizon.

Pannonian, zone D, late Miocene.

##### Type locality.

“Kottingbrunn [...] Ziegelei a”, Austria.

##### Remarks.

Wenz (1929: 2741) considered the taxon as a junior synonym of *Melanopsis haueri* Handmann, 1882.

#### † Melanopsis avellana

Taxon classificationAnimaliaSorbeoconchaMelanopsidae

Sandberger, 1870

##### Original source.

Sandberger 1870–1875: pl. 5, figs 15–15a.

##### Type horizon.

Maastrichtian, Cretaceous.

##### Type locality.

“Auzas”, France.

##### Remarks.

Plate 5 of Sandberger’s monograph was issued in 1870, while the description on p. 110 appeared in 1871 (Woodward 1906).

#### † Melanopsis avellana

Taxon classificationAnimaliaSorbeoconchaMelanopsidae

Fuchs, 1873 [invalid]

##### Original source.

Fuchs 1873: 20, pl. 4, figs 16–17.

##### Type horizon.

Middle Pannonian, late Miocene.

##### Type locality.

“Sulzlacke bei Margarethen nächst Oedenburg [...]. Tinnye bei Ofen” [Sulzlacke near St. Margarethen (Burgenland, Austria); Tinnye (Hungary)].

##### Remarks.

Junior homonym of *Melanopsis avellana* Sandberger, 1870. Pallary (1920b) introduced *Melanopsis ampla* as replacement name.

#### Melanopsis lorcana var. aynensis

Taxon classificationAnimaliaSorbeoconchaMelanopsidae

Azpeitia Moros, 1929

##### Original source.

Azpeitia Moros 1929: 146, pl. 2, figs 33–35.

##### Type locality.

“Del río Mundo, cerca de Ayna (Albacete)” [in Mundo river, near Ayna, prov. Albacete], Spain.

#### † Melanopsis baconica

Taxon classificationAnimaliaSorbeoconchaMelanopsidae

Oppenheim, 1892

##### Original source.

Oppenheim 1892: 770, pl. 34, figs 10–10b.

##### Type horizon.

Late Santonian–early Campanian, late Cretaceous.

##### Type locality.

“Csingerthal” (locality only given in plate captions) [near Ajka], Hungary.

##### Remarks.

Bandel and Riedel (1994: 19) considered this taxon as a junior synonym of *Melanopsis ajkaensis* Tausch, 1886.

#### † Melanopsis (Canthidomus) balatonensis

Taxon classificationAnimaliaSorbeoconchaMelanopsidae

Cossmann, 1909

##### Original source.

Cossmann 1909: 178.

##### Type horizon.

Transdanubian, Pannonian, late Miocene.

##### Type locality.

“Vörös-Bereny im Hohlweg nächst des Füzfö-major und in Kenese [...] Fonyód; [...] Szt-György-hegy in Hegymagyos” (Halaváts 1903: 49) [Vörösberény, in the hollow-way near the Füzfö-major (?) and in Balatonkenese; Fonyód; Szent György-hegy in Hegymagas], Hungary.

##### Remarks.

Replacement name for *Melanopsis boettgeri* Halaváts, 1903, non Klika, 1891.

#### † Melanopsis banatica

Taxon classificationAnimaliaSorbeoconchaMelanopsidae

Jekelius, 1944

##### Original source.

Jekelius 1944: 132, pl. 50, figs 3–8, 12–14.

##### Type horizon.

Early Pannonian, late Miocene.

##### Type locality.

“Turislav-Tal bei Soceni” [Turislav valley near Soceni], Romania.

#### † Melanoptychia banatica

Taxon classificationAnimaliaSorbeoconchaMelanopsidae

Jekelius, 1944 [invalid]

##### Original source.

Jekelius 1944: 139, pl. 58, figs 11–12.

##### Type horizon.

Early Pannonian, late Miocene.

##### Type locality.

“Turislav-Tal bei Soceni” [Turislav valley near Soceni], Romania.

##### Remarks.

Junior secondary homonym of *Melanopsis banatica* Jekelius, 1944 (same work). Neubauer et al. (2014a: 462) considered both synonymous and gave priority to *Melanopsis banatica* (since *Melanoptychia* is considered as a synonym of *Melanopsis*).

#### Melanopsis praemorsa bandeli

Taxon classificationAnimaliaSorbeoconchaMelanopsidae

Schütt, 1988

##### Original source.

Schütt 1988b: 216, pl. 1, fig. 7.

##### Type locality.

Unclear: Schütt referred to two different localities: “Wasserfall 1 km oberhalb der Straßenbrücke Jerash - Amman über den Zarqa” (p. 216) [waterfall 1 km above the bridge over the Zarqā’ along the road Jerash to Amman] and “Fluß Zerqa bei der alten Brücke am King Talal-See” (p. 219) [Zarqā’ river at the old bridge at the King Talal Dam], Jordan.

##### Types.

Natural History Museum Vienna, Austria, coll. no. 85.544.

##### Remarks.

Heller et al. (2005: 248) considered the variety as a junior synonym of *Melanopsis saulcyi* Bourguignat, 1853.

#### † Melanopsis banovaci

Taxon classificationAnimaliaSorbeoconchaMelanopsidae

Brusina, 1902

##### Original source.

Brusina 1902: pl. 6, figs 67–68.

##### Type horizon.

Middle Pannonian, late Miocene.

##### Type locality.

“Kúp”, Hungary.

##### Types.

Milan et al. (1974: 87) indicated a holotype, but it is uncertain whether the specimen was the only one Brusina had at hand (holotype by monotypy, Art. 73.1.2). The specimen is stored in the Croatian Natural History Museum, Zagreb, coll. no. 2527-173.

#### Melanopsis barbini

Taxon classificationAnimaliaSorbeoconchaMelanopsidae

Pallary, 1911

##### Original source.

Pallary 1911: 129, [unnumbered plate], figs 4–5.

##### Type locality.

“Tout près d’Oudjda, à 4 kilom. S.-E., sourdent les belles sources de Sidi-Yahia qui alimentent une véritable oasis, puis la ville d’Oudjda, et vont finalement se déverser dans l’oued Isly” [near Oujda, 4 km southeast, at the sources of Sidi Yahya that feed an oasis and the city of Oujda, and ultimately will flow into the Oued Isly], Morocco.

#### † Melanopsis barthai

Taxon classificationAnimaliaSorbeoconchaMelanopsidae

Bandel, 2000

##### Original source.

Bandel 2000: 160, figs 43–44, 46–51.

##### Type horizon.

Late Pannonian, late Miocene.

##### Type locality.

“Sand pit near Papkesi [Papkeszi]”, Hungary.

##### Types.

Geological-Palaeontological Institute and Museum University of Hamburg, coll. no. 4268.

#### † Melanopsis bartolinii

Taxon classificationAnimaliaSorbeoconchaMelanopsidae

Capellini, 1873

##### Original source.

Capellini 1873: 550, pl. 8, figs 1–4.

##### Type horizon.

Messinian, late Miocene.

##### Type locality.

“Sterza di Laiatico”, Italy.

##### Remarks.

Illustration not on pl. 7 as indicated by Capellini (1873). The name “*bartolini*” as mentioned in Ligios et al. (2012: 358) is an incorrect subsequent spelling.

#### Melanopsis bedei

Taxon classificationAnimaliaSorbeoconchaMelanopsidae

Pallary, 1933

##### Original source.

Pallary 1933: 248.

##### Type locality.

“L’Oued Bou Regreg, au pont des Seouls” [in the Oued Bou Regreg, at the bridge of the Séouls (?)], Morocco.

#### † Melanopsis belestensis

Taxon classificationAnimaliaSorbeoconchaMelanopsidae

Villatte, 1952

##### Original source.

Villatte 1952: 329, pl. 2, figs 16–21.

##### Type horizon.

Campanian, Cretaceous.

##### Type locality.

“Bélesta (Ariège)”, France.

#### Melanopsis belonidaea

Taxon classificationAnimaliaSorbeoconchaMelanopsidae

Bourguignat, 1884

##### Original source.

Bourguignat 1884: 110.

##### Type locality.

“Ruisseau d’eau chaude à Ouargla (prov. de Constantine) et eaux thermales du Djérid, au nord du chott Tiraoun (sud de la Tunisie)” [in a warm water stream in Ouargla (Algeria) and thermal waters of Djerid, north of chott Tiraoun (southern Tunisia)].

##### Remarks.

Multiple spellings occur in the original work: “*belonidoea*” on p. 110 but “*belonidaea*” on p. 75. Apparently, the spelling on p. 110 is based on a typesetting mistake regarding the ligature (“œ” instead of “æ”). Letourneux & Bourguignat (1887) acted as First Reviser sensu Art. 24.2.2, giving the name as “*belonidaea*”. The spellings “*balonidaea*” mentioned in Westerlund (1886: 125) and “*belonidae*” given in Pallary (1912: 14, 19, 22; 1926: 75) are incorrect spellings.

#### Melanopsis belusi

Taxon classificationAnimaliaSorbeoconchaMelanopsidae

Bourguignat, 1884

##### Original source.

Bourguignat 1884: 134.

##### Type locality.

“Du Bélus, près de Saint-Jean-d’Acre (Syrie)” [in the Na’aman river, near Acre], Israel.

##### Remarks.

Bourguignat (1884) denoted the authority as “Letourneux, 1882”, but there is no evidence that the description really derived from that author. Heller et al. (2002: 596) considered the species as a junior synonym of *Melanopsis costata* (Olivier, 1804). Heller et al. (2005: 244) in turn treated it as a junior synonym of *Melanopsis lampra* Bourguignat, 1884.

#### Melanopsis belusiensis

Taxon classificationAnimaliaSorbeoconchaMelanopsidae

Germain, 1921 [invalid]

##### Original source.

Germain 1921: 463.

##### Remarks.

Unjustified emendation and therefore junior objective synonym of *Melanopsis belusi* Bourguignat, 1884.

#### † Melanopsis (Calodiona) bergeroni

Taxon classificationAnimaliaSorbeoconchaMelanopsidae

Stefanescu, 1896

##### Original source.

Stefanescu 1896: 131, pl. 11, figs 32–37.

##### Type horizon.

Sienisian to Pelendavian, Pliocene.

##### Type locality.

“À Gura-Motrului et à Bocovatz, dans la vallée de Jiu” [at Gura-Motrului and at Bucovăț, in the valley of the river Jiu], Romania.

#### Melanopsis magnifica var. berkanensis

Taxon classificationAnimaliaSorbeoconchaMelanopsidae

Pallary, 1911

##### Original source.

Pallary 1911: 128, [unnumbered plate], fig. 12.

##### Type locality.

“Près du village de Berkane, à la lisière Sud-Ouest de la plaine des Triffas, tout au Nord des Beni-Znassen, [...] source connue sous le nom berbère d’Aoûllout” [near Berkane, to the southwestern edge of the plain of Triffa, just north of Beni Snassene, in a spring known as Aïn Aoullout], Morocco.

#### Fagotia berlani

Taxon classificationAnimaliaSorbeoconchaMelanopsidae

Bourguignat, 1884

##### Original source.

Bourguignat 1884: 34.

##### Type locality.

“Le Danube à Ibraila; la Save entre Agram et Sissek” [Danube river at Brăila (Romania); Sava river between Zagreb and Sisak (Croatia)].

##### Remarks.

Note that Bourguignat denoted the authority as “Bourguignat, 1880”.

#### Melanella berlani

Taxon classificationAnimaliaSorbeoconchaMelanopsidae

Bourguignat, 1884

##### Original source.

Bourguignat 1884: 22.

##### Type locality.

“Le Danube près Ibraila” [Danube river at Brăila], Romania.

##### Remarks.

Note that Bourguignat denoted the authority as “Bourguignat, 1879”.

#### † Melanopsis textilis var. bicarinata

Taxon classificationAnimaliaSorbeoconchaMelanopsidae

Handmann, 1887

##### Original source.

Handmann 1887: 16.

##### Type horizon.

Pannonian, zone B–D, late Miocene.

##### Type locality.

“Leobersdorf”, Austria.

#### Melanopsis mellalensis var. bicarinata

Taxon classificationAnimaliaSorbeoconchaMelanopsidae

Pallary, 1928 [invalid]

##### Original source.

Pallary 1928b: 19, pl. 2, figs 22–23.

##### Type locality.

“De Béni Mellal; de l’oued Daï” [from Beni Mellal; from Oued Daï (?)], Morocco.

##### Remarks.

Junior homonym of *Melanopsis textilis bicarinata* Handmann, 1882 (see Note 1).

#### † Melanopsis doboi bicarinata

Taxon classificationAnimaliaSorbeoconchaMelanopsidae

Schréter, 1975 [invalid]

##### Original source.

Schréter 1975: 8, pl. 2, figs 7–10.

##### Type horizon.

Riss/Würm end to early Würm Ice Age, Pleistocene.

##### Type locality.

“Eger, az egri vár Zárkándy bástyájának átmetszése” [Eger, section at the Zarkandy bastion of the fortress Eger], Hungary.

##### Types.

Magyar Állami Földtani Intézet (Hungarian Geological Museum), Budapest; no number indicated.

##### Remarks.

Junior homonym of *Melanopsis textilis bicarinata* Handmann, 1882 (see Note 1). Neubauer et al. (2016a) attributed the species *Melanopsis doboi* to the genus *Microcolpia*.

#### † Melanopsis bicincta

Taxon classificationAnimaliaSorbeoconchaMelanopsidae

Blanckenhorn, 1897

##### Original source.

Blanckenhorn 1897: 119, pl. 9, figs 35–40.

##### Type horizon.

Plio-Pleistocene.

##### Type locality.

“In der zweiten Thonbank des linken Orontesufers und der ersten und zweiten des rechten Ufers” [in the second clay bank at the left riverside of the Orontes and the first and second bank of the right riverside], Syria?

##### Remarks.

Introduced as “n. mut.” but clearly as a binomen and hence not infrasubspecific in the sense of ICZN Art. 45.6.

#### † Melanopsis tothi var. bicingulata

Taxon classificationAnimaliaSorbeoconchaMelanopsidae

Brusina, 1903

##### Original source.

Brusina 1903: 115.

##### Type horizon.

Late Pleistocene–early Holocene.

##### Type locality.

“Bischofsbad” [Püspökfürdő, Băile 1 Mai, Lake Pețea], Romania.

##### Remarks.

Neubauer et al. (2014d: 125) considered this taxon as a junior synonym of *Microcolpia parreyssii sikorai* (Brusina, 1903).

#### Melanopsis praerosa [sic] var. bicolorata

Taxon classificationAnimaliaSorbeoconchaMelanopsidae

Paetel, 1888

##### Original source.

Paetel 1888: 403.

##### Type locality.

“W. of Shiraz” (Nevill 1884: 209), Iran.

##### Remarks.

Originally introduced as infrasubspecific taxon (“subvariety”) by Nevill (1884), but made available by Paetel (1888) who treated it as variety (Art. 45.5.1). Paetel clearly referred to the description of Nevill.

#### † Melanopsis (Martinia) capulus var. biconica

Taxon classificationAnimaliaSorbeoconchaMelanopsidae

Handmann, 1887

##### Original source.

Handmann 1887: 21, pl. 2, figs 16–18.

##### Type horizon.

Pannonian, zone B–D, late Miocene.

##### Type locality.

“Leobersdorf”, Austria.

#### † Melanopsis bicoronata

Taxon classificationAnimaliaSorbeoconchaMelanopsidae

Brusina, 1884

##### Original source.

Brusina 1884b: 56.

##### Type horizon.

Early Langhian, middle Miocene.

##### Type locality.

“Stuparuša” (p. 47) [near Sinj], Croatia.

##### Types.

Milan et al. (1974: 87) stated that Brusina (1897) had indicated one of the specimens illustrated by him (pl. 4, fig. 15) as type. However, it is uncertain whether the specimen actually derives from the original type series. The specimen is stored in the Croatian Natural History Museum, Zagreb, coll. no. 2975-621/1.

#### † Melanopsis hazayi var. bifilosa

Taxon classificationAnimaliaSorbeoconchaMelanopsidae

Brusina, 1903 [unresolved]

##### Original source.

Brusina 1903: 113.

##### Type horizon.

Late Pleistocene–early Holocene.

##### Type locality.

“Bischofsbad” [Püspökfürdő, Băile 1 Mai, Lake Pețea], Romania.

##### Remarks.

Brusina (1903) introduced several varieties with this name, apparently considering it only a descriptive term; the homonymy issue needs to be solved by a First Reviser. Currently, all of them are considered junior synonyms of *Microcolpia parreyssii sikorai* (Brusina, 1903) (Neubauer et al. 2014d: 125).

#### † Melanopsis sikorai var. bifilosa

Taxon classificationAnimaliaSorbeoconchaMelanopsidae

Brusina, 1903 [unresolved]

##### Original source.

Brusina 1903: 112.

##### Type horizon.

Late Pleistocene–early Holocene.

##### Type locality.

“Bischofsbad” [Püspökfürdő, Băile 1 Mai, Lake Pețea], Romania.

##### Remarks.

Brusina (1903) introduced several varieties with this name, apparently considering it only a descriptive term; the homonymy issue needs to be solved by a First Reviser. Currently, all of them are considered junior synonyms of *Microcolpia parreyssii sikorai* (Brusina, 1903) (Neubauer et al. 2014d: 125).

#### † Melanopsis themaki var. bifilosa

Taxon classificationAnimaliaSorbeoconchaMelanopsidae

Brusina, 1903 [unresolved]

##### Original source.

Brusina 1903: 110.

##### Type horizon.

Late Pleistocene–early Holocene.

##### Type locality.

“Bischofsbad” [Püspökfürdő, Băile 1 Mai, Lake Pețea], Romania.

##### Remarks.

Brusina (1903) introduced several varieties with this name, apparently considering it only a descriptive term; the homonymy issue needs to be solved by a First Reviser. Currently, all of them are considered junior synonyms of *Microcolpia parreyssii sikorai* (Brusina, 1903) (Neubauer et al. 2014d: 125).

#### † “ Melanopsis tothi var. bifilosa

Taxon classificationAnimaliaSorbeoconchaMelanopsidae

” mentioned in Brusina (1903: 114) [unavailable]

##### Horizon.

Late Pleistocene–early Holocene.

##### Locality.

“Bischofsbad” [Püspökfürdő, Băile 1 Mai, Lake Pețea], Romania.

##### Remarks.

Nomen nudum (Brusina apparently considered the term self-explanatory). Brusina (1903) introduced several varieties with this name, apparently considering it only a descriptive term. Neubauer et al. (2014d: 125) considered this taxon as a junior synonym of *Microcolpia parreyssii sikorai* (Brusina, 1903).

#### Hemisinus acicularis var. biharensis

Taxon classificationAnimaliaSorbeoconchaMelanopsidae

Clessin, 1890

##### Original source.

Clessin 1887–1890: 683, fig. 467.

##### Type locality.

“In der warmen Quelle bei Robogany im Bihargebirge in Ungarn” [in the thermal spring near Răbăgani in the Bihar Mts], Romania.

##### Remarks.

Based on an “in schedis” name from Hazay.

#### Melanopsis crenocarinata var. bilineata

Taxon classificationAnimaliaSorbeoconchaMelanopsidae

Moricand, 1841

##### Original source.

Moricand 1841: 62.

##### Type locality.

“Rio de Pedra Branca, procince de Bahia” [Pedra Branca river, province Bahia], Brazil.

##### Remarks.

Although not explicitly stated, this variety was apparently considered to belong to the new genus *Verena* by Adams and Adams (1854) (Thiaridae), of which *Melanopsis crenocarinata* is the type species (see Nuttall 1990: 253).

#### † Melanopsis biliottii

Taxon classificationAnimaliaSorbeoconchaMelanopsidae

Bukowski, 1892

##### Original source.

Bukowski 1892: 249.

##### Type horizon.

Apolakkia/Monolithos Formation, Pliocene.

##### Type locality.

“Rhodos” (locality specified as “Monolithos” in Bukowski 1893), Greece.

##### Remarks.

The names “*billiottii*” and “*biolittii*” as mentioned in Pallary (1926a: 76) and Wenz (1929: 2667) are incorrect subsequent spellings.

#### † Melanopsis binodosa

Taxon classificationAnimaliaSorbeoconchaMelanopsidae

Blanckenhorn, 1897

##### Original source.

Blanckenhorn 1897: 117, pl. 9, figs 28–34.

##### Type horizon.

Plio-Pleistocene.

##### Type locality.

“Aus der zweiten Thonbank des rechten und linken Orontesufers; obere Thonbank des rechten Ufers” [from the second clay bank of the right and left riversides of the Orontes; upper clay bank of the right riverside], Syria?

##### Remarks.

Introduced as “n. mut.” but clearly as a binomen and hence not infrasubspecific in the sense of ICZN Art. 45.6.

#### Melanopsis bullio var. bipartita

Taxon classificationAnimaliaSorbeoconchaMelanopsidae

Dautzenberg, 1894

##### Original source.

Dautzenberg 1894: 344.

##### Type locality.

“Lac de Homs”, Syria.

#### † Melanopsis bittneri

Taxon classificationAnimaliaSorbeoconchaMelanopsidae

Fuchs, 1877

##### Original source.

Fuchs 1877: 40, pl. 4, figs 12–17.

##### Type horizon.

Chaudian, late Pleistocene.

##### Type locality.

“Livonates bei Talandi” (p. 36) [Livanates], Greece.

#### † Melanoptychia bittneri

Taxon classificationAnimaliaSorbeoconchaMelanopsidae

Neumayr, 1880 [invalid]

##### Original source.

Neumayr 1880c: 480, pl. 7, fig. 11.

##### Type horizon.

Langhian, middle Miocene.

##### Type locality.

“Žepj” [Džepi], Bosnia and Herzegovina.

##### Remarks.

Neubauer et al. (2013: 135) considered *Melanoptychia* as a junior synonym of *Melanopsis* and this species as a junior secondary homonym of *Melanopsis bittneri* Fuchs, 1877. They introduced *Melanopsis medinae* as replacement name (see also *Melanoptychia carusi* Brusina, 1902).

#### † Melanopsis bittneri

Taxon classificationAnimaliaSorbeoconchaMelanopsidae

Brusina, 1902 [invalid]

##### Original source.

Brusina 1902: pl. 7, figs 18–21.

##### Type horizon.

Langhian, middle Miocene.

##### Type locality.

“Džepe” [Džepi], Bosnia and Herzegovina.

##### Remarks.

Junior homonym of *Melanopsis bittneri* Fuchs, 1877. Neubauer et al. (2013: 134) considered it as a junior synonym of *Melanopsis cvijici* Brusina, 1902.

#### † Melanopsis blanchardi

Taxon classificationAnimaliaSorbeoconchaMelanopsidae

Brusina, 1892

##### Original source.

Brusina 1892: 143.

##### Type horizon.

Middle Pannonian, late Miocene.

##### Type locality.

“Markuševec”, Croatia.

##### Types.

Milan et al. (1974: 100) indicated a holotype, but it is uncertain whether the specimen actually derives from the original type series and whether it was the only specimen Brusina had at hand. The specimen is stored in the Croatian Natural History Museum, Zagreb, coll. no. 2539-185.

#### † Melanopsis blanckenhorni

Taxon classificationAnimaliaSorbeoconchaMelanopsidae

Wenz, 1929

##### Original source.

Wenz 1929: 2667.

##### Type horizon.

Pleistocene.

##### Type locality.

“Dans le lac d’Antioche, dans l’oued Baradah près Aïn Fidji, et dans l’Aïn Plaça, fontaine de la plaine du Bahr-el-Houlé” (Bourguignat 1884: 82) [in Lake Anuk (also as Amik), in the river Barada near Aïn al-Fiji, and in Aïn el Placa, a spring in the plains of the Hula valley], Syria.

##### Remarks.

Replacement name for *Melanopsis prophetarum minor* Bourguignat, 1884, non Grateloup, 1838 (see Note 1). The type locality given by Wenz (“U. Orontestal, 7 km unterhalb Antâkîje”) is incorrect.

#### † Melanopsis blanckenhorni

Taxon classificationAnimaliaSorbeoconchaMelanopsidae

Schütt, 1988 [invalid]

##### Original source.

Schütt 1988a: 137, pl. 3, figs 18–20.

##### Type horizon.

Plio-Pleistocene.

##### Type locality.

“Höhere Lage am westlichen Orontes-Ufer 12 km S Ǧisr aš-Šugur” [western riverside of the Orontes, 12 km south of Jisr Ash-Shughur], Syria.

##### Types.

Senckenberg Forschungsinstitut und Naturmuseum Frankfurt, coll. no. SMF 307206.

##### Remarks.

Junior homonym of *Melanopsis blanckenhorni* Wenz, 1929. Heller and Sivan (2001: 131) considered the species as junior synonym of *Melanopsis costata* (Olivier, 1804).

#### † Purpuroidea blaschkei

Taxon classificationAnimaliaSorbeoconchaMelanopsidae

Schenk, 1969

##### Original source.

Schenk 1969: 39, pl. 1, fig. 15.

##### Type horizon.

Late Santonian to early Campanian, late Cretaceous.

##### Type locality.

“Zöttbachalm bei Brandenberg, Tirol” [Zöttbachalm near Brandenberg, Tyrol], Austria.

##### Remarks.

The species was originally attributed to the marine genus *Purpuroidea* Lycett, 1848 (Purpuroideidae), but considered as a junior synonym of the melanopsid *Megalonoda spiniger* (Sowerby in Sedgwick & Murchison, 1832) by Kollmann (1984: 58).

#### Melanopsis bleicheri

Taxon classificationAnimaliaSorbeoconchaMelanopsidae

Paladilhe, 1874

##### Original source.

Paladilhe 1874: 403, pl. 8, figs 23–25.

##### Type locality.

“Environs d’Oran” [surroundings of Oran], Algeria.

#### † Melanopsis bleunardi

Taxon classificationAnimaliaSorbeoconchaMelanopsidae

Porumbaru, 1881

##### Original source.

Porumbaru 1881: 29, pl. 9, fig. 6.

##### Type horizon.

Early Romanian, Pliocene.

##### Type locality.

“Bucovatzu” [Bucovăț], Romania.

##### Remarks.

Wenz (1929) erroneously gave the town of Craiova as type locality, near which Bucovăț lies.

#### † Hydrobia bodeica

Taxon classificationAnimaliaSorbeoconchaMelanopsidae

Tausch, 1886

##### Original source.

Tausch 1886: 12, pl. 1, figs 47a–c.

##### Type horizon.

Late Santonian–early Campanian, late Cretaceous.

##### Type locality.

“Csingerthal bei Ajka” [Csinger valley near Ajka], Hungary.

##### Remarks.

Bandel and Riedel (1994: 20) considered this taxon as a juvenile specimen and as a junior synonym of *Melanopsis ajkaensis* Tausch, 1886.

#### † Melanopsis boettgeri

Taxon classificationAnimaliaSorbeoconchaMelanopsidae

Klika, 1891

##### Original source.

Klika 1891: 112, fig. 111a–d.

##### Type horizon.

Oligocene.

##### Type locality.

“Wärzen; [...] Tuchořic” [Dvérce; Tuchořice], Czech Republic.

#### † Melanopsis conemenosiana var. boettgeri

Taxon classificationAnimaliaSorbeoconchaMelanopsidae

Oppenheim, 1891 [invalid]

##### Original source.

Oppenheim 1891: 470.

##### Type horizon.

Plio-Pleistocene.

##### Type locality.

“Preveza in Epirus”, Greece.

##### Remarks.

Junior homonym of *Melanopsis boettgeri* Klika, 1891. Pallary (1920b: 112) introduced Melanopsis conemenosiana var. turritella as replacement name.

#### † Melanopsis (Pauluccia) boettgeri

Taxon classificationAnimaliaSorbeoconchaMelanopsidae

Brusina, 1902 [invalid]

##### Original source.

Brusina 1902: pl. 29, figs 5–8.

##### Type horizon.

Transdanubian, Pannonian, late Miocene.

##### Type locality.

“Radmanest” [Rădmănești], Romania.

##### Types.

The syntypes are stored in the Croatian Natural History Museum, Zagreb; no number indicated (Milan et al. 1974: 99).

##### Remarks.

Junior homonym of *Melanopsis boettgeri* Klika, 1891. Pallary (1920b) introduced *Melanopsis delicata* as replacement name.

#### † Melanopsis boettgeri

Taxon classificationAnimaliaSorbeoconchaMelanopsidae

Halaváts, 1903 [invalid]

##### Original source.

Halaváts 1903: 49, pl. 2, fig. 14.

##### Type horizon.

Transdanubian, Pannonian, late Miocene.

##### Type locality.

“Vörös-Bereny im Hohlweg nächst des Füzfö-major und in Kenese [...] Fonyód; [...] Szt-György-hegy in Hegymagyos” [Vörösberény, in the hollow-way near the Füzfö-major (?) and in Balatonkenese; Fonyód; Szent György-hegy in Hegymagas], Hungary.

##### Remarks.

Junior homonym of *Melanopsis boettgeri* Klika, 1891. Cossmann (1909) introduced *Canthidomus balatonensis* as replacement name.

#### Melanopsis bofilliana

Taxon classificationAnimaliaSorbeoconchaMelanopsidae

Bourguignat, 1884

##### Original source.

Bourguignat 1884: 101.

##### Type locality.

“Environs de Lorca, en Espagne” [surroundings of Lorca], Spain.

##### Remarks.

The name “*bofolliana*” as mentioned in Westerlund (1886: 126) is an incorrect subsequent spelling.

#### † Melanopsis bogdanowi

Taxon classificationAnimaliaSorbeoconchaMelanopsidae

Brusina, 1892

##### Original source.

Brusina 1892: 142.

##### Type horizon.

Middle Pannonian, late Miocene.

##### Type locality.

“Markuševec”, Croatia.

##### Types.

The illustrated syntypes are stored in the Croatian Natural History Museum, Zagreb, coll. no. 2538-184/1-3 (Milan et al. 1974: 100).

##### Remarks.

The name “*bogdanovi*” as mentioned in Wenz (1929: 2868) is an incorrect subsequent spelling.

#### “ Melanopsis bondeensis

Taxon classificationAnimaliaSorbeoconchaMelanopsidae

Gassies” mentioned in Paetel (1888: 399) and Pallary (1926a: 76) [unavailable]

##### Locality.

“Bondé”, New Caledonia.

##### Remarks.

Nomen nudum, found only in species lists by Paetel (1888) and Pallary (1926a). Perhaps confused with “*Bulimus bondeensis*” from the same locality, which Gassies (1871: 203) listed in the plate captions right below the *Melanopsis* species.

#### † Melanopsis bonellii

Taxon classificationAnimaliaSorbeoconchaMelanopsidae

Manzoni, 1870

##### Original source.

Manzoni 1870: 498, pl. 3, figs 8–9.

##### Type horizon.

Late Miocene to Pliocene.

##### Type locality.

“Mte. Gibio nel Modanese ed a St. Agata nel Tortonese; [...] a Sogliano” [Mt. Gibio, Sant’Agata Fossili, Sogliano al Rubicone], Italy.

##### Remarks.

In the old literature the name appears frequently as “*Melanopsis bonellii* Sismonda”. The name is not available from Sismonda (1847: 55), however, who solely referred to a misidentified name (*Melanopsis carinata* non Sowerby) in the unpublished museum catalogue of Bonelli (1827) but did not supply a description. It appears as a nomen nudum also in d’Orbigny (1852: 28) and Doderlein (1863: 16). Hörnes (1856: 595) mentioned the name along with two other melanopsids from Italy and gave a collective description for all of them (translated from German: “all individuals are smaller as the Viennese [*Melanopsis martiniana*], as well as more elongate, but they have the characteristic marginal bulge and the typical keel below the suture”). Following Art. 12.1, every name [...] must be accompanied by a description or a definition of the taxon that it denotes [...], which is not the case for the mentioning of *Melanopsis bonellii* in Hörnes (1856) because the description given there refers to a group of taxa and not a single taxon. Manzoni (1870) made the name available by providing an illustration (Art. 12.2.7).

The name “*bonelli*” as mentioned in Sacco (1889: 65) and Syrides (1998: 173) is an incorrect subsequent spelling.

#### Melanella letourneuxi var. bosnica

Taxon classificationAnimaliaSorbeoconchaMelanopsidae

Servain, 1884

##### Original source.

Servain 1884: 379.

##### Type locality.

“Migliaska” [Miljacka river near Sarajevo], Bosnia and Herzegovina.

#### † Melanopsis bouei

Taxon classificationAnimaliaSorbeoconchaMelanopsidae

Férussac, 1823

##### Original source.

Férussac 1819–1851: Mélanopsides fossiles, pl. 2 (1823), figs 9–10 or Férussac 1823: 159, pl. 8, figs 9–10 (precedence not established; see introduction for discussion).

##### Type horizon.

Pannonian, late Miocene.

##### Type locality.

“Près de Bisentz, dans la vallée de la Marsch, en Moravie; [...] près de Scharditz” [near Bzenec, in the valley of the March river, in Moravia; near Šardice], Czech Republic.

##### Types.

Papp (1953b) designated the specimen illustrated in Férussac (1823, pl. 8, fig. 9) as lectotype (after Art. 74.5). Whereabouts of type specimen unknown.

#### Fagotia bourguignati

Taxon classificationAnimaliaSorbeoconchaMelanopsidae

Bourguignat, 1884

##### Original source.

Bourguignat 1884: 48.

##### Type locality.

“Dans la rivière de Krapina et dans celle entre Plaski et Ostaria, en Croatie” [in the Krapina river and that between Plaški and Oštarije], Croatia.

##### Remarks.

Bourguignat denoted the authority as “Letourneux, 1884”, but there is no evidence that the description really derived from that author. Starobogatov et al. (1992: 60) considered the species as a junior synonym of *Fagotia* [= *Esperiana*] *esperi* (Férussac, 1823).

#### Melanella bourguignati

Taxon classificationAnimaliaSorbeoconchaMelanopsidae

Bourguignat, 1884

##### Original source.

Bourguignat 1884: 26.

##### Type locality.

“Rivière d’Ogulin (Croatie); la Save, à Sissek (Slavonie)” [river at Ogulin; Sava river at Sisak], Croatia.

##### Remarks.

Bourguignat denoted the authority as “Letourneux, 1879”, but there is no evidence that the description really derived from that author.

#### Melanopsis bovieri

Taxon classificationAnimaliaSorbeoconchaMelanopsidae

Pallary in Germain, 1921

##### Original source.

Germain 1921: 504, pl. 20, figs 7–8.

##### Type locality.

“Le Nahr ez Zaïr (Liban)” [locality not found], Lebanon.

##### Remarks.

Heller et al. (2005: 245) considered the species as a junior synonym of *Melanopsis lampra* Bourguignat, 1884.

#### Melanopsis brachymorpha

Taxon classificationAnimaliaSorbeoconchaMelanopsidae

Pallary, 1936

##### Original source.

Pallary 1936: 58, pl. 3, fig. 4.

##### Type locality.

“Aïn Akseri Ifesfassen à Imouzer d’Agadir” [Aïn Akseri Ifesfassen at Imouzzer near Agadir], Morocco.

#### † Melanopsis brachyptycha

Taxon classificationAnimaliaSorbeoconchaMelanopsidae

Neumayr, 1880

##### Original source.

Neumayr 1880c: 478, pl. 7, fig. 3.

##### Type horizon.

Pannonian, late Miocene.

##### Type locality.

“Posušje”, Bosnia and Herzegovina.

##### Remarks.

The name “*brachyptychia*” as mentioned in Wenz (1929: 2682) is an incorrect subsequent spelling.

#### Melanopsis brasiliensis

Taxon classificationAnimaliaSorbeoconchaMelanopsidae

Moricand, 1838

##### Original source.

Moricand 1838: 144, pl. 3, figs 12–13.

##### Type locality.

“Près de Villa de Barra” [near Villa de Barra, province Bahia], Brazil.

##### Remarks.

Currently considered to belong in the genus *Hemisinus* Swainson, 1840 (Thiaridae) (Nuttall 1990: 244).

#### † Melanopsis braueri

Taxon classificationAnimaliaSorbeoconchaMelanopsidae

Neumayr in Neumayr & Paul, 1875

##### Original source.

Neumayr and Paul 1875: 43, pl. 8, figs 26–27.

##### Type horizon.

Cernikian, Pliocene.

##### Type locality.

“Graben hinter der Kirche von Podwin; [...] Graben zwischen der Čapla und Podwin; [...] Čapla; [...] Gromačnik; [...] Strassengraben zwischen Gromačnik und Sibin; [...] Cigelnik” [ditch behind the church of Podvinje; Čaplja trench near Slavonski Brod; Čaplja; Gromačnik; roadside ditch between Gromačnik and Sibinj; Ciglenik], Croatia.

#### † Melanopsis pterochila var. breastensis

Taxon classificationAnimaliaSorbeoconchaMelanopsidae

Stefanescu, 1896

##### Original source.

Stefanescu 1896: 130, pl. 11, figs 26, 28, 30–31.

##### Type horizon.

Early Cernikian, early Pliocene.

##### Type locality.

“À Breasta, à Bocovatz, à Glodu et à Bâzdâna, dans la vallée de Jiu, et à Beceni, dans la vallée de Slanic, district de Buzau” [at Breasta, at Bucovăț, at Glodu and at Bâzdâna, in the valley of the river Jiu, and at Beceni, in the valley of the river Slănic, Buzau county], Romania.

#### Melanopsis brevicula

Taxon classificationAnimaliaSorbeoconchaMelanopsidae

Pallary, 1918

##### Original source.

Pallary 1918: 150.

##### Type locality.

“Agouraï, Aïn Mahrouf” [Agourai, Oued Ain Maarouf], Morocco.

#### † Melanopsis brevis

Taxon classificationAnimaliaSorbeoconchaMelanopsidae

Sowerby, 1826

##### Original source.

Sowerby 1826–1829: 42, pl. 523, fig. 2.

##### Type horizon.

Eocene.

##### Type locality.

“Upon the Hampshire coast”, United Kingdom.

##### Remarks.

After Cossmann (1909: 153) this species belongs in the genus *Coptostylus* Sandberger, 1872 (Thiaridae).

#### Melanopsis brevis

Taxon classificationAnimaliaSorbeoconchaMelanopsidae

Parreyss in Mousson, 1854 [invalid]

##### Original source.

Mousson 1854: 51.

##### Type locality.

“Dans les eaux de l’ancien Léonthes” [in the Litani river], Lebanon.

##### Remarks.

Junior homonym of *Melanopsis brevis* Sowerby, 1826. Blanckenhorn (1897) introduced *Melanopsis maroccana media* as replacement name (see also *Melanopsis moussoni* Pallary, 1916). The authority is clear from the discussion provided in Mousson (1854); in fact, Mousson even seemed to doubt the specific separation of this taxon from other melanopsids.

#### Melanopsis brevis

Taxon classificationAnimaliaSorbeoconchaMelanopsidae

Morelet, 1857 [invalid]

##### Original source.

Morelet 1857: 32.

##### Type locality.

“[Ad Sanctam-Mariam de Balade]” [Balade], New Caledonia.

##### Remarks.

Junior homonym of *Melanopsis brevis* Sowerby, 1826. Pallary (1916: 81) introduced *Melanopsis moreleti* as replacement name.

#### † Melanopsis (?Macrospira) brevis

Taxon classificationAnimaliaSorbeoconchaMelanopsidae

Doncieux, 1908 [invalid]

##### Original source.

Doncieux 1908: 205, pl. 11, figs 13a–b.

##### Type horizon.

Middle Lutetian, Eocene.

##### Type locality.

“Au Nord d’Albas”, France.

##### Remarks.

Junior homonym of *Melanopsis brevis* Sowerby, 1826. Pallary (1916: 81) introduced *Melanopsis abbreviata* as replacement name, which is itself a junior homonym of *Melanopsis abbreviata* Brusina, 1874 (see *Melanopsis doncieuxi* Wenz, 1919 and *Melanopsis atacica* Wenz, 1928).

#### Melanopsis graellsii var. brevis

Taxon classificationAnimaliaSorbeoconchaMelanopsidae

Pallary, 1924 [invalid]

##### Original source.

Pallary 1924: 250.

##### Type locality.

“Navajas (Castellón), Alcudia, Alberrique, Jativa (Valencia)” [Navajas, L’Alcúdia, Alberic, Xàtiva (prov. Valencia)], Spain.

##### Remarks.

Junior homonym of *Melanopsis brevis* Sowerby, 1826. Not available from Rossmässler (1854), to whom Pallary referred to, because he did not use the term “*brevis*” to denote a species-group taxon but only cited Férussac’s (1823) description of an unnamed variety of *Melanopsis dufourii*.

#### † Melanopsis (Melanosteira) aetolica brevisesta

Taxon classificationAnimaliaSorbeoconchaMelanopsidae

[sic] Papp & Thenius, 1952

##### Original source.

Papp and Thenius 1952: 4.

##### Type horizon.

Gelasian, early Pleistocene.

##### Type locality.

Not stated in Papp and Thenius (1952), but specified by Papp (1955) as “Angelocastron, (Agrinion)” [Angelókastro], Greece.

##### Types.

Museum of Palaeontology and Geology of the University of Athens; no number indicated (Papp 1955).

##### Remarks.

Although probably unintentionally, Papp and Thenius (1952) briefly described and thus validly introduced the taxon. The spelling “*brevisesta*” is probably a lapsus calami for “*brevitesta*” (Latin for “short shell”), as used in the re-description by Papp (1955: 126, pl. 20, figs 10–14). Nevertheless, “*brevisesta*” is the correct name following Art. 32.5.1 and “*brevitesta*” is an incorrect subsequent spelling.

#### Melanopsis jordanica var. breviuscula

Taxon classificationAnimaliaSorbeoconchaMelanopsidae

Bourguignat, 1884

##### Original source.

Bourguignat 1884: 142.

##### Type locality.

“Lac de Tibériade” [Sea of Galilee], Israel.

#### † Melanopsis briarti

Taxon classificationAnimaliaSorbeoconchaMelanopsidae

Cossmann, 1888

##### Original source.

Cossmann 1888: 282.

##### Type horizon.

Late Danian, Paleocene.

##### Type locality.

“Mons” (Briart and Cornet 1873: 71), Belgium.

##### Remarks.

Introduced for *Melania buccinoidea* sensu Briart & Cornet, 1873, “non Férussac” (actually it should read “non Olivier, 1801”).

#### † Melanopsis briarti

Taxon classificationAnimaliaSorbeoconchaMelanopsidae

Munier-Chalmas, 1897 [invalid]

##### Original source.

Munier-Chalmas 1897: 86.

##### Type horizon.

Late Danian, Paleocene.

##### Type locality.

“Mons” (Briart and Cornet 1873: 71), Belgium.

##### Remarks.

Introduced for *Melania buccinoidea* sensu Briart & Cornet, 1873, “non Férussac” (actually it should read “non Olivier, 1801”). Junior homonym, as well as junior objective synonym of *Melanopsis briarti* Cossmann, 1888. Obviously, Munier-Chalmas overlooked that the name had already been introduced for the very same misidentified taxon by Cossmann.

#### † Melania

Taxon classificationAnimaliaSorbeoconchaMelanopsidae

Brocchii Michelotti, 1847

##### Original source.

Michelotti 1847: 189.

##### Type horizon.

Late Miocene.

##### Type locality.

“Près de Tortone” [near Tortona], Italy.

##### Remarks.

Wenz (1929: 2879) considered it a junior synonym of *Ptychomelania buccinella* Sacco, 1895 (Thiaridae).

#### † Melanopsis brongniarti

Taxon classificationAnimaliaSorbeoconchaMelanopsidae

Locard, 1883

##### Original source.

Locard 1883b: 99.

##### Type horizon.

Mammal zone MN 15, Pliocene.

##### Type locality.

“Montgardon”, France.

#### † Melanopsis broti

Taxon classificationAnimaliaSorbeoconchaMelanopsidae

Neumayr, 1880

##### Original source.

Neumayr 1880b: 295, pl. 1, fig. 29.

##### Type horizon.

Plio-Pleistocene.

##### Type locality.

“Zwischen Pylle und Antimachia” [between Pýli and Antimácheia, Kos Island], Greece.

##### Remarks.

Pallary (1925) and Wenz (1929) erroneously referred to it as homonym of “*Melanopsis broti*” Gassies, 1874, which was actually introduced as *Melanopsis brotiana*. The replacement name *Melanopsis cosiana* Pallary, 1925 is thus invalid as it is a junior objective synonym (see also Willmann 1981).

#### Melanopsis brotiana

Taxon classificationAnimaliaSorbeoconchaMelanopsidae

Gassies, 1874

##### Original source.

Gassies 1874: 386.

##### Type locality.

“La Conception, prope Noumea” [Conception, near Nouméa], New Caledonia.

#### † ? Melanopsis brownii

Taxon classificationAnimaliaSorbeoconchaMelanopsidae

Etheridge, 1879

##### Original source.

Etheridge 1879: 87, pl. 7, fig. 4.

##### Type horizon.

Pebasian, late Cenozoic.

##### Type locality.

“Canama”, Peru.

##### Remarks.

Currently considered to belong in the genus *Verena* H. Adams & A. Adams, 1854 (Thiaridae) (Nuttall 1990: 256). The name “*browni*” as given by Nuttall (1990) is an incorrect subsequent spelling.

#### † Melanopsis brusinai

Taxon classificationAnimaliaSorbeoconchaMelanopsidae

Lörenthey, 1902

##### Original source.

Lörenthey 1902: 223, pl. 16, fig. 7.

##### Type horizon.

Middle Pannonian, late Miocene.

##### Type locality.

“Tinnye”, Hungary.

#### † Melanoptychia brusinai

Taxon classificationAnimaliaSorbeoconchaMelanopsidae

Jekelius, 1944 [invalid]

##### Original source.

Jekelius 1944: 137, pl. 56, figs 1–23.

##### Type horizon.

Early Pannonian, late Miocene.

##### Type locality.

“Turislav-Tal bei Soceni” [Turislav valley near Soceni], Romania.

##### Remarks.

Junior secondary homonym and junior synonym of *Melanopsis brusinai* Lörenthey, 1902 (see Neubauer et al. 2014a: 462).

#### † Melanopsis (Homalia) bucciniformis

Taxon classificationAnimaliaSorbeoconchaMelanopsidae

Handmann, 1887

##### Original source.

Handmann 1887: 14, pl. 1, figs 8–9.

##### Type horizon.

Pannonian, zone B–D, late Miocene.

##### Type locality.

“Leobersdorf”, Austria.

##### Remarks.

Wenz (1929: 2813) considered the taxon as a junior synonym of *Melanopsis pygmaea* Hörnes, 1856.

#### Melania buccinoidea

Taxon classificationAnimaliaSorbeoconchaMelanopsidae

Olivier, 1801

##### Original source.

Olivier 1801: 297, pl. 17, fig. 8.

##### Type locality.

“De Scio, de presque toutes les îles de l’Archipel, de Crète, de Syrie” [from Chios, from almost all islands of the Archipelago, from Crete (Greece), from Syria].

##### Remarks.

Today combined as *Melanopsis buccinoidea*, the species is one of the few stable taxa in melanopsid taxonomy (e.g., Glaubrecht 1996).

#### † Melanopsis buccinulum

Taxon classificationAnimaliaSorbeoconchaMelanopsidae

Melleville, 1843

##### Original source.

Melleville 1843: 95, pl. 4, figs 13–15.

##### Type horizon.

Thanetian, Paleocene.

##### Type locality.

“Châlons” [Châlons-sur-Vesle], France.

##### Remarks.

Perhaps unaware of Melleville’s publication, Deshayes (1862: 469, pl. 31, figs 11–13) erroneously described this species as new: he marked the name with “Desh.”, provided a Latin diagnosis and did not list any citations. Judging from the illustrations in both works, Deshayes’ species is undoubtedly the same as Melleville’s. Moreover, both were recorded from the same locality (Châlons-sur-Vesles).

#### Melanopsis tingitana var. bucheti

Taxon classificationAnimaliaSorbeoconchaMelanopsidae

Pallary, 1899

##### Original source.

Pallary 1899: 147, pl. 9, fig. 6.

##### Type locality.

“L’O.[ued] Ida ou Guert, près de Mogador” [Wadi Ida Ou Gourdh at Essaouira], Morocco.

#### † Melanopsis bukovaci

Taxon classificationAnimaliaSorbeoconchaMelanopsidae

Brusina, 1902

##### Original source.

Brusina 1902: pl. 6, figs 8–10.

##### Type horizon.

Middle–late Cernikian, late Pliocene–early Pleistocene.

##### Type locality.

“Gromačnik”, Croatia.

##### Types.

The illustrated syntypes are stored in the Croatian Natural History Museum, Zagreb, coll. no. 2506-152/1-3 (Milan et al. 1974: 87).

#### Bithynia bulgarica

Taxon classificationAnimaliaSorbeoconchaMelanopsidae

Drensky, 1947

##### Original source.

Drensky 1947: 45.

##### Type locality.

“Познат за сега само от р. Дунав, северно от гр. Лом” [from the river Danube, near the city of Lom], Bulgaria.

##### Remarks.

Angelov (2000: 15), while incorrectly treating the name as nomen nudum, considered the species as a junior synonym of *Amphimelania holandri* [sic] (Pfeiffer, 1828).

#### Melanopsis bullio

Taxon classificationAnimaliaSorbeoconchaMelanopsidae

Kobelt, 1879

##### Original source.

Kobelt 1879–1880: 17, pl. 188, figs 1902–1903.

##### Type locality.

Not indicated, probably in the Middle East.

##### Remarks.

Introduced in synonymy of *Melania costata* by Kobelt (1879), referring to an “in schedis” name by Parreyss. Dautzenberg (1894) made the name available by treating it as valid name (see Note 2).

#### † Melanopsis calamonensis

Taxon classificationAnimaliaSorbeoconchaMelanopsidae

Magrograssi, 1928

##### Original source.

Magrograssi 1928: 261, pl. 6, fig. 22.

##### Type horizon.

Plio-Pleistocene.

##### Type locality.

“Rodi: colline sulla sinistra del fiume Dimilia” [Rhodes island: hills on the left bank of the river Dimilia], Greece.

#### Melanopsis callichroa

Taxon classificationAnimaliaSorbeoconchaMelanopsidae

Bourguignat, 1884

##### Original source.

Bourguignat 1884: 91.

##### Type locality.

“Dans l’intérieur de la grotte du Nahr-el-Kelb, près de Beyrouth (Syrie)” [in the cave of Nahr el-Kalb near Beirut], Lebanon.

#### Melanopsis callista

Taxon classificationAnimaliaSorbeoconchaMelanopsidae

Bourguignat, 1884

##### Original source.

Bourguignat 1884: 118.

##### Type locality.

“Sadjour-Sou entre Ain-Taïb et Alep” [at Sadjour-Sou between Gaziantep (Turkey) and Aleppo (Syria)].

##### Remarks.

Heller et al. (2005: 243) tentatively considered the species as a junior synonym of *Melanopsis costata* (Olivier, 1804).

#### † Melanopsis callosa

Taxon classificationAnimaliaSorbeoconchaMelanopsidae

Voltz, 1852

##### Original source.

Voltz 1852: viii, pl. 3, fig. 10.

##### Type horizon.

Mammal zone MN 1, early Miocene.

##### Type locality.

“Weisenau”, Germany.

##### Remarks.

The name appeared first as nomen nudum in Genth (1848: 198–199), Sandberger (1850: 18; 1851: 676; 1852: 684) and Braun in Walchner (1851: 1126). Wenz (1929: 2764) considered this taxon as a junior synonym of *Melanopsis fritzei* Thomä, 1845.

#### † Melanopsis camptogramma

Taxon classificationAnimaliaSorbeoconchaMelanopsidae

Brusina, 1876

##### Original source.

Brusina 1876: 109.

##### Type horizon.

Early Langhian, middle Miocene.

##### Type locality.

Originally given as “Sinj” and later specified as “Župića potok” in Brusina (1884b), Croatia.

##### Types.

Milan et al. (1974: 87) stated that Brusina (1897) had indicated one of the specimens illustrated by him (pl. 5, figs 1–2) as type. However, it is uncertain whether the specimen actually derives from the original type series. The specimen is stored in the Croatian Natural History Museum, Zagreb, coll. no. 2980-626/1.

#### Microcolpia canaliculata

Taxon classificationAnimaliaSorbeoconchaMelanopsidae

Bourguignat, 1884

##### Original source.

Bourguignat 1884: 62.

##### Type locality.

“Le Danube à Ibraïla” [Danube river at Brăila], Romania.

#### † Melanopsis capelliniana

Taxon classificationAnimaliaSorbeoconchaMelanopsidae

Pallary, 1920

##### Original source.

Pallary 1920b: 115.

##### Type horizon.

Messinian, late Miocene.

##### Type locality.

“Colognole e al casino Cubbe; [...] presso il casino Sant’Andrea sotto Colognole” (Capellini 1880: 397) [at Colognole and at Casa Cubbe; near Casa Sant’Andrea below Colognole”, Italy.

##### Remarks.

Introduced for “*Melanopsis dufourii* var. a” in Capellini, 1880. Pallary used multiple original spellings (“*capelliniana*”, “*capelliana*”) but according to 32.5.1 the name must be *capelliniana*. Wenz (1929: 2785) considered Capellini’s material synonymous with *Melanopsis narzolina* Manzoni, 1870.

#### † Melanopsis harpula var. capreniensis

Taxon classificationAnimaliaSorbeoconchaMelanopsidae

Fontannes, 1887

##### Original source.

Fontannes 1887: 335.

##### Type horizon.

Early Cernikian, early Pliocene.

##### Type locality.

“Capreni, val. Amaradii (Jud. Gorjiu)” [Căpreni], Romania.

#### † Melanopsis (Martinia) vindobonensis var. capuliformis

Taxon classificationAnimaliaSorbeoconchaMelanopsidae

Handmann, 1887

##### Original source.

Handmann 1887: 29, pl. 6, figs 5–6.

##### Type horizon.

Pannonian, zone B–D, late Miocene.

##### Type locality.

“Leobersdorf”, Austria.

#### † Melanopsis capulus

Taxon classificationAnimaliaSorbeoconchaMelanopsidae

Handmann, 1882

##### Original source.

Handmann 1882: 554.

##### Type horizon.

Pannonian, zone D, late Miocene.

##### Type locality.

“Kottingbrunn [...] Ziegelei a”, Austria.

##### Remarks.

The species epithet is a noun in apposition and needs not to agree in gender with the generic name (Art. 31.2.1). Wenz (1929: 2718) considered this taxon as a junior synonym of *Melanopsis fossilis* (Gmelin, 1791).

#### † Melanopsis caputinensis

Taxon classificationAnimaliaSorbeoconchaMelanopsidae

Stefanescu, 1897

##### Original source.

Stefanescu 1897: 311, pl. 8, figs 8–10.

##### Type horizon.

Pliocene.

##### Type locality.

“Au ravin de Tura, à Capatzineni; [...] aussi à Salatrucu-Mare” [in the ravine of Tura in Căpățânenii; also at Sălătrucu], Romania.

#### † Melanopsis carasiensis

Taxon classificationAnimaliaSorbeoconchaMelanopsidae

Jekelius, 1944

##### Original source.

Jekelius 1944: 131, pl. 49, figs 7–10.

##### Type horizon.

Early Pannonian, late Miocene.

##### Type locality.

“Turislav-Tal bei Soceni” [Turislav valley near Soceni], Romania.

#### † Melanopsis (Melanoptychia) cari

Taxon classificationAnimaliaSorbeoconchaMelanopsidae

Pavlović, 1927

##### Original source.

Pavlović 1927: 61, pl. 7, figs 3–4.

##### Type horizon.

Pannonian, zone D–E, late Miocene.

##### Type locality.

“Из Рамаће” [from the village Ramača (near Ripanj)], Serbia.

##### Types.

The illustrated syntype is stored in the Natural History Museum, Belgrade, coll. no. 796 (Milošević 1962: 23).

#### † Melanopsis carinata

Taxon classificationAnimaliaSorbeoconchaMelanopsidae

Sowerby, 1826

##### Original source.

Sowerby 1826–1829: 41, pl. 523, fig. 1.

##### Type horizon.

Eocene.

##### Type locality.

“In a well near Newport, Isle of Wight; [...] from Hampstead Cliff to Cowes, and [...] on the opposite Cliffs of Hampshire”, United Kingdom.

#### Melanopsis carinata

Taxon classificationAnimaliaSorbeoconchaMelanopsidae

Gassies, 1861 [invalid]

##### Original source.

Gassies 1861: 289, pl. 7, fig. 13.

##### Type locality.

“Dans le Diahot, à Balade; à Jengen, à Kanala, dans les marais et les petits ruisseaux” [in the Diahot river at Balade; at Hienghène, at Canala, in swamps and small streams], New Caledonia.

##### Remarks.

Junior homonym of *Melanopsis carinata* Sowerby, 1826. Pallary (1916: 82) introduced *Melanopsis ducosi* as replacement name.

#### Melanopsis mingrelica var. carinata

Taxon classificationAnimaliaSorbeoconchaMelanopsidae

Issel, 1865 [invalid]

##### Original source.

Issel 1865 [page unknown; original work not seen; described on p. 400 of the re-published version from 1866].

##### Type locality.

“Nel lago di Paleaston presso Poti” [Paliastomi Lake near Poti], Georgia.

##### Remarks.

Junior homonym of *Melanopsis carinata* Sowerby, 1826.

#### Melanopsis dufourii var. carinata

Taxon classificationAnimaliaSorbeoconchaMelanopsidae

Gentiluomo, 1868 [invalid]

##### Original source.

Gentiluomo 1868: 97, pl. 6, figs 10–11.

##### Type locality.

“Lago dell’Accesa, Toscana”, Italy.

##### Remarks.

Junior homonym of *Melanopsis carinata* Sowerby, 1826.

#### † Melanopsis hazayi var. carinata

Taxon classificationAnimaliaSorbeoconchaMelanopsidae

Brusina, 1903 [invalid]

##### Original source.

Brusina 1903: 113.

##### Type horizon.

Late Pleistocene–early Holocene.

##### Type locality.

“Bischofsbad” [Püspökfürdő, Băile 1 Mai, Lake Pețea], Romania.

##### Remarks.

Junior homonym of *Melanopsis carinata* Sowerby, 1826. Neubauer et al. (2014d: 125) considered this taxon as a junior synonym of *Microcolpia parreyssii sikorai* (Brusina, 1903).

#### † Melanopsis sikorai var. carinata

Taxon classificationAnimaliaSorbeoconchaMelanopsidae

Kormos, 1903 [invalid]

##### Original source.

Kormos 1903: 456, 501, pl. 13, fig. 1.

##### Type horizon.

Late Pleistocene–early Holocene.

##### Type locality.

“Püspökfürdő” [Băile 1 Mai, Lake Pețea], Romania.

##### Remarks.

Obviously unaware of the fact that variety names are available in nomenclature as species-group names, Kormos (1903) stated that, if raised to species, he suggests “*Melanopsis mucronifera*” as name for the taxon. Both names were simultaneously published and are junior objective synonyms. Since *carinata* sensu Kormos is a junior homonym of *Melanopsis carinata* Sowerby, 1826, *Melanopsis mucronifera* is the valid name of the taxon.

#### † Melanopsis staubi var. carinata

Taxon classificationAnimaliaSorbeoconchaMelanopsidae

Brusina, 1903 [invalid]

##### Original source.

Brusina 1903: 115.

##### Type horizon.

Late Pleistocene–early Holocene.

##### Type locality.

“Bischofsbad” [Püspökfürdő, Băile 1 Mai, Lake Pețea], Romania.

##### Remarks.

Junior objective synonym of *Melanopsis staubi*: Brusina (1903) indicated it as the typical form of the species. Junior homonym of *Melanopsis carinata* Sowerby, 1826. Neubauer et al. (2014d: 125) considered this taxon as a junior synonym of *Microcolpia parreyssii sikorai* (Brusina, 1903).

#### Melanopsis doriae var. carinata

Taxon classificationAnimaliaSorbeoconchaMelanopsidae

Biggs, 1937 [invalid]

##### Original source.

Biggs 1937: 248.

##### Type locality.

“From Jelalabad” [Jalalabad], Iran.

##### Remarks.

Junior homonym of *Melanopsis carinata* Sowerby, 1826.

#### † Melanopsis impressa var. carinatissima

Taxon classificationAnimaliaSorbeoconchaMelanopsidae

Sacco, 1889

##### Original source.

Sacco 1889: 65, pl. 2, figs 24–25.

##### Type horizon.

Tortonian–early Messinian, late Miocene.

##### Type locality.

“Delle colline tortonesi presso S. Agata” [from the Torino hills near Sant’Agata Fossili], Italy.

#### † Melanopsis aetolica var. carinatocostata

Taxon classificationAnimaliaSorbeoconchaMelanopsidae

Oppenheim, 1891

##### Original source.

Oppenheim 1891: 468, pl. 27, figs 1–2.

##### Type horizon.

Gelasian, early Pleistocene.

##### Type locality.

“Stamna”, Greece.

##### Remarks.

Not available from Oppenheim (1890b), where he gave it as “mutation”, which is not ruled by the provisions of the Code. In 1891 he introduced the name as “*carinato-costata*”.

#### Melanopsis praerosa [sic] var. carinifera

Taxon classificationAnimaliaSorbeoconchaMelanopsidae

Paetel, 1888

##### Original source.

Paetel 1888: 403.

##### Type locality.

“Kerman” (Nevill 1884: 209), Iran.

##### Remarks.

Originally introduced as infrasubspecific taxon (“subvariety”) by Nevill (1884), but made available by Paetel (1888) who treated it as variety (Art. 45.5.1). Paetel clearly referred to the description of Nevill.

#### Murex cariosus

Taxon classificationAnimaliaSorbeoconchaMelanopsidae

Linnaeus, 1767

##### Original source.

Linnaeus 1767: 1220.

##### Type locality.

“In Aqaeductu ad Sevillam” [in an aqueduct near Sevilla], Spain.

##### Remarks.

Today combined as *Melanopsis cariosa*, the species is one of the few stable taxa in melanopsid taxonomy (e.g., Glaubrecht 1996).

#### † Melanoptychia carusi

Taxon classificationAnimaliaSorbeoconchaMelanopsidae

Brusina, 1902

##### Original source.

Brusina 1902: pl. 30, figs 6–7.

##### Type horizon.

Langhian, middle Miocene.

##### Type locality.

“Džepe” [Džepi], Bosnia and Herzegovina.

##### Types.

The original type material of Brusina (1902) is lost. Neubauer et al. (2016c: 275–276) defined a neotype from the type locality. The specimen is stored in the Geological Survey Austria, Vienna, coll. no. 2014/010/0002.

#### † Lyrcaea [sic] caryota

Taxon classificationAnimaliaSorbeoconchaMelanopsidae

Brusina, 1902

##### Original source.

Brusina 1902: pl. 5, figs 21–25.

##### Type horizon.

Transdanubian, Pannonian, late Miocene.

##### Type locality.

“Kenese” [Balatonkenese], Hungary.

##### Types.

The illustrated syntypes are stored in the Croatian Natural History Museum, Zagreb, coll. no. 2485-131/1-5 (Milan et al. 1974: 83).

#### “ Melanopsis castanea

Taxon classificationAnimaliaSorbeoconchaMelanopsidae

” mentioned in Férussac (1814: 54) [unavailable]

##### Locality.

Not indicated.

##### Remarks.

Nomen nudum. Férussac (1823: 149) listed it in synonymy of *Melania buccinoidea* var. α [= *Melania buccinoidea* (Olivier, 1801)].

#### Melanella castanea

Taxon classificationAnimaliaSorbeoconchaMelanopsidae

Bourguignat, 1884

##### Original source.

Bourguignat 1884: 27.

##### Type locality.

“Rivière à Ostaria et à Ogulin (Croatie)” [river at Oštarije and at Ogulin], Croatia.

##### Remarks.

Note that Bourguignat denoted the authority as “Bourguignat, 1879”.

#### † Melanopsis castrensis

Taxon classificationAnimaliaSorbeoconchaMelanopsidae

Noulet, 1854

##### Original source.

Noulet 1854: 50.

##### Type horizon.

Bartonian, Eocene.

##### Type locality.

“À Labruguière; [...] à Augmontel (Tarn)”, France.

#### Melanella coronata var. catoleia

Taxon classificationAnimaliaSorbeoconchaMelanopsidae

Bourguignat, 1884

##### Original source.

Bourguignat 1884: 12.

##### Type locality.

“De la Savina” [from the Savinja river], Slovenia.

#### Melanopsis ceardi

Taxon classificationAnimaliaSorbeoconchaMelanopsidae

Pallary, 1928

##### Original source.

Pallary 1928a: 268, pl. 6, figs 4–5.

##### Type locality.

“Ouakda, à 4 kil. N.-E. de Colomb-Béchar” [Ouakda, 4 km northeast of Bechar], Algeria.

#### † Melanopsis cepula

Taxon classificationAnimaliaSorbeoconchaMelanopsidae

Guppy, 1866

##### Original source.

Guppy 1866: 580, pl. 26, fig. 14.

##### Type horizon.

Late Miocene.

##### Type locality.

“Cumana”, Venezuela.

##### Remarks.

Given as “*capula*” on p. 580, but as “*cepula*” in the plate captions. Guppy (1867) acted as First Reviser and fixed the correct original spelling as “*cepula*” (Art. 24.2.3, 32.2.1). Guppy (1867) did not consider the species a melanopsid anymore and introduced the new genus *Crepitacella* (Rissoinidae) with *Melanopsis cepula* as type species.

#### † Melanopsis cerithiformis

Taxon classificationAnimaliaSorbeoconchaMelanopsidae

Watelet, 1851

##### Original source.

Watelet 1851: 121, pl. 1, figs 1–2.

##### Type horizon.

Cuisian, late Ypresian, Eocene.

##### Type locality.

“Mercin” [Mercin-et-Vaux], France.

##### Remarks.

Cossmann (1888: 279) classified the species in the genus *Faunus* Montfort, 1810 (Pachychilidae). Wenz (1929: 2637) apparently agreed with that classification but listed it a second time (p. 2563) in synonymy of *Semisinus* [= *Hemisinus*] *resectus* (Deshayes, 1834).

#### Melanopsis cerithiopsis

Taxon classificationAnimaliaSorbeoconchaMelanopsidae

Bourguignat, 1884

##### Original source.

Bourguignat 1884: 130.

##### Type locality.

“Plaine du Bahr-el-Houlé (haut Jourdain) dans l’Aïn-el-Mellaha” [in the plains of the Hula valley (upper Jordan), in Aïn Mallahah], Israel.

##### Remarks.

Given as “*cerithiopis*” in heading of description, but as “*cerithiopsis*” throughout the rest of the work. Heller et al. (2005: 248) considered the species as a junior synonym of *Melanopsis saulcyi* Bourguignat, 1853.

#### Melanopsis cerullii

Taxon classificationAnimaliaSorbeoconchaMelanopsidae

Pallary, 1920

##### Original source.

Pallary 1920b: 110.

##### Type locality.

“M. Mario: Farnesina” (Cerulli-Irelli 1914: 186), Italy.

##### Remarks.

Replacement name for *Melanopsis transiens* Cerulli-Irelli, 1914, non Blanckenhorn, 1897. Girotti (1972: 232) considered *Melanopsis transiens* Cerulli-Irelli, 1914 a junior synonym of “*Melanopsis affinis* Férussac”, which is not an available name.

#### Melanopsis cesari

Taxon classificationAnimaliaSorbeoconchaMelanopsidae

Pallary, 1920

##### Original source.

Pallary 1920a: 30.

##### Type locality.

“Beni Abbès” [prov. Béchar], Algeria.

#### † Melanopsis clavigera f. cesticillus

Taxon classificationAnimaliaSorbeoconchaMelanopsidae

Brusina, 1897

##### Original source.

Brusina 1897: 7, pl. 5, fig. 20.

##### Type horizon.

Cernikian, Pliocene.

##### Type locality.

“Kozarica” [Kozarice], Croatia.

##### Types.

Milan et al. (1974: 88) indicated a holotype, but it is uncertain whether the specimen was the only one Brusina had at hand (holotype by monotypy, Art. 73.1.2). The specimen is stored in the Croatian Natural History Museum, Zagreb, coll. no. 2991-637.

##### Remarks.

Mandic et al. (2015: 194) considered this taxon as a junior synonym of *Melanopsis clavigera* Neumayr in Neumayr & Paul, 1875.

#### Melanopsis chantrei

Taxon classificationAnimaliaSorbeoconchaMelanopsidae

Locard, 1883

##### Original source.

Locard 1883a: 268, pl. 23, figs 44–49.

##### Type locality.

“Lac d’Antioche” [Lake Anuk (also as Amik)], Turkey.

#### Melanopsis charpentieri

Taxon classificationAnimaliaSorbeoconchaMelanopsidae

Brot, 1879

##### Original source.

Brot 1874–1879: 430, pl. 46, fig. 8.

##### Type locality.

“Schiraz”, Iran.

##### Remarks.

Brot (1879) atrributed the name to Parreyss, based on his “in schedis” determination.

#### Melanopsis chehirensis

Taxon classificationAnimaliaSorbeoconchaMelanopsidae

Pallary, 1939

##### Original source.

Pallary 1939: 94, pl. 6, figs 51–58, 75.

##### Type locality.

“Dans le source de Yeni Chehir, [...] entre Antioche et Alep, à l’intersection de la route d’Alexandrette” [at the source of Yenişehir, between Antakya and Aleppo, at the intersection of the road from İskenderun], Turkey.

#### Melanopsis (Mesopotamia) cheragragensis

Taxon classificationAnimaliaSorbeoconchaMelanopsidae

Pallary, 1939

##### Original source.

Pallary 1939: 104, pl. 5, figs 8–9, 11.

##### Type locality.

“Cheragrag, en Mésopotamie, entre Rakka, au Sud et Tell Abiad au Nord, sur la rive gauche du Karamouk Sou, affluent de la rive droite du Nahr Bâhlik” [Sharakrak in Mesopotamia, beteween Ar Raqqah in the south and Tall Abyaḑ in the north, on the left bank of the Qarah Mūkh, tributary of the right side of the Nahr al Balīkh], Syria.

##### Remarks.

Heller et al. (2005: 254) considered the species as a junior synonym of *Melanopsis infracincta* Martens, 1874.

#### Melanopsis buccinoidea var. chlorotica

Taxon classificationAnimaliaSorbeoconchaMelanopsidae

Pallary, 1913

##### Original source.

Pallary 1913: 364.

##### Type locality.

“L’Abreuvoir de Dar-Beida” [water trough of Dar Beïda], southern Morocco.

#### Melanopsis chobauti

Taxon classificationAnimaliaSorbeoconchaMelanopsidae

Nicolas, 1898

##### Original source.

Nicolas 1898: 530.

##### Type locality.

“Aux environs de Biskra, à Ain-Oumach [...] dans une source d’eau chaude” [around Biskra, at Ain Oumache; in a hot spring], Algeria.

##### Remarks.

Pallary (1912: 14) considered the taxon as a junior synonym of *Melanopsis saharica* Bourguignat, 1864.

#### † Melanopsis choctavensis

Taxon classificationAnimaliaSorbeoconchaMelanopsidae

Aldrich, 1886

##### Original source.

Aldrich 1886: 35, pl. 3, fig. 8.

##### Type horizon.

Early Eocene.

##### Type locality.

“Hatchetigbee, Butler, Choctaw County, Alabama”, United States.

##### Types.

Palmer and Brann (1966: 546) stated the the figured syntype was missing. A remaining syntype is stored in the collection of the National Museum of Natural History, Smithsonian Institution, Washington, DC, coll. no. 638787.

##### Remarks.

Classified within the marine genus *Bulliopsis* Conrad, 1862 (Nassariidae) by Harris (1899: 58) and Allmon (1990: 53).

#### † Melanopsis cikosi

Taxon classificationAnimaliaSorbeoconchaMelanopsidae

Brusina, 1902

##### Original source.

Brusina 1902: pl. 6, fig. 5.

##### Type horizon.

Early Cernikian, early Pliocene.

##### Type locality.

“Čerević”, Serbia.

##### Types.

Milan et al. (1974: 88) indicated a holotype, but it is uncertain whether the specimen was the only one Brusina had at hand (holotype by monotypy, Art. 73.1.2). The specimen is stored in the Croatian Natural History Museum, Zagreb, coll. no. 2504-150.

#### † Melanopsis cincta

Taxon classificationAnimaliaSorbeoconchaMelanopsidae

Neumayr, 1880 

##### Original source.

Neumayr 1880b: 290, pl. 1, fig. 10.

##### Type horizon.

Plio-Pleistocene.

##### Type locality.

“Zwischen Pylle und Antimachia” [between Pýli and Antimácheia, Kos Island], Greece.

##### Remarks.

Willmann (1981: 179) considered this taxon as a junior synonym of *Melanopsis delessei* Tournouër, 1875.

#### † Melanopsis citharella

Taxon classificationAnimaliaSorbeoconchaMelanopsidae

Merian, 1849

##### Original source.

Merian 1849: 31.

##### Type horizon.

Burdigalian, early Miocene.

##### Type locality.

“Nahe am Gipfel des Plateaus des Randens” [near the top of the plateau of Mt. Randen], Switzerland.

#### † Melanopsis clava

Taxon classificationAnimaliaSorbeoconchaMelanopsidae

Sandberger, 1872

##### Original source.

Sandberger 1870–1875: pl. 25, fig. 31.

##### Type horizon.

Badenian, middle Miocene.

##### Type locality.

“Grund [...], Vöslau” (Hörnes 1856: 597), Austria.

##### Remarks.

Plate 25 of Sandberger’s monograph was issued in 1872, while the description on pp. 512, 521 appeared in 1875 (Woodward 1906). Introduced for *Melanopsis aquensis* sensu Hörnes, 1856, non Grateloup, 1838.

#### † Melanopsis clavigera

Taxon classificationAnimaliaSorbeoconchaMelanopsidae

Neumayr in Neumayr & Paul, 1875

##### Original source.

Neumayr and Paul 1875: 41, pl. 7, figs 13–14.

##### Type horizon.

Cernikian, Pliocene.

##### Type locality.

“Cigelnik; [...] Graben zwischen Podwin und der Čapla; [...] Čapla; [...] An der Strasse von Sibin nach Gromačnik; [...] Gromačnik” [Ciglenik; Čaplja trench near Slavonski Brod; Čaplja; at the road from Sibinj to Gromačnik; Gromačnik], Croatia.

#### † Melanopsis clementina

Taxon classificationAnimaliaSorbeoconchaMelanopsidae

Michelin, 1833

##### Original source.

Michelin 1833: pl. 29.

##### Type horizon.

Cretaceous?

##### Type locality.

“À Gérodot, département de l’Aube”, France.

##### Remarks.

Probably not a Melanopsidae.

#### † Melanopsis (Martinia) martiniana var. coaequata

Taxon classificationAnimaliaSorbeoconchaMelanopsidae

Handmann, 1887

##### Original source.

Handmann 1887: 25, pl. 4, figs 8–9.

##### Type horizon.

Pannonian, zone B–D, late Miocene.

##### Type locality.

“Leobersdorf”, Austria.

##### Types.

A lectotype was designated by Fischer (1996b: 20). It is stored in the collection of the Geological Survey Austria, Vienna; no number indicated.

#### Melanella codiella

Taxon classificationAnimaliaSorbeoconchaMelanopsidae

Servain, 1884

##### Original source.

Bourguignat 1884: 27.

##### Type locality.

“La Migliaska à Serajewo (Bosnie); Ostaria (Croatie)” [in the Miljacka river at Sarajevo (Bosnia and Herzegovina); Oštarije (Croatia)].

##### Remarks.

Appeared first as a nomen nudum in Servain (1884).

#### † Melanopsis cognata

Taxon classificationAnimaliaSorbeoconchaMelanopsidae

Brusina, 1878

##### Original source.

Brusina 1878: 349.

##### Type horizon.

Late Portaferrian, late Miocene–early Pliocene.

##### Type locality.

“Karlowitz [...] Görgetek in Syrmien” (Neumayr and Paul 1875: 49) [Karlovci; Grgeteg], Serbia.

##### Types.

Milan et al. (1974: 88) indicated a holotype, but it is uncertain whether the specimen was the only one Brusina had at hand (holotype by monotypy, Art. 73.1.2). The specimen is stored in the Croatian Natural History Museum, Zagreb, coll. no. 3001-647.

##### Remarks.

Introduced for “Melanopsis cf. visianiana” in Neumayr and Paul, 1875, non Brusina, 1874.

#### Melanopsis guiraoi var. communis

Taxon classificationAnimaliaSorbeoconchaMelanopsidae

Pallary, 1924

##### Original source.

Pallary 1924: 251, pl. 25, fig. 20.

##### Type locality.

“Dans les cours d’eau de la province de Murcie” [in rivers of the province Murcia], Spain.

#### † Melanopsis neumayri var. compacta

Taxon classificationAnimaliaSorbeoconchaMelanopsidae

Fontannes, 1880 [invalid]

##### Original source.

Fontannes 1879–1882: 174.

##### Type horizon.

Mammal zone MN 11, late Miocene.

##### Type locality.

“Les marnes à *Potamides Basteroti* de Visan (Vaucluse)” [in the marls with *Potamides basteroti* in Visan, Dép. Vaucluse], France.

##### Remarks.

Wenz (1929: 2793) considered the variety as a junior synonym *Melanopsis neumayri* Tournouër, 1874.

#### Melanopsis compacta

Taxon classificationAnimaliaSorbeoconchaMelanopsidae

Pallary, 1920 [invalid]

##### Original source.

Pallary 1920a: 31.

##### Type locality.

“Aït Taleb sur le Sefrou près d’el Menzel, avant l’oued Sebou” [Douar Ait Taleb at Sefrou near El Menzel, before the Sebou river], Morocco.

##### Remarks.

Junior homonym of *Melanopsis compacta* Fontannes, 1880.

#### † Lyrcaea narzolina var. compressoides

Taxon classificationAnimaliaSorbeoconchaMelanopsidae

Sacco, 1895

##### Original source.

Sacco 1895: 14, pl. 1, fig. 26.

##### Type horizon.

Tortonian–early Messinian, late Miocene.

##### Type locality.

“S. Agata fossili” [Sant’Agata Fossili], Italy.

#### † Melanopsis conemenosiana

Taxon classificationAnimaliaSorbeoconchaMelanopsidae

Oppenheim, 1891

##### Original source.

Oppenheim 1891: 469, pl. 27, figs 7–8.

##### Type horizon.

Late Pliocene–early Pleistocene.

##### Type locality.

“Preveza”, Greece.

##### Remarks.

Appeared first as a nomen nudum (as “*M. Conemenosi* Bttg. in litt.”) in Oppenheim (1890b).

#### † Melanopsis confusa

Taxon classificationAnimaliaSorbeoconchaMelanopsidae

Strausz, 1941

##### Original source.

Strausz 1941: 143.

##### Type horizon.

Transdanubian, Pannonian, late Miocene.

##### Type locality.

“Radmanest” (Fuchs 1870: 353) [Rădmănești], Romania.

##### Remarks.

Replacement name for *Melanopsis fuchsi* Brusina, 1884, non Handmann, 1882 and *Melanopsis hungarica* Pallary, 1916, non Kormos, 1904, which were in turn introduced for *Melania costata* sensu Fuchs, 1870, non Olivier, 1804 (see also Strausz 1942; Neubauer et al. 2014a).

#### Melanopsis praerosa var. conica

Taxon classificationAnimaliaSorbeoconchaMelanopsidae

Bronn, 1848 [invalid]

##### Original source.

Bronn 1848: 718.

##### Type horizon.

Not stated, probably fossil.

##### Type locality.

Not indicated.

##### Remarks.

Junior objective synonym of *Melanopsis antediluviana* (Poiret, 1801), which Bronn (1848) gave in synonymy.

#### † Pseudofagotia conica

Taxon classificationAnimaliaSorbeoconchaMelanopsidae

Anistratenko, 1993

##### Original source.

Anistratenko 1993: 74, textfig. 2.

##### Type horizon.

Duab Beds, middle to late Kimmerian, Pliocene.

##### Type locality.

“Окр. с. Мокви, Очамчирский р-н” [near the village Mok’vi, Ochamchirskiy rayon], Georgia.

##### Types.

Schmalhausen Institute of Zoology of National Academy of Sciences of Ukraine, Kiev; no number indicated.

#### † Pseudofagotia coniformis

Taxon classificationAnimaliaSorbeoconchaMelanopsidae

Anistratenko, 1993

##### Original source.

Anistratenko 1993: 74, textfig. 2.

##### Type horizon.

Duab Beds, middle to late Kimmerian, Pliocene.

##### Type locality.

“Окр. с. Мокви, Очамчирский р-н” [near the village Mok’vi, Ochamchirskiy rayon], Georgia.

##### Types.

Schmalhausen Institute of Zoology of National Academy of Sciences of Ukraine, Kiev; no number indicated.

#### † Melanopsis coniungens

Taxon classificationAnimaliaSorbeoconchaMelanopsidae

Sacco, 1886

##### Original source.

Sacco 1886: 450, pl. 1, fig. 9.

##### Type horizon.

Late Burdigalian, early Miocene.

##### Type locality.

“Collina di Torino” [Torino hills], Italy.

##### Remarks.

The name “*conjungens*” as mentioned in Sacco (1889: 68) is an incorrect subsequent spelling.

#### Melanopsis jordanica var. conoidaea

Taxon classificationAnimaliaSorbeoconchaMelanopsidae

Bourguignat, 1884

##### Original source.

Bourguignat 1884: 142.

##### Type locality.

“Lac de Tibériade” [Sea of Galilee], Israel.

#### † Melanopsis conoidea

Taxon classificationAnimaliaSorbeoconchaMelanopsidae

Pană, 2003

##### Original source.

Pană 2003: 319, pl. 9, figs 6–13.

##### Type horizon.

Parscovian–Pelendavian, Pliocene.

##### Type locality.

“Forage Mihăița, profondeur 109 m” [borehole Mihăița, at a depth of 109 m], Romania.

##### Types.

Laboratory of Paleontology, Bucharest, coll. no. 651.

##### Remarks.

Pană denoted the authorship as “Pană, 1989”. Originally the gender was indicated as masculine (“*conoideus*”), but *Melanopsis* is feminine, which is why the name must be “*conoidea*”.

#### † Melanopsis (Martinia) vindobonensis var. consimilis

Taxon classificationAnimaliaSorbeoconchaMelanopsidae

Handmann, 1887

##### Original source.

Handmann 1887: 29, pl. 6, figs 7–10.

##### Type horizon.

Pannonian, zone B–D, late Miocene.

##### Type locality.

“Leobersdorf”, Austria.

#### † Lyrcaea megacantha f. conspicua

Taxon classificationAnimaliaSorbeoconchaMelanopsidae

Brusina, 1897

##### Original source.

Brusina 1897: 13, pl. 7, figs 3–4.

##### Type horizon.

Pannonian, zone D–E, late Miocene.

##### Type locality.

“Begaljica”, Serbia.

##### Types.

The syntypes are stored in the Croatian Natural History Museum, Zagreb; no number indicated (Milan et al. 1974: 84).

##### Remarks.

Wenz (1929: 2682) considered this taxon as a junior synonym of *Melanopsis bouei megacantha* Handmann, 1887.

#### † Melanopsis conspicua

Taxon classificationAnimaliaSorbeoconchaMelanopsidae

Pallary, 1916 [invalid]

##### Original source.

Pallary 1916: 80.

##### Type horizon.

Chattian, Oligocene.

##### Type locality.

“Dax. St-Geours, Abesse” (Grateloup 1840: captions of the plate “Mollusques terrestres et fluviatiles fossiles de Dax”), France.

##### Remarks.

Introduced for *Melania costata* sensu Grateloup, 1840, for which d’Orbigny (1852) had already introduced *Melanopsis nereis* as new name. Thus, *Melanopsis conspicua* is a junior objective synonym of *Melanopsis nereis*. Moreover, it is a junior secondary homonym of *Lyrcaea conspicua* Brusina, 1897.

#### † Melanopsis constricta

Taxon classificationAnimaliaSorbeoconchaMelanopsidae

Brusina, 1878

##### Original source.

Brusina 1878: 348.

##### Type horizon.

Cernikian, Pliocene.

##### Type locality.

“Kozarica” [Kozarice], Croatia.

##### Types.

Milan et al. (1974: 88) stated that Brusina (1897) had indicated one of the specimens illustrated by him (pl. 5, fig. 21) as type. However, it is uncertain whether the specimen actually derives from the original type series. The specimen is stored in the Croatian Natural History Museum, Zagreb, coll. no. 2992-638/1.

#### † Melanopsis (Martinia) martiniana var. constricta

Taxon classificationAnimaliaSorbeoconchaMelanopsidae

Handmann, 1887 [invalid]

##### Original source.

Handmann 1887: 26, pl. 5, figs 1–2.

##### Type horizon.

Pannonian, zone B–D, late Miocene.

##### Type locality.

“Leobersdorf”, Austria.

##### Remarks.

Junior homonym of *Melanopsis constricta* Brusina, 1878. Fischer (1996a) introduced *Melanopsis handmanniana* as replacement name.

#### † Melanopsis vindobonensis var. contecta

Taxon classificationAnimaliaSorbeoconchaMelanopsidae

Handmann, 1882

##### Original source.

Handmann 1882: 554.

##### Type horizon.

Pannonian, zone D, late Miocene.

##### Type locality.

“Kottingbrunn”, Austria.

##### Remarks.

Wenz (1929: 2848) considered the taxon as a junior synonym of *Melanopsis vindobonensis* Fuchs, 1870. Note that Wenz gave the name as “*costata*”, which is an incorrect subsequent spelling.

#### † Melanopsis (Martinia) vindobonensis var. contigua

Taxon classificationAnimaliaSorbeoconchaMelanopsidae

Handmann, 1887

##### Original source.

Handmann 1887: 29, pl. 6, figs 11–12.

##### Type horizon.

Pannonian, zone B–D, late Miocene.

##### Type locality.

“Leobersdorf”, Austria.

#### † Melanopsis hazayi var. contracta

Taxon classificationAnimaliaSorbeoconchaMelanopsidae

Brusina, 1903 [invalid]

##### Original source.

Brusina 1903: 112.

##### Type horizon.

Late Pleistocene–early Holocene.

##### Type locality.

“Bischofsbad” [Püspökfürdő, Băile 1 Mai, Lake Pețea], Romania.

##### Remarks.

Junior objective synonym of *Melanopsis hazayi*: Brusina (1903) indicated it as the typical form of the species. Neubauer et al. (2014d: 125) considered this taxon as a junior synonym of *Microcolpia parreyssii sikorai* (Brusina, 1903).

#### † Melanopsis convexa

Taxon classificationAnimaliaSorbeoconchaMelanopsidae

Doncieux, 1908 

##### Original source.

Doncieux 1908: 202, pl. 11, fig. 10.

##### Type horizon.

Lutetian, Eocene.

##### Type locality.

“Au Nord d’Albas”, France.

#### Melanopsis cookiana

Taxon classificationAnimaliaSorbeoconchaMelanopsidae

Pallary, 1916 [invalid]

##### Original source.

Pallary 1916: 82.

##### Type locality.

“Prope Kanala [...]; insula Ouen” (Gassies 1870: 148) [near Canala; Île Ouen], New Caledonia.

##### Remarks.

Replacement name for the junior homonym *Melanopsis fusiformis* Gassies, 1870, non Sowerby, 1822, for which Gassies (1880) had already introduced *Melanopsis rossiteri* as replacement name. Thus, *Melanopsis cookiana* is a junior objective synonym of *Melanopsis rossiteri*.

#### † Melanopsis corici

Taxon classificationAnimaliaSorbeoconchaMelanopsidae

Neubauer, Mandic, Harzhauser & Hrvatović, 2013

##### Original source.

Neubauer et al. 2013: 134, figs 4E–H.

##### Type horizon.

Langhian, middle Miocene.

##### Type locality.

“Fatelj section”, Bosnia and Herzegovina.

##### Types.

Geological-Paleontological Department, Natural History Museum Vienna, Austria, coll. no. 2011/0138/0100.

#### Melanopsis cornea

Taxon classificationAnimaliaSorbeoconchaMelanopsidae

Pfeiffer, 1828

##### Original source.

Pfeiffer 1828: 50, pl. 8, figs 22–23.

##### Type locality.

“In der Donau bei Pesth” [in the Danube river near Budapest], Hungary.

#### Melania cornea

Taxon classificationAnimaliaSorbeoconchaMelanopsidae

Reeve, 1860

##### Original source.

Reeve 1860: Section *Melania*, pl. 34, fig. 233.

##### Type locality.

“Dalmatia” [no locality indicated], Croatia.

##### Remarks.

Based on a manuscript or “in schedis” name from Küster in the museum of Von dem Busch (see Reeve 1860).

#### Melania coronata

Taxon classificationAnimaliaSorbeoconchaMelanopsidae

Reeve, 1860 [invalid]

##### Original source.

Reeve 1860: Section *Melania*, pl. 34, fig. 228.

##### Type locality.

“Römerbad in Steiermark” [Rimske Toplice], Slovenia (after Clessin 1890, no locality given in Reeve 1860).

##### Remarks.

Based on a manuscript name by Küster and introduced in synonymy of *Melania hollandri* [sic] Pfeiffer, 1828 (see also Brot 1874 and Clessin 1890). It was made available by Bourguignat (1884), who treated it as valid name (although he denoted the authority with “Bourguignat, 1877”; see Note 2). Junior homonym of *Melania coronata* Von dem Busch in Philippi, 1845 (Bengal).

#### † Melanopsis (Lyrcea) coronata

Taxon classificationAnimaliaSorbeoconchaMelanopsidae

Brusina, 1878

##### Original source.

Brusina 1878: 348.

##### Type horizon.

Cernikian, Pliocene.

##### Type locality.

“Repusnica, Slobodnica”, Croatia.

##### Types.

The syntype (?) illustrated in Brusina (1897: 12, pl. 5, figs 15–16) is stored in the Croatian Natural History Museum, Zagreb, coll. no. 2987-633 (Milan et al. 1974: 84).

#### Melanopsis tricarinata coronata

Taxon classificationAnimaliaSorbeoconchaMelanopsidae

Ahuir Galindo, 2015 [invalid]

##### Original source.

Ahuir Galindo 2015: 22, unnumbered figure.

##### Type locality.

“In brooks and ‘gorgos’ from Anna, Valencia”, Spain.

##### Types.

Museo Malacologico di Cupra Marittima, Italy; no number indicated.

##### Remarks.

Junior homonym of *Melanopsis coronata* Brusina, 1878.

#### Melanopsis obediensis var. coroniformis

Taxon classificationAnimaliaSorbeoconchaMelanopsidae

Picard, 1934

##### Original source.

Picard 1934: 121, pl. 7, figs 45–52.

##### Type locality.

“Jarmukmündung” [Yarmouk river mouth], Jordan/Israel.

#### † Melanopsis rumana var. correcta

Taxon classificationAnimaliaSorbeoconchaMelanopsidae

Stefanescu, 1896

##### Original source.

Stefanescu 1896: 128, pl. 11, figs 4–6.

##### Type horizon.

Pliocene.

##### Type locality.

“À Mosculesti, dans la vallée de Gilortu, à Gura-Motrului et à Breasta, dans la vallée de Jiu” [at Musculești in the valley of the river Gilortu, at Gura Motrului and at Breasta in the valley of the river Jiu], Romania.

#### † Melanopsis corrugata

Taxon classificationAnimaliaSorbeoconchaMelanopsidae

Schütt in Schütt & Ortal, 1993 

##### Original source.

Schütt and Ortal 1993: 91, pl. 2, figs 15–16.

##### Type horizon.

Pleistocene, Riss glacial epoch.

##### Type locality.

“Galilee, southern Hula basin, about 500 m north of the new bridge over the river Jordan at Benot Ya’Aqov”, Israel.

##### Types.

Paleontology Collection of the Hebrew University of Jerusalem; no number indicated.

#### † Melanopsis cosiana

Taxon classificationAnimaliaSorbeoconchaMelanopsidae

Pallary, 1925 [invalid]

##### Original source.

Pallary 1925: 257.

##### Type horizon.

Plio-Pleistocene.

##### Type locality.

“Zwischen Pylle und Antimachia” (Neumayr 1880b: 295) [between Pýli and Antimácheia, Kos Island], Greece.

##### Remarks.

Invalid replacement name for *Melanopsis broti* Neumayr, 1880, “non Gassies, 1874”, but Gassies had actually introduced his species as “*Melanopsis brotiana*”.

#### † Melanopsis cosmanni

Taxon classificationAnimaliaSorbeoconchaMelanopsidae

[sic] Pallary, 1916 [invalid]

##### Original source.

Pallary 1916: 80.

##### Type horizon.

Cernikian, Pliocene.

##### Type locality.

“De Slavonie” (Cossmann 1909: 176), Croatia.

##### Remarks.

Introduced for *Melania costata* sensu Cossmann, 1909, non Olivier, 1804. The name was corrected to “*cossmanni*” by Neubauer et al. (2014a: 461), which is a justified emendation according to Art. 32.5.1 and 33.2.2. Wenz (1929) considered all records of “*Melania costata*” from the Pliocene of Slavonia to represent the same species and synonymized them with *Melanopsis abbreviata cosmanni*. Obviously, he was unaware that *Melanopsis croatica* Brusina, 1884 is the first available name for them (see also discussion of *Melanopsis pseudocostata* Oppenheim, 1890).

#### † Melanopsis cossmanni

Taxon classificationAnimaliaSorbeoconchaMelanopsidae

Pallary, 1916

##### Original source.

Pallary 1916: 80 (as “*cosmanni*”).

##### Type horizon.

Cernikian, Pliocene.

##### Type locality.

“De Slavonie” (Cossmann 1909: 176), Croatia.

##### Remarks.

Justified emendation of *Melanopsis cosmanni* Pallary, 1916 by Neubauer et al. (2014a: 461).

#### Melanopsis cossoni

Taxon classificationAnimaliaSorbeoconchaMelanopsidae

Bourguignat, 1884 

##### Original source.

Bourguignat 1884: 111.

##### Type locality.

“Eaux thermales d’Ouargla et près du chott Tiraoun dans le sud de la province de Constantine et de la Tunisie” [in thermal waters at Ouargla (Algeria) and near chott Tiraoun (Tunisia)], Algeria.

#### Melania costata

Taxon classificationAnimaliaSorbeoconchaMelanopsidae

Olivier, 1804

##### Original source.

Olivier 1804: 294 (footnote), pl. 31, fig. 3.

##### Type locality.

“De Orontes [Gesser-Chourl]” [in the Orontes river, at Jisr Ash-Shughur], Syria.

##### Remarks.

Type species of the genus *Melanopsis* Férussac in Férussac & Férussac, 1807.

#### † Melanopsis costata

Taxon classificationAnimaliaSorbeoconchaMelanopsidae

Ludwig, 1865 [invalid]

##### Original source.

Ludwig 1865: 71, pl. 21, figs 7–7b.

##### Type horizon.

Early Rupelian, Oligocene.

##### Type locality.

“Grossalmerode”, Germany.

##### Remarks.

Junior secondary homonym of *Melania costata* (Olivier, 1804). Speyer (1870: 97) introduced *Melanopsis ludwigi* as replacement name.

#### † Melanopsis bergeroni var. costata

Taxon classificationAnimaliaSorbeoconchaMelanopsidae

Botez, 1914 [invalid]

##### Original source.

Botez 1914: 240.

##### Type horizon.

*Viviparus stricturatus* Zone, Cernikian, Pliocene.

##### Type locality.

“Moreni”, Romania.

##### Remarks.

Junior secondary homonym of *Melanopsis costata* (Olivier, 1804).

#### † Melanopsis lanzae costata

Taxon classificationAnimaliaSorbeoconchaMelanopsidae

Olujić, 1999 [invalid]

##### Original source.

Olujić 1999: 21, 49, pl. 2, figs 19–24, pl. 3, figs 26–28.

##### Type horizon.

Langhian, middle Miocene.

##### Type locality.

It is unclear from the original work in which of the studied localities/sections along the valleys of the Sutina, Batarelov and Vojskava rivers (4 km W of Sinj) the taxon occurred and in which not, Croatia.

##### Remarks.

Junior secondary homonym of *Melanopsis costata* (Olivier, 1804). Neubauer et al. (2011) considered it as a junior synonym of *Melanopsis lanzaeana*.

#### † Melanoptychia lyrata costata

Taxon classificationAnimaliaSorbeoconchaMelanopsidae

Olujić, 1999 [invalid] 

##### Original source.

Olujić 1999: 20, 48, pl. 1, fig. 5.

##### Type horizon.

Langhian, middle Miocene.

##### Type locality.

It is unclear from the original work in which of the studied localities/sections along the valleys of the Sutina, Batarelov and Vojskava rivers (4 km W of Sinj) the taxon occurred and in which not, Croatia.

##### Remarks.

Junior secondary homonym of *Melanopsis costata* (Olivier, 1804). Considered as a junior synonym of *Melanopsis lyrata* Neumayr, 1869 by Neubauer et al. (2011: 207).

#### “ Mellanella hollandri [sic] var. costala

Taxon classificationAnimaliaSorbeoconchaMelanopsidae

[sic] Kucik” mentioned in Brusina (1867: 85) [unavailable]

##### Locality.

“U Savi i u potoku kod Susjeda, u Žirovcu, u Maksimiru kod Zagreba, u potoku Toplici kod Oroslavja i u Kutinji blizu Jastrebarskoga” [in the river Sava and a creek at Podsused in Zagreb, in Žirovac, in Maksimir at Zagreb, in the river Toplica at Oroslavje and in Kutinja (?) near Jastrebarsko], Croatia.

##### Remarks.

Nomen nudum, based on an “in schedis” name from the collection of Kucik (also read as “Kutschig”). The name was probably a typesetting mistake for “*costata*”.

#### † Melanopsis (Canthidomus) costatiformis

Taxon classificationAnimaliaSorbeoconchaMelanopsidae

Papp, 1953

##### Original source.

Papp 1953a: 108, pl. 23, figs 1–8.

##### Type horizon.

Gelasian, early Pleistocene.

##### Type locality.

“Aghios Georgios” [Agios Georgios, Elis], Greece.

##### Types.

Museum of Palaeontology and Geology of the University of Athens; no number indicated.

#### Melanopsis costellata

Taxon classificationAnimaliaSorbeoconchaMelanopsidae

Férussac, 1823 [invalid]

##### Original source.

Férussac 1823: 157.

##### Type locality.

“Dans l’aqueduc de Séville [...] et dans les ruisseaux des environs. [...] Dans les lacs et les rivières du royaume de Maroc” [in the aqueduct of Sevilla and in the rivers of its surroundings (Spain); in the lakes and rivers of Morocco].

##### Remarks.

Junior objective synonym of *Melanopsis cariosa* (Linnaeus, 1767), which Férussac listed in synonymy.

#### † Melanopsis costellata

Taxon classificationAnimaliaSorbeoconchaMelanopsidae

Douvillé, 1904 [invalid] 

##### Original source.

Douvillé 1904: 327, pl. 46, figs 7–11.

##### Type horizon.

Maastrichtian, Cretaceous.

##### Type locality.

“Du versant oriental du Kouh Mapeul” [eastern slope of mount Kuh-e Mapel, c. 60 km WNW of Khorramābād], Iran.

##### Remarks.

Junior homonym of *Melanopsis costellata* Férussac, 1823. Pallary (1916: 83) introduced *Melanopsis douvillei* as replacement name.

#### † Pseudofagotia costifera

Taxon classificationAnimaliaSorbeoconchaMelanopsidae

Anistratenko, 1993

##### Original source.

Anistratenko 1993: 74, textfig. 2.

##### Type horizon.

Duab Beds, middle to late Kimmerian, Pliocene.

##### Type locality.

“Окр. с. Мокви, Очамчирский р-н” [near the village Mok’vi, Ochamchirskiy rayon], Georgia.

##### Types.

Schmalhausen Institute of Zoology of National Academy of Sciences of Ukraine, Kiev; no number indicated.

#### Melania holandri var. costulata

Taxon classificationAnimaliaSorbeoconchaMelanopsidae

Schmidt, 1847

##### Original source.

Schmidt 1847: 25.

##### Type locality.

“Aus einem Mühlbache bei Klinze ob Schischka” [from a mill creek at Glinica near Šiška], Slovenia.

#### † Melanopsis inconstans var. costulata

Taxon classificationAnimaliaSorbeoconchaMelanopsidae

Brusina, 1874 [invalid]

##### Original source.

Brusina 1874: 39.

##### Type horizon.

Langhian, middle Miocene.

##### Type locality.

“Miočić”, Croatia.

##### Types.

The syntypes are stored in the Croatian Natural History Museum, Zagreb; no number indicated (Milan et al. 1974: 92).

##### Remarks.

Brusina (1874) divided the species *Melanopsis inconstans* Neumayr, 1869 into three varieties, none of which he termed “*inconstans*”. The first one, “var. *costulata*”, he referred to as the typical one, which makes it an objective synonym of the nominal subspecies and hence *Melanopsis inconstans*.

#### † Melanopsis visianiana f. costulata

Taxon classificationAnimaliaSorbeoconchaMelanopsidae

Brusina, 1897 [invalid]

##### Original source.

Brusina 1897: 12, pl. 5, fig. 6.

##### Type horizon.

Langhian, middle Miocene.

##### Type locality.

“Miočić”, Croatia.

##### Types.

Milan et al. (1974: 99) indicated a holotype, but it is uncertain whether the specimen was the only one Brusina had at hand (holotype by monotypy, Art. 73.1.2). The specimen is stored in the Croatian Natural History Museum, Zagreb, coll. no. 2981-627/2.

##### Remarks.

Junior homonym of *Melanopsis inconstans costulata* Brusina, 1874 (see Note 1). Currently considered as a junior synonym of *Melanopsis visianiana* Brusina, 1874 (Neubauer et al. 2016b: 25).

#### Melanopsis laevigata var. costulata

Taxon classificationAnimaliaSorbeoconchaMelanopsidae

Pallary, 1899 [invalid]

##### Original source.

Pallary 1899: 137.

##### Type locality.

“Souani près Tanger” [Souani near Tanger], Morocco.

##### Remarks.

Junior homonym of *Melanopsis costulata* Brusina, 1897.

#### † Melanopsis staubi var. costulata

Taxon classificationAnimaliaSorbeoconchaMelanopsidae

Brusina, 1903 [invalid]

##### Original source.

Brusina 1903: 115.

##### Type horizon.

Late Pleistocene–early Holocene.

##### Type locality.

“Bischofsbad” [Püspökfürdő, Băile 1 Mai, Lake Pețea], Romania.

##### Remarks.

Junior homonym of *Melanopsis costulata* Brusina, 1897. Neubauer et al. (2014d: 125) considered this taxon as a junior synonym of *Microcolpia parreyssii sikorai* (Brusina, 1903).

#### † Melanopsis (Coptostylus) costulata

Taxon classificationAnimaliaSorbeoconchaMelanopsidae

Doncieux, 1908 [invalid]

##### Original source.

Doncieux 1908: 205, pl. 11, figs 13a–13b.

##### Type horizon.

Middle Lutetian, Eocene.

##### Type locality.

“Au Nord d’Albas” (Doncieux 1908: 205), France.

##### Remarks.

Junior homonym of *Melanopsis costulata* Brusina, 1897. Has been considered to belong to the genus *Coptostylus* Sandberger, 1872 (Thiaridae).

#### Melanopsis vondeli var. costulata

Taxon classificationAnimaliaSorbeoconchaMelanopsidae

Pallary, 1928 [invalid]

##### Original source.

Pallary 1928b: 16, pl. 2, fig. 13.

##### Type locality.

“O. Taguenout” [Oued Taguenout, said to be near Beni Mellal], Morocco.

##### Remarks.

Junior homonym of *Melanopsis costulata* Brusina, 1897.

#### Melanopsis (Mesopotamia) infracincta var. costulata

Taxon classificationAnimaliaSorbeoconchaMelanopsidae

Pallary, 1939 [invalid] 

##### Original source.

Pallary 1939: 104, pl. 4, fig. 13.

##### Type locality.

“Ras el ‘Ain du Khabour” [Chabur river near Ra’s al ‘Ayn], Syria.

##### Remarks.

Junior homonym of *Melanopsis costulata* Brusina, 1897.

#### † Melanopsis cotrocenensis

Taxon classificationAnimaliaSorbeoconchaMelanopsidae

Cobălcescu, 1883

##### Original source.

Cobălcescu 1883: 123, pl. 9, figs 8a–b.

##### Type horizon.

Cernikian, Pliocene.

##### Type locality.

“Cotroceni lăngă București” [Cotroceni near Bucarest], Romania.

#### Melanopsis coupha

Taxon classificationAnimaliaSorbeoconchaMelanopsidae

Bourguignat, 1884

##### Original source.

Bourguignat 1884: 94.

##### Type locality.

“Dans les eaux chaudes du Djerid, au nord du chott Tiraoun, dans le sud de la Tunisie” [in the warm water of Djérid, north of chott Tiraoun], Tunisia.

#### Microcolpia coutagniana

Taxon classificationAnimaliaSorbeoconchaMelanopsidae

Bourguignat, 1884

##### Original source.

Bourguignat 1884: 56.

##### Type locality.

“Lac Sabandja, près d’Ismidt, en Anatolie” [Lake Sapanca near İzmit], Turkey.

#### † Melanopsis covurluensis

Taxon classificationAnimaliaSorbeoconchaMelanopsidae

Cobălcescu, 1883

##### Original source.

Cobălcescu 1883: 123, pl. 9, figs 7a–d.

##### Type horizon.

Pliocene.

##### Type locality.

“Barboschi” (p. 156) [Barboși], Romania.

#### Melania crassa

Taxon classificationAnimaliaSorbeoconchaMelanopsidae

Brusina, 1866 [invalid]

##### Original source.

Brusina 1866: 106.

##### Type locality.

“In der Muhr” (Pfeiffer 1828: 48) [in the river Mur], Austria.

##### Remarks.

Brusina (1866) introduced the name for “var. A” in Pfeiffer (1828), which that author, however, had affiliated with the name “*Melania agnata* Ziegler” (see Pfeiffer 1828: 67). Therefore, *Melania crassa* Brusina, 1866 is a junior objective synonym of *Melania agnata*. Moreover, it is a junior homonym of *Melania crassa* Münster, 1841. Note that the latter species is certainly no Melanopsidae as it derives from the marine deposits of the Triassic St. Cassian Fm.

#### † Melanopsis anceps var. crassicosta

Taxon classificationAnimaliaSorbeoconchaMelanopsidae

Gaudry & P. Fischer in Gaudry, 1867

##### Original source.

Gaudry 1862–1867: 446.

##### Type horizon.

Pliocene.

##### Type locality.

“Mégare” (p. 444), Greece.

#### Melanella crassilabris

Taxon classificationAnimaliaSorbeoconchaMelanopsidae

Bourguignat, 1884

##### Original source.

Bourguignat 1884: 16.

##### Type locality.

“La Save à Agram et rivière d’Ostaria en Croatie” [Sava river at Zagreb and Oštarije river], Croatia.

##### Remarks.

Note that Bourguignat denoted the authority as “Bourguignat, 1879”.

#### † Melanopsis crassitesta

Taxon classificationAnimaliaSorbeoconchaMelanopsidae

Blanckenhorn, 1897

##### Original source.

Blanckenhorn 1897: 134, pl. 10, fig. 18.

##### Type horizon.

Plio-Pleistocene.

##### Type locality.

“In der pliocänen Dreissensiaschicht von Dschisr esch-Schurr” [in the pliocene *Dreissena* layer at Jisr Ash-Shughur], Syria.

#### † Melanopsis crastina

Taxon classificationAnimaliaSorbeoconchaMelanopsidae

Vidal, 1874

##### Original source.

Vidal 1874: 235, pl. 2, fig. 12, pl. 5, figs 32–34.

##### Type horizon.

Maastrichtian, Cretaceous.

##### Type locality.

“Isona”, Spain.

#### Melanopsis crenocarinata

Taxon classificationAnimaliaSorbeoconchaMelanopsidae

Moricand, 1841

##### Original source.

Moricand 1841: 61, pl. 4, figs 10–11.

##### Type locality.

“Rio de Pedra Branca, procince de Bahia” [Pedra Branca river, province Bahia], Brazil.

##### Remarks.

Type species of the genus *Verena* H. Adams & A. Adams, 1854 (Thiaridae) (see Nuttall 1990: 253).

#### † Melanopsis maresi var. crenulata

Taxon classificationAnimaliaSorbeoconchaMelanopsidae

Pallary, 1901

##### Original source.

Pallary 1901a: 180, pl. 2, fig. 22.

##### Type horizon.

Pleistocene.

##### Type locality.

“De Géryville” [Aïn Sefra], Algeria.

#### Melanopsis fasensis var. cristata

Taxon classificationAnimaliaSorbeoconchaMelanopsidae

Pallary, 1920

##### Original source.

Pallary 1920c: 147, pl. 4, fig. 13.

##### Type locality.

“Fès”, Morocco.

#### † Melanopsis croatica

Taxon classificationAnimaliaSorbeoconchaMelanopsidae

Brusina, 1884

##### Original source.

Brusina 1884a: 168.

##### Type horizon.

Cernikian, Pliocene.

##### Type locality.

“Repušnica” (Neumayr 1869: 372), Croatia.

##### Types.

Milan et al. (1974: 89) indicated a holotype, but it is uncertain whether the specimen actually derives from the original type series and whether it was the only specimen Brusina had at hand. The specimen is stored in the Croatian Natural History Museum, Zagreb, coll. no. 2988-634.

##### Remarks.

Introduced for *Melania costata* sensu Neumayr, 1969, non Olivier, 1804. Wenz (1929) considered all records of “*Melania costata*” from the Pliocene of Slavonia to represent the same species and synonymized them with *Melanopsis abbreviata cosmanni*. Obviously, he was unaware that *Melanopsis croatica* Brusina, 1884 is the first available name for them (see also discussion of *Melanopsis pseudocostata* Oppenheim, 1890).

#### † Melanopsis austriaca croatica

Taxon classificationAnimaliaSorbeoconchaMelanopsidae

Brusina, 1902 [invalid]

##### Original source.

Brusina 1902: pl. 6, figs 71–72.

##### Type horizon.

Middle Pannonian, late Miocene.

##### Type locality.

“Markuševec”, Croatia.

##### Types.

The illustrated syntypes are stored in the Croatian Natural History Museum, Zagreb, coll. no. 2529-175/1-2 (Milan et al. 1974: 86).

##### Remarks.

Junior homonym of *Melanopsis croatica* Brusina, 1884. Wenz (1930) introduced *Melanopsis haueri markusevecensis* as replacement name.

#### † Melanopsis cuisiensis

Taxon classificationAnimaliaSorbeoconchaMelanopsidae

Dominici & Kowalke, 2014

##### Original source.

Dominici and Kowalke 2014: 147, pl. 2, figs 5a–b.

##### Type horizon.

Castigaleu group, late Ypresian, Eocene.

##### Type locality.

“Morillo de Lena (Esera valley, Tremp-Graus basin), [...] CG-A2, sample 53”, Spain.

##### Types.

Museo di Storia Naturale, Università degli Studi di Firenze, coll. no. IGF 4334E.

#### Melanopsis curta

Taxon classificationAnimaliaSorbeoconchaMelanopsidae

Gassies, 1870

##### Original source.

Gassies 1870: 146.

##### Type locality.

“Tuo” [Touho], New Caledonia.

##### Remarks.

Brot (1879: 444) considered the taxon as a junior synonym of *Melanopsis frustulum* Morelet, 1857.

#### Melanopsis turcica var. curta

Taxon classificationAnimaliaSorbeoconchaMelanopsidae

Locard, 1883 [invalid]

##### Original source.

Locard 1883a: 270.

##### Type locality.

“Lac d’Antioche” [Lake Anuk (also as Amik)], Turkey.

##### Remarks.

Junior homonym of *Melanopsis curta* Gassies, 1870.

#### Melanopsis cerithiopsis var. curta

Taxon classificationAnimaliaSorbeoconchaMelanopsidae

Bourguignat, 1884 [invalid]

##### Original source.

Bourguignat 1884: 131.

##### Type locality.

“Plaine du Bahr-el-Houlé (haut Jourdain) dans l’Aïn-el-Mellaha” [in the plains of the Hula valley (upper Jordan), in Aïn Mallahah], Israel.

##### Remarks.

Junior homonym of *Melanopsis curta* Gassies, 1870. Heller et al. (2005: 248) considered the variety as a junior synonym of *Melanopsis saulcyi* Bourguignat, 1853.

#### † Melanopsis callosa var. curta

Taxon classificationAnimaliaSorbeoconchaMelanopsidae

Locard, 1893 [invalid]

##### Original source.

Locard 1893: 181, pl. 9, fig. 20.

##### Type horizon.

Late Burdigalian–Langhian, early–middle Miocene.

##### Type locality.

“Le Locle; [...] Vermes”, France.

##### Remarks.

Junior homonym of *Melanopsis curta* Gassies, 1870. Wenz (1929: 2764) considered the variety as a junior synonym of *Melanopsis kleini* Kurr, 1856.

#### “ Melanopsis praemorsa f. curta

Taxon classificationAnimaliaSorbeoconchaMelanopsidae

” mentioned in Pérès (1939) [unavailable] 

##### Locality.

“Station 119. Aïn Attig. Source près de la route de Rabat à Casablanca à 13 kilomètres de Rabat” [station 119 at Ain Attig. A spring near the road from Rabat to Casablanca, 13 km from Rabat], Morocco.

##### Remarks.

First of all, the name as given by Pérès (1939) is a nomen nudum – Pérès apparently considered the expression “*curta*” self-explanatory and did not describe it. Moreover, he obviously used the name not as separate taxon but rather as descriptive term to fit existing species into his morphological concept. He even indicated *Melanopsis brevis* Morelet, 1857 as its “type”.

#### “ Melania cuspidata

Taxon classificationAnimaliaSorbeoconchaMelanopsidae

Parreyss” mentioned in Brot (1874–1879: 12) [unavailable]

##### Locality.

Not indicated.

##### Remarks.

Nomen nudum, “in schedis” name from Parreyss listed in the synonymy list of “*Melania holandri*” [sic] by Brot (1874).

#### † Melanopsis cvijici

Taxon classificationAnimaliaSorbeoconchaMelanopsidae

Brusina, 1902

##### Original source.

Brusina 1902: pl. 29, figs 19–22.

##### Type horizon.

Langhian, middle Miocene.

##### Type locality.

“Vatelj” [Fatelj hill], Bosnia and Herzegovina.

##### Types.

Milan et al. (1974: 89) stated that only one of the specimens illustrated by Brusina (1902: pl. 29, figs 19–20) has been preserved, which they designated as lectotype. The specimen is stored in the Croatian Natural History Museum, Zagreb, coll. no. 2900-546/1.

#### † Melanopsis lyrata var. cylindracea

Taxon classificationAnimaliaSorbeoconchaMelanopsidae

Brusina, 1874

##### Original source.

Brusina 1874: 45.

##### Type horizon.

Langhian, middle Miocene.

##### Type locality.

“Ribarić”, Croatia.

##### Remarks.

Currently considered as a junior synonym of *Melanopsis lyrata* Neumayr, 1869 (Neubauer et al. 2016b: 24; see also Wenz 1929: 2698).

#### † Melanopsis cylindrata

Taxon classificationAnimaliaSorbeoconchaMelanopsidae

Blanckenhorn, 1897

##### Original source.

Blanckenhorn 1897: 136, pl. 10, figs 22–24.

##### Type horizon.

Plio-Pleistocene.

##### Type locality.

“Im Rab zwischen Dschisr esch-Schurr und Kal ‘at el-Mdik; [...] in der pliocänen Dreissensiaschicht von Dschisr esch-Schurr auf dem rechten Orontesufer” [in the Al Ghāb between Jisr Ash-Shughur and Qal’at al Maḑīq; in the Pliocene *Dreissena* layer at Jisr Ash-Shughur at the right riverbank of the Orontes], Syria.

#### † Lyrcea cylindrica

Taxon classificationAnimaliaSorbeoconchaMelanopsidae

Stoliczka, 1862

##### Original source.

Stoliczka 1862: 537, pl. 17, fig. 9.

##### Type horizon.

Transdanubian, Pannonian, late Miocene.

##### Type locality.

“Bei Zala Apati; [...] in der Umgegend des Plattensees, wie auf der Halbinsel Tihany” [near Zalaapáti; in the surroundings of Lake Balaton, as well as on the Tihany peninsula], Hungary.

#### † Melanopsis (Duabiana) cylindrica

Taxon classificationAnimaliaSorbeoconchaMelanopsidae

Anistratenko, 1993 [invalid]

##### Original source.

Anistratenko 1993: 69.

##### Type horizon.

Duab Beds, middle to late Kimmerian, Pliocene.

##### Type locality.

“Окр. с. Мокви, Очамчирский р-н” [near the village Mok’vi, Ochamchirskiy rayon], Georgia.

##### Types.

Schmalhausen Institute of Zoology of National Academy of Sciences of Ukraine, Kiev; no number indicated.

##### Remarks.

Type species of *Duabiana* Starobogatov & Anistratenko in Anistratenko, 1993. Junior secondary homonym of *Lyrcea cylindrica* Stoliczka, 1862. Neubauer et al. (2014a: 456) introduced *Melanopsis anistratenkoi* as replacement name.

#### † Melanopsis (Duabiana) cylindrospica

Taxon classificationAnimaliaSorbeoconchaMelanopsidae

Anistratenko, 1993

##### Original source.

Anistratenko 1993: 71, textfig. 1.

##### Type horizon.

Duab Beds, middle to late Kimmerian, Pliocene.

##### Type locality.

“Окр. с. Мокви, Очамчирский р-н” [near the village Mok’vi, Ochamchirskiy rayon], Georgia.

##### Types.

Schmalhausen Institute of Zoology of National Academy of Sciences of Ukraine, Kiev; no number indicated.

#### † Melanoptychia dalmatina

Taxon classificationAnimaliaSorbeoconchaMelanopsidae

Bourguignat, 1880

##### Original source.

Bourguignat 1880: 31.

##### Type horizon.

Langhian, middle Miocene.

##### Type locality.

“Vallée de la Cettina” [Cetina river valley], Croatia.

##### Remarks.

The taxon is not included in the Fossilium Catalogus of Wenz (1929).

#### † Melanopsis dalmatina

Taxon classificationAnimaliaSorbeoconchaMelanopsidae

Brusina, 1884 [invalid]

##### Original source.

Brusina 1884b: 55.

##### Type horizon.

Early Langhian, middle Miocene.

##### Type locality.

“Oberes Niveau v. Zupića potok” (p. 47) [upper horizon of the Župića potok (near Sinj)], Croatia.

##### Types.

Milan et al. (1974: 90) stated that Brusina (1897) had indicated one of the specimens illustrated by him (pl. 5, fig. 10) as type. However, it is uncertain whether the specimen actually derives from the original type series. The specimen is stored in the Croatian Natural History Museum, Zagreb, coll. no. 2984-630/1.

##### Remarks.

Junior secondary homonym of *Melanoptychia dalmatina* Bourguignat, 1880.

#### Fagotia danubialis

Taxon classificationAnimaliaSorbeoconchaMelanopsidae

Bourguignat, 1884

##### Original source.

Bourguignat 1884: 35.

##### Type locality.

“Le Danube à Ibraila; la Save à Agram; la Krapina à Sused (Croatie)” [Danube river at Brăila (Romania); Sava river at Zagreb; Krapina river at Podsused, in Zagreb (Croatia)].

##### Remarks.

Note that Bourguignat denoted the authority as “Bourguignat, 1880”.

#### † Melanopsis daphnes

Taxon classificationAnimaliaSorbeoconchaMelanopsidae

Gaudry & P. Fischer in Gaudry, 1867

##### Original source.

Gaudry 1862–1867: 407, pl. 62, figs 16–18.

##### Type horizon.

Late Miocene.

##### Type locality.

“Daphné”, Greece.

##### Remarks.

The species epithet is a noun in apposition and needs not to agree in gender with the generic name (Art. 31.2.1). The name “*daphne*” as mentioned in Neumayr (1869: 369) and Hoernes (1876: 16) is an incorrect subsequent spelling. Wenz (1929: 2772) considered the taxon as a junior synonym of *Melanopsis longa* Deshayes in Férussac, 1839.

#### Melanopsis daudebartii

Taxon classificationAnimaliaSorbeoconchaMelanopsidae

[Prevost], 1821

##### Original source.

[Prevost] 1821: 137.

##### Type locality.

“Auprès de Baden, en Autriche, dans un bassin d’eau thermale sulfureuse” [near Baden, Austria, in a sulphurous thermal water basin], Austria.

##### Remarks.

There is considerable uncertainty about the correct authority and spelling of this species in the literature. It was first mentioned and validly described in the year 1821 as “*Melanopsis Daudebartii*” in an article in the Bulletin des Sciences, par la Société philomatique de Paris. Constant Prevost was often considered to be the author of this article, but from the title and text it is obvious that the article is an “Extrait” of a talk given by Prevost earlier and summarized by an anonymous author. According to Art. 50.2 and Recommendation 51D, the correct citation should be *Melanopsis daudebartii* [Prevost], 1821. The names “*daudebarti*”, “*audebarti*” or “*audebardi*”, each occurring multiple times in the literature, are incorrect subsequent spellings. Currently, the species is classified within the genus *Microcolpia* (see also Neubauer et al. 2014d: 126).

#### † Melanopsis dautzenbergi

Taxon classificationAnimaliaSorbeoconchaMelanopsidae

Pallary, 1901

##### Original source.

Pallary 1901a: 179, pl. 2, fig. 25.

##### Type horizon.

Pleistocene.

##### Type locality.

“De l’Oued Tiout (Sud-Oranais)” [Tiout], Algeria.

#### Melanopsis debilis

Taxon classificationAnimaliaSorbeoconchaMelanopsidae

Pallary, 1920

##### Original source.

Pallary 1920a: 33.

##### Type locality.

“Sidi Yahia, près d’Oudjda” [Sidi Yahya near Oujda], Morocco.

#### Melanopsis (Mesopotamia) infracincta var. debilis

Taxon classificationAnimaliaSorbeoconchaMelanopsidae

Pallary, 1939 [invalid]

##### Original source.

Pallary 1939: 104.

##### Type locality.

“Ras el ‘Ain du Khabour” [Chabur river near Ra’s al ‘Ayn], Syria.

##### Remarks.

Junior homonym of *Melanopsis debilis* Pallary, 1920.

#### † Melanopsis decipiens

Taxon classificationAnimaliaSorbeoconchaMelanopsidae

P. Fischer, 1883

##### Original source.

Fischer 1883: 60, pl. 3, fig. 3.

##### Type horizon.

Late Miocene.

##### Type locality.

“Smendou, province de Constantine, Algérie” [Zighoud Youcef], Algeria.

##### Remarks.

Fischer attributed the authority to Tournouër, but from the foregoing introduction it is clear that the species was described by Fischer.

#### † Melanopsis decollata

Taxon classificationAnimaliaSorbeoconchaMelanopsidae

Stoliczka, 1862 

##### Original source.

Stoliczka 1862: 536, pl. 17, fig. 8.

##### Type horizon.

Late Transdanubian, Pannonian, late Miocene.

##### Type locality.

“Bei Zala Apati am rechten Ufer der Zala und [...] im Gebiete des Plattensees” [near Zalaapáti at the right riverside of the Zala river and in the area around Lake Balaton], Hungary.

#### Melanopsis praerosa [sic] var. decollata

Taxon classificationAnimaliaSorbeoconchaMelanopsidae

Paetel, 1888 [invalid]

##### Original source.

Paetel 1888: 403.

##### Type locality.

“Persia” (Nevill 1884: 209), Iran.

##### Remarks.

Originally introduced as infrasubspecific taxon (“subvariety”) by Nevill (1884), but made available by Paetel (1888) who treated it as variety (Art. 45.5.1). Paetel clearly referred to the description of Nevill. Junior homonym of *Melanopsis decollata* Stoliczka, 1862.

#### † Melanopsis bouei var. decorata

Taxon classificationAnimaliaSorbeoconchaMelanopsidae

Pallary, 1916

##### Original source.

Pallary 1916: 85.

##### Type horizon.

Late Miocene or Pliocene.

##### Type locality.

Sabba Stefanescu, to whom Pallary referred, did not denote the localities of the figured specimens. He reported *Melanopsis bouei* from many localities in Romania.

##### Remarks.

Introduced for a part of Stefanescu’s material of *Melanopsis bouei* (Stefanescu 1896: pl. 11, figs 63–64). Wenz (1929: 2681) considered the taxon as a junior synonym of *Melanopsis bouei* Férussac, 1823.

#### Melanopsis douttei var. decorata

Taxon classificationAnimaliaSorbeoconchaMelanopsidae

Pallary, 1920 [invalid]

##### Original source.

Pallary 1920c: 150, pl. 4, fig. 2.

##### Type locality.

“La Makina”, Morocco.

##### Remarks.

Junior homonym of *Melanopsis decorata* Pallary, 1916.

#### † Melanopsis decostata

Taxon classificationAnimaliaSorbeoconchaMelanopsidae

Penecke, 1884

##### Original source.

Penecke 1884: 22, pl. 10, fig. 7.

##### Type horizon.

Cernikian, Pliocene.

##### Type locality.

“Repusnica” [Repušnica], Croatia.

##### Remarks.

The name was also marked as new taxon by Brusina (1902: vii). It is uncertain whether Brusina really intended to introduce a new subspecies or actually referred to the species of Penecke (1884), which he had ranked as subspecies of *Melanopsis croatica* earlier (Brusina 1897).

#### Melanopsis decussata

Taxon classificationAnimaliaSorbeoconchaMelanopsidae

Férussac, 1823

##### Original source.

Férussac 1823: 159.

##### Type locality.

“Plattensée, en Hongrie [...]; à Stary Maydan Zakrzewski, dans le gouvernement de Podolie, non loin de Kamieniec-Podolsk” [Lake Balaton; at Staryy Zakrevskiy Maydan, not far from Kam’yanets’-Podil’s’kyi], Hungary.

##### Remarks.

Considered as a junior synonym of “*Hemisinus Esperi*” Férussac (1823) by Brot (1878: 372).

#### † Melanopsis defensa

Taxon classificationAnimaliaSorbeoconchaMelanopsidae

Fuchs, 1870

##### Original source.

Fuchs 1870a: 353, pl. 14, figs 77–79.

##### Type horizon.

Transdanubian, Pannonian, late Miocene.

##### Type locality.

“Radmanest” [Rădmănești], Romania.

#### Melanopsis costata var. degenerata

Taxon classificationAnimaliaSorbeoconchaMelanopsidae

Preston, 1914

##### Original source.

Preston 1914: 467, pl. 27, fig. 9.

##### Type locality.

“Lake of Tiberias [Sea of Galilee] at the exit of the Jordan”, Israel.

#### † Melanopsis (Lyrcea) delessei

Taxon classificationAnimaliaSorbeoconchaMelanopsidae

Tournouër, 1875

##### Original source.

Tournouër 1875: 77.

##### Type horizon.

Tafi Formation, early Pleistocene.

##### Type locality.

“Prope Antimaki” [near Antimácheia, Kos Island], Greece.

#### † Melanopsis delicata

Taxon classificationAnimaliaSorbeoconchaMelanopsidae

Pallary, 1920

##### Original source.

Pallary 1920b: 112.

##### Type horizon.

Transdanubian, Pannonian, late Miocene.

##### Type locality.

“Radmanest” (Brusina 1902: captions of pl. 29) [Rădmănești], Romania.

##### Remarks.

Replacement name for *Melanopsis boettgeri* Brusina, 1902, non Klika, 1891. Jekelius (1944: 74) considered this species as a junior synonym of *Melanopsis sturii* Fuchs, 1873.

#### Melanopsis delmasi

Taxon classificationAnimaliaSorbeoconchaMelanopsidae

Pallary, 1936

##### Original source.

Pallary 1936: 55, pl. 3, fig. 3.

##### Type locality.

“Dans les canaux de l’Aguedal, à Marrakech” [in the channels of Aguedal in Marrakech], Morocco.

#### Melanopsis denegabilis

Taxon classificationAnimaliaSorbeoconchaMelanopsidae

Pallary, 1939

##### Original source.

Pallary 1939: 85, textfig. 11, pl. 6, figs 9–13.

##### Type locality.

“En Iraq; [...] à Rahalya Springs, à Kani Seip et à Karsi” [in Iraq, at Rahalya springs (?), at Kānī Seip (?) and at Karsī], Iraq.

##### Remarks.

Pallary attributed the authority to Férussac based on a manuscript name.

#### † Melanopsis deperdita

Taxon classificationAnimaliaSorbeoconchaMelanopsidae

de Serres, 1829

##### Original source.

De Serres 1829: 101.

##### Type horizon.

Early Campanian, Cretaceous.

##### Type locality.

“Martigues”, France.

#### † Melanopsis depereti

Taxon classificationAnimaliaSorbeoconchaMelanopsidae

Boistel, 1898

##### Original source.

Boistel 1898: 28 (footnote), figs 3A–D.

##### Type horizon.

Mammal zone MN 10–12, late Miocene.

##### Type locality.

“D’Ambronay [Vallon de Jurancieu]” [from Ambronay, in the valley of Jurancieu], France.

#### † Melanopsis depressa

Taxon classificationAnimaliaSorbeoconchaMelanopsidae

Pallary, 1916

##### Original source.

Pallary 1916: 77.

##### Type horizon.

Eocene?

##### Type locality.

“L’île de Wight” [Isle of Wight], United Kingdom.

##### Remarks.

Based on the record of “*Melanopsis buccinoidea* var. γ) antiqua; *elongata*” sensu Férussac, 1823 (pl. 7, fig. 6).

#### † Melanopsis bouei var. depressa

Taxon classificationAnimaliaSorbeoconchaMelanopsidae

Pallary, 1916 [invalid]

##### Original source.

Pallary 1916: 78.

##### Type horizon.

Pannonian, late Miocene.

##### Type locality.

“De la Moravie” (Férussac 1823: 164; no precise locality indicated), Czech Republic.

##### Remarks.

Based on a part of Férussac’s (1823: pl. 8, fig. 10) material of *Melanopsis bouei*. Homonym of the simultaneously published *Melanopsis depressa* Pallary, 1916, which has the higher rank in the species group and thus takes precedence (Art. 24.1).

#### † Lyrcaea [sic] narzolina var. dertocylindrica

Taxon classificationAnimaliaSorbeoconchaMelanopsidae

Sacco, 1895

##### Original source.

Sacco 1895: 14, pl. 1, fig. 25.

##### Type horizon.

Tortonian–early Messinian, late Miocene.

##### Type locality.

“S. Agata fossili” [Sant’Agata Fossili], Italy.

##### Remarks.

Harzhauser et al. (2015: 9) considered the taxon as a junior synonym of *Melanopsis narzolina* d’Archiac in Viquesnel, 1846.

#### † Lyrcaea [sic] pedemontana var. dertoliva

Taxon classificationAnimaliaSorbeoconchaMelanopsidae

Sacco, 1895

##### Original source.

Sacco 1895: 11, pl. 1, fig. 19.

##### Type horizon.

Tortonian–early Messinian, late Miocene.

##### Type locality.

“S. Agata fossili” [Sant’Agata Fossili], Italy.

##### Remarks.

The name “*dertolina*” as mentioned in Wenz (1929: 2802) is an incorrect subsequent spelling.

#### Melanopsis deserticola

Taxon classificationAnimaliaSorbeoconchaMelanopsidae

Annandale & Prashad, 1919

##### Original source.

Annandale and Prashad 1919: 37, pl. 3, fig. 8.

##### Type locality.

“Kaindak (long. 60°48'E., lat. 29°48'N.), Persian Baluchistan”, Iran.

##### Types.

Indian Museum, Calcutta, coll. no. 11535/2.

#### Melanopsis desertorum

Taxon classificationAnimaliaSorbeoconchaMelanopsidae

Bourguignat, 1884

##### Original source.

Bourguignat 1884: 134.

##### Type locality.

“Ruisseaux entre Tarsous et Mersina (Anatolie)” [streams between Tarsus and Mersin], Turkey.

##### Remarks.

Heller et al. (1999: 56) considered the species as a junior synonym of *Melanopsis buccinoidea* (Olivier, 1801). In Heller et al. (2005: 248) in turn it is treated as a junior synonym of *Melanopsis saulcyi* Bourguignat, 1853.

#### Melanopsis deshayesiana

Taxon classificationAnimaliaSorbeoconchaMelanopsidae

Gassies, 1861

##### Original source.

Gassies 1861: 292, pl. 7, fig. 12.

##### Type locality.

“La Nouvelle-Calédonie, dans l’intérieur” [inland of New Caledonia], New Caledonia.

#### † Melanopsis esperi var. desori

Taxon classificationAnimaliaSorbeoconchaMelanopsidae

De Stefani, 1877

##### Original source.

De Stefani 1877: 310, pl. 18, fig. 9.

##### Type horizon.

Villafranchian, Plio-Pleistocene.

##### Type locality.

“Spoleto”, Italy.

#### “ Melanella hollandri [sic] var. detrita

Taxon classificationAnimaliaSorbeoconchaMelanopsidae

Kucik” mentioned in Brusina (1867: 85) [unavailable]

##### Locality.

“U Savi kod Zagreba” [from the Sava river at Zagreb], Croatia.

##### Remarks.

Nomen nudum, based on an “in schedis” name in the collection of Kucik (also read as “Kutschig”). Brot (1874: 11) listed it in synonymy of *Melania holandri* [sic].

#### Melanopsis diabetensis

Taxon classificationAnimaliaSorbeoconchaMelanopsidae

Pallary, 1915

##### Original source.

Pallary 1915: 28.

##### Type locality.

“Dans la source du jardin du Sultan, à Diabet, près Mogador” [in the springs in the garden of the Sultan at Douar Dyabat, near Essaouira], Morocco.

#### Melanopsis sesteri var. diadema

Taxon classificationAnimaliaSorbeoconchaMelanopsidae

Bourguignat, 1884

##### Original source.

Bourguignat 1884: 119.

##### Type locality.

“Petit cours d’eau à Sadjour-Sou, entre Aïn-Taïb et Alep [...]; Aïn-el-Bass, dans la plaine du Bahr-el-Houlé (Syrie)” [small brook at Sadjour-Sou between Gaziantep (Turkey) and Aleppo (Syria) [...]; Aïn el Bass, in the plains of the Hula valley (Israel)].

#### † Melanopsis dianaeformis

Taxon classificationAnimaliaSorbeoconchaMelanopsidae

Andrusov, 1909

##### Original source.

Andrusov 1909: 84, 159, pl. 4, figs 21–27.

##### Type horizon.

Pontian (sensu stricto), late Miocene.

##### Type locality.

“Babadjan, Sundi, Meissary und Chilaalidasch” [Babadzhan, Syundi, Meysary canyon, Mount Chila-alidasch], Azerbaijan.

#### † Melanopsis dichtli

Taxon classificationAnimaliaSorbeoconchaMelanopsidae

Handmann, 1882

##### Original source.

Handmann 1882: 555.

##### Type horizon.

Pannonian, zone D, late Miocene.

##### Type locality.

“Kottingbrunn”, Austria.

#### Melanella letourneuxi var. dilatata

Taxon classificationAnimaliaSorbeoconchaMelanopsidae

Bourguignat, 1884

##### Original source.

Bourguignat 1884: 26.

##### Type locality.

“Ogulin”, Croatia.

#### Melanopsis buccinoidea var. dilatata

Taxon classificationAnimaliaSorbeoconchaMelanopsidae

Pallary, 1899

##### Original source.

Pallary 1899: 138, pl. 9, fig. 10.

##### Type locality.

“Dans la Souani, à Tanger” [in Souani at Tanger], Morocco.

#### Melanopsis dircaeana

Taxon classificationAnimaliaSorbeoconchaMelanopsidae

Pallary, 1939

##### Original source.

Pallary 1939: 87, pl. 6, figs 31–35.

##### Type locality.

Unclear: given as “Dans l’Oronte” [in the Orontes river] in text but as “Du lac de Homs” [Lake Homs (through which the Orontes flows)] in plate captions, Syria.

##### Remarks.

The name “*dircaena*” as mentioned in Heller et al. (2005: 238) is an incorrect subsequent spelling.

#### † Melanopsis dispar

Taxon classificationAnimaliaSorbeoconchaMelanopsidae

Deshayes, 1862

##### Original source.

Deshayes 1861–1864: 473, pl. 31, figs 29–30.

##### Type horizon.

Lutetian, Eocene.

##### Type locality.

“Brasles”, France.

##### Remarks.

Cossmann (1888: 279) and Wenz (1929: 2639) classified the species in the genus *Faunus* Montfort, 1810 (Pachychilidae).

#### † Melanopsis dissimilis

Taxon classificationAnimaliaSorbeoconchaMelanopsidae

Pallary, 1916

##### Original source.

Pallary 1916: 85.

##### Type horizon.

Langhian, middle Miocene.

##### Type locality.

“Ribarić” (Neumayr 1869: 358), Croatia.

##### Remarks.

Replacement name for *Melanopsis lyrata* Neumayr, 1869 [June], non *Melanopsis lirata* Gassies, 1869 [January]. Both names are deemed to be identical after Art. 58.2.

#### Melanella divina

Taxon classificationAnimaliaSorbeoconchaMelanopsidae

Bourguignat, 1884

##### Original source.

Bourguignat 1884: 11.

##### Type locality.

“Mare du moulin de la Cettina, près Almissa, en Dalmatie” [millpond at the river Cetina, near Omiš], Croatia.

##### Remarks.

Bourguignat denoted the authority as “Letourneux, 1879”, but there is no evidence that the description really derived from that author.

#### Fagotia (Dneprifagotia) dneprensis

Taxon classificationAnimaliaSorbeoconchaMelanopsidae

Starobogatov, Alexenko & Levina, 1992

##### Original source.

Starobogatov et al. 1992: 63, figs 1 (2), 2 (4), 3 (11).

##### Type locality.

“Из Днепра у Херсона” [from the Dniepr river at Kherson], Ukraine.

##### Types.

Zoological Institute of Russian Academy of Sciences, St.-Petersburg; no number indicated.

#### † Melanopsis doboi

Taxon classificationAnimaliaSorbeoconchaMelanopsidae

Schréter, 1975

##### Original source.

Schréter 1975: 7, textfig. 1, pl. 1, figs 3–6.

##### Type horizon.

Riss/Würm end to early Würm Ice Age, Pleistocene.

##### Type locality.

“Eger, az egri vár Zárkándy bástyájának átmetszése” [Eger, section at the Zarkandy bastion of the fortress Eger], Hungary.

##### Types.

Magyar Állami Földtani Intézet (Hungarian Geological Museum), Budapest; no number indicated.

##### Remarks.

Neubauer et al. (2016a) attributed the species to the genus *Microcolpia*.

#### † Melanopsis matheroni var. doderleini

Taxon classificationAnimaliaSorbeoconchaMelanopsidae

Pantanelli, 1886

##### Original source.

Pantanelli 1886a: 69 or 1886b: 78, pl. 3, figs 5, 8 (precedence not established).

##### Type horizon.

Tortonian–early Messinian, late Miocene.

##### Type locality.

“S. Valentino, S. Agata, Boggione” [San Valentino, Sant’Agata Fossili, Boggione near Siena], Italy.

##### Remarks.

Harzhauser et al. (2015: 9) considered the taxon as a junior synonym of *Melanopsis narzolina* d’Archiac in Viquesnel, 1846.

#### Melanopsis (Mesopotamia) khabourensis var. dolichosoma

Taxon classificationAnimaliaSorbeoconchaMelanopsidae

Pallary, 1939

##### Original source.

Pallary 1939: 103, pl. 5, fig. 18.

##### Type locality.

“Ras el ‘Ain du Khabour” [Chabur river near Ra’s al ‘Ayn], Syria.

#### † “ Melanopsis doliolum

Taxon classificationAnimaliaSorbeoconchaMelanopsidae

” mentioned in Graves (1847: 600) [unavailable]

##### Horizon.

Eocene.

##### Locality.

“Cuise-Lamotte, Jaulzy, Tiverny, Saint-Vaast-de-Longmont”, France.

##### Remarks.

Nomen nudum. Graves attributed the authority to Defrance.

#### † Melanopsis (Canthidomus) bouei var. doliolum

Taxon classificationAnimaliaSorbeoconchaMelanopsidae

Handmann, 1887

##### Original source.

Handmann 1887: 35, pl. 7, figs 6–7.

##### Type horizon.

Pannonian, zone B–D, late Miocene.

##### Type locality.

“Leobersdorf”, Austria.

#### † Melanopsis doncieuxi

Taxon classificationAnimaliaSorbeoconchaMelanopsidae

Pallary, 1916

##### Original source.

Pallary 1916: 80.

##### Type horizon.

Lutetian, Eocene.

##### Type locality.

“Au Nord d’Albas” (Doncieux 1908: 204), France.

##### Remarks.

Replacement name for *Melanopsis nodosa* Doncieux, 1908, non Férussac, 1822.

#### † Melanopsis (Stylospirula) doncieuxi

Taxon classificationAnimaliaSorbeoconchaMelanopsidae

Wenz, 1919 [invalid]

##### Original source.

Wenz 1919b: 65.

##### Type horizon.

Middle Lutetian, Eocene.

##### Type locality.

“Au Nord d’Albas” (Doncieux 1908: 204), France.

##### Remarks.

Replacement name for *Melanopsis brevis* Doncieux, 1908, non Sowerby, 1826. For that homonym, Pallary (1916) had introduced the replacement name *Melanopsis abbreviata*, which is a junior homonym of *Melanopsis abbreviata* Brusina, 1874. (Note that Wenz 1919 was unaware of that name.) *Melanopsis doncieuxi* Wenz, 1919 is, however, invalid too it is a junior homonym of *Melanopsis doncieuxi* Pallary, 1916 (see *Melanopsis atacica* Wenz, 1928).

#### Melanopsis doriae

Taxon classificationAnimaliaSorbeoconchaMelanopsidae

Issel, 1865

##### Original source.

Issel 1865 [page unknown; original work not seen; described on p. 400 of the re-published version from 1866].

##### Type locality.

“Di Kerman nella Persia meridionale” [Kerman], Iran.

##### Remarks.

Considered a junior synonym of *Melanopsis ammonis* Tristram, 1865 by Heller et al. (2005). Considered a synonym of *Melanopsis variabilis* v.d. Busch in Philippi, 1847 by Martens (1874).

#### † Melanopsis (Macrospira) doroghensis

Taxon classificationAnimaliaSorbeoconchaMelanopsidae

Oppenheim, 1892

##### Original source.

Oppenheim 1892: 705, pl. 33, figs 7–11.

##### Type horizon.

Paleocene.

##### Type locality.

“Dorogh, Annathal, Nagy Kovacsi” [Dorog, Annavölgy, Nagykovácsi], Hungary.

#### Melanopsis dos

Taxon classificationAnimaliaSorbeoconchaMelanopsidae

Preston, 1913

##### Original source.

Preston 1913: 436.

##### Type locality.

“Island of Beilan-Beilan, to the north of the Obi Islands, Dutch East Indies” [Belangbelang Island], Indonesia.

##### Remarks.

Probably not a Melanopsidae.

#### † Melanopsis (Smendovia) doumerguei

Taxon classificationAnimaliaSorbeoconchaMelanopsidae

Pallary, 1901

##### Original source.

Pallary 1901a: 177, pl. 2, fig. 24.

##### Type horizon.

Late Miocene.

##### Type locality.

“Smendou” [Zighoud Youcef], Algeria.

#### Melanopsis doumeti

Taxon classificationAnimaliaSorbeoconchaMelanopsidae

Letourneux & Bourguignat, 1887

##### Original source.

Letourneux and Bourguignat 1887: 157.

##### Type locality.

“De Gafsa; [...] de Tozer et de Nefta” [from Gafsa; in Tozeur and Nafta], Tunisia.

#### Melanopsis douttei

Taxon classificationAnimaliaSorbeoconchaMelanopsidae

Pallary, 1911

##### Original source.

Pallary 1911: 133, [unnumbered plate], figs 21–22.

##### Type locality.

“Fez” [Fes], Morocco.

##### Remarks.

Given as “*doutte*” on p. 133, but as “*douttei*” in plate caption. Since Pallary explicitly named the species after E. Doutté, the name must read “*douttei*” (Art. 32.5.1).

#### † Melanopsis douvillei

Taxon classificationAnimaliaSorbeoconchaMelanopsidae

Pallary, 1916

##### Original source.

Pallary 1916: 83.

##### Type horizon.

Maastrichtian, Cretaceous.

##### Type locality.

“Du versant oriental du Kouh Mapeul” (Douvillé 1904: captions of pl. 46) [eastern slope of mount Kuh-e Mapel, c. 60 km WNW of Khorramābād], Iran.

##### Remarks.

Replacement name for *Melanopsis costellata* Douvillé, 1904, non Férussac, 1823.

#### † Melanopsis draghiceniani

Taxon classificationAnimaliaSorbeoconchaMelanopsidae

Cobălcescu, 1883

##### Original source.

Cobălcescu 1883: 124, pl. 9, figs 9a–b.

##### Type horizon.

Dacian, Pliocene.

##### Type locality.

“De Bécéni” [Beceni], Romania.

##### Remarks.

The name “*draghiceniana*” as mentioned in Wenz (1929: 2709) is an incorrect subsequent spelling.

#### Melanopsis (Sistaniana) drangianensis

Taxon classificationAnimaliaSorbeoconchaMelanopsidae

Starobogatov & Izzatullaev, 1985

##### Original source.

Starobogatov and Izzatullaev 1985: 34, fig. 4.

##### Type locality.

“Систан” [Sistan region], Iran.

##### Types.

Zoological Institute of Russian Academy of Sciences, St.-Petersburg; no number indicated.

#### † Melanopsis dubia

Taxon classificationAnimaliaSorbeoconchaMelanopsidae

Stoliczka, 1860

##### Original source.

Stoliczka 1860: 486, pl. 1, figs 14, 15a–b.

##### Type horizon.

Late Turonian, late Cretaceous.

##### Type locality.

“Neualpe im Russbachthal” [Neualm near Russbach am Pass Gschütt], Austria.

#### † Melanopsis dubiosa

Taxon classificationAnimaliaSorbeoconchaMelanopsidae

Matheron, 1879

##### Original source.

Matheron 1878–1880: pl. O1, fig. 14.

##### Type horizon.

Cuisian, late Ypresian, early Eocene.

##### Type locality.

“Brignan, couches inférieures du Montaiguet et de Grabels; lignite de la Caunette” (it is unclear if this statement describes a single locality or several and, if so, from which the species derives), France.

#### † Melanopsis (Canthidomus) dubiosa

Taxon classificationAnimaliaSorbeoconchaMelanopsidae

Kühn, 1963 [invalid]

##### Original source.

Kühn 1963: 387, pl. 3, figs 12–13.

##### Type horizon.

Mammal zone MN 9–10, late Miocene.

##### Type locality.

“Daphni” [Dafní valley near Athens], Greece.

##### Types.

Museum of Palaeontology and Geology of the University of Athens, coll. no. 1963/84.

##### Remarks.

Junior homonym of *Melanopsis dubiosa* Matheron, 1878. Papp (1979: 668) considered the species as a junior synonym of *Melanopsis longa* Deshayes in Férussac, 1839.

#### Melanopsis ducosi

Taxon classificationAnimaliaSorbeoconchaMelanopsidae

Pallary, 1916

##### Original source.

Pallary 1916: 82.

##### Type locality.

“Dans le Diahot, à Balade; à Jengen, à Kanala, dans les marais et les petits ruisseaux” (Gassies 1861: 289) [in the Diahot river at Balade; at Hienghène, at Canala, in swamps and small streams], New Caledonia.

##### Remarks.

Replacement name for *Melanopsis carinata* Gassies, 1861, non Sowerby, 1826.

#### † Melanopsis dufourii

Taxon classificationAnimaliaSorbeoconchaMelanopsidae

Férussac, 1822

##### Original source.

Férussac 1819–1851: Mélanopsides fossiles, pl. 1 (1822), fig. 16.

##### Type horizon.

Burdigalian, early Miocene (?).

##### Type locality.

“Des environs de Dax” [surroundings of Dax], France.

##### Remarks.

The name first appeared in 1822 on the captions for plate 1 of the “Mélanopsides fossiles” in Férussac’s “Histoire naturelle” (see also introduction for details). While Férussac (1823) included also recent specimens under that name in his monograph on the Melanopsidae, the only specimen illustrated in 1822 in the “Histoire naturelle” was a fossil one from the Miocene of France. This fact remained widely unknown to biologists and paleontologists alike. Only few malacologists, such as Grateloup (1828, 1838, 1840), used the name correctly and attributed it to the French fossils. Consequently, none of the specimens referred to as *Melanopsis dufourii* in the biological literature actually corresponds to the real *Melanopsis dufourii*. Very likely, some of the other melanopsids from Dax (see, e.g., Grateloup 1838, d’Orbigny 1852, Pallary 1916) are unrecognized junior synonyms of this species.

The names “*dufouri*” as mentioned by numerous authors and databases and “*dufourei*” as given by Morelet (1853: 297) and Bourguignat (1884: 114) are incorrect subsequent spellings.

#### † Melanopsis dufresnii

Taxon classificationAnimaliaSorbeoconchaMelanopsidae

Deshayes, 1825

##### Original source.

Deshayes 1824–1837: 120, pl. 12, figs 3–4.

##### Type horizon.

Sparnacian, early Ypresian, Eocene.

##### Type locality.

“Les environs de Soissons” [surroundings of Soisson], France.

##### Remarks.

Cossmann (1888: 280) classified the species in the genus *Faunus* Montfort, 1810, Wenz (1929: 2623) affiliated it with *Melanatria* Bowdich, 1822 (both Pachychilidae). Note that both authors gave the name as “*dufresnei*”, which is an incorrect subsequent spelling. Moreover, *Melanatria* Bowdich, 1822 [February] is not an available name because it is a replacement name for the vernacular “Pyrene Lamarck” which was not available in nomenclature before April 1822.

#### Melanopsis dumbeensis

Taxon classificationAnimaliaSorbeoconchaMelanopsidae

Crosse, 1869

##### Original source.

Crosse 1869a: 70 (Latin) [January].

##### Type locality.

“Dumbea”, New Caledonia.

##### Remarks.

Re-described in French by Crosse (1869b: 281, pl. 8, fig. 4) [October].

#### † Melanopsis nodosa var. duonodulosa

Taxon classificationAnimaliaSorbeoconchaMelanopsidae

Ippolito, 1947

##### Original source.

Ippolito 1947: [p. 4 of the Separatum], [unnumbered plate], figs 32–35.

##### Type horizon.

Late Villafranchian, early Pleistocene.

##### Type locality.

“Colle dell’Oro presso Terni”, Italy.

#### † Melanopsis bouei var. duplicata

Taxon classificationAnimaliaSorbeoconchaMelanopsidae

Handmann, 1882

##### Original source.

Handmann 1882: 557.

##### Type horizon.

Pannonian, zone D, late Miocene.

##### Type locality.

“Kottingbrunn [...] Ziegelei a”, Austria.

##### Remarks.

The taxon is not included in the Fossilium Catalogus of Wenz (1929).

#### † Melanopsis dutemplei

Taxon classificationAnimaliaSorbeoconchaMelanopsidae

Deshayes, 1862

##### Original source.

Deshayes 1861–1864: 427, pl. 21, fig. 31.

##### Type horizon.

Sparnacian, early Ypresian, Eocene.

##### Type locality.

“Saran, Cramant, Damery”, France.

##### Remarks.

Wenz (1929: 2637) considered the taxon as a junior synonym of *Faunus cerithiformis* (Watelet, 1851) (Pachychilidae).

#### Melanopsis duveyrieri

Taxon classificationAnimaliaSorbeoconchaMelanopsidae

Bourguignat in Letourneux & Bourguignat, 1887

##### Original source.

Letourneux and Bourguignat 1887: 160.

##### Type locality.

“El-Hammam, près Tozer” [El-Hamma-Djerid near Tozeur], Tunisia.

#### † Melanopsis matheroni var. ecarinata

Taxon classificationAnimaliaSorbeoconchaMelanopsidae

Fontannes, 1880

##### Original source.

Fontannes 1879–1882: 176, pl. 10, fig. 3.

##### Type horizon.

Miocene or Pliocene.

##### Type locality.

Rhône Basin (no exact locality given), France.

##### Remarks.

Based on material from the Rhône Basin, not Italy as claimed by Wenz (1929); probably the taxon was mixed up by Italian authors cited by Wenz (1929). Harzhauser et al. (2015: 9) considered the taxon as a junior synonym of *Melanopsis narzolina* d’Archiac in Viquesnel, 1846.

#### Melanopsis edrissiana

Taxon classificationAnimaliaSorbeoconchaMelanopsidae

Pallary, 1918

##### Original source.

Pallary 1918: 151.

##### Type locality.

“Ras el Mâ et Fès” [Ras el Ma], Morocco.

#### Melanopsis egregia

Taxon classificationAnimaliaSorbeoconchaMelanopsidae

Bourguignat, 1884

##### Original source.

Bourguignat 1884: 146.

##### Type locality.

“Dans le Jourdain; [...] dans le Belus près de Saint-Jean-d’Acre” [in the Jordan river; in the Na’aman river, near Acre], Israel.

##### Remarks.

Heller et al. (2005: 247) considered the species as a junior synonym of *Melanopsis obliqua* Bourguignat, 1884.

#### Melanopsis dufourii var. elata

Taxon classificationAnimaliaSorbeoconchaMelanopsidae

Issel, 1866

##### Original source.

Issel 1866: 33.

##### Type locality.

“Nella Maremma Toscana in un fiumicello d’acqua calda detto Caldana di Ravi, presso Campiglia” [in the Tuscan Maremma, in a stream of warm water called Caldana di Ravi, at Campiglia Marittima], Italy.

#### Melanopsis graellsii var. elatior

Taxon classificationAnimaliaSorbeoconchaMelanopsidae

Pallary, 1924

##### Original source.

Pallary 1924: 250.

##### Type locality.

“Gandia (Valencia)”, Spain.

##### Remarks.

Not available from Rossmässler (1854), to whom Pallary referred, because Rossmässler did not use the term “*elatior*” to denote a species-group taxon but only cited Férussac’s (1823) description of an unnamed variety of *Melanopsis dufourii*. Pallary briefly described the taxon (by indicating its size) and therefore made the name available.

#### Melanopsis elegans

Taxon classificationAnimaliaSorbeoconchaMelanopsidae

Gassies, 1869

##### Original source.

Gassies 1869: 76.

##### Type locality.

“Noumea”, New Caledonia.

#### “ Melanella elegans

Taxon classificationAnimaliaSorbeoconchaMelanopsidae

” mentioned in Bourguignat (1884: 15) [unavailable]

##### Locality.

“De la Save et de la Savina; [...] du Danube, près de Belgrade (Serbie), de Zenica (Bosnie), de la rivière d’Ostaria et d’un ruisseau sur là route de Pregrada, non loin de Krapina-Toeplitz, en Croatie; [...] au pont de la Save, près d’Agram” [in the Sava and Savinja rivers (Croatia, Slovenia); [...] Danube river near Belgrade (Serbia), Zenica (Bosnia and Herzegovina), in the river at Oštarije and a stream at the road to Pregrada, near Krapinske toplice (Croatia); [...] at the Sava bridge near Zagreb].

##### Remarks.

The name “Var. *elegans*” as mentioned by Rossmässler (1839) is a nomen nudum, referring to the personal opinion of Schmidt, with whom Rossmässler collected the material. If available, it would be a junior objective synonym of *Melanopsis holandrii legitima* Rossmässler, 1839, under which “*elegans*” was listed as a synonym by Rossmässler.

#### † Melanopsis eleis

Taxon classificationAnimaliaSorbeoconchaMelanopsidae

Oppenheim, 1891

##### Original source.

Oppenheim 1891: 465, pl. 26, fig. 5.

##### Type horizon.

Pliocene.

##### Type locality.

“Bizerè, nördlich von Pyrgos (Elis)” [Bizeré, north of Pyrgos], Greece.

#### Melanopsis orontis var. elevata

Taxon classificationAnimaliaSorbeoconchaMelanopsidae

Pallary, 1939 [unavailable]

##### Original source.

Pallary 1939: 92, pl. 6, fig. 74.

##### Type locality.

Unclear: given as “L’Oronte à Djishr ech Chegour” [Orontes river at Jisr Ash-Shughur] in text but as “L’Oronte à Homs” [Orontes river at Homs (which is far to the south of the former)] in plate captions, Syria.

##### Remarks.

Unavailable according to Art. 13.1, because it lacks a verbal description or definition. Heller et al. (2005: 244) considered it as a junior synonym of *Melanopsis costata* (Olivier, 1804).

#### † Melanopsis impressa var. elliptica

Taxon classificationAnimaliaSorbeoconchaMelanopsidae

Handmann, 1887

##### Original source.

Handmann 1887: 23, pl. 3, fig, 9.

##### Type horizon.

Pannonian, zone B–D, late Miocene.

##### Type locality.

“Leobersdorf”, Austria.

#### † Melanopsis buccinoidea var. γ) antiquua elongata

Taxon classificationAnimaliaSorbeoconchaMelanopsidae

Férussac, 1822

##### Original source.

Férussac 1819–1851: Mélanopsides fossiles, pl. 1 (1822), figs 5–7.

##### Type horizon.

Eocene.

##### Type locality.

“Des environs d’Epernay. [...] De l’île de Wight” [surroundings of Épernay (France); Isle of Wight (United Kingdom)].

##### Remarks.

Introduced within the var. γ, to which Férussac attributed the term “antiquua”, which was probably not intended as species-group name (see introduction for a detailed discussion of the names introduced by Férussac 1823). Note that for figs 6 and 7 of pl. 7 in Férussac (1823), which is the same as pl. 1 of the “Mélanopsides fossiles” in Férussac (1819–1851), Pallary (1916) introduced two new names, i.e., *Melanopsis depressa* and *Melanopsis sparnacensis*.

#### Melanopsis elongata

Taxon classificationAnimaliaSorbeoconchaMelanopsidae

Gassies, 1874 [invalid]

##### Original source.

Gassies 1874: 384.

##### Type locality.

“Bourail”, New Caledonia.

##### Remarks.

Junior homonym of *Melanopsis buccinoidea elongata* Férussac, 1822. Pallary (1916: 82) introduced *Melanopsis goulvaini* as replacement name for the junior homonym.

#### † Melanopsis narzolina var. elongata

Taxon classificationAnimaliaSorbeoconchaMelanopsidae

Locard, 1878 [invalid]

##### Original source.

Locard 1878: 58.

##### Type horizon.

Tortonian, late Miocene.

##### Type locality.

“À Tersannes près de Hauterives (Drôme)” [at Tersanne near Hauterives], France.

##### Remarks.

Junior homonym of *Melanopsis buccinoidea elongata* Férussac, 1822. Wenz (1929: 2785) considered the taxon as a junior synonym of *Melanopsis narzolina* d’Archiac in Viquesnel, 1846.

#### † Melanopsis bouei var. elongata

Taxon classificationAnimaliaSorbeoconchaMelanopsidae

Handmann, 1882 [invalid]

##### Original source.

Handmann 1882: 557.

##### Type horizon.

Pannonian, zone D, late Miocene.

##### Type locality.

“Kottingbrunn [...] Ziegelei a”, Austria.

##### Remarks.

Junior homonym of *Melanopsis buccinoidea elongata* Férussac, 1822. Not included in the Fossilium Catalogus by Wenz (1929).

#### Melanella letourneuxi var. elongata

Taxon classificationAnimaliaSorbeoconchaMelanopsidae

Bourguignat, 1884

##### Original source.

Bourguignat 1884: 26.

##### Type locality.

“Ogulin”, Croatia.

#### † Melanopsis (Martinia) vindobonensis var. elongata

Taxon classificationAnimaliaSorbeoconchaMelanopsidae

Handmann, 1887 [invalid]

##### Original source.

Handmann 1887: 28, pl. 6, figs 3–4.

##### Type horizon.

Pannonian, zone B–D, late Miocene.

##### Type locality.

“Leobersdorf”, Austria.

##### Remarks.

Junior homonym of *Melanopsis buccinoidea elongata* Férussac, 1822.

#### † Melanopsis citharella var. elongata

Taxon classificationAnimaliaSorbeoconchaMelanopsidae

Locard, 1893 [invalid]

##### Original source.

Locard 1893: 178, pl. 9, fig. 17.

##### Type horizon.

“Helicidenmergel”, late Burdigalian, early Miocene.

##### Type locality.

“Uetken, en Argovie”, Switzerland.

##### Remarks.

Junior homonym of *Melanopsis buccinoidea elongata* Férussac, 1822. Neubauer et al. (2014c: 29) considered this taxon as a junior synonym of *Melanopsis citharella* Merian, 1849.

#### † Melanopsis hazayi var. elongata

Taxon classificationAnimaliaSorbeoconchaMelanopsidae

Brusina, 1903 [invalid]

##### Original source.

Brusina 1903: 112.

##### Type horizon.

Late Pleistocene–early Holocene.

##### Type locality.

“Bischofsbad” [Püspökfürdő, Băile 1 Mai, Lake Pețea], Romania.

##### Remarks.

Junior homonym of *Melanopsis buccinoidea elongata* Férussac, 1822. Neubauer et al. (2014d: 125) considered this taxon as a junior synonym of *Microcolpia parreyssii sikorai* (Brusina, 1903).

#### † Melanopsis elongata

Taxon classificationAnimaliaSorbeoconchaMelanopsidae

Doncieux, 1908 [invalid]

##### Original source.

Doncieux 1908: 202, pl. 11, fig. 10.

##### Type horizon.

Middle Lutetian, Eocene.

##### Type locality.

“Au Nord d’Albas”, France.

##### Remarks.

Junior homonym of *Melanopsis buccinoidea elongata* Férussac, 1822. Pallary (1916: 82) introduced *Melanopsis sublongata* [sic] as replacement name.

#### Melanopsis (Mesopotamia) khabourensis var. elongata

Taxon classificationAnimaliaSorbeoconchaMelanopsidae

Pallary, 1939 [invalid]

##### Original source.

Pallary 1939: 103, pl. 5, fig. 16.

##### Type locality.

“Ras el ‘Ain du Khabour” [Chabur river near Ra’s al ‘Ayn], Syria.

##### Remarks.

Junior homonym of *Melanopsis buccinoidea elongata* Férussac, 1822. Heller et al. (2005: 239) tentatively considered the variety as a junior synonym of *Melanopsis khabourensis* Pallary, 1939.

#### “ Melanopsis praemorsa f. elongata

Taxon classificationAnimaliaSorbeoconchaMelanopsidae

” mentioned in Pérès (1939) [unavailable]

##### Locality.

Collection stations 80 [tributary rivulet to the river Tensift] and 144 [Oued Sidi Raho, approximately 6 km upstream of its confluence with the Bou Regreg] (Pérès 1939: 139), Morocco.

##### Remarks.

First of all, the name as given by Pérès (1939) is a nomen nudum – Pérès apparently considered the expression “*elongata*” self-explanatory and did not describe it. Moreover, he obviously used the name not as separate taxon but rather as descriptive term to fit existing species into his morphological concept. He even indicated *Melanopsis olivieri* Bourguignat, 1884 as its “type”.

#### † Melanopsis (Canthidomus) defensa elongata

Taxon classificationAnimaliaSorbeoconchaMelanopsidae

Gillet & Marinescu, 1971 [invalid]

##### Original source.

Gillet and Marinescu 1971: 55, pl. 23, figs 38–48.

##### Type horizon.

Transdanubian, Pannonian, late Miocene.

##### Type locality.

“Rădmănești”, Romania.

##### Remarks.

Junior homonym of *Melanopsis buccinoidea elongata* Férussac, 1822. Neubauer et al. (2014c: 31) considered this variety a junior synonym of *Melanopsis defensa* Fuchs, 1870.

#### † Melanopsis elophila

Taxon classificationAnimaliaSorbeoconchaMelanopsidae

Pallary, 1925

##### Original source.

Pallary 1925: 257.

##### Type horizon.

Messinian, late Miocene.

##### Type locality.

“Della valle della Sterza” (Capellini 1880: 420) [La Sterza near Lajatico], Italy.

##### Remarks.

Introduced for *Melania buccinoidea* sensu Capellini, 1880, non Férussac, 1823.

#### “ Melanopsis elysaea

Taxon classificationAnimaliaSorbeoconchaMelanopsidae

” mentioned in Brot (1874–1879: 419) [unavailable]

##### Locality.

Not indicated.

##### Remarks.

Nomen nudum, “in schedis” name from Tarnier listed in the synonymy list of *Melania buccinoidea* (Olivier, 1801) by Brot (1879).

#### Melanopsis foleyi var. emaciata

Taxon classificationAnimaliaSorbeoconchaMelanopsidae

Pallary, 1928 [unresolved]

##### Original source.

Pallary 1928a: 271.

##### Type locality.

“Aïn Mélias, près de Figuig” [Ain Melias near Figuig], Algeria.

##### Remarks.

Homonym of the simultaneously published name *Melanopsis letourneuxi emaciata* Pallary, 1928 (see Note 1). This case requires the action of a First Reviser.

#### Melanopsis letourneuxi var. emaciata

Taxon classificationAnimaliaSorbeoconchaMelanopsidae

Pallary, 1928 [unresolved]

##### Original source.

Pallary 1928a: 273, pl. 5, fig. 7.

##### Type locality.

“Berguent” [Aïn Beni Mathar], Morocco.

##### Remarks.

Homonym of the simultaneously published name *Melanopsis foleyi emaciata* Pallary, 1928 (see Note 1). This case requires the action of a First Reviser.

#### Melanopsis monteli var. emaciata

Taxon classificationAnimaliaSorbeoconchaMelanopsidae

Pallary, 1936 [invalid]

##### Original source.

Pallary 1936: 61.

##### Type locality.

Not explicitly stated but probably the same as for the species (“L’Oued Sous, au pont des Aït Melloul, sur la route d’Agadir à Tiznit, à 14 kil. S. O. d’Agadir” [in the Oued Sous, at the bridge of Ait Melloul, at the road from Agadir to Tiznit, 14 km southwest of Agadir], Morocco).

##### Remarks.

Junior homonym of *Melanopsis letourneuxi emaciata* Pallary, 1928 and *Melanopsis foleyi emaciata* Pallary, 1928 (simultaneously published; no priority fixed yet; see Note 1).

#### Melanopsis cerithiopsis var. emaciata

Taxon classificationAnimaliaSorbeoconchaMelanopsidae

Pallary, 1939 [invalid]

##### Original source.

Pallary 1939: 96, pl. 6, figs 40–43.

##### Type locality.

“À Jéricho, dans ‘Ain Solthan” [at Jericho, in the spring ‘Ayn as Sulţān], Palestine.

##### Remarks.

Junior homonym of *Melanopsis letourneuxi emaciata* Pallary, 1928 and *Melanopsis foleyi emaciata* Pallary, 1928 (simultaneously published; no priority fixed yet; see Note 1).

#### † Melanopsis emerici

Taxon classificationAnimaliaSorbeoconchaMelanopsidae

d’Orbigny, 1850

##### Original source.

d’Orbigny 1850: 309.

##### Type horizon.

Early Rupelian, Oligocene.

##### Type locality.

“Levit, près de Castellanne” [Vît-de-Castellane near Castellane], France.

#### † Melanopsis geniculata f. enodata

Taxon classificationAnimaliaSorbeoconchaMelanopsidae

Brusina, 1897

##### Original source.

Brusina 1897: 11.

##### Type horizon.

Early Langhian, middle Miocene.

##### Type locality.

“Sinj (Župića potok)”, Croatia.

##### Types.

The syntypes are stored in the Croatian Natural History Museum, Zagreb; no number indicated (Milan et al. 1974: 91).

##### Remarks.

Wenz (1929: 2734) considered the taxon as a junior synonym of *Melanopsis geniculata* Brusina, 1874, while Neubauer et al. (2016b: 23) treated it as a distinct species. The name “*enodota*” as mentioned in Wenz (1929: 2734) is an incorrect subsequent spelling.

#### “ Melania enodes

Taxon classificationAnimaliaSorbeoconchaMelanopsidae

Ziegler” mentioned in Reeve (1860) [unavailable]

##### Locality.

Not indicated.

##### Remarks.

Perhaps a manuscript or “in schedis” name from Ziegler. Reeve (1860) considered this taxon as a junior synonym of *Melania hollandrii* [sic].

#### † Melanopsis entzi

Taxon classificationAnimaliaSorbeoconchaMelanopsidae

Brusina, 1902

##### Original source.

Brusina 1902: pl. 6, figs 34–37.

##### Type horizon.

Transdanubian, Pannonian, late Miocene.

##### Type locality.

“Tihany; Kenese” [Tihany; Balatonkenese], Hungary.

##### Types.

Milan et al. (1974: 90) defined a “lectosyntypus”, a term not accepted by the Code, based on the specimen from Tihany illustrated by Brusina (1902: pl. 6, figs 34–36). This fixation is insufficient for a valid lectotype designation (Art. 74). The specimen is stored in the Croatian Natural History Museum, Zagreb, coll. no. 2515-161/1-2.

##### Remarks.

Bartha (1955: 294) considered this taxon as a junior synonym of *Melanopsis fuchsi* Handmann, 1882.

#### † Melanopsis eocenica

Taxon classificationAnimaliaSorbeoconchaMelanopsidae

Pallary, 1916

##### Original source.

Pallary 1916: 79.

##### Type horizon.

Sparnacian, early Ypresian, Eocene.

##### Type locality.

“Mont Bernon bei Epernay” (only this locality is the type locality as Pallary clearly referred to the specimen illustrated by Sandberger 1870–1875: pl. 9, fig. 5), France.

##### Remarks.

Introduced for *Melania buccinoidea* sensu Sandberger, 1870. Wenz (1929: 2660) considered that record a junior synonym of *Melanopsis antidiluviana* (Poiret, 1801). The name “*eocaenica*” as mentioned in Wenz (1929: 2662) is an incorrect subsequent spelling.

#### Melanopsis episema

Taxon classificationAnimaliaSorbeoconchaMelanopsidae

Bourguignat, 1884

##### Original source.

Bourguignat 1884: 88.

##### Type locality.

“Près de Biskra, dans un ruisseau d’eau chaude à Ouargla, dans un puits artésien de Mazer au Ziban, ainsi qu’aux environs de Boghar et dans les rivières du djebel Takreda entre Mogador et Maroc [sic]” [near Biskra, in a stream of warm water at Ouargla, in an artesian well of Mazer, as well as around Boghar (Algeria) and in rivers of Jebel Takreda (?) between Essaouira and Morocco (?)].

#### Melanopsis eremita

Taxon classificationAnimaliaSorbeoconchaMelanopsidae

Tristram, 1865

##### Original source.

Tristram 1865: 542.

##### Type locality.

“Wady Um Bagkek, between Sebbeh and Jebel Usdum, at the south-west corner of the Dead Sea”, Israel.

##### Remarks.

Heller et al. (2005: 236) considered the species as a junior synonym of *Melanopsis ammonis* Tristram, 1865.

#### Melanopsis laevigata var. erosa

Taxon classificationAnimaliaSorbeoconchaMelanopsidae

Roth, 1839

##### Original source.

Roth 1839: 24.

##### Type locality.

“In Peloponneso”, Greece.

#### Melanopsis esperi

Taxon classificationAnimaliaSorbeoconchaMelanopsidae

Férussac, 1823

##### Original source.

Férussac 1823: 160.

##### Type locality.

“La Laybach” [Ljubljanica river], Slovenia.

##### Remarks.

Type species of *Esperiana* Bourguignat, 1877.

#### † Melanopsis esperioides

Taxon classificationAnimaliaSorbeoconchaMelanopsidae

Stefanescu, 1896

##### Original source.

Stefanescu 1896: 128, pl. 11, figs 6–11.

##### Type horizon.

Pliocene.

##### Type locality.

“À Bucovatz, dans la vallée de Jiu, et à Milcov, près de Slatina, dans la vallée de l’Oltu” [at Bucovăț, in the valley of the river Jiu, and at Milcov, near Slatina, in the valley of the river Olt], Romania.

##### Remarks.

The name “*esperiodea*” as mentioned in Huică (1977: 41) is an incorrect subsequent spelling.

#### Melanopsis lorcana var. etrusca

Taxon classificationAnimaliaSorbeoconchaMelanopsidae

Westerlund, 1886

##### Original source.

Westerlund 1886: 128.

##### Type locality.

“Italien, Balearen [Westerlund]; aus Toscana als auch aus Jumilla (Spanien) und Merknes (Marocco) [Brot]” [Italy, Balearic Islands (Spain); Tuscany (Italy), Jumilla (prov. Murcia, Spain), Merknes (Morocco)].

##### Remarks.

The name was first mentioned by Brot (1862: 63) as “*etrusca* Villa (v. min)” from the locality “Toscane”, without description or bibliographical reference. Brot listed it under the name “*Melanopsis Dufourii* Fér.”, for which a bibliographical reference (to “Rossm. Icon. 835–839”) and locality “Espagne” was given. Since *etrusca* was neither described by Brot nor by Rossmässler, the name *etrusca* is not available from this case (Art. 12.1).

Brot (1879: 434) listed the name (“*Melanopsis etrusca* Villa MSS.”) as synonym of *Melanopsis Dufourii* var. β, which he introduced there with a short Latin description. On p. 435 Brot even associated *etrusca* with an illustration of a specimen from Toscana (pl. 47, fig. 3). However, the name was explicitly referred to as synonym of the (unnamed) variety β, which is not a valid name, and thus the requirements of Art. 11.6 (and therefore 12.1) are not met (see Note 2).

Westerlund (1886: 128) used the name as “[*Melanopsis lorcana*] var. *etrusca* Villa ap. Brot (Cat. syst. Melan. 1862)” and provided a description and thus made it available as species-group taxon. Since he referred to Brot’s catalogue, the type locality comprises both his and Brot’s records. The authority of *Melanopsis etrusca* remains with Westerlund. Art. 11.6.1 does not apply here as Brot (1879) had listed the name as synonym of “Var. β”, which is not a valid name (see Note 2).

#### † Melanopsis eulimopsis

Taxon classificationAnimaliaSorbeoconchaMelanopsidae

Brusina, 1902

##### Original source.

Brusina 1902: pl. 5, figs 42–44.

##### Type horizon.

Middle Pannonian, late Miocene.

##### Type locality.

“Kúp”, Hungary.

##### Types.

The illustrated syntypes are stored in the Croatian Natural History Museum, Zagreb, coll. no. 2494-140/1-3 (Milan et al. 1974: 90).

##### Remarks.

Considered a junior synonym of *Melanopsis pygmaea mucronata* Handmann, 1887 by Papp (1953b: 150).

#### Melanopsis eumorphia

Taxon classificationAnimaliaSorbeoconchaMelanopsidae

Bourguignat, 1884

##### Original source.

Bourguignat 1884: 146.

##### Type locality.

“Dans le Jourdain, à 4 kilomètres au-dessus de la Mer Morte” [in the Jordan river, 4 km above the Dead Sea], Palestine/Jordan.

##### Remarks.

Apparently by mistake, Heller et al. (2005: 245, 247) listed the species in synonymy of *Melanopsis lampra* Bourguignat, 1884 as well as of *Melanopsis obliqua* Bourguignat, 1884, both of which they ranked as subspecies of *Melania costata* (Olivier, 1804).

#### † Melanopsis (Lyrcea) euphrosinae

Taxon classificationAnimaliaSorbeoconchaMelanopsidae

Cobălcescu, 1883

##### Original source.

Cobălcescu 1883: 124, pl. 9, figs 11a–b.

##### Type horizon.

Dacian, Pliocene.

##### Type locality.

“Beceni”, Romania.

#### † Melanopsis eurystoma

Taxon classificationAnimaliaSorbeoconchaMelanopsidae

Neumayr in Neumayr & Paul, 1875

##### Original source.

Neumayr and Paul 1875: 49, pl. 8, fig. 30.

##### Type horizon.

Cernikian, Pliocene.

##### Type locality.

“Čaplathal” [Čaplja trench near Slavonski Brod], Croatia.

#### Melanopsis excoriata

Taxon classificationAnimaliaSorbeoconchaMelanopsidae

Pallary, 1920

##### Original source.

Pallary 1920a: 31.

##### Type locality.

“Aït Brahim; Tazouta; El Menzel; [...] l’Oued Raha, à Agouraï” [Aït Brahim, Tazouta, El Menzel, Oued Rha at Agourai], Morocco.

#### Melanopsis chehirensis var. exilis

Taxon classificationAnimaliaSorbeoconchaMelanopsidae

Pallary, 1939

##### Original source.

Pallary 1939: 95, pl. 6, figs 51, 52, 63.

##### Type locality.

“Yeni Chehir” [Yenişehir], Turkey.

#### Melanella eximia

Taxon classificationAnimaliaSorbeoconchaMelanopsidae

Bourguignat, 1884

##### Original source.

Bourguignat 1884: 13.

##### Type locality.

“Rivière d’Ostaria, entre Plaski et Ogulin, en Croatie; la Save, à Sissek, en Slavonie; la Narenta, en Dalmatie” [river at Oštarije, between Plaški and Ogulin; Sava river at Sisak in Slavonia; in the Narenta river in Dalmatia], Croatia.

##### Remarks.

Note that Bourguignat denoted the authority as “Bourguignat, 1880”.

#### Melanopsis eximia

Taxon classificationAnimaliaSorbeoconchaMelanopsidae

Pallary, 1928

##### Original source.

Pallary 1928a: 260, pl. 5, figs 16–18.

##### Type locality.

“Agouraï, à 29 kil. sud de Fès” [Agourai, claimed to be 29 km south of Fès; judging from the map, Pallary obviously mixed up Fès with Meknès in this case], Morocco.

#### Melanopsis magnifica var. expansa

Taxon classificationAnimaliaSorbeoconchaMelanopsidae

Pallary, 1920

##### Original source.

Pallary 1920a: 33.

##### Type locality.

“Agouraï” [Agourai, south of Meknes], Morocco.

#### † Melanopsis (Lyrcea) delessei var. expansa

Taxon classificationAnimaliaSorbeoconchaMelanopsidae

Magrograssi, 1928 [invalid]

##### Original source.

Magrograssi 1928: 259, pl. 6, fig. 15.

##### Type horizon.

Plio-Pleistocene.

##### Type locality.

“Coo: frequente nella zona centrale fossilifera” [Kos island: common in the central fossiliferous zone], Greece.

##### Remarks.

Junior homonym of *Melanopsis magnifica expansa* Pallary, 1920 (see Note 1).

#### † Melanopsis (Martinia) martiniana var. extensa

Taxon classificationAnimaliaSorbeoconchaMelanopsidae

Handmann, 1887

##### Original source.

Handmann 1887: 26, pl. 5, figs 3–4.

##### Type horizon.

Pannonian, zone B–D, late Miocene.

##### Type locality.

“Leobersdorf”, Austria.

##### Remarks.

Wenz (1929: 2720) considered the taxon as a junior synonym of *Melanopsis fossilis* (Gmelin, 1791).

#### † Melanopsis faberi

Taxon classificationAnimaliaSorbeoconchaMelanopsidae

Brusina, 1884

##### Original source.

Brusina 1884a: 167, pl. 29, fig. 1.

##### Type horizon.

Portaferrian (Pannonian Basin), late Miocene–Pliocene.

##### Type locality.

“Okrugljak” [near Zagreb], Croatia.

##### Types.

Milan et al. (1974: 90) indicated a holotype, but it is uncertain whether the specimen was the only one Brusina had at hand (holotype by monotypy, Art. 73.1.2). The specimen is stored in the Croatian Natural History Museum, Zagreb, coll. no. 2966-612.

#### Melanella fagotiana

Taxon classificationAnimaliaSorbeoconchaMelanopsidae

Bourguignat, 1884

##### Original source.

Bourguignat 1884: 18.

##### Type locality.

“Dans la rivière de la Krapina, à Sused, en Croatie” [in the river Krapina at Podsused, in Zagreb], Croatia.

##### Remarks.

Note that Bourguignat denoted the authority as “Bourguignat, 1879”.

#### † Melanopsis fallax

Taxon classificationAnimaliaSorbeoconchaMelanopsidae

Pantanelli, 1886

##### Original source.

Pantanelli 1886a: 64 or 1886b: 67, pl. 3, figs 12–13 (precedence not established).

##### Type horizon.

Messinian, late Miocene.

##### Type locality.

“Sterza” (Capellini 1873: 550) [near Lajatico], Italy.

##### Remarks.

Introduced for the record of *Melanopsis acicularis* sensu Capellini, 1873, non Férussac, 1823.

#### Melanopsis fasciata

Taxon classificationAnimaliaSorbeoconchaMelanopsidae

Gassies, 1874

##### Original source.

Gassies 1874: 381.

##### Type locality.

“Nékété” [Nakéty], New Caledonia.

##### Remarks.

Brot (1879: 444) considered the taxon as a junior synonym of *Melanopsis frustulum* Morelet, 1857.

#### † Melanopsis fasciata

Taxon classificationAnimaliaSorbeoconchaMelanopsidae

Handmann, 1882 [invalid]

##### Original source.

Handmann 1882: 559.

##### Type horizon.

Pannonian, zone D, late Miocene.

##### Type locality.

“Kottingbrunn [...] Ziegelei a”, Austria.

##### Remarks.

Junior homonym of *Melanopsis fasciata* Gassies, 1874. Pallary (1916: 86) introduced *Melanopsis vittata* as replacement name. The taxon was considered as a junior synonym of *Melanopsis haueri* by Wenz (1929: 2741).

#### Melanopsis doriae var. fasciata

Taxon classificationAnimaliaSorbeoconchaMelanopsidae

Biggs, 1937 [invalid]

##### Original source.

Biggs 1937: 248.

##### Type locality.

“From a ‘qanat’ [= typical Iranian channel] at Jelalabad, near Kerman”, Iran.

##### Remarks.

Junior homonym of *Melanopsis fasciata* Gassies, 1874.

#### “ Melania fasciata

Taxon classificationAnimaliaSorbeoconchaMelanopsidae

” mentioned in Brusina (1866: 106) and Brusina (1867: 86) [unavailable]

##### Locality.

“Cetina”, Croatia.

##### Remarks.

Nomen nudum, “in schedis” name in collection of Kucik (also read as “Kutschig”), occasionally listed with authority “Stentz”. If available, the name would be junior homonym of *Melania fasciata* Sowerby, 1818 described from the Paleogene of the United Kingdom.

#### Melanopsis maroccana var. fasciolata

Taxon classificationAnimaliaSorbeoconchaMelanopsidae

Bourguignat, 1864

##### Original source.

Bourguignat 1864: 260.

##### Type locality.

“Ruisseau de Ngouca” [N’Goussa], Algeria.

#### Melanopsis fasensis

Taxon classificationAnimaliaSorbeoconchaMelanopsidae

Pallary, 1920

##### Original source.

Pallary 1920a: 32.

##### Type locality.

“Taforalt. Aïn Sfa. Fès (Ras el Mâ). [...] Dar Batha, jardin public de bou Djeloud, séguias de dar el Maghzen, séguias en dehors de bab sidi bou Jida” [Taforhalt; Aïn Sfa; Fes; Ras el Ma; Dar Batha, park of Bou Jeloud, irrigation channels of Dar El Makhzen, irrigation channels outside Bab Sidi Boujida (all latter localities are within Fes)], Morocco.

#### Melanopsis faseolaria

Taxon classificationAnimaliaSorbeoconchaMelanopsidae

Brot, 1868

##### Original source.

Brot 1868: 58, pl. 2, fig. 10.

##### Type locality.

“Persepolis”, Iran.

##### Remarks.

Brot attributed the authority to Parreyss, based on an “in schedis” determination by that author. However, from the following text it becomes clear that Brot is the author of the species.

#### † Melanopsis fateljensis

Taxon classificationAnimaliaSorbeoconchaMelanopsidae

Neubauer, Mandic & Harzhauser, 2014

##### Original source.

Neubauer et al. 2014b: 52, figs 2–11.

##### Type horizon.

Langhian, middle Miocene.

##### Type locality.

“NW slope of Fatelj hill near Kupres”, Bosnia and Herzegovina.

##### Types.

Geological-Paleontological Department, Natural History Museum Vienna, Austria, coll. no. 2011/0138/0107a.

##### Remarks.

Introduced for formerly misidentified *Melanopsis mojsisovicsi* Neumayr, 1880 in Neubauer et al. (2013: 137, figs 5E–F, I–K).

#### Melanopsis feliciani

Taxon classificationAnimaliaSorbeoconchaMelanopsidae

Bourguignat, 1884

##### Original source.

Bourguignat 1884: 145.

##### Type locality.

“Dans le Jourdain, non loin de son embouchure dans la Mer Morte” [in the Jordan river, near its mouth at the Dead Sea], Palestine/Jordan.

#### Melanopsis ferussaci

Taxon classificationAnimaliaSorbeoconchaMelanopsidae

Roth, 1839

##### Original source.

Roth 1839: 24, pl. 2, fig. 10.

##### Type locality.

“Smyrna” [Izmir], Turkey.

##### Remarks.

Heller et al. (2005: 232) considered the species as a junior synonym of *Melanopsis buccinoidea* (Olivier, 1801).

#### Melanopsis excoriata var. festiva

Taxon classificationAnimaliaSorbeoconchaMelanopsidae

Pallary, 1920

##### Original source.

Pallary 1920c: 144, pl. 4, figs 21–22.

##### Type locality.

“Aït Brahim”, Morocco.

#### Melanopsis foleyi var. festiva

Taxon classificationAnimaliaSorbeoconchaMelanopsidae

Pallary, 1928 [invalid]

##### Original source.

Pallary 1928a: 271.

##### Type locality.

“Aïn Mélias, près de Figuig” [Ain Melias near Figuig], Algeria.

##### Remarks.

Junior homonym of *Melanopsis excoriata festiva* Pallary, 1920 (see Note 1).

#### † Melanopsis filifera

Taxon classificationAnimaliaSorbeoconchaMelanopsidae

Neumayr, 1880

##### Original source.

Neumayr 1880c: 479, pl. 7, figs 6–7.

##### Type horizon.

Early Langhian, middle Miocene.

##### Type locality.

“Westlich von Drvar” [west of Drvar], Bosnia and Herzegovina.

#### † Melanopsis flammulata

Taxon classificationAnimaliaSorbeoconchaMelanopsidae

De Stefani, 1877

##### Original source.

De Stefani 1877: 306, pl. 18, fig. 7.

##### Type horizon.

Villafranchian, Plio-Pleistocene.

##### Type locality.

“Monte Albuccio, [...] Valli della Pescaia, e del Riluogo [...] presso Siena; Castellacela presso Massa Marittima [...]; Colline di Piedimonte presso Terni nell’Umbria [...], tra S. Gemine e Carsoli sulla strada di Narni, e fra Otricoli e le Vigne sulla strada da Roma a Foligno?” [Monte Albuccio, Valli della Pescaia, Riluogo near Siena, Castellacela near Massa Marittima, Piedimonte hills near Terni in Umbria, between San Gemini and Carsoli on the road to Narni, and between Otricoli and Le Vigne], Italy.

##### Remarks.

Wenz (1929: 2663) considered the taxon as a junior synonym of “*Melanopsis antiqua* Férussac”, which is not an available name.

#### “ Melania holandri var. flava

Taxon classificationAnimaliaSorbeoconchaMelanopsidae

Zelebor” mentioned in Brot (1874–1879: 12) [unavailable]

##### Locality.

Not indicated.

##### Remarks.

Nomen nudum, “in schedis” name from Zelebor listed in synonymy of “*Melania holandri*” [sic] by Brot (1874). If available, it would be junior homonym of *Melania flava* Deshayes in Deshayes & Jullien, 1876 described from Cambodia.

#### Melanopsis foleyi

Taxon classificationAnimaliaSorbeoconchaMelanopsidae

Pallary, 1920

##### Original source.

Pallary 1920a: 29.

##### Type locality.

“Aïn Mélias, près de Figuig” [Ain Melias near Figuig], Algeria.

#### † Melanopsis foliacea

Taxon classificationAnimaliaSorbeoconchaMelanopsidae

Gümbel, 1861

##### Original source.

Gümbel 1861: 753.

##### Type horizon.

Chattian, Oligocene.

##### Type locality.

“Wester-Buchberg, Loherflötz, Aubach, Rohmbach, Sulzgrabenflötz, Schlierachthal, Neumühle, bei Rimselrain, Pensberg, im Höllgraben, im Buchbergflötz, bei Schmerold, am hohen Peissenberge” [all localities near Miesbach, southern Bavaria], Germany.

#### † Melanopsis (Canthidomus) fontanensis

Taxon classificationAnimaliaSorbeoconchaMelanopsidae

Villatte in Tambareau & Villatte, 1966

##### Original source.

Tambareau and Villatte 1966: 512, pl. 1, figs 4–18.

##### Type horizon.

Sparnacian, early Ypresian, Eocene.

##### Type locality.

“Fontané (Ariège)”, France.

##### Types.

Laboratoire de Géologie de la Faculté des Sciences de Toulouse; no number indicated.

#### † Melanopsis (Martinia) capulus var. fornicata

Taxon classificationAnimaliaSorbeoconchaMelanopsidae

Handmann, 1887

##### Original source.

Handmann 1887: 21, pl. 2, fig. 20.

##### Type horizon.

Pannonian, zone B–D, late Miocene.

##### Type locality.

“Leobersdorf”, Austria.

#### Melanopsis costata var. forticostata

Taxon classificationAnimaliaSorbeoconchaMelanopsidae

Paetel, 1888

##### Original source.

Paetel 1888: 400.

##### Type locality.

“Lake Huleh [Palestine]” (Nevill 1884: 212), Palestine.

##### Remarks.

Originally introduced as infrasubspecific taxon (“subvariety”) by Nevill (1884), but made available by Paetel (1888) who treated it as variety (Art. 45.5.1). Paetel clearly referred to the description of Nevill.

#### † Melania fossariformis

Taxon classificationAnimaliaSorbeoconchaMelanopsidae

Tournouër, 1879

##### Original source.

Tournouër 1879: 261.

##### Type horizon.

Cernikian, Pliocene.

##### Type locality.

“Nisipulu, Rumaniae” [Nisip], Romania.

##### Remarks.

Considered to belong in the genus *Amphimelania* by Cossmann (1909: 126) and Wenz (1929: 2873).

#### † Buccinum fossile

Taxon classificationAnimaliaSorbeoconchaMelanopsidae

Gmelin, 1791

##### Original source.

Gmelin 1971: 3485.

##### Type horizon.

Sarmatian (sensu stricto), middle Miocene.

##### Type locality.

“Zwischen Oedinburg and Ruß” (Martini 1777: 204) [between Sopron (Hungary) and Rust (Austria)], Hungary/Austria.

##### Types.

Papp (1953b: 133) designated the specimen illustrated by Martini (1777: pl. 94, figs 913) as lectotype (see also Art. 74.5). Since Gmelin explicitly referred to Martini’s work when describing the species, this designation is valid.

##### Remarks.

The taxon in question was first mentioned in the Catalogus by Walch (1768: 121, pl. C. II.*, figs 1–5), yet without name. Martini (1777: 203, pl. 94, figs 912–914) described and illustrated the species as “Pyrum fossile monstrosum f. amorphon” and referred to Walch’s work. Species-group names introduced in volumes 1–11 of Martini and Chemnitz’ Neues Systematischer Conchylien Cabinet (1769–1795) are, however, unavailable according to Opinion 184 (ICZN 1944). Gmelin (1791: 3485) referred to Martini’s work, described the species and was the first to assign an available name to the taxon: *Buccinum fossile*.

Férussac (1823), who was the first to combine the species with *Melanopsis*, listed the records by Walch, Martini and Gmelin but apparently did not consider “*fossile*” a valid name and introduced “*Melanopsis martiniana*” as new name. That name is permanently invalid as it is an objective synonym (see also Pallary 1916: 81).

The last summary of the status of *Melanopsis fossilis* was provided by Fischer (1996a), but his conclusions were unfortunately based on misinterpretations and nomenclatural errors. He was unaware of the fact that “*Melanopsis martiniana*” is an objective synonym of *Melanopsis fossilis*. Moreover, he considered the outcrops near Siegendorf as “locus typicus restrictus”, a term not existing in nomenclature.

#### Melanopsis foucauldiana

Taxon classificationAnimaliaSorbeoconchaMelanopsidae

Pallary, 1920

##### Original source.

Pallary 1920a: 30.

##### Type locality.

“Ouggarta, à 60 kil. au sud de Beni Abbès, Sahara occidental” [Ougarta, prov. Béchar], Algeria.

#### Melanopsis foureaui

Taxon classificationAnimaliaSorbeoconchaMelanopsidae

Pallary, 1920

##### Original source.

Pallary 1920a: 29.

##### Type locality.

“Tabebala, dans le Sahara occidental” [Tabebala (?), in the western Sahara], Algeria.

#### Melanopsis fragilis

Taxon classificationAnimaliaSorbeoconchaMelanopsidae

Gassies, 1874

##### Original source.

Gassies 1874: 382.

##### Type locality.

“Ouagap” [Wagap], New Caledonia.

#### “ Melania fragilis

Taxon classificationAnimaliaSorbeoconchaMelanopsidae

Schmidt” mentioned in Brusina (1867: 86) [unavailable]

##### Locality.

Not indicated.

##### Remarks.

Nomen nudum, “in schedis” name in collection of Kucik (also read as “Kutschig”). If available, the name would be junior homonym of *Melania fragilis* Lamarck, 1804 from the Paleogene (Eocene?) of the Paris Basin.

#### † Melanopsis franciscae

Taxon classificationAnimaliaSorbeoconchaMelanopsidae

Brusina, 1903

##### Original source.

Brusina 1903: 113.

##### Type horizon.

Late Pleistocene–Holocene.

##### Type locality.

“Bischofsbad” [Püspökfürdő, Băile 1 Mai, Lake Pețea], Romania.

##### Remarks.

Neubauer et al. (2014d: 125) considered this taxon as a junior synonym of *Microcolpia parreyssii sikorai* (Brusina, 1903).

#### † Melanopsis (Canthidomus) freybergi

Taxon classificationAnimaliaSorbeoconchaMelanopsidae

Kühn, 1951

##### Original source.

Kühn 1951: 186, pl. 17, figs 4–5.

##### Type horizon.

Mammal zone MN 10, late Miocene.

##### Type locality.

“Nordwestlich Nea Liossia” [northwest of Ilion], Greece.

##### Types.

Geological-Paleontological Department, Natural History Museum Vienna, Austria, coll. no. 1949/0004/0006.

#### † Amphimelania frici

Taxon classificationAnimaliaSorbeoconchaMelanopsidae

Brusina, 1897

##### Original source.

Brusina 1897: 6.

##### Type horizon.

Late Cernikian, late Pliocene–early Pleistocene.

##### Type locality.

“Kutina (šuma Mišinka)” [Kutina, Mišinka forest], Croatia.

##### Types.

Milan et al. (1974: 57) stated that Brusina (1897) had indicated one of the specimens illustrated by him (pl. 3, figs 27–28) as type. This can be accepted as valid lectotype designation in the sense of Art 74.5. The specimen is stored in the Croatian Natural History Museum, Zagreb, coll. no. 2964-610/1.

#### † Melanopsis friedeli

Taxon classificationAnimaliaSorbeoconchaMelanopsidae

Brusina, 1885

##### Original source.

Brusina 1885: 160.

##### Type horizon.

Cernikian, Pliocene.

##### Type locality.

“Kravarsko und Podvornica”, Croatia.

##### Types.

Milan et al. (1974: 91) defined a neotype based on the specimens illustrated by Brusina (1897: 8, pl. 6, figs 5–8) because the specimen from 1885 was apparently lost. The type fixation is insufficient with respect to Art. 75.3 of the Code. The specimen is stored in the Croatian Natural History Museum, Zagreb, coll. no. 2998-644.

#### † Melanopsis fritzei

Taxon classificationAnimaliaSorbeoconchaMelanopsidae

Thomä, 1845

##### Original source.

Thomä 1845: 158, pl. 2, figs 7a–b.

##### Type horizon.

Mammal zone MN 2, early Miocene.

##### Type locality.

“Mühltal bei Wiesbaden”, Germany.

##### Remarks.

The name “*Fritzii*” as mentioned in Chenu (1859: 297) is an incorrect subsequent spelling.

#### Melanopsis frustulum

Taxon classificationAnimaliaSorbeoconchaMelanopsidae

Morelet, 1857

##### Original source.

Morelet 1857: 33.

##### Type locality.

“[Ad Sanctam-Mariam de Balade]” [Balade], New Caledonia.

#### † Melanopsis fuchsi

Taxon classificationAnimaliaSorbeoconchaMelanopsidae

Handmann, 1882

##### Original source.

Handmann 1882: 556.

##### Type horizon.

Pannonian, zone D, late Miocene.

##### Type locality.

“Kottingbrunn [...] Ziegelei a”, Austria.

##### Remarks.

Wenz (1929: 2813) synonymized the species with *Melanopsis pygmaea* Hörnes, 1856. Jekelius (1944: 75) invalidly synonymized it with *Melanopsis handmanni*, which is the objective junior synonym of *Melanopsis fuchsi* Handmann, 1882.

#### † Melanopsis fuchsi

Taxon classificationAnimaliaSorbeoconchaMelanopsidae

Brusina, 1884 [invalid]

##### Original source.

Brusina 1884a: 168.

##### Type horizon.

Transdanubian, Pannonian, late Miocene.

##### Type locality.

“Radmanest” (Fuchs 1870: 353) [Rădmănești], Romania.

##### Types.

The syntypes are stored in the Croatian Natural History Museum, Zagreb; no number indicated (Milan et al. 1974: 91).

##### Remarks.

Introduced for *Melania costata* sensu Fuchs, 1870, non Olivier, 1804. Junior homonym of *Melanopsis fuchsi* Handmann, 1882 (see *Melanopsis confusa* Strausz, 1941, *Melanopsis handmanni* Brusina, 1892 and *Melanopsis hungarica* Pallary, 1916, as well as Neubauer et al. 2014a: 461 for details).

#### † Amphimelania fuchsi

Taxon classificationAnimaliaSorbeoconchaMelanopsidae

Wenz, 1928

##### Original source.

Wenz 1928a: 119.

##### Type horizon.

Late Pliocene to early Pleistocene.

##### Type locality.

“Kalamaki” [near Corinth], Greece.

##### Remarks.

Replacement name for Paludina (Vivipara) ornata Fuchs, 1877, non *Vivipara ornata* Neumayr, 1875, which was considered a Melanopsidae by Wenz (1928a).

#### Melanopsis fulgurans

Taxon classificationAnimaliaSorbeoconchaMelanopsidae

Gassies, 1858

##### Original source.

Gassies 1858: 371.

##### Type locality.

“Des petits cours d’eau à Balade; [...] à l’île des Pins” [in small streams near Balade; L’Île des Pins], New Caledonia.

##### Remarks.

Brot (1879: 444) considered the taxon as a junior synonym of *Melanopsis frustulum* Morelet, 1857.

#### “ Melanopsis fulminans

Taxon classificationAnimaliaSorbeoconchaMelanopsidae

Born.” mentioned in De Cristofori & Jan (1832: Conchylia terrestria et fluviatilia, p. 7) [unavailable]

##### Locality.

“Austr.” [Australia].

##### Remarks.

Nomen nudum, found only in the species list of De Cristofori and Jan (1832).

#### Melanopsis fulminata

Taxon classificationAnimaliaSorbeoconchaMelanopsidae

Brot, 1879

##### Original source.

Brot 1874–1879: 458, pl. 49, fig. 10.

##### Type locality.

“Ouagap” [Wagap], New Caledonia.

#### † Melanopsis costata var. funiculata

Taxon classificationAnimaliaSorbeoconchaMelanopsidae

P. Fischer in Gaudry, 1867

##### Original source.

Gaudry 1862–1867: 446, pl. 62, figs 13–15.

##### Type horizon.

Pliocene.

##### Type locality.

“Mégare” (p. 444), Greece.

##### Remarks.

The taxon is not included in the Fossilium Catalogus of Wenz (1929).

#### Melanopsis fusca

Taxon classificationAnimaliaSorbeoconchaMelanopsidae

Gassies, 1870

##### Original source.

Gassies 1870: 147.

##### Type locality.

“Prope Pouebo” [near Pouébo], New Caledonia.

#### † Melanopsis fusiformis

Taxon classificationAnimaliaSorbeoconchaMelanopsidae

Sowerby, 1822

##### Original source.

Sowerby 1821–1823: 35, pl. 332, figs 1–7.

##### Type horizon.

Eocene.

##### Type locality.

“Isle of Wight; [...] New Charlton; [...] Woolwich; [...] New Cross near Deptford; [...] Hordwell”, United Kingdom.

#### † Melanopsis buccinoidea var. fusiformis

Taxon classificationAnimaliaSorbeoconchaMelanopsidae

Grateloup, 1838 [invalid]

##### Original source.

Grateloup 1838: 146.

##### Type horizon.

Burdigalian, early Miocene.

##### Type locality.

“Dax. [...] Mandillot”, France.

##### Remarks.

Based on some of the illustrated syntypes of “*Melanopsis antiqua* Férussac, 1823” (actually, Grateloup referred to the illustrations of *Melania buccinoidea* in Férussac 1823; “*Melanopsis antiqua* Férussac” is not an available name). Junior homonym of *Melanopsis fusiformis* Sowerby, 1822.

#### Melanopsis fusiformis

Taxon classificationAnimaliaSorbeoconchaMelanopsidae

Gassies, 1870 [invalid]

##### Original source.

Gassies 1870: 148.

##### Type locality.

“Prope Kanala; insula Ouen” [near Canala; Île Ouen], New Caledonia.

##### Remarks.

Junior homonym of *Melanopsis fusiformis* Sowerby, 1822. Gassies (1880) introduced *Melanopsis rossiteri* as replacement name. Unaware of this, Pallary (1916) introduced *Melanopsis cookiana* as replacement name, which is its junior objective synonym.

#### † Melanopsis fusiformis

Taxon classificationAnimaliaSorbeoconchaMelanopsidae

Handmann, 1882 [invalid]

##### Original source.

Handmann 1882: 560.

##### Type horizon.

Pannonian, zone D, late Miocene.

##### Type locality.

“Kottingbrunn [...] Ziegelei a”, Austria.

##### Remarks.

Junior homonym of *Melanopsis fusiformis* Sowerby, 1822. Pallary (1916: 82) introduced *Melanopsis angusta* as replacement name. Wenz (1929: 2742) considered the taxon as a junior synonym of *Melanopsis haueri* Handmann, 1882.

#### † Melanopsis (Canthidomus) proteus var. fusiformis

Taxon classificationAnimaliaSorbeoconchaMelanopsidae

Magrograssi, 1928 [invalid]

##### Original source.

Magrograssi 1928: 260, pl. 6, fig. 17.

##### Type horizon.

Plio-Pleistocene.

##### Type locality.

“Coo: V. Tuvachiu, fra Antimachia e Pili” [Kos island: Tuvachia valley (?), between Antimácheia and Pýli], Greece.

##### Remarks.

Junior homonym of *Melanopsis fusiformis* Sowerby, 1822.

#### † Melanopsis praemorsa var. fusulatina

Taxon classificationAnimaliaSorbeoconchaMelanopsidae

Sacco, 1895

##### Original source.

Sacco 1895: 9, pl. 1, fig. 13.

##### Type horizon.

Messinian, late Miocene.

##### Type locality.

“Priosa presso Narzole, Castelletto d’Orba, S. Marzano Oliveto”, Italy.

#### † Melanopsis gaasensis

Taxon classificationAnimaliaSorbeoconchaMelanopsidae

Vergnau-Saubade, 1968

##### Original source.

Vergnau-Saubade 1968: 201, [unnumbered plate], fig. 10.

##### Type horizon.

Oligocene.

##### Type locality.

“Gaas - Lesbarritz”, France.

##### Remarks.

First mentioned in an unpublished thesis (Vergnau-Saubade 1966).

#### † Melania gaji

Taxon classificationAnimaliaSorbeoconchaMelanopsidae

Brusina, 1878

##### Original source.

Brusina 1878: 347.

##### Type horizon.

Middle–late Cernikian, late Pliocene–early Pleistocene.

##### Type locality.

“Sibinj”, Croatia.

##### Types.

Milan et al. (1974: 57) indicated a holotype, but it is uncertain whether the specimen is part of the original type series and whether it was the only one Brusina had at hand (holotype by monotypy, Art. 73.1.2). The specimen is stored in the Croatian Natural History Museum, Zagreb, coll. no. 2963-609.

##### Remarks.

Considered to belong in the genus *Amphimelania* by Brusina (1897: 6), Cossmann (1909: 126) and Wenz (1929: 2874). The names “*caji*” as mentioned in Cossmann (1909: 126) and “*gayi*” as given by Bandel (2000: 140) are incorrect subsequent spellings.

#### Fagotia gallandi

Taxon classificationAnimaliaSorbeoconchaMelanopsidae

Bourguignat, 1884

##### Original source.

Bourguignat 1884: 41.

##### Type locality.

“Affluents du lac Sabandja, près d’Ismidt” [tributaries to the Lake Sapanca near İzmit], Turkey.

##### Remarks.

Appeared first as a nomen nudum in Locard (1883a).

#### Microcolpia gallandi

Taxon classificationAnimaliaSorbeoconchaMelanopsidae

Bourguignat, 1884

##### Original source.

Bourguignat 1884: 60.

##### Type locality.

“Constantinople [...], a été trouvée dans la rivière d’Ismidt (Anatolie)” [Istanbul, found in a river at İzmit], Turkey.

##### Remarks.

Starobogatov et al. (1992: 68) considered the species as a junior synonym of *Microcolpia coutagniana* (Pfeiffer, 1828).

#### † Melanopsis galloprovincialis

Taxon classificationAnimaliaSorbeoconchaMelanopsidae

Matheron, 1842

##### Original source.

Matheron 1842: 291, pl. 37, figs 1–6.

##### Type horizon.

Campanian–Maastrichtian, Cretaceous.

##### Type locality.

“Les Martigues, les Pennes, Simiane, Gardanne, Fuveau, Peynier, Trets”, France.

#### † Melanopsis garumnica

Taxon classificationAnimaliaSorbeoconchaMelanopsidae

Munier-Chalmas, 1884

##### Original source.

Munier-Chalmas 1884: 330, pl. 7, figs 21–22.

##### Type horizon.

Maastrichtian, Cretaceous.

##### Type locality.

“D’Auzas”, France.

##### Remarks.

Munier-Chalmas (1884) indicated the authority with “Mun. Ch., 1870”, which is probably due to the fact that issues 1–3 of vol. 1 of Annales de Malacologie, in which the original publication appeared, were published in 1870 (issue 4, however, in 1884).

#### Melanopsis gassiesiana

Taxon classificationAnimaliaSorbeoconchaMelanopsidae

Crosse, 1867

##### Original source.

Crosse 1867: 435, pl. 12, fig. 7.

##### Type locality.

“Wagap”, New Caledonia.

#### † Melanopsis gearyae

Taxon classificationAnimaliaSorbeoconchaMelanopsidae

Neubauer, Harzhauser, Georgopoulou, Mandic & Kroh, 2014

##### Original source.

Neubauer et al. 2014a: 456.

##### Type horizon.

Early Pleistocene (?).

##### Type locality.

“Weganrisse und Regenrisse 3 Km N Antirrion in Akarnanien” (Symeonidis et al. 1986: 342) [sections 3 km N of Antirrio, Aetolia-Acarnania], Greece.

##### Types.

Senckenberg Forschungsinstitut und Naturmuseum Frankfurt; no number indicated.

##### Remarks.

Replacement name for *Melanopsis posterior* Schütt, 1986, non Papp, 1953.

#### † Melanopsis geniculata

Taxon classificationAnimaliaSorbeoconchaMelanopsidae

Brusina, 1874

##### Original source.

Brusina 1874: 40, pl. 1, figs 9–10.

##### Type horizon.

Langhian, middle Miocene.

##### Type locality.

“Miočić”, Croatia.

##### Types.

Milan et al. (1974: 91) indicated a holotype, but it is uncertain whether the specimen was the only one Brusina had at hand (holotype by monotypy, Art. 73.1.2). The specimen is stored in the Croatian Natural History Museum, Zagreb, coll. no. 3207-853.

#### Melanopsis germaini

Taxon classificationAnimaliaSorbeoconchaMelanopsidae

Pallary, 1939

##### Original source.

Pallary 1939: 93, pl. 6, figs 5–8, 14.

##### Type locality.

“Sources du Nahr es Sine, au Sud de Lattaquié, sur la route de Beyrouth” [Nahr as Sinn, south of Latakia, at the road to Beirut], Syria.

##### Remarks.

Heller et al. (1999: 61) considered the species as a junior synonym of *Melanopsis costata* (Olivier, 1804).

#### † Melanopsis gersondei

Taxon classificationAnimaliaSorbeoconchaMelanopsidae

Willmann, 1981

##### Original source.

Willmann 1981: 143.

##### Type horizon.

Palioskala Formation, middle Tortonian, late Miocene.

##### Type locality.

“Bachriß beim Höhenzug von Palioskala, ca. 700 m westlich vom Kap Phoka” [brook section near the mountain range of Palioskala, ca. 700 m west of Ágios Fokás, Kos Island], Greece.

##### Types.

Geological-Paleontological Institute, University of Kiel, Germany; no number indicated.

#### † Melanopsis gibbosula

Taxon classificationAnimaliaSorbeoconchaMelanopsidae

Grateloup, 1838

##### Original source.

Grateloup 1838: 141, pl. 4, fig. 50.

##### Type horizon.

Chattian–Burdigalian (?), late Oligocene–early Miocene.

##### Type locality.

“Dax. [...] d’Abesse et de Quillac à Saint-Paul” [Dax; Abesse and Quillac at Saint-Paul-lès-Dax], France.

##### Remarks.

Wenz (1929: 2774) considered the taxon as a junior synonym of “*Melanopsis major* Férussac, 1823”, which is not an available name.

#### Melanella gigantea

Taxon classificationAnimaliaSorbeoconchaMelanopsidae

Bourguignat, 1884

##### Original source.

Bourguignat 1884: 19.

##### Type locality.

“Rivière près d’une villa sur la route de Pregrada, aux environs de Krapina, en Croatie” [river near a villa on the road to Pregrada, near Krapina], Croatia.

##### Remarks.

Note that Bourguignat denoted the authority as “Bourguignat, 1879”.

#### † Melanopsis narzolina gigantea

Taxon classificationAnimaliaSorbeoconchaMelanopsidae

Robles, 1975

##### Original source.

Robles 1975: 362, pl. 2, figs 2–8.

##### Type horizon.

Late Miocene.

##### Type locality.

“Venta del Moro (Valencia)”, Spain.

##### Types.

Museo Nacional de Ciencias Naturales, Madrid, coll. no. M-327.

#### † Melanopsis costata var. glabra

Taxon classificationAnimaliaSorbeoconchaMelanopsidae

Brusina, 1874

##### Original source.

Brusina 1874: 41, pl. 7, fig. 9.

##### Type horizon.

Cernikian, Pliocene.

##### Type locality.

“Sibinj; Farkašić; Dubranjec”, Croatia.

##### Types.

Milan et al. (1974: 89) stated that the single specimen of this taxon was lost.

#### † Melanopsis (Melanoptychia) glabra

Taxon classificationAnimaliaSorbeoconchaMelanopsidae

Pavlović, 1927 [invalid]

##### Original source.

Pavlović 1927: 60, pl. 6, figs 18–19.

##### Type horizon.

Middle Pannonian, late Miocene.

##### Type locality.

“У Карагачу” [from Karagača near Vrčin], Serbia.

##### Types.

The illustrated syntype is stored in the Natural History Museum, Belgrade, coll. no. 209 (Milošević 1962: 23).

##### Remarks.

Junior homonym of *Melanopsis glabra* Brusina, 1874. Neubauer et al. (2014a: 457) introduced *Melanopsis vrcinensis* as replacement name.

#### † “ Melanopsis glabra

Taxon classificationAnimaliaSorbeoconchaMelanopsidae

” mentioned in Bogachev (1938: 13, 33, 53) [unavailable]

##### Horizon.

Miocene.

##### Locality.

“Tori bei Borshomi” [Tori near Borjomi], Georgia.

##### Remarks.

The name was only mentioned in a species list by Bogachev without description or illustration. Moreover, he applied multiple original spellings: the name is given as “*glabra*” on p. 53, but “*glarba*” on p. 13, 33.

#### † Melanoptychia lyrata glabra

Taxon classificationAnimaliaSorbeoconchaMelanopsidae

Olujić, 1999 [invalid]

##### Original source.

Olujić 1999: 20, 48, pl. 1, fig. 1.

##### Type horizon.

Langhian, middle Miocene.

##### Type locality.

It is unclear from the original work in which of the studied localities/sections along the valleys of the Sutina, Batarelov and Vojskava rivers (4 km W of Sinj) the taxon occurred and in which not, Croatia.

##### Remarks.

Junior secondary homonym of *Melanopsis glabra* Brusina, 1874. Neubauer et al. (2011: 207) considered the taxon as a junior synonym of *Melanopsis lyrata* Neumayr, 1869.

#### Melanopsis parreyssii var. glabrata

Taxon classificationAnimaliaSorbeoconchaMelanopsidae

Clessin, 1890

##### Original source.

Clessin 1887–1890: 690, fig. 475.

##### Type locality.

“Ungarn ([...] ohne nähere Fundortangabe)” [Hungary, without indication of a locality], Hungary.

##### Remarks.

Neubauer et al. (2014d: 125) considered this taxon as a junior synonym of *Microcolpia parreyssii* (Philippi, 1847).

#### † Melanopsis glandicula

Taxon classificationAnimaliaSorbeoconchaMelanopsidae

Sandberger, 1872

##### Original source.

Sandberger 1870–1875: pl. 26, figs 3–3b.

##### Type horizon.

Mammal zone MN 5, early–middle Miocene.

##### Type locality.

“Pontlevoy bei Blois” [Pontlevoy near Blois], France.

##### Remarks.

Plate 26 of Sandberger’s monograph was issued in 1872, while the description on p. 520 appeared in 1875 (Woodward 1906).

#### Melanopsis foleyi var. glandinopsis

Taxon classificationAnimaliaSorbeoconchaMelanopsidae

Pallary, 1928

##### Original source.

Pallary 1928a: 271, pl. 6, figs 10, 15.

##### Type locality.

“Aïn Mélias, près de Figuig” [Ain Melias near Figuig], Algeria.

#### † Melanopsis glaubrechti

Taxon classificationAnimaliaSorbeoconchaMelanopsidae

Neubauer & Harzhauser in Neubauer et al., 2015

##### Original source.

Neubauer et al. 2015b: 145, figs 3A–D.

##### Type horizon.

Middle Pannonian, late Miocene.

##### Type locality.

“Brickyard near the town of Martin, Turiec Basin”, Slovak Republic.

##### Types.

Múzeum Andreja Kmeťa in Martin, Slovak Republic, coll. no. SNM 16/2011 (PZ-703g).

#### Microcolpia glinaica

Taxon classificationAnimaliaSorbeoconchaMelanopsidae

Bourguignat, 1884

##### Original source.

Bourguignat 1884: 54.

##### Type locality.

“La Glina en Hongrie” [Glina river], Croatia.

##### Remarks.

Starobogatov et al. (1992: 65) considered the species as a junior synonym of *Microcolpia acicularis* (Férussac, 1823).

#### Melanopsis glinensis

Taxon classificationAnimaliaSorbeoconchaMelanopsidae

Brot, 1878

##### Original source.

Brot 1874–1879: 370–371, pl. 38, fig. 4e.

##### Type locality.

“Glina Fl.” [Glina river], Croatia.

##### Remarks.

“In schedis” name by Parreyss, introduced by Brot (1878) in synonymy of “*Hemisinus acicularis* (Férussac, 1823)”. Clessin (1890) made it available by treating it as a valid name (see Note 2). Starobogatov et al. (1992: 65) again considered the species as a junior synonym of *Microcolpia acicularis*.

#### † Melanopsis gorceixi var. globosa

Taxon classificationAnimaliaSorbeoconchaMelanopsidae

Magrograssi, 1928

##### Original source.

Magrograssi 1928: 259, pl. 6, fig. 14.

##### Type horizon.

Plio-Pleistocene.

##### Type locality.

“Coo: V. Bocasia, torrente Sefto, C. Foca, tra Antimachia e Pili” [Kos island: Vokasia valley, Sefto river, Ágios Fokás, between Antimácheia and Pýli], Greece.

#### Melanopsis pachya var. gombaulti

Taxon classificationAnimaliaSorbeoconchaMelanopsidae

Pallary, 1939

##### Original source.

Pallary 1939: 87, pl. 5, figs 37–39.

##### Type locality.

“Dans les sources de Mézérib, au N. O. de Derâa (Syrie méridionale)” [in the sources of the Muzayrīb, northwest of Dar’ā], Syria.

#### † Melanopsis gorceixi

Taxon classificationAnimaliaSorbeoconchaMelanopsidae

Tournouër, 1875

##### Original source.

Tournouër 1875: 76.

##### Type horizon.

Tafi Formation, early Pleistocene.

##### Type locality.

“Prope vicum Antimaki [...] et prope civitatem Cos” [near near the village Antimácheia and near the city of Kos], Greece.

#### Melanopsis goulvaini

Taxon classificationAnimaliaSorbeoconchaMelanopsidae

Pallary, 1916

##### Original source.

Pallary 1916: 82.

##### Type locality.

“Bourail” (Gassies 1874: 384), New Caledonia.

##### Remarks.

Replacement name for *Melanopsis elongata* Gassies, 1874, non Férussac, 1822 (see Note 1).

#### Melanopsis gracilenta

Taxon classificationAnimaliaSorbeoconchaMelanopsidae

Pallary, 1911

##### Original source.

Pallary 1911: 130, [unnumbered plate], fig. 19.

##### Type locality.

“Tout près d’Oudjda, à 4 kilom. S.-E., sourdent les belles sources de Sidi-Yahia qui alimentent une véritable oasis, puis la ville d’Oudjda, et vont finalement se déverser dans l’oued Isly” [near Oujda, 4 km southeast, at the sources of Sidi Yahya that feed an oasis and the city of Oujda, and ultimately will flow into the Oued Isly], Morocco.

#### † Melanopsis (Melanosteira) conemenosiana graciliformis

Taxon classificationAnimaliaSorbeoconchaMelanopsidae

Papp, 1955

##### Original source.

Papp 1955: 129, pl. 20, fig. 26.

##### Type horizon.

Early Pleistocene.

##### Type locality.

“Brochitza, Elis” [Vrokhítsa], Greece.

##### Types.

Museum of Palaeontology and Geology of the University of Athens; no number indicated.

#### † Melanopsis bouei var. gracilis

Taxon classificationAnimaliaSorbeoconchaMelanopsidae

Brusina, 1874

##### Original source.

Brusina 1874: 47.

##### Type horizon.

Pannonian, zone C, late Miocene.

##### Type locality.

“Sused (Sopot bei Goljak)” [Podsused, in Zagreb], Croatia.

##### Remarks.

Wenz (1929: 2673) considered the variety as a junior synonym *Melanopsis bouei* Férussac, 1823.

#### † Melanopsis neumayri var. gracilis

Taxon classificationAnimaliaSorbeoconchaMelanopsidae

Fontannes, 1880 [invalid]

##### Original source.

Fontannes 1879–1882: 174.

##### Type horizon.

Mammal zone MN 11, late Miocene.

##### Type locality.

“Les marnes à *Potamides Basteroti* de Visan (Vaucluse)” [in the marls with *Potamides basteroti* at Visan, Dép. Vaucluse], France.

##### Remarks.

Junior homonym of *Melanopsis bouei gracilis* Brusina, 1874 (see Note 1). Wenz (1929: 2793) considered the variety as a junior synonym *Melanopsis neumayri* Tournouër, 1874.

#### Melanopsis costata var. gracilis

Taxon classificationAnimaliaSorbeoconchaMelanopsidae

Locard, 1883 [invalid]

##### Original source.

Locard 1883a: 288.

##### Type locality.

“Lac d’Homs” [Lake Homs], Syria.

##### Remarks.

Junior homonym of *Melanopsis bouei gracilis* Brusina, 1874 (see Note 1).

#### † Melanopsis (Hyphantria) gracilis

Taxon classificationAnimaliaSorbeoconchaMelanopsidae

Handmann, 1887 [invalid]

##### Original source.

Handmann 1887: 37, pl. 8, fig. 17.

##### Type horizon.

Pannonian, zone B–D, late Miocene.

##### Type locality.

“Leobersdorf”, Austria.

##### Remarks.

Junior homonym of *Melanopsis bouei gracilis* Brusina, 1874 (see Note 1). Wenz (1929: 2673) considered the variety as a junior synonym *Melanopsis bouei* Férussac, 1823.

#### † Melanopsis graciosa

Taxon classificationAnimaliaSorbeoconchaMelanopsidae

Seninski, 1905

##### Original source.

Seninski 1905: 59, pl. 1, fig. 16.

##### Type horizon.

Duab Beds, middle to late Kimmerian, Pliocene.

##### Type locality.

“Моквинскіе пласты, разрѣзъ р. Дуабъ” [Mokvi layers at Duab river], Georgia.

#### † Melanopsis gradata

Taxon classificationAnimaliaSorbeoconchaMelanopsidae

Rolle, 1858

##### Original source.

Rolle 1858: 28, pl. 2, fig. 13.

##### Type horizon.

Paleocene.

##### Type locality.

“Herrmanns-Stollen im Lubellina-Graben, Gemeinde Ober-Skallis, nordöstlich von Schönstein” [Hermanns adit in the Lubellina ditch in Škale, northeast of Šoštanj], Slovenia.

##### Remarks.

After Wenz (1929: 2505) this species belongs in the genus *Pyrgulifera* Meek, 1877 (Pleuroceridae), but Yen (1958: 194) doubted this classification.

#### † Melanopsis gradata

Taxon classificationAnimaliaSorbeoconchaMelanopsidae

Fuchs, 1870 [invalid]

##### Original source.

Fuchs 1870b: 539, pl. 20, figs 13–14.

##### Type horizon.

Transdanubian, Pannonian, late Miocene.

##### Type locality.

“Tihany” (p. 533), Hungary.

##### Remarks.

Junior homonym of *Melanopsis gradata* Rolle, 1858. Wenz (1928b: 219) introduced *Melanopsis tihanyensis* as replacement name.

#### Melanopsis (Mesopotamia) khabourensis var. gradata

Taxon classificationAnimaliaSorbeoconchaMelanopsidae

Pallary, 1939 [invalid]

##### Original source.

Pallary 1939: 103, pl. 5, fig. 20.

##### Type locality.

“Ras el ‘Ain du Khabour” [Chabur river near Ra’s al ‘Ayn], Syria.

##### Remarks.

Junior homonym of *Melanopsis gradata* Rolle, 1858.

#### Melanopsis graellsii

Taxon classificationAnimaliaSorbeoconchaMelanopsidae

Villa & Villa in Graells, 1846

##### Original source.

Graells 1846: 17, [unnumbered plate], figs 16–19.

##### Type locality.

Spain [no locality indicated].

##### Remarks.

Graells (1846) indicated that the “brothers Villa” dedicated the species to him. The name “*graellsi*” as mentioned in Pallary (1924: 250) is an incorrect subsequent spelling. It is unclear why Pallary considered the species as a junior synonym of “*Buccinum annulatum* Chemnitz”. That species, which is not available from Chemnitz (1795: 91, pl. 188, figs 1812–1813) according to Opinion 184 (ICZN 1944), is not even a freshwater snail but dwells in the southern Pacific (Nassariidae?).

#### Melanopsis chehirensis var. granifera

Taxon classificationAnimaliaSorbeoconchaMelanopsidae

Pallary, 1939

##### Original source.

Pallary 1939: 95.

##### Type locality.

Not explicitly stated but probably the same as for the species (“Dans le source de Yeni Chehir, [...] entre Antioche et Alep, à l’intersection de la route d’Alexandrette” [at the source of Yenişehir, between Antakya and Aleppo, at the intersection of the road from İskenderun], Turkey).

#### † Melanopsis granum

Taxon classificationAnimaliaSorbeoconchaMelanopsidae

Calvert & Neumayr, 1880

##### Original source.

Calvert and Neumayr 1880: 376, pl. 2, fig. 23.

##### Type horizon.

Late Sarmatian, Khersonian, late Miocene.

##### Type locality.

“Renkiöi” [north of İntepe], Turkey.

#### † “ Melanopsis grateloupii

Taxon classificationAnimaliaSorbeoconchaMelanopsidae

” mentioned in Hoeninghaus (1831: 142) [unavailable]

##### Horizon.

Burdigalian, early Miocene (?).

##### Locality.

“Dax”, France.

##### Remarks.

Nomen nudum. Grateloup (1838: 139) listed the name (as “*gratelupii*”) in synonymy of *Melanopsis aquensis* Grateloup, 1838. Wenz (1929: 2774) synonymized the name (as “*grateloupi*”) with “*Melanopsis major* Férussac, 1823”, which is not an available name.

#### Fagotia gravida

Taxon classificationAnimaliaSorbeoconchaMelanopsidae

Bourguignat, 1884

##### Original source.

Bourguignat 1884: 40.

##### Type locality.

“Rivières du bassin du lac Sabandja (Anatolie)” [rivers of the basin of Lake Sapanca], Turkey.

##### Remarks.

Note that Bourguignat denoted the authority as “Bourguignat, 1880”. Starobogatov et al. (1992: 63) considered the species as a junior synonym of *Fagotia* [= *Esperiana*] *gallandi* Bourguignat, 1884.

#### † Melanopsis groyei

Taxon classificationAnimaliaSorbeoconchaMelanopsidae

Pallary, 1916

##### Original source.

Pallary 1916: 78.

##### Type horizon.

Sparnacian, early Ypresian, Eocene.

##### Type locality.

“Entre St.-Germini et Carsoli” (Férussac 1823: 164) [between San Gemini and Carsoli], Italy.

##### Remarks.

Introduced for a part of the material of *Melanopsis buccinoidea* sensu Férussac, 1823 (pl. 8, fig. 3).

#### † Melanopsis guernei

Taxon classificationAnimaliaSorbeoconchaMelanopsidae

Brusina, 1902

##### Original source.

Brusina 1902: pl. 5, fig. 60.

##### Type horizon.

Middle Pannonian, late Miocene.

##### Type locality.

“Markuševec”, Croatia.

##### Types.

Milan et al. (1974: 91) indicated a holotype, but it is uncertain whether the specimen is part of the original type series and whether it was the only one Brusina had at hand (holotype by monotypy, Art. 73.1.2). The specimen is stored in the Croatian Natural History Museum, Zagreb, coll. no. 2500-146.

##### Remarks.

The name “*guerneri*” as mentioned in Wenz (1929: 2736) is an incorrect subsequent spelling.

#### Melanopsis etrusca var. guineensis

Taxon classificationAnimaliaSorbeoconchaMelanopsidae

Azpeitia Moros, 1929

##### Original source.

Azpeitia Moros 1929: 247, pl. 9, fig. 223.

##### Type locality.

“De las cercanías del Cabo San Juan en la Guinea española” [in the vicinity of Cabo San Juan], Equatorial Guinea.

##### Remarks.

Certainly not a Melanopsidae.

#### Melanopsis guiraoi

Taxon classificationAnimaliaSorbeoconchaMelanopsidae

Bourguignat, 1884

##### Original source.

Bourguignat 1884: 108.

##### Type locality.

“Prope Cehejin Prov. Murcica” (Brot 1879: 438) [near Cehegín, prov. Murcia], Spain.

##### Remarks.

Replacement name for *Melanopsis obesa* Brot, 1879, non Gassies, 1856.

#### Melanopsis hadiensis

Taxon classificationAnimaliaSorbeoconchaMelanopsidae

Pallary, 1928

##### Original source.

Pallary 1928a: 269, pl. 6, figs 11–14.

##### Type locality.

“Bel Hadi-Kenadsa (Sud Oranais)” [Bel Hadi, near Kenadsa], Algeria.

#### Microcolpia hagenmuelleriana

Taxon classificationAnimaliaSorbeoconchaMelanopsidae

Bourguignat, 1884

##### Original source.

Bourguignat 1884: 57.

##### Type locality.

“Le Danube près de Buda-Pesth, en Hongrie” [in the Danube river near Budapest], Hungary.

##### Remarks.

Bourguignat (1884) listed the name as “*Hagenmulleria*” on p. 51 but as “*Hagenmülleriana*” on p. 57. To my knowledge no later author has acted as First Reviser sensu Art. 24.2.3 and has mentioned both names and selected one as correct. Starobogatov et al. (1992: 65) considered the species as a junior synonym of *Microcolpia cornea* (Pfeiffer, 1828).

#### † Melanopsis entzi var. halavatsi

Taxon classificationAnimaliaSorbeoconchaMelanopsidae

Pavlovic, 1927

##### Original source.

Pavlović 1927: 68.

##### Type horizon.

Middle Pannonian, late Miocene.

##### Type locality.

“Карагача” [Karagača near Vrčin], Serbia.

##### Remarks.

From the writing in the original work it seems that Pavlović had apparently doubts about the validity of the taxon.

#### Melanopsis hammamensis

Taxon classificationAnimaliaSorbeoconchaMelanopsidae

Gassies, 1856

##### Original source.

Gassies 1856: 11, figs 9–10.

##### Type locality.

“L’Oued-el-Hammam [entre le Sig et Mascara]” [Oued el Hammam between Sig and Mascara], Algeria.

#### † Melanopsis handmanni

Taxon classificationAnimaliaSorbeoconchaMelanopsidae

Brusina, 1892 [invalid]

##### Original source.

Brusina 1892: 140.

##### Type horizon.

Early–middle Pannonian, late Miocene.

##### Type locality.

“Kotingbrunn; [...] Leobersdorf”, Austria.

##### Types.

The illustrated syntypes are stored in the Croatian Natural History Museum, Zagreb, coll. no. 2513-159/1-4 (Milan et al. 1974: 92).

##### Remarks.

Introduced as replacement name for the senior (!) homonym *Melanopsis fuchsi* Handmann, 1882, non Brusina, 1884. Wenz (1929: 2813–2814) synonymized *Melanopsis handmanni* as well as *Melanopsis fuchsi* Handmann, 1882 with *Melanopsis pygmaea*.

#### † Melanopsis handmanniana

Taxon classificationAnimaliaSorbeoconchaMelanopsidae

W. Fischer, 1996

##### Original source.

Fischer 1996a: 27, figs 3, 5–10.

##### Type horizon.

Pannonian, zone B–D, late Miocene.

##### Type locality.

“Leobersdorf”, Austria.

##### Types.

Fischer (1996a: 27) designated a lectotype (for *Melanopsis constricta* Handmann), which is stored in the collection of the Geological Survey Austria, Vienna; no number indicated.

##### Remarks.

Replacement name for *Melanopsis martiniana constricta* Handmann, 1887, non Brusina, 1878 (see Note 1).

#### † Melanopsis hantkeni

Taxon classificationAnimaliaSorbeoconchaMelanopsidae

Hofmann, 1870

##### Original source.

Hofmann 1870: 26, pl. 3, figs 5a–c.

##### Type horizon.

Egerian, late Oligocene–early Miocene.

##### Type locality.

“A zsily-völgyi [...], továbbá Valia-Aninossaban és Paren lui Marin-ban, Lupény mellett. [...] gyakori Buda és Esztergom között [...], jelesen a Miklóshegyen, Annavölgyen, Mogyoróson és Pomázon” [in the Zsily valley; also in Valea Aninoasa and Paren lui Marin (?) near Lupeni (Romania); between Buda and Esztergom, at Miklóshegy, Annavölgy, Mogyorós and Pomáz (Hungary)], Hungary.

#### † Melanopsis haranti

Taxon classificationAnimaliaSorbeoconchaMelanopsidae

De Laubrière & Carez, 1881

##### Original source.

De Laubrière and Carez 1881: 403, pl. 16, figs 1–2.

##### Type horizon.

Lutetian, Eocene.

##### Type locality.

“Brasles”, France.

##### Remarks.

Cossmann (1888: 279) and Wenz (1929: 2640) classified the species in the genus *Faunus* Montfort, 1810 (Pachychilidae).

#### Melanopsis costata var. harpa

Taxon classificationAnimaliaSorbeoconchaMelanopsidae

Westerlund, 1892

##### Original source.

Westerlund 1892: 199.

##### Type locality.

“Bei Sevilla im Guadalquivir” [near Sevilla, in the Guadalquivir river], Spain.

#### † Melanopsis harpula

Taxon classificationAnimaliaSorbeoconchaMelanopsidae

Neumayr in Neumayr & Paul, 1875

##### Original source.

Neumayr and Paul 1875: 38, pl. 7, fig. 1.

##### Type horizon.

Cernikian, Pliocene.

##### Type locality.

“Čaplathal bei Podwin” [Čaplja graben near Slavonski Brod], Croatia.

#### † Melanopsis hassiaca

Taxon classificationAnimaliaSorbeoconchaMelanopsidae

Sandberger, 1873

##### Original source.

Sandberger 1870–1875: 315.

##### Type horizon.

Early Rupelian, Oligocene.

##### Type locality.

“Grossalmerode [...]; Nordshausen” (Speyer 1870: 95–96), Germany.

##### Remarks.

Introduced for *Melanopsis subulata* sensu Speyer, 1870, non Sowerby, 1822 and *Melanopsis praerosa* [= *Melanopsis praemorsa*] sensu Speyer, 1870, non Linnaeus, 1758.

#### † Melanopsis hastata

Taxon classificationAnimaliaSorbeoconchaMelanopsidae

Neumayr in Neumayr & Paul, 1875

##### Original source.

Neumayr and Paul 1875: 40, pl. 7, figs 7–8.

##### Type horizon.

Cernikian, Pliocene.

##### Type locality.

“Gromačnik; [...] Slobodnica; [...] Sibin [Sibinj]; [...] Malino”, Croatia.

#### † Melanopsis haueri

Taxon classificationAnimaliaSorbeoconchaMelanopsidae

Handmann, 1882

##### Original source.

Handmann 1882: 558.

##### Type horizon.

Pannonian, zone D, late Miocene.

##### Type locality.

“Kottingbrunn [...] Ziegelei a”, Austria.

#### † Melanopsis haugi

Taxon classificationAnimaliaSorbeoconchaMelanopsidae

Popescu-Voitești, 1910

##### Original source.

Popescu-Voitești 1910: 360, pl. 21 (4), figs 4–4l.

##### Type horizon.

Lutetian, Eocene.

##### Type locality.

“Gropile Vulpilor près Titești” [Gropile Vulpilor (?) near Titești], Romania.

##### Remarks.

Wenz (1929: 2570) classified the species within the genus *Coptostylus* Sandberger, 1872 (Thiaridae).

#### † Melanopsis hazayi

Taxon classificationAnimaliaSorbeoconchaMelanopsidae

Brusina, 1903

##### Original source.

Brusina 1903: 112.

##### Type horizon.

Late Pleistocene–Holocene.

##### Type locality.

“Bischofsbad” [Püspökfürdő, Băile 1 Mai, Lake Pețea], Romania.

##### Remarks.

Neubauer et al. (2014d: 125) considered this taxon as a junior synonym of *Microcolpia parreyssii sikorai* (Brusina, 1903).

#### † Melania heberti

Taxon classificationAnimaliaSorbeoconchaMelanopsidae

Hantken, 1878

##### Original source.

Hantken 1878: 179–180, fig. 23.

##### Type horizon.

Late Santonian–early Campanian, late Cretaceous.

##### Type locality.

“[Ajka]” (p. 175, 178), Hungary.

##### Remarks.

Currently attributed to the melanopsid genus *Campylostylus* (Bandel and Riedel 1994: 21).

#### Melanopsis hebraica

Taxon classificationAnimaliaSorbeoconchaMelanopsidae

Bourguignat, 1884

##### Original source.

Bourguignat 1884: 131.

##### Type locality.

“Aïn-Saadi, près de Kaifa, en Syrie” [Aïn Saadi, in Haifa], Israel.

##### Remarks.

Heller et al. (2002: 601) considered the species as a junior synonym of *Melanopsis saulcyi* Bourguignat, 1853.

#### † Amphimelania heckneri

Taxon classificationAnimaliaSorbeoconchaMelanopsidae

Koch, 1917

##### Original source.

Koch 1917: 9, figs 1–6.

##### Type horizon.

Cernikian, Pliocene.

##### Type locality.

“U okolici Dubranca, [...] u Zagreb šumi, [...] kod Prvonožina” [near Dubranec, in the Zagreb woods, and at Prvonožina], Croatia.

#### † Melanopsis heeri

Taxon classificationAnimaliaSorbeoconchaMelanopsidae

Mayer-Eymar, 1887

##### Original source.

Mayer-Eymar 1887: 68, pl. 6, fig. 10.

##### Type horizon.

Chattian, Oligocene.

##### Type locality.

“Schloss Ralligen” [Ralligen Castle near Sigriswil], Switzerland.

##### Remarks.

Appeared first as a nomen nudum (as “*heerii*”) in Sandberger (1873: 341).

#### † Melanopsis heldreichi

Taxon classificationAnimaliaSorbeoconchaMelanopsidae

Neumayr, 1880

##### Original source.

Neumayr 1880b: 295, pl. 2, figs 2–3.

##### Type horizon.

Plio-Pleistocene.

##### Type locality.

“Zwischen Pylle und Antimachia; [...] beim Castell von Antimachia und in der näheren Umgebung des Dorfes Antimachia” [between Pýli and Antimácheia; near the citadel of and in the vicinity of the village Antimácheia, Kos Island], Greece.

#### Melania helena

Taxon classificationAnimaliaSorbeoconchaMelanopsidae

Von dem Busch in Philippi, 1847

##### Original source.

Philippi 1847: 170, pl. 4, fig. 4.

##### Type locality.

“Insula Java”, Indonesia.

##### Remarks.

Although described as *Melania* and not considered a Melanopsidae today, this species is included and briefly discussed here because it has been repeatedly considered a *Melanopsis*. The specimens had been apparently sent to Von dem Busch or Philippi by Meder, who had named the species “*Melanopsis Helena*”. The description, however, stems clearly from Von dem Busch. The species epithet is a noun in apposition and needs not to agree in gender with the generic name (Art. 31.2.1). Currently considered to belong in the marine genus *Clea* H. Adams & A. Adams, 1855 (Buccinidae) (e.g., Coelho et al. 2013).

#### Melanopsis heliophila

Taxon classificationAnimaliaSorbeoconchaMelanopsidae

Bourguignat, 1884

##### Original source.

Bourguignat 1884: 161.

##### Type locality.

“Dans l’oasis d’Aïn-Chair, à l’extrême sud saharien du Maroc” [in the oasis of Ain Chair (= Oued Mellah), at the far southern Sahara in Morocco], Marocco.

##### Remarks.

Note that Bourguignat denoted the authority as “Bourguignat, 1872”.

#### † Melania hellespontica

Taxon classificationAnimaliaSorbeoconchaMelanopsidae

Calvert & Neumayr, 1880

##### Original source.

Calvert and Neumayr 1880: 374, pl. 2, fig. 14.

##### Type horizon.

Late Sarmatian, Khersonian, late Miocene.

##### Type locality.

“Renkiöi” [north of İntepe], Turkey.

##### Remarks.

Considered to belong in the genus *Amphimelania* by Wenz (1929: 2874).

#### † Melanopsis hemimorpha

Taxon classificationAnimaliaSorbeoconchaMelanopsidae

Blanckenhorn, 1897

##### Original source.

Blanckenhorn 1897: 138, pl. 10, fig. 26.

##### Type horizon.

Plio-Pleistocene.

##### Type locality.

“Dschisr esch-Schurr, Dreissensiaschicht” [Jisr Ash-Shughur, *Dreissena* layer], Syria.

#### † Melanopsis hennersdorfensis

Taxon classificationAnimaliaSorbeoconchaMelanopsidae

W. Fischer, 1993

##### Original source.

Fischer 1993: 180, figs 1, 3.

##### Type horizon.

Pannonian, zone D, late Miocene.

##### Type locality.

“Tegelgrube der Fa. Wienerberger, Hennersdorf, NÖ” [claypit of the Wienerberger company at Hennersdorf, Lower Austria], Austria.

##### Types.

Geological Survey Austria, Vienna; no number indicated.

#### † Melanopsis hericarti

Taxon classificationAnimaliaSorbeoconchaMelanopsidae

Fontannes, 1881

##### Original source.

Fontannes 1881: 981, pl. 1, figs 5–6.

##### Type horizon.

Rupelian, Oligocene.

##### Type locality.

“Baume-Cornillane”, France.

#### Melanopsis hiera

Taxon classificationAnimaliaSorbeoconchaMelanopsidae

Bourguignat, 1884

##### Original source.

Bourguignat 1884: 121.

##### Type locality.

“Sources du Jourdain; [...] d’Aïn-el-Mellaha, dans la plaine du Bahr-el-Houlé; [...] lacs d’Homs et d’Antioche” [Sources of the Jordan; [...] Aïn Mallahah, in the plains of the Hula valley; [...] lakes Homs and Anuk (also as Amik)], Syria.

##### Remarks.

Introduced for *Melania costata* sensu Kobelt, 1880, non Olivier, 1804. Bourguignat attributed the authority to Letourneux, but there is no evidence that the description really derived from that author. Heller et al. (2005: 243) listed the species as a junior synonym of *Melanopsis costata* (Olivier, 1804) as well as of *Melanopsis saulcyi* Bourguignat, 1884.

#### † Fusus (Anura) decipiens var. hilarionis

Taxon classificationAnimaliaSorbeoconchaMelanopsidae

De Gregorio, 1880

##### Original source.

De Gregorio 1880: 89, pl. 7, fig. 61.

##### Type horizon.

Eocene.

##### Type locality.

“San Giovanni Ilarione”, Italy.

##### Remarks.

Considered a *Melanopsis* and a separate species by Quaggiotto and Mellini (2008: 44).

#### Melania (Amphimelania) hispanica

Taxon classificationAnimaliaSorbeoconchaMelanopsidae

Westerlund, 1898

##### Original source.

Westerlund 1898: 178.

##### Type locality.

“Albarracin”, Spain.

#### † Melanopsis hispidula

Taxon classificationAnimaliaSorbeoconchaMelanopsidae

Pallary, 1916

##### Original source.

Pallary 1916: 84.

##### Type horizon.

Pannonian, zone B–D, late Miocene.

##### Type locality.

“Leobersdorf” (Handmann 1887), Austria.

##### Remarks.

Replacement name for the junior homonym *Melanopsis turrita* Handmann, 1887, non Rossmässler, 1854.

#### † Melanopsis hoernesi

Taxon classificationAnimaliaSorbeoconchaMelanopsidae

Sandberger, 1872

##### Original source.

Sandberger 1870–1875: pl. 25, figs 32–32a.

##### Type horizon.

Burdigalian, early Miocene.

##### Type locality.

“St. Paul, Mandillot (Landes)”, France.

##### Remarks.

Plate 25 of Sandberger’s monograph was issued in 1872, while the description on p. 512 appeared in 1875 (Woodward 1906). Introduced for *Melanopsis buccinoides* [sic] sensu Grateloup, 1840, non Olivier, 1801, for which d’Orbigny (1852) had already introduced *Melanopsis subbuccinoides* as replacement name. Hence, *Melanopsis hoernesi* is a junior objective synonym of *Melanopsis subbuccinoides*. Sandberger attributed the authority to Mayer, apparently based on an “in schedis” determination.

#### Melanopsis costata var. hoernesi

Taxon classificationAnimaliaSorbeoconchaMelanopsidae

Blanckenhorn, 1897 [invalid]

##### Original source.

Blanckenhorn 1897: 130, pl. 10, figs 9–10.

##### Type horizon.

Pleistocene (?)–Recent.

##### Type locality.

“Fossil [..] bei Antakije. Lebend am unteren Orontes [...], desgleichen im Kara Su, einem nördlichen Zufluss des Sees von Antiochia und im Sadjür Su bei Aleppo” [as fossil near Antakya (Turkey). Alive in the lower Orontes (Turkey/Syria), as well as in the Karasu, a northern tributary of the Lake Anuk (Turkey), and in the Sājūr river near Aleppo (Syria)].

##### Remarks.

Junior homonym of *Melanopsis hoernesi* Sandberger, 1872 (note that latter is a junior objective synonym of *Melanopsis subbuccinoides* and thus invalid). Heller et al. (2005: 244) considered the species as a junior synonym of *Melanopsis costata* (Olivier, 1804).

#### Melania holandrii

Taxon classificationAnimaliaSorbeoconchaMelanopsidae

Pfeiffer, 1828

##### Original source.

Pfeiffer 1828: 47.

##### Type locality.

“Bei Kroatisch Feistritz, am Fusse des Berges Terglou in Illyrien” [near Bohinjska Bistrica, at the foot of Mt. Triglav], Slovenia.

##### Remarks.

Type species of *Holandriana* Bourguignat, 1884 and *Amphimelania* P. Fischer, 1885. The names “*holandri*” and “*hollandri*”, each occurring multiple time in the literature (e.g., Walderdorff 1864: 512; Brusina 1867: 85; Tschapeck 1881: 101), are incorrect subsequent spellings.

#### † Melanopsis hranilovici

Taxon classificationAnimaliaSorbeoconchaMelanopsidae

Brusina, 1897

##### Original source.

Brusina 1897: 12, pl. 5, fig. 12.

##### Type horizon.

Pannonian, late Miocene.

##### Type locality.

“Ad viam inter Sarajevo et Lukavica” [at the road between Sarajevo and Lukavica], Bosnia and Herzegovina.

##### Types.

Milan et al. (1974: 92) indicated a holotype, but it is uncertain whether the specimen was the only one Brusina had at hand (holotype by monotypy, Art. 73.1.2). The specimen is stored in the Croatian Natural History Museum, Zagreb, coll. no. 2985-631.

#### Melanopsis huidobroi

Taxon classificationAnimaliaSorbeoconchaMelanopsidae

Azpeitia Moros, 1929

##### Original source.

Azpeitia Moros 1929: 326, pl. 12, fig. 280.

##### Type locality.

“Guadalquivir en Lora del Río, Sevilla” [in the Guadalquivir river at Lora del Río, prov. Sevilla], Spain.

#### Melanopsis humilis

Taxon classificationAnimaliaSorbeoconchaMelanopsidae

Pallary, 1928

##### Original source.

Pallary 1928a: 255, pl. 4, figs 14–15.

##### Type locality.

“Fès et Meknès”, Morocco.

#### † Melanopsis hungarica

Taxon classificationAnimaliaSorbeoconchaMelanopsidae

Kormos, 1904

##### Original source.

Kormos 1904: 107, fig. 2.

##### Type horizon.

Late Pleistocene–early Holocene.

##### Type locality.

“Püspökfürdő” [Băile 1 Mai, Lake Pețea], Romania.

##### Remarks.

Neubauer et al. (2014d: 125) considered this taxon as a junior synonym of *Microcolpia parreyssii* (Philippi, 1847).

#### † Melanopsis hungarica

Taxon classificationAnimaliaSorbeoconchaMelanopsidae

Pallary, 1916 [invalid]

##### Original source.

Pallary 1916: 83.

##### Type horizon.

Transdanubian, Pannonian, late Miocene.

##### Type locality.

“Radmanest” (Fuchs 1870: 353) [Rădmănești], Romania.

##### Remarks.

Replacement name for *Melanopsis fuchsi* Brusina, 1884 non Handmann, 1882, which itself was introduced for *Melania costata* sensu Fuchs, 1870, non Olivier, 1804. It is a junior homonym of *Melanopsis hungarica* Kormos, 1904 (see *Melanopsis confusa* Strausz, 1941).

#### † Melanopsis hybostoma

Taxon classificationAnimaliaSorbeoconchaMelanopsidae

Brusina, 1874

##### Original source.

Brusina 1874: 31.

##### Type horizon.

Cernikian, Pliocene.

##### Type locality.

“Podvinje (Čaplja)” [Čaplja trench near Slavonski Brod], Croatia.

##### Remarks.

The names “*hibostoma*” and “*hypostoma*” as mentioned in Pană (2003: 324) and Macarovici (1940: 344) are incorrect subsequent spellings.

#### “ Melanopsis tingitana var. hybrida

Taxon classificationAnimaliaSorbeoconchaMelanopsidae

” mentioned in Brot (1874–1879: 442) [unavailable]

##### Locality.

“Morocco, Mogador; [...] Spanien, Andalousien” [Morocco, Essaouira; Spain, Andalusia].

##### Remarks.

Nomen nudum, listed in synonymy of “*Melanopsis tingitana* var. β” by Brot (1879), which is described briefly in Latin. He attributed the name to Morelet, but it remains unclear why - the only author mentioning a (nameless) variety of *Melanopsis tingitana* that matches the description by Brot is Mousson (1874).

#### † Melanopsis impressa

Taxon classificationAnimaliaSorbeoconchaMelanopsidae

Krauss, 1852

##### Original source.

Krauss 1852: 143, pl. 3, fig. 3.

##### Type horizon.

Ottnangian, middle Burdigalian, early Miocene.

##### Type locality.

“Kirchberg an der Iller” [Illerkirchberg], Germany.

#### † Melanopsis incerta

Taxon classificationAnimaliaSorbeoconchaMelanopsidae

Férussac, 1822

##### Original source.

Férussac 1819–1851: Mélanopsides fossiles, pl. 1 (1822), fig. 12.

##### Type horizon.

Sarmatian (sensu lato), middle–late Miocene.

##### Type locality.

“De Sestos” [Sestos, prov. Çanakkale], Turkey.

##### Remarks.

The species was also illustrated on pl. 2 of the “Mélanopsides fossiles” in the “Histoire naturelle” (Férussac 1819–1851) and on pl. 8 in Férussac (1823). However, Férussac obviously mixed up *Melanopsis incerta* with *Melanopsis daudebartii* and erroneously illustrated the latter of both species, which was already recognized by Deshayes when preparing the final explanations for the plates of the “Histoire naturelle”.

#### † Melanopsis incerta

Taxon classificationAnimaliaSorbeoconchaMelanopsidae

Fuchs, 1877 [invalid]

##### Original source.

Fuchs 1877: 14, pl. 2, figs 13–21.

##### Type horizon.

Pliocene.

##### Type locality.

“Megara”, Greece.

##### Remarks.

Junior homonym of *Melanopsis incerta* Férussac, 1822. Pallary (1920b: 110) introduced *Melanopsis revelata* as replacement name.

#### Melanopsis inconspicua

Taxon classificationAnimaliaSorbeoconchaMelanopsidae

Pallary, 1928

##### Original source.

Pallary 1928a: 261, pl. 4, figs 22–25.

##### Type locality.

“Sources tièdes un peu avant Kerrando, au S.-E. de Rich (grand Atlas oriental, versant sud)” [hot Springs just before Kerrando, southeast of Rich (High Atlas range, southern side)], Morocco.

#### Microcolpia inconspicua

Taxon classificationAnimaliaSorbeoconchaMelanopsidae

Starobogatov in Starobogatov et al., 1992

##### Original source.

Starobogatov et al. 1992: 66, fig. 3 (17).

##### Type locality.

“Фёслау, близ Вены” [Vöslau near Vienna], Austria.

##### Types.

Zoological Institute of Russian Academy of Sciences, St.-Petersburg; no number indicated.

#### † Melanopsis inconstans

Taxon classificationAnimaliaSorbeoconchaMelanopsidae

Neumayr, 1869

##### Original source.

Neumayr 1869: 356, pl. 11, figs 9–18.

##### Type horizon.

Langhian, middle Miocene.

##### Type locality.

“Miocic” [Miočić], Croatia.

##### Types.

Illustrated syntypes are stored at the Geological Survey Austria, Vienna, coll. no. 1869/01/3/1-10.

#### Melania (Amphimelania) induta

Taxon classificationAnimaliaSorbeoconchaMelanopsidae

Westerlund, 1898

##### Original source.

Westerlund 1898: 179.

##### Type locality.

“Muchalatka” [not found; stated to be on the Crimean Peninsula], Ukraine.

#### † Melanopsis inermis

Taxon classificationAnimaliaSorbeoconchaMelanopsidae

Handmann, 1882

##### Original source.

Handmann 1882: 554.

##### Type horizon.

Pannonian, zone D, late Miocene.

##### Type locality.

“Kottingbrunn [...] Ziegelei a”, Austria.

#### † Melanopsis klerici inermis

Taxon classificationAnimaliaSorbeoconchaMelanopsidae

Brusina, 1897 [invalid]

##### Original source.

Brusina 1897: 8.

##### Type horizon.

Pannonian, zone D–E, late Miocene.

##### Type locality.

“Begaljica”, Serbia.

##### Types.

Milan et al. (1974: 93) indicated a holotype, but it is uncertain whether the specimen was the only one Brusina had at hand (holotype by monotypy, Art. 73.1.2). The specimen is stored in the Croatian Natural History Museum, Zagreb, coll. no. 3020-666.

##### Remarks.

Junior homonym of *Melanopsis inermis* Handmann, 1882. Neubauer et al. (2014a) introduced *Melanopsis magyari* as replacement name.

#### † Boistelia inermis

Taxon classificationAnimaliaSorbeoconchaMelanopsidae

Jekelius, 1944 [invalid]

##### Original source.

Jekelius 1944: 137, pl. 57, figs 22–27.

##### Type horizon.

Early Pannonian, late Miocene.

##### Type locality.

“Turislav-Tal bei Soceni” [Turislav valley near Soceni], Romania.

##### Remarks.

Junior secondary homonym and junior synonym of *Melanopsis inermis* Handmann, 1882 (see discussion in Neubauer et al. 2014a: 463).

#### † Melanopsis inexspectata

Taxon classificationAnimaliaSorbeoconchaMelanopsidae

Willmann, 1981

##### Original source.

Willmann 1981: 193.

##### Type horizon.

Gurniati Formation, late Pliocene to early Pleistocene.

##### Type locality.

“Küstenaufschluß ca. 4 km südwestl. von Kardhamena/Kos (Profil K 3)” [section K 3, ca. 4 km southwest of Kardámaina, Kos Island], Greece.

##### Types.

Geological-Paleontological Institute, University of Kiel, Germany; no number indicated.

##### Remarks.

The name “*inexpectata*” as given by Kapan Yeşilyurt and Taner (2002: 107) is an incorrect subsequent spelling.

#### † Melanopsis buccinoidea var. γ) antiquua inflata

Taxon classificationAnimaliaSorbeoconchaMelanopsidae

Férussac, 1822

##### Original source.

Férussac 1819–1851: Mélanopsides fossiles, pl. 1 (1822), figs 1–3, 9.

##### Type horizon.

Eocene; late Villafranchian, early Pleistocene.

##### Type locality.

“Des environs d’Epernay. [...] Du dépôt situé entre St.-Germini et Carsoli” [surroundings of Épernay (France); from deposits between San Gemini and Carsoli (Italy)].

##### Remarks.

Introduced within the var. γ, to which Férussac attributed the term “antiquua”, which was probably not intended as species-group name (see introduction for a detailed discussion of the names introduced by Férussac 1823).

#### † Melanopsis pygmaea var. inflata

Taxon classificationAnimaliaSorbeoconchaMelanopsidae

Handmann, 1882 [invalid]

##### Original source.

Handmann 1882: 553.

##### Type horizon.

Pannonian, zone D, late Miocene.

##### Type locality.

“Kottingbrunn [...] Ziegelei a, Ziegelei c”, Austria.

##### Remarks.

Junior homonym of *Melanopsis buccinoidea inflata* Férussac, 1822. Wenz (1929: 2813) considered the taxon as a junior synonym of *Melanopsis pygmaea* Hörnes, 1856.

#### † Melanopsis pygmaea inflata

Taxon classificationAnimaliaSorbeoconchaMelanopsidae

Sauerzopf, 1952 [invalid]

##### Original source.

Sauerzopf 1952: 13, pl. 2, fig. 4.

##### Type horizon.

Pannonian, zone D–F, late Miocene.

##### Type locality.

“Stegersbach, Litzelsdorf, Olbendorf, Oberdorf”, Austria.

##### Remarks.

Junior homonym of *Melanopsis buccinoidea inflata* Férussac, 1822. Neubauer et al. (2014c: 28) considered the taxon as a junior synonym of *Melanopsis fuchsi* Handmann, 1882.

#### Melanopsis costata var. inflexa

Taxon classificationAnimaliaSorbeoconchaMelanopsidae

Pallary, 1939

##### Original source.

Pallary 1939: 90.

##### Type locality.

Not explicitly stated but probably the same as for the species (“Disjr ech Chogour” [Jisr Ash-Shughur], Syria).

#### Melanopsis costata var. infracincta

Taxon classificationAnimaliaSorbeoconchaMelanopsidae

Martens, 1874

##### Original source.

Martens 1874: 32, pl. 5, fig. 38.

##### Type locality.

“Quellen des Chabur bei Ras-el-Ain” [source of Chabur river near Ra’s al ‘Ayn], Syria.

#### Melanopsis magnifica var. ingens

Taxon classificationAnimaliaSorbeoconchaMelanopsidae

Pallary, 1928

##### Original source.

Pallary 1928a: 273, pl. 5, fig. 15.

##### Type locality.

“Aïn Chekef, près Fès” [Aïn Chkef, near Fes], Morocco.

#### Melanopsis parreyssi f. innodata

Taxon classificationAnimaliaSorbeoconchaMelanopsidae

Westerlund, 1886

##### Original source.

Westerlund 1886: 123.

##### Type locality.

“Bei Gross-Wardein in der schnellen Körös” [near Oradea in the Crișul Repede], Romania.

##### Remarks.

Based on a specimen of *Melanopsis parreyssii* figured in Kobelt (1880: 19, pl. 189, fig. 1909d). Neubauer et al. (2014d: 125) considered this taxon as a junior synonym of *Microcolpia parreyssii* (Philippi, 1847).

#### Melanopsis monteli var. innodata

Taxon classificationAnimaliaSorbeoconchaMelanopsidae

Pallary, 1936 [invalid]

##### Original source.

Pallary 1936: 61.

##### Type locality.

Not explicitly stated but probably the same as for the species (“L’Oued Sous, au pont des Aït Melloul, sur la route d’Agadir à Tiznit, à 14 kil. S. O. d’Agadir” [in the Oued Sous, at the bridge of Ait Melloul, at the road from Agadir to Tiznit, 14 km southwest of Agadir], Morocco).

##### Remarks.

Junior homonym of *Melanopsis parreyssii innodata* Westerlund, 1886 (see Note 1).

#### Melanopsis costata var. inodata

Taxon classificationAnimaliaSorbeoconchaMelanopsidae

Pallary, 1939 

##### Original source.

Pallary 1939: 91.

##### Type locality.

Not explicitly stated but probably the same as for the species (“Djishr ech Chegour” [Jisr Ash-Shughur], Syria).

#### Melanopsis insignis

Taxon classificationAnimaliaSorbeoconchaMelanopsidae

Locard, 1883

##### Original source.

Locard 1883a: 202.

##### Type locality.

“Samava” [As Samawah], Iraq.

##### Remarks.

Appeared first as a nomen nudum in Martens (1874: 43, 67). Locard (1883a) introduced the species by referring to “*Melanopsis Turcica* de Mousson [...] 1874, p. 44” [act. p. 49], which he obviously considered different from the variety described in Mousson (1874: 33).

#### † Melanopsis intermedia

Taxon classificationAnimaliaSorbeoconchaMelanopsidae

Rzehak, 1883

##### Original source.

Rzehak 1883: 43, pl. 2, figs 7a–c.

##### Type horizon.

*Oncophora* Beds, middle Burdigalian, early Miocene.

##### Type locality.

“Eibenschitz, [...] Oslawan” [Ivančice; Oslavany], Czech Republic.

##### Remarks.

Čtyroký (1972: 74) considered this taxon as a junior synonym of *Melanopsis impressa* Krauss, 1852.

#### † Melanopsis (Lyrcea) senatoria var. intermedia

Taxon classificationAnimaliaSorbeoconchaMelanopsidae

Handmann, 1887 [invalid]

##### Original source.

Handmann 1887: 19, pl. 2, fig. 14.

##### Type horizon.

Pannonian, zone B–D, late Miocene.

##### Type locality.

“Leobersdorf”, Austria.

##### Remarks.

Junior homonym of *Melanopsis intermedia* Rzehak, 1883.

#### Melanopsis mellalensis var. intermedia

Taxon classificationAnimaliaSorbeoconchaMelanopsidae

Pallary, 1928 [invalid]

##### Original source.

Pallary 1928b: 19, pl. 2, fig. 20.

##### Type locality.

“À Béni Mellal” [at Beni Mellal], Morocco.

##### Remarks.

Junior homonym of *Melanopsis intermedia* Rzehak, 1883.

#### † Melanopsis involuta

Taxon classificationAnimaliaSorbeoconchaMelanopsidae

Handmann, 1882

##### Original source.

Handmann 1882: 554.

##### Type horizon.

Pannonian, zone D, late Miocene.

##### Type locality.

“Kottingbrunn [...] Ziegelei a, Ziegelei c”, Austria.

#### Melanopsis iraqensis

Taxon classificationAnimaliaSorbeoconchaMelanopsidae

Pallary, 1939

##### Original source.

Pallary 1939: 89, pl. 4, figs 1, 2, 4, 5.

##### Type locality.

“Tappah, à 3 km Est de Belad Sindjar et ‘Ain Haglan” [Tappah, 3 km east of Sinjar and Ain Haglan (?)], Iraq.

#### Melanopsis irellii

Taxon classificationAnimaliaSorbeoconchaMelanopsidae

Pallary, 1920

##### Original source.

Pallary 1920b: 111.

##### Type locality.

“M. Mario” (Cerulli-Irelli 1914: 184), Italy.

##### Remarks.

Introduced for *Melanopsis nodosa* sensu Cerulli-Irelli, 1914, non Férussac, 1822. Girotti (1972: 232) considered *Melanopsis nodosa* sensu Cerulli-Irelli, 1914 a junior synonym of “*Melanopsis affinis* Férussac”, which is not an available name.

#### Melanopsis jordanica var. irregularis

Taxon classificationAnimaliaSorbeoconchaMelanopsidae

Mousson, 1861

##### Original source.

Mousson 1861: 149.

##### Type locality.

“Lac de Tiberias” [Sea of Galilee], Israel.

#### † Melanopsis irregularis

Taxon classificationAnimaliaSorbeoconchaMelanopsidae

Handmann, 1882 [invalid]

##### Original source.

Handmann 1882: 557.

##### Type horizon.

Pannonian, zone D, late Miocene.

##### Type locality.

“Kottingbrunn [...] Ziegelei a”, Austria.

##### Remarks.

Junior homonym of *Melanopsis jordanica irregularis* Mousson, 1861 (see Note 1).

#### Melanopsis isabelae

Taxon classificationAnimaliaSorbeoconchaMelanopsidae

Ahuir Galindo, 2016

##### Original source.

Ahuir Galindo 2016: 27.

##### Type locality.

“ln a Za river affluent, between Guefait and Hassi Blal”, Morocco.

##### Types.

Museo Malacologico di Cupra Marittima, Italy; no number indicated.

#### Melanopsis isseli

Taxon classificationAnimaliaSorbeoconchaMelanopsidae

Bourguignat, 1884

##### Original source.

Bourguignat 1884: 115.

##### Type locality.

“Dans le lac d’Accesa, près de Massa, en Toscane (Italie); aux environs d’Oran; [...] dans la vallée du Nahr-el-Kelb, près de Beyrouth” [in the Lago dell’Accesa near Massa Marittima, in Tuscany (Italy); in the surroundings of Oran (Algeria); in the cave of Nahr el-Kalb near Beirut (Lebanon)].

##### Remarks.

Introduced for *Melanopsis maroccana* sensu Bourguignat, 1864, non “Chemnitz” (which is not an available name; see there for details).

#### † Melanopsis martiniana var. italica

Taxon classificationAnimaliaSorbeoconchaMelanopsidae

Sandberger, 1872

##### Original source.

Sandberger 1870–1875: 666, 687, pl. 26, fig. 25.

##### Type horizon.

Tortonian–early Messinian, late Miocene.

##### Type locality.

“Stazzano”, Italy.

##### Remarks.

Plate 26 of Sandberger’s monograph appeared already in 1872, while the description on p. 666 (see also p. 687) was issued in 1875 (Woodward 1906). Harzhauser et al. (2015: 10) considered the taxon as a junior synonym of *Melanopsis narzolina* d’Archiac in Viquesnel, 1846.

#### † Melanopsis (Stilospirula) jansseni

Taxon classificationAnimaliaSorbeoconchaMelanopsidae

Anderson, 1964

##### Original source.

Anderson 1964: 208, pl. 13, fig. 118.

##### Type horizon.

Middle Miocene.

##### Type locality.

“Bachbett der Küningsbeke an der Königsmühle bei Dingden” [Küningsbeke brook at the Königsmühle near Dingden], Germany.

##### Remarks.

Probably not a Melanopsidae.

#### † Melanopsis jasonis

Taxon classificationAnimaliaSorbeoconchaMelanopsidae

Stache in Sandberger, 1871

##### Original source.

Sandberger 1870–1875: 133, pl. 19, fig. 12.

##### Type horizon.

Liburnian, Danian, Paleocene.

##### Type locality.

“Zablachie bei Sebenico in Dalmatien” [Zablaće near Šibenik], Croatia.

#### Melanopsis jebusitica

Taxon classificationAnimaliaSorbeoconchaMelanopsidae

Bourguignat, 1884

##### Original source.

Bourguignat 1884: 126.

##### Type locality.

“Dans quelques sources de la plaine de Jéricho (Syrie)” [in a few springs in the plains of Jericho], Palestine.

##### Remarks.

Bourguignat denoted the authority as “Letourneux, 1882”, but there is no evidence that the description really derived from that author.

#### † Melanopsis (Canthidomus) jekeliusi

Taxon classificationAnimaliaSorbeoconchaMelanopsidae

Gillet & Marinescu, 1971 [invalid]

##### Original source.

Gillet and Marinescu 1971: 55, pl. 22, figs 20–30.

##### Type horizon.

Transdanubian, Pannonian, late Miocene.

##### Type locality.

“Radmanest” (Fuchs 1870: 353) [Rădmănești], Romania.

##### Remarks.

Replacement name for the junior homonyms *Melanopsis fuchsi* Brusina, 1902, non Handmann, 1882 and *Melanopsis hungarica* Pallary, 1916, non Kormos, 1904, which were in turn introduced for *Melania costata* sensu Fuchs, 1870, non Olivier, 1804. However, already Strausz (1941) had introduced *Melanopsis confusa* as replacement name, which makes *Melanopsis jekeliusi* its junior objective synonym.

#### Melanopsis costata var. jordanica

Taxon classificationAnimaliaSorbeoconchaMelanopsidae

Roth, 1839

##### Original source.

Roth 1839: 25, pl. 2, figs 12–13.

##### Type locality.

“In flumine Jordano; in mari Galilaeo” [in Jordan river; in Sea of Galilee], Israel.

##### Remarks.

The name “*judaica*” as mentioned in Morelet (1880: 75) is an incorrect subsequent spelling.

#### Melanopsis jordanicensis

Taxon classificationAnimaliaSorbeoconchaMelanopsidae

Germain, 1921 [invalid]

##### Original source.

Germain 1921: 466, 496.

##### Remarks.

Unjustified emendation and therefore junior objective synonym of *Melanopsis jordanica* Roth, 1839.

#### † Melanopsis kacici

Taxon classificationAnimaliaSorbeoconchaMelanopsidae

Brusina, 1902

##### Original source.

Brusina 1902: pl. 6, figs 56–57.

##### Type horizon.

Middle Pannonian, late Miocene.

##### Type locality.

“Markuševec”, Croatia.

##### Types.

The syntypes are stored in the Croatian Natural History Museum, Zagreb; no number indicated (Milan et al. 1974: 92).

##### Remarks.

Wenz (1929: 2678) considered this taxon as a junior synonym of *Melanopsis bouei* Férussac, 1823.

#### † Melanopsis (Lyrcea) vindobonensis var. karagacensis

Taxon classificationAnimaliaSorbeoconchaMelanopsidae

Pavlović, 1927

##### Original source.

Pavlović 1927: 84, pl. 12, figs 1–2.

##### Type horizon.

Middle Pannonian, late Miocene.

##### Type locality.

“Карагач” [from Karagača near Vrčin], Serbia.

##### Types.

The illustrated syntype is stored in the Natural History Museum, Belgrade, coll. no. 226 (Milošević 1962: 23).

#### † “ Melanopsis kaltenbachi

Taxon classificationAnimaliaSorbeoconchaMelanopsidae

” mentioned in Mertin (1939: 254) [unavailable]

##### Horizon.

Heidelberg Formation, late Santonian, late Cretaceous.

##### Locality.

“Flugplatz Quedlinburg” [airfield at Quedlinburg], Germany.

##### Remarks.

Nomen nudum. Obviously a lapsus calami of *Odostomia kaltenbachi* Mertin, 1939.

#### † Melanopsis karici

Taxon classificationAnimaliaSorbeoconchaMelanopsidae

Pavlović, 1903

##### Original source.

Pavlović 1903: 156, pl. 3, figs 3–6.

##### Type horizon.

Middle Miocene.

##### Type locality.

“Из Бабиног Дола близу Скопља” (p. 155) [Babin Dol near Skopje], Macedonia.

##### Types.

The illustrated syntype is stored in the Natural History Museum, Belgrade, coll. no. 1448 (Milošević 1962: 24).

#### † Melanopsis (Lyrcaea) nobilis karpovi

Taxon classificationAnimaliaSorbeoconchaMelanopsidae

Gozhik in Gozhik & Datsenko, 2007

##### Original source.

Gozhik and Datsenko 2007: 94, pl. 88, fig. 5.

##### Type horizon.

Pliocene.

##### Type locality.

“Oз. Сасык” [Lake Sasyk], Ukraine.

##### Types.

Institute of Geological Sciences of the National Academy of Sciences of Ukraine, coll. no. 4602.

#### † Melanopsis katzeri

Taxon classificationAnimaliaSorbeoconchaMelanopsidae

Brusina, 1904

##### Original source.

Brusina 1904: 495, pl. 2, fig. 4, pl. 3, fig. 4.

##### Type horizon.

Langhian, middle Miocene.

##### Type locality.

“Varcar-Vakufa” [Mrkonjić Grad], Bosnia and Herzegovina.

#### † Melanopsis kerneri

Taxon classificationAnimaliaSorbeoconchaMelanopsidae

Kühn, 1946

##### Original source.

Kühn 1946: 75, textfig. 6.

##### Type horizon.

Late middle Eocene–early Oligocene.

##### Type locality.

“Monte Promina” [Promina Mountains], Croatia.

##### Types.

Geological-Paleontological Department, Natural History Museum Vienna, Austria, coll. no. 1869/0009/0013.

#### Melanopsis (Mesopotamia) khabourensis

Taxon classificationAnimaliaSorbeoconchaMelanopsidae

Pallary, 1939

##### Original source.

Pallary 1939: 102, pl. 5, figs 13–21.

##### Type locality.

“Sources de Khabour, dites Ras el ‘Ain” [source of Chabur river near Ra’s al ‘Ayn], Syria.

#### “ Melanopsis kindermanni

Taxon classificationAnimaliaSorbeoconchaMelanopsidae

Zelebor” mentioned in Brot (1874–1879: 429) [unavailable]

##### Locality.

Not indicated.

##### Remarks.

Nomen nudum, apparently based on an unpublished manuscript name from Zelebor listed in the synonymy list of *Melanopsis saulcyi* by Brot (1879).

#### † Melanopsis kispatici

Taxon classificationAnimaliaSorbeoconchaMelanopsidae

Brusina, 1897

##### Original source.

Brusina 1897: 10.

##### Type horizon.

Langhian, middle Miocene.

##### Type locality.

“Miočić”, Croatia.

##### Types.

Milan et al. (1974: 93) indicated a holotype, but it is uncertain whether the specimen is part of the original type series and whether it was the only one Brusina had at hand (holotype by monotypy, Art. 73.1.2). The specimen is stored in the Croatian Natural History Museum, Zagreb, coll. no. 2970-616.

##### Remarks.

Neubauer et al. (2013: 135) considered this taxon as a junior synonym of *Melanopsis lyrata* Neumayr, 1869.

#### † Melanopsis (Canthidomus) kittli

Taxon classificationAnimaliaSorbeoconchaMelanopsidae

Handmann, 1887

##### Original source.

Handmann 1887: 32, pl. 7, fig. 14.

##### Type horizon.

Pannonian, zone B–D, late Miocene.

##### Type locality.

“Leobersdorf”, Austria.

##### Remarks.

Wenz (1929: 2675) considered the taxon as a junior synonym of *Melanopsis bouei* Férussac, 1823.

#### † Melanoptychia klecakiana

Taxon classificationAnimaliaSorbeoconchaMelanopsidae

Bourguignat, 1880

##### Original source.

Bourguignat 1880: 37.

##### Type horizon.

Langhian, middle Miocene.

##### Type locality.

“Vallée de la Cettina” [Cetina river valley], Croatia.

##### Remarks.

The taxon is not included in the Fossilium Catalogus of Wenz (1929).

#### † Melanopsis kleinii

Taxon classificationAnimaliaSorbeoconchaMelanopsidae

Kurr, 1856

##### Original source.

Kurr 1856: 42.

##### Type horizon.

Middle–late Burdigalian, early Miocene.

##### Type locality.

“Zwiefalten und Andelfingen” (Dunker 1848: 159), Germany.

##### Remarks.

Introduced for *Melanopsis praerosa* [= *Melanopsis praemorsa*] sensu Dunker, 1848 and Klein, 1852, non Linnaeus, 1758. The name “*kleini*” as mentioned in Wenz (1929: 2762) is an incorrect subsequent spelling.

#### † Melanopsis klerici

Taxon classificationAnimaliaSorbeoconchaMelanopsidae

Brusina, 1893

##### Original source.

Brusina 1893: 203 (Serbian part), 53 (Italian part).

##### Type horizon.

Pannonian, zone D–E, late Miocene.

##### Type locality.

“Begaljica”, Serbia.

##### Types.

The syntypes are stored in the Croatian Natural History Museum, Zagreb; no number indicated (Milan et al. 1974: 93).

#### Melania (Melanopsis) kotschyi

Taxon classificationAnimaliaSorbeoconchaMelanopsidae

Von dem Busch in Philippi, 1847

##### Original source.

Philippi 1847: 175, pl. 4, fig. 11.

##### Type locality.

“Persepolis Persiae”, Iran.

#### † Melanopsis kottingbrunnensis

Taxon classificationAnimaliaSorbeoconchaMelanopsidae

Handmann, 1882

##### Original source.

Handmann 1882: 559.

##### Type horizon.

Pannonian, zone D, late Miocene.

##### Type locality.

“Kottingbrunn [...] Ziegelei a”, Austria.

##### Remarks.

Wenz (1929: 2741) considered the taxon as a junior synonym of *Melanopsis haueri* Handmann, 1882.

#### † “ Melanopsis kozlovskii

Taxon classificationAnimaliaSorbeoconchaMelanopsidae

” mentioned in Bogachev (1938: 13, 33, 53) [unavailable]

##### Horizon.

Miocene.

##### Locality.

“Tori bei Borshomi” [Tori near Borjomi], Georgia.

##### Remarks.

The name was only mentioned in a species list by Bogachev without description or illustration.

#### † Melanopsis krambergeri

Taxon classificationAnimaliaSorbeoconchaMelanopsidae

Brusina, 1892

##### Original source.

Brusina 1892: 142.

##### Type horizon.

Middle Pannonian, late Miocene.

##### Type locality.

“Markuševec”, Croatia.

##### Types.

Milan et al. (1974: 93) indicated collection numbers for “syntypes” illustrated in Brusina (1902), but it is uncertain whether the specimens were part of the original type series. They are stored in the Croatian Natural History Museum, Zagreb, coll. no. 2499-145/1-2.

##### Remarks.

Appeared first as a nomen nudum in Brusina (1884a: 138).

#### † Amphimelania krambergeri

Taxon classificationAnimaliaSorbeoconchaMelanopsidae

Brusina, 1902

##### Original source.

Brusina 1902: pl. 5, figs 10–11.

##### Type horizon.

Cernikian, Pliocene.

##### Type locality.

“Hrastina”, Croatia.

##### Types.

Milan et al. (1974: 58) indicated a holotype, but it is uncertain whether the specimen was the only one Brusina had at hand (holotype by monotypy, Art. 73.1.2). The specimen is stored in the Croatian Natural History Museum, Zagreb, coll. no. 2481-127.

##### Remarks.

Appeared first as a nomen nudum in Gorjanović-Kramberger (1892: 109).

#### Melanella krapinensis

Taxon classificationAnimaliaSorbeoconchaMelanopsidae

Bourguignat, 1884

##### Original source.

Bourguignat 1884: 18.

##### Type locality.

“Dans le canal de sortie des eaux thermales de Krapina-Toeplitz, en Croatie” [in the outlet channel of the thermal waters at Krapinske toplice], Croatia.

##### Remarks.

Bourguignat denoted the authority as “Letourneux, 1879”, but there is no evidence that the description really derived from that author.

#### † Melanopsis kupensis

Taxon classificationAnimaliaSorbeoconchaMelanopsidae

Fuchs, 1870

##### Original source.

Fuchs 1870b: 544, pl. 22, figs 3–4.

##### Type horizon.

Middle Pannonian, late Miocene.

##### Type locality.

“Kúp”, Hungary.

#### † Melanopsis kurdica

Taxon classificationAnimaliaSorbeoconchaMelanopsidae

Brusina, 1902

##### Original source.

Brusina 1902: pl. 7, figs 8–11.

##### Type horizon.

Portaferrian (Pannonian Basin), late Miocene–Pliocene.

##### Type locality.

“Kurd”, Hungary.

##### Types.

The illustrated syntypes are stored in the Croatian Natural History Museum, Zagreb, coll. no. 2535-181/1-4 (Milan et al. 1974: 93).

#### † Melanopsis kuzmici

Taxon classificationAnimaliaSorbeoconchaMelanopsidae

Brusina, 1907

##### Original source.

Brusina 1907: 197.

##### Type horizon.

Late Burdigalian–early Langhian, early–middle Miocene.

##### Type locality.

Not indicated by Brusina (1905, 1907), apart from “probably Sinj Basin”, Croatia.

##### Remarks.

Appeared first as a nomen nudum in Brusina (1905: 32).

#### † Melanopsis lactacea

Taxon classificationAnimaliaSorbeoconchaMelanopsidae

Cossmann, 1888

##### Original source.

Cossmann 1888: 283, pl. 11, figs 12–13.

##### Type horizon.

Thanetian, Paleocene.

##### Type locality.

“Chenay [...]; Jonchery [...]; Châlons-sur-Vesle”, France.

#### Melanopsis laevigata

Taxon classificationAnimaliaSorbeoconchaMelanopsidae

Lamarck, 1822

##### Original source.

Lamarck 1822: 168.

##### Type locality.

“Dans les rivières des îles de l’Archipel” [it is unknown which Archipel Lamarck referred to].

##### Remarks.

The species first appeared without name on a plate in the “Tableau encyclopédique et méthodique, vol. 3” by Lamarck (1797). The captions for most plates of the three volumes work were prepared by Bory de Saint-Vincent, but apparently not before 1824, and added to the “Tableau encyclopédique et méthodique, vol. 1” of Bruguière (1791; see note on pp. 83–84). In the captions, Bory de Saint-Vincent also referred to the “Histoire naturelle” (vol. 6, part 2) by Lamarck (1822), where the name first occurred.

#### Melania holandri var. laevigata

Taxon classificationAnimaliaSorbeoconchaMelanopsidae

Rossmässler, 1839

##### Original source.

Rossmässler 1838–1844: Heft 4 (10), 37, pl. 50, figs 664–667.

##### Type locality.

“Gradaschza und Ringelsza [bei Laibach]; aus einem Mühlbache bei Nassenfuss in Unterkrain” [in Gradaščica and Ringelsza (?) brooks near Ljubljana; from a mill creek near Mokronog] (the remaining localities mentioned cannot be assigned to the present variety without doubt), Slovenia.

##### Remarks.

Note that Rossmässler (1839) seems to have listed *Melania holandri* in synonymy of this variety, but from the text it is clear that the list is intended to refer to the species as a whole.

#### † Melanopsis laevigata

Taxon classificationAnimaliaSorbeoconchaMelanopsidae

Łomnicki, 1886 [invalid]

##### Original source.

Łomnicki 1886: 76.

##### Type horizon.

Late Miocene.

##### Type locality.

“Wyczółki (wrzynka kol. trans. na zachodnio-południowym końcu wsi)” [Goncharivka, at southwestern end of village], Ukraine.

##### Remarks.

Junior homonym of *Melanopsis laevigata* Lamarck, 1822. Wenz (1928a: 119) introduced *Melanopsis lomnickii* as replacement name.

#### Melanopsis tunetana var. laevigata

Taxon classificationAnimaliaSorbeoconchaMelanopsidae

Pallary, 1912 [invalid]

##### Original source.

Pallary 1912: 20, [unnumbered plate], figs 31–33.

##### Type locality.

“Nefta”, Tunisia.

##### Remarks.

Junior homonym of *Melanopsis laevigata* Lamarck, 1822.

#### Melanopsis penchinati var. laevigata

Taxon classificationAnimaliaSorbeoconchaMelanopsidae

Pallary, 1924 [invalid]

##### Original source.

Pallary 1924: 248, pl. 15, fig. 12.

##### Type locality.

“Eaux de Mathen, Alhama de Aragon” [waters of Mathen (?), Alhama de Aragón], Spain.

##### Remarks.

Junior homonym of *Melanopsis laevigata* Lamarck, 1822. The taxon appears at infrasubspecific rank in the plate captions of the original publication.

#### † Melanopsis laevis

Taxon classificationAnimaliaSorbeoconchaMelanopsidae

Stoliczka, 1860

##### Original source.

Stoliczka 1860: 484, pl. 1, figs 4a–b.

##### Type horizon.

Late Turonian, late Cretaceous.

##### Type locality.

“Neualpe im Russbachthal” [Neualm near Russbach am Pass Gschütt], Austria.

#### Melanopsis vespertina var. laevis

Taxon classificationAnimaliaSorbeoconchaMelanopsidae

Bourguignat, 1884 [invalid]

##### Original source.

Bourguignat 1884: 125.

##### Type locality.

“Ruisseau de l’île d’Ivice, aux Baléares” [stream on the island of Ibiza], Spain.

##### Remarks.

Junior homonym of *Melanopsis laevis* Stoliczka, 1860.

#### Melanopsis douttei var. laevis

Taxon classificationAnimaliaSorbeoconchaMelanopsidae

Pallary, 1920 [invalid]

##### Original source.

Pallary 1920c: 150.

##### Type locality.

“Oued Masmouda; Ruisseau de Bab Hadid” [Oued Masmouda (?); stream Bab Hadid (?)], Morocco.

##### Remarks.

Junior homonym of *Melanopsis laevis* Stoliczka, 1860.

#### Melanopsis foleyi var. laevis

Taxon classificationAnimaliaSorbeoconchaMelanopsidae

Pallary, 1928 [invalid]

##### Original source.

Pallary 1928a: 270.

##### Type locality.

“Aïn Mélias, près de Figuig” [Ain Melias near Figuig], Algeria.

##### Remarks.

Junior homonym of *Melanopsis laevis* Stoliczka, 1860.

#### Melanopsis lamarckii

Taxon classificationAnimaliaSorbeoconchaMelanopsidae

Potiez & Michaud, 1838

##### Original source.

Potiez and Michaud 1838: 351, pl. 31, figs 5–6.

##### Type locality.

“Les rivières de Madagascar?” [rivers of Madagascar?].

##### Remarks.

Köhler and Glaubrecht (2010: 873) considered the taxon as a junior synonym of *Madagasikara spinosa* (Lamarck, 1822) (Pachychilidae).

#### † Melanopsis lamarckii

Taxon classificationAnimaliaSorbeoconchaMelanopsidae

Deshayes, 1862 [invalid]

##### Original source.

Deshayes 1861–1864: 472, pl. 31, figs 25–26.

##### Type horizon.

Calcaire de Montabuzard, middle–late Burdigalian.

##### Type locality.

“Damery”, France.

##### Remarks.

Junior homonym of *Melanopsis lamarckii* Potiez & Michaud, 1838. Cossmann (1888: 279) and Wenz (1929: 2640) classified the species in the genus *Faunus* Montfort, 1810 (Pachychilidae). The name “*lamarcki*” as mentioned in Wenz is an incorrect subsequent spelling.

#### Melanopsis lamberti

Taxon classificationAnimaliaSorbeoconchaMelanopsidae

Souverbie, 1872

##### Original source.

Souverbie 1872: 148.

##### Type locality.

“Baye du Sud” [Baie Sud], New Caledonia.

##### Remarks.

Brot (1879: 463) considered the taxon as a junior synonym of *Melanopsis mariei* Crosse, 1869.

#### Melanopsis olivieri var. lamellata

Taxon classificationAnimaliaSorbeoconchaMelanopsidae

Bourguignat, 1884

##### Original source.

Bourguignat 1884: 98.

##### Type locality.

“Environs de Constantinople” [surroundings of Istanbul], Turkey.

#### Melanopsis lampra

Taxon classificationAnimaliaSorbeoconchaMelanopsidae

Bourguignat, 1884

##### Original source.

Bourguignat 1884: 132.

##### Type locality.

“Belus, près de Saint-Jean-d’Acre, en Syrie” [in the Na’aman river, near Acre], Israel.

##### Remarks.

Heller et al. (2002: 596) considered the species as a junior synonym of *Melanopsis costata* (Olivier, 1804). Heller et al. (2005: 244) in turn treated it as an accepted name.

#### † Melanopsis lanceolata

Taxon classificationAnimaliaSorbeoconchaMelanopsidae

Neumayr in Neumayr & Paul, 1875

##### Original source.

Neumayr and Paul 1875: 39, pl. 7, figs 5, 15.

##### Type horizon.

Cernikian, Pliocene.

##### Type locality.

“Slobodnica; [...] Sibin [Sibinj]; [...] Malino; [...] Gromačnik; [...] Čapla [Čaplja trench near Slavonski Brod]; [...] Cigelnik”, Croatia.

#### † Melanopsis (Melanoptychia) langhofferi

Taxon classificationAnimaliaSorbeoconchaMelanopsidae

Pavlović, 1927

##### Original source.

Pavlović 1927: 61, pl. 7, figs 7–8.

##### Type horizon.

Middle Pannonian, late Miocene.

##### Type locality.

“Из карагачких пескова” [from the sands of Karagača near Vrčin], Serbia.

##### Types.

The illustrated syntype is stored in the Natural History Museum, Belgrade, coll. no. 211 (Milošević 1962: 23).

#### † Melanopsis lanzaeana

Taxon classificationAnimaliaSorbeoconchaMelanopsidae

Brusina, 1874

##### Original source.

Brusina 1874: 34.

##### Type horizon.

Langhian, middle Miocene.

##### Type locality.

“Ribarić; Turjake [Turjaci]”, Croatia.

##### Types.

Milan et al. (1974: 94) indicated a holotype, but it is uncertain whether the specimen is part of the original type series and whether it was the only one Brusina had at hand (holotype by monotypy, Art. 73.1.2). The specimen is stored in the Croatian Natural History Museum, Zagreb, coll. no. 2982-628.

##### Remarks.

The name “*lanzae*” as mentioned in Olujić (1999: 20, 49) is an incorrect subsequent spelling.

#### Melanopsis latastei

Taxon classificationAnimaliaSorbeoconchaMelanopsidae

Letourneux & Bourguignat, 1887

##### Original source.

Letourneux and Bourguignat 1887: 158.

##### Type locality.

“Des environs de Nefta; [...] dans l’Oued Gabès et aux alentours de Tozer” [surroundings of Nefta; in the Oued Gabès and around Tozeur], Tunisia.

#### † Melanopsis laubrierei

Taxon classificationAnimaliaSorbeoconchaMelanopsidae

Carez, 1879

##### Original source.

Carez 1879: 637, pl. 12, figs 1–5.

##### Type horizon.

Early–middle Eocene.

##### Type locality.

“La Maladrerie (Brasles); Gland (Aisne)”, France.

#### † Melanopsis lebedai

Taxon classificationAnimaliaSorbeoconchaMelanopsidae

Lueger, 1980

##### Original source.

Lueger 1980: 104.

##### Type horizon.

Pannonian, zone D, late Miocene.

##### Type locality.

“Föllig d1 [...] und d2” [Föllig hill near Großhöflein], Austria.

##### Types.

Geological-Paleontological Department, Natural History Museum Vienna, Austria. The number Lueger provided is not the collection number but the acqusition number, which could be used to trace back the object in our database.

#### Melanopsis lecointrei

Taxon classificationAnimaliaSorbeoconchaMelanopsidae

Pallary, 1918

##### Original source.

Pallary 1918: 150.

##### Type locality.

“Taza, Ain en nsa, source est, chaude” [Taza, in the hot spring Ain en nsa], Morocco.

#### Melania holandri var. legitima

Taxon classificationAnimaliaSorbeoconchaMelanopsidae

Rossmässler, 1839

##### Original source.

Rossmässler 1838–1844: Heft 4 (10), 37, pl. 50, figs 662–663.

##### Type locality.

“In der Laibach; in der Save” [Ljubljanica and Sava rivers] (the remaining localities mentioned cannot be assigned to the present variety without doubt), Slovenia.

#### “ Melanopsis lembergensis

Taxon classificationAnimaliaSorbeoconchaMelanopsidae

” mentioned in Brot (1870: 310) and Brot (1874: 370) [unavailable]

##### Locality.

“Lemberg, Galizien” [Lviv], Ukraine.

##### Remarks.

Nomen nudum, apparently based on an unused manuscript name. It appears only in the synonymy list of *Hemisinus* [now *Esperiana*] *acicularis* in Brot (1870) with the authority “Schröter” as well in Brot (1874) with the authority “Parreyss”.

#### Melanopsis lentiginosa

Taxon classificationAnimaliaSorbeoconchaMelanopsidae

Reeve, 1860

##### Original source.

Reeve 1860: Section *Melanopsis*, pl. 3, figs 9a–b.

##### Type locality.

“New Caledonia” (France) [no locality indicated].

##### Remarks.

Brot (1879: 444) considered the taxon as a junior synonym of *Melanopsis frustulum* Morelet, 1857.

#### † Melanopsis (Martinia) leobersdorfensis

Taxon classificationAnimaliaSorbeoconchaMelanopsidae

Handmann, 1887

##### Original source.

Handmann 1887: 23, pl. 3, fig. 10.

##### Type horizon.

Pannonian, zone B–D, late Miocene.

##### Type locality.

“Leobersdorf”, Austria.

##### Remarks.

Wenz (1929: 2719) considered the taxon as a junior synonym of *Melanopsis fossilis* (Gmelin, 1791).

#### † Melanopsis lepavinensis

Taxon classificationAnimaliaSorbeoconchaMelanopsidae

Brusina, 1897

##### Original source.

Brusina 1897: 9, pl. 6, figs 17–20.

##### Type horizon.

*Congeria rhomboidea* Zone, Portaferrian, late Miocene.

##### Type locality.

“Lepavina”, Croatia.

##### Types.

Milan et al. (1974: 94) indicated a holotype, but it is uncertain whether the specimen is part of the original type series and whether it was the only one Brusina had at hand (holotype by monotypy, Art. 73.1.2). The specimen is stored in the Croatian Natural History Museum, Zagreb, coll. no. 3003-649.

#### Fagotia letourneuxi

Taxon classificationAnimaliaSorbeoconchaMelanopsidae

Bourguignat, 1884

##### Original source.

Bourguignat 1884: 45.

##### Type locality.

“Rivière entre Plaski et Ostaria, et dans la Save entre Agram et Sissek” [river between Plaški and Oštarije, and in the Sava river between Zagreb and Sisak], Croatia.

##### Remarks.

Note that Bourguignat denoted the authority as “Bourguignat, 1879”. Starobogatov et al. (1992: 60) considered the species as a junior synonym of *Fagotia* [= *Esperiana*] *acroxia* Bourguignat, 1884

#### Melanella letourneuxi

Taxon classificationAnimaliaSorbeoconchaMelanopsidae

Bourguignat, 1884

##### Original source.

Bourguignat 1884: 25.

##### Type locality.

“Dans la rivière d’Ogulin, en Croatie; [...] dans la Migliaska, près de Sérajewo” [in the river at Ogulin (Croatia); in the Miljacka river near Sarajevo (Bosnia and Herzegovina)].

##### Remarks.

Appeared first as a nomen nudum in Servain (1884: 379) [January]. Bourguignat (1884) [May] denoted the authority as “Bourguignat, 1879”.

#### Melanopsis letourneuxi

Taxon classificationAnimaliaSorbeoconchaMelanopsidae

Bourguignat, 1884

##### Original source.

Bourguignat 1884: 116.

##### Type locality.

“Dans la source et la rivière de la Moulouiah, à l’ouest de Lalla-Maghnia” [in the source and the river Moulouya, west of Maghnia], Morocco or Algeria.

##### Remarks.

Bourguignat denoted the authority as “Bourguignat, 1872, et Letourneux”, apparently referring to Letourneux (1876b). That work is not available to me, but after Crosse (1877: 33) that record is a nomen nudum. It appears also as nomen nudum in Letourneux (1876a: 48). In addition, Bourguignat listed “Bourguignat. Spec. nov. Moll., no. 198, 1878”, which displays the second part of “Species novissimae Molluscorum” (Bourguignat 1876), but which was never published.

#### Microcolpia letourneuxi

Taxon classificationAnimaliaSorbeoconchaMelanopsidae

Bourguignat, 1884

##### Original source.

Bourguignat 1884: 64.

##### Type locality.

“La Save, au-dessous d’Agram” [Sava river below Zagreb], Croatia.

#### Melanopsis crenocarinata var. leucostoma

Taxon classificationAnimaliaSorbeoconchaMelanopsidae

Moricand, 1841

##### Original source.

Moricand 1841: 62.

##### Type locality.

“Rio de Pedra Branca, procince de Bahia” [Pedra Branca river, province Bahia], Brazil.

##### Remarks.

Although not explicitly stated, this variety was apparently considered to belong to the new genus *Verena* by Adams and Adams (1854) (Thiaridae), of which *Melanopsis crenocarinata* is the type species (see Nuttall 1990: 253).

#### † Melanopsis libanensis

Taxon classificationAnimaliaSorbeoconchaMelanopsidae

Delpey, 1940

##### Original source.

Delpey 1940: 93, fig. 66, pl. 5, fig. 5.

##### Type horizon.

Aptian, early Cretaceous.

##### Type locality.

“Bekfaya; [...] Mar Abda” [Bikfaïya; Mâr Abdâ], Lebanon.

##### Remarks.

Probably not a Melanopsidae.

#### † Melanopsis liburnica

Taxon classificationAnimaliaSorbeoconchaMelanopsidae

Stache, 1889

##### Original source.

Stache 1889: 145, pl. 3, figs 25–27, pl. 5, fig. 12.

##### Type horizon.

Danian, Paleocene.

##### Type locality.

“Corgnale, Divacca und unteres Gaberg-Gehänge” [Lokev, Divača, and lower slopes of Mt. Vremščica], Italy.

##### Remarks.

Appeared first as a nomen nudum in Stache (1880: 199).

#### † Hemisinus lignitarius

Taxon classificationAnimaliaSorbeoconchaMelanopsidae

Tausch, 1886

##### Original source.

Tausch 1886: 8, pl. 1, figs 24–27.

##### Type horizon.

Late Santonian–early Campanian, late Cretaceous.

##### Type locality.

“Ajka”, Hungary.

##### Remarks.

Oppenheim (1892: 757) and Bandel and Riedel (1994: 20) considered the species as a junior synonym of *Esperiana obeloides* (Tausch, 1886).

#### † Melanopsis limbata

Taxon classificationAnimaliaSorbeoconchaMelanopsidae

Pallary, 1916

##### Original source.

Pallary 1916: 83.

##### Type horizon.

Pannonian, zone D, late Miocene.

##### Type locality.

“Kottingbrunn [...] Ziegelei a” (Handmann 1882: 559), Austria.

##### Remarks.

Replacement name for *Melanopsis scalaris* Handmann, 1882, non Gassies, 1856. Wenz (1929: 2741) considered the taxon as a junior synonym of *Melanopsis haueri* Handmann, 1882.

#### Melanopsis costata var. lineata

Taxon classificationAnimaliaSorbeoconchaMelanopsidae

Pallary, 1939

##### Original source.

Pallary 1939: 91, pl. 5, fig. 33.

##### Type locality.

“Djishr ech Chegour” [Jisr Ash-Shughur], Syria.

##### Remarks.

Pallary erroneously gave the name as “*unicincta*” in the plate captions. Heller and Sivan (2002a: 49) considered the taxon as a junior synonym of *Melanopsis multiformis* Blanckenhorn, 1897.

#### † Pseudofagotia lineata

Taxon classificationAnimaliaSorbeoconchaMelanopsidae

Anistratenko, 1993

##### Original source.

Anistratenko 1993: 72, textfig. 2.

##### Type horizon.

Duab Beds, middle to late Kimmerian, Pliocene.

##### Type locality.

“Окр. с. Мокви, Очамчирский р-н” [near the village Mok’vi, Ochamchirskiy rayon], Georgia.

##### Types.

Schmalhausen Institute of Zoology of National Academy of Sciences of Ukraine, Kiev; no number indicated.

##### Remarks.

Type species of the genus *Pseudofagotia* Anistratenko, 1993.

#### Melanopsis lineolata

Taxon classificationAnimaliaSorbeoconchaMelanopsidae

Gassies, 1857

##### Original source.

Gassies 1857: 276, pl. 9, figs 9–10.

##### Type locality.

“La rivière Balade” [in the river Balade], New Caledonia.

##### Remarks.

Gassies (1861) apparently considered *Melania lineolata* Gray in Griffith & Pidgeon, 1833 as a *Melanopsis*, which resulted in secondary homonymy for his species *Melanopsis lineoloata* Gassies, 1857. Thus, he introduced *Melanopsis livida* as a replacement name. Secondary homonymy, however, is not given anymore, since *Melania lineolata* Gray was recently shown to belong to the genus *Cerithidea* Swainson, 1840 (Potamididae) by Reid (2014). (Note, moreover, that *Melania lineolata* Gray is a junior homonym of *Melania lineolata* Wood, 1828). *Melanopsis livida* is a junior objective synonym of *Melanopsis lineolata* Gassies (see also Art. 59.3). Brot (1879: 444) considered the taxon as a junior synonym of *Melanopsis frustulum* Morelet, 1857.

#### Melanopsis liocephala

Taxon classificationAnimaliaSorbeoconchaMelanopsidae

Pallary, 1936

##### Original source.

Pallary 1936: 55, pl. 3, fig. 5.

##### Type locality.

“Guefaït (Maroc oriental)”, Morocco.

#### Melanopsis lirata

Taxon classificationAnimaliaSorbeoconchaMelanopsidae

Gassies, 1869

##### Original source.

Gassies 1869: 77.

##### Type locality.

“Prope Noumea” [near Nouméa], New Caledonia.

##### Remarks.

Brot (1879: 444) considered the taxon as a junior synonym of *Melanopsis frustulum* Morelet, 1857.

#### Melanopsis livida

Taxon classificationAnimaliaSorbeoconchaMelanopsidae

Gassies, 1861 [invalid]

##### Original source.

Gassies 1861: 290, pl. 7, fig. 9.

##### Type locality.

“Le Diahot à Balade” [in the Diahot river at Balade], New Caledonia.

##### Remarks.

Replacement name for the presumed secondary homonym *Melanopsis lineolata* Gassies, 1856, non *Melania lineolata* Gray in Griffith & Pidgeon, 1833. Secondary homonymy, however, is not given anymore, since *Melania lineolata* Gray was recently shown to belong to the genus *Cerithidea* Swainson, 1840 (Potamididae) by Reid (2014) (see also Art. 59.3). (Note, moreover, that *Melania lineolata* Gray is a junior homonym of *Melania lineolata* Wood, 1828). *Melanopsis livida* is a junior objective synonym of *Melanopsis lineolata* Gassies. Brot (1879: 444) considered the taxon as a junior synonym of *Melanopsis frustulum* Morelet, 1857.

#### Melanopsis locardi

Taxon classificationAnimaliaSorbeoconchaMelanopsidae

Blanckenhorn, 1897

##### Original source.

Blanckenhorn 1897: 132, pl. 10, fig. 15.

##### Type horizon.

Pleistocene (?)–Recent.

##### Type locality.

“Lebend im See von Antiochia. Halbfossil bei Selemije” [living in Lake Anuk (also as Amik) (Turkey); subfossil near As Salamīyah (Syria)].

#### Fagotia locardiana

Taxon classificationAnimaliaSorbeoconchaMelanopsidae

Bourguignat, 1884

##### Original source.

Bourguignat 1884: 39.

##### Type locality.

“Dans le lac Sabandja, près d’Ismidt (Anatolie)” [Lake Sapanca near İzmit], Turkey.

##### Remarks.

Note that Bourguignat denoted the authority as “Bourguignat, 1882”.

#### † Melanopsis lomnickii

Taxon classificationAnimaliaSorbeoconchaMelanopsidae

Wenz, 1928

##### Original source.

Wenz 1928a: 119.

##### Type horizon.

Badenian, middle Miocene.

##### Type locality.

“Wyczółki (wrzynka kol. trans. na zachodnio-południowym końcu wsi)” (Łomnicki 1886: 76) [Goncharivka, at southwestern end of village], Ukraine.

##### Remarks.

Replacement name for *Melanopsis laevigata* Łomnicki, 1886, non Lamarck, 1792.

#### † Melanopsis nodosa var. longa

Taxon classificationAnimaliaSorbeoconchaMelanopsidae

Deshayes in Férussac, 1839

##### Original source.

Férussac 1819–1851: Mélanopsides fossiles, pl. 2, fig. 8 (altered caption by Deshayes delivered with Livraison 29; see introduction for details).

##### Type horizon.

Late Miocene.

##### Type locality.

“D’Athènes” [Athens], Greece.

#### † Microcolpia longa

Taxon classificationAnimaliaSorbeoconchaMelanopsidae

Gozhik in Gozhik & Datsenko, 2007

##### Original source.

Gozhik and Datsenko 2007: 95, pl. 95, figs 1–5, pl. 97, fig. 3.

##### Type horizon.

Late Pleistocene.

##### Type locality.

“Aллювия V террасы р. Дунай у с. Нагорное” [Alluvial terrace V of the Danube river near Nagornoye], Ukraine.

##### Types.

Institute of Geological Sciences of the National Academy of Sciences of Ukraine, coll. no. 6461.

##### Remarks.

Originally the gender was indicated as masculine (“*longus*”), but *Microcolpia* is feminine, which is why the name must be “*longa*”.

#### † Melanopsis (Lyrcea) senatoria var. longata

Taxon classificationAnimaliaSorbeoconchaMelanopsidae

Handmann, 1887

##### Original source.

Handmann 1887: 19, pl. 2, figs 12–13.

##### Type horizon.

Pannonian, zone B–D, late Miocene.

##### Type locality.

“Leobersdorf”, Austria.

#### † Melanopsis longirostris

Taxon classificationAnimaliaSorbeoconchaMelanopsidae

Pallary, 1920

##### Original source.

Pallary 1920b: 112.

##### Type horizon.

Pontian (sensu stricto), late Miocene.

##### Type locality.

“Babadjan” (Andrusov 1909: captions of pl. 5) [Babadzhan], Azerbaijan.

##### Remarks.

Based on the record of “*Melanopsis* sp.” in Andrusov (1909: 88, pl. 5, figs 1–2).

#### Melanopsis cesari var. longirostris

Taxon classificationAnimaliaSorbeoconchaMelanopsidae

Pallary, 1928 [invalid]

##### Original source.

Pallary 1928a: 255.

##### Type locality.

“Dans les séguias de Beni Abbès” [in the irrigation channel of Beni Abbès], Algeria.

##### Remarks.

Junior homonym of *Melanopsis longirostris* Pallary, 1920.

#### † Melanopsis (Canthidomus) macrosculpturata longitesta

Taxon classificationAnimaliaSorbeoconchaMelanopsidae

Papp, 1953

##### Original source.

Papp 1953a: 110, pl. 23, figs 16–18.

##### Type horizon.

Gelasian, early Pleistocene.

##### Type locality.

“Pyrgos (Elis)”, Greece.

##### Types.

Museum of Palaeontology and Geology of the University of Athens; no number indicated.

#### † Melanopsis praemorsa var. longopyrulata

Taxon classificationAnimaliaSorbeoconchaMelanopsidae

Sacco, 1895

##### Original source.

Sacco 1895: 9, pl. 1, fig. 15.

##### Type horizon.

Early Messinian, late Miocene.

##### Type locality.

“S. Marzano Oliveto”, Italy.

##### Remarks.

Wenz (1929: 2733) considered the taxon as a junior synonym of *Melanopsis fusulatina* Sacco, 1895.

#### Melanopsis lorcana

Taxon classificationAnimaliaSorbeoconchaMelanopsidae

Guirao, 1854

##### Original source.

Guirao 1854: 32.

##### Type locality.

“In rivulo Rambla de Viznaga et in Pantano de Puentes non procul Lorca in Regno Murcico” [in the river Rambla de Viznaga and in Pantano de Puentes, not far from Lurca, prov. Murcia], Spain.

#### † Melanopsis (Canthidomus) lorentheyi

Taxon classificationAnimaliaSorbeoconchaMelanopsidae

Andrusov, 1909

##### Original source.

Andrusov 1909: 82, 158, pl. 4, figs 11–20.

##### Type horizon.

Pontian (sensu stricto), late Miocene.

##### Type locality.

“Babadjan” [Babadzhan], Azerbaijan.

#### † Melanopsis lorioli

Taxon classificationAnimaliaSorbeoconchaMelanopsidae

Locard, 1893

##### Original source.

Locard 1893: 187, pl. 9, fig. 22.

##### Type horizon.

Middle–late Burdigalian, early Miocene.

##### Type locality.

“Vernier, près Genève”, Switzerland.

#### Melanopsis lortetiana

Taxon classificationAnimaliaSorbeoconchaMelanopsidae

Locard, 1883

##### Original source.

Locard 1883a: 271, pl. 23, figs 50–51.

##### Type locality.

“Lac d’Antioche” [Lake Anuk (also as Amik)], Turkey.

##### Remarks.

Appears as “*Lorteti*” in Locard’s remarks on p. 272, which is apparently based on a typesetting error.

#### † Melanopsis lozanici

Taxon classificationAnimaliaSorbeoconchaMelanopsidae

Brusina, 1893

##### Original source.

Brusina 1893: 201 (Serbian part), 34 (Italian part), pl. 2, fig. 6.

##### Type horizon.

Pannonian, zone D–E, late Miocene.

##### Type locality.

“Ripanj”, Serbia.

##### Types.

Milan et al. (1974: 94) indicated a holotype, but it is uncertain whether the specimen is part of the original type series and whether it was the only one Brusina had at hand (holotype by monotypy, Art. 73.1.2). The specimen is stored in the Croatian Natural History Museum, Zagreb, coll. no. 3639-1279/1.

#### † Melanopsis lucanensis

Taxon classificationAnimaliaSorbeoconchaMelanopsidae

Neubauer in Neubauer et al., 2011

##### Original source.

Neubauer et al. 2011: 206, pl. 1, figs 10–12.

##### Type horizon.

Early Langhian, middle Miocene.

##### Type locality.

“Sinj, Lučane [= Sutina] section”, Croatia.

##### Types.

Geological-Paleontological Department, Natural History Museum Vienna, Austria, coll. no. 2010/0042/0001.

#### † ? Amphimelania lucida

Taxon classificationAnimaliaSorbeoconchaMelanopsidae

Cossmann, 1886

##### Original source.

Cossmann 1886: 228, pl. 10, fig. 7.

##### Type horizon.

Eocene.

##### Type locality.

“Houdan”, France.

##### Remarks.

This taxon was considered to belong to the marine genus *Nozeba* Iredale, 1915 (Iravadiidae) by Ponder (1984: 56).

#### “ Melanopsis lucio

Taxon classificationAnimaliaSorbeoconchaMelanopsidae

” mentioned in Erber (1868: 904) [unavailable]

##### Locality.

“Rhodus” [Rhodes island, no locality indicated], Greece.

##### Remarks.

Nomen nudum. Erber attributed the authority to Mousson. Given as “*Luciae*” in Paetel (1888).

#### Melanopsis dircaeana var. luctuosa

Taxon classificationAnimaliaSorbeoconchaMelanopsidae

Pallary, 1939

##### Original source.

Pallary 1939: 88, pl. 4, fig. 19, pl. 6, fig. 35.

##### Type locality.

“Lac de Homs”, Syria.

#### † Melanopsis ludwigi

Taxon classificationAnimaliaSorbeoconchaMelanopsidae

Speyer, 1870

##### Original source.

Speyer 1870: 97, pl. 15, figs 3–4.

##### Type horizon.

Early Rupelian, Oligocene.

##### Type locality.

“Grossalmerode” (Ludwig 1865: 71), Germany.

##### Remarks.

Replacement name for *Melania costata* Ludwig, 1865, non Olivier, 1804.

#### † Melanopsis lushani

Taxon classificationAnimaliaSorbeoconchaMelanopsidae

d’Archiac in Viquesnel, 1846

##### Original source.

Viquesnel 1846: 265, pl. 16, fig. 1.

##### Type horizon.

Pannonian, late Miocene.

##### Type locality.

“Entre Koulana et Lus-han” [between Koulana and Lus-han, S of Drin river mouth], Albania.

##### Remarks.

Originally the name was introduced as “*Lus-Hani*”; the name “*Lushami*” as mentioned in Chenu (1859: 297) is an incorrect subsequent spelling. Wenz (1929: 2715) considered the taxon as a junior synonym of *Melanopsis fossilis* (Gmelin, 1791).

#### Melanopsis iraqensis var. lutea

Taxon classificationAnimaliaSorbeoconchaMelanopsidae

Pallary, 1939

##### Original source.

Pallary 1939: 89.

##### Type locality.

“‘Ain Haglan” [not found], Iraq.

#### Melanopsis (Sistaniana) lutensis

Taxon classificationAnimaliaSorbeoconchaMelanopsidae

Starobogatov & Izzatullaev, 1985

##### Original source.

Starobogatov and Izzatullaev 1985: 35, fig. 5.

##### Type locality.

“Пустыне Деште-Лут” [Dasht-e Loot desert], Iran.

##### Types.

Zoological Institute of Russian Academy of Sciences, St.-Petersburg; no number indicated.

#### Melanopsis costata var. luteopsis

Taxon classificationAnimaliaSorbeoconchaMelanopsidae

Germain, 1921

##### Original source.

Germain 1921: 494, pl. 20, figs 9–10.

##### Type locality.

“Lac d’Homs”, Syria.

##### Remarks.

Heller et al. (2005: 248) considered the variety as a junior synonym of *Melanopsis saulcyi* Bourguignat, 1853.

#### † Melanopsis lyra

Taxon classificationAnimaliaSorbeoconchaMelanopsidae

Matheron, 1842

##### Original source.

Matheron 1842: 293, pl. 37, figs 8–10.

##### Type horizon.

Early Campanian, Cretaceous.

##### Type locality.

“Les Martigues”, France.

##### Remarks.

Sandberger (1871: 88) attributed the species to the genus *Paludomus* Swainson, 1840 (Paludomidae).

#### † Melanopsis (Canthidomus) lyrata

Taxon classificationAnimaliaSorbeoconchaMelanopsidae

Neumayr, 1869 [invalid]

##### Original source.

Neumayr 1869: 358, pl. 11, fig. 8.

##### Type horizon.

Langhian, middle Miocene.

##### Type locality.

“Ribaric” [Ribarić], Croatia.

##### Types.

Illustrated syntype is stored at the Geological Survey Austria, Vienna, coll. no. 1869/01/6.

##### Remarks.

Unlike stated by Neubauer et al. (2016b: 25), the name *Melanopsis lyrata* Neumayr, 1869 [June] is deemed to be a junior homonym of *Melanopsis lirata* Gassies, 1869 [January] after Art. 58.2. Pallary (1916: 85) introduced *Melanopsis dissimilis* as replacement name. Wenz (1929: 2698) was aware of the homonymy issue and synonymized this species with *Melanopsis cylindracea* Brusina, 1874.

#### Microcolpia mabilliana

Taxon classificationAnimaliaSorbeoconchaMelanopsidae

Bourguignat, 1884

##### Original source.

Bourguignat 1884: 59.

##### Type locality.

“La rivière entre Plaski et Ostaria (Croatie)” [river between Plaški and Oštarije], Croatia.

##### Remarks.

Starobogatov et al. (1992: 65) considered the species as a junior synonym of *Microcolpia acicularis* (Férussac, 1823).

#### † Melania macedonica

Taxon classificationAnimaliaSorbeoconchaMelanopsidae

Burgerstein, 1877

##### Original source.

Burgerstein 1877: 248, pl. 3, figs 13–16.

##### Type horizon.

Late Miocene.

##### Type locality.

“Ueskueb” [Skopje], Macedonia (not Zvezdan in Serbia as given by Wenz 1929).

##### Remarks.

Considered to belong in the genus *Amphimelania* by Brusina (1893: 63) and Wenz (1929: 2875).

#### Melanopsis macilenta

Taxon classificationAnimaliaSorbeoconchaMelanopsidae

Pallary, 1928

##### Original source.

Pallary 1928b: 17, pl. 2, figs 15–17.

##### Type locality.

“Beni Mellal, dans l’oued Taguenout” [Beni Mellal, in the Oued Taguenout (?)], Morocco.

#### “ Melania macilenta

Taxon classificationAnimaliaSorbeoconchaMelanopsidae

Ziegler” mentioned in Brot (1874–1879: 13) [unavailable]

##### Locality.

“In der Muhr” [in the river Mur], Austria.

##### Remarks.

Nomen nudum, apparently based on an unused manuscript name from Parreyss listed in synonymy of “*Melania Holandri*” [sic] by Brot (1874).

#### † Melanopsis (Canthidomus) macrosculpturata

Taxon classificationAnimaliaSorbeoconchaMelanopsidae

Papp, 1953

##### Original source.

Papp 1953a: 109, pl. 23, figs 13–18.

##### Type horizon.

Gelasian, early Pleistocene.

##### Type locality.

“Pyrgos”, Greece.

##### Types.

Museum of Palaeontology and Geology of the University of Athens; no number indicated.

#### Melanopsis macrostoma

Taxon classificationAnimaliaSorbeoconchaMelanopsidae

Bourguignat, 1884

##### Original source.

Bourguignat 1884: 157.

##### Type locality.

“Le Guadalquivir aux environs de Séville” [in the Guadalquivir river, around Sevilla], Spain.

#### Melanopsis maculata

Taxon classificationAnimaliaSorbeoconchaMelanopsidae

Lea, 1837

##### Original source.

Lea 1837: 82, pl. 19, fig. 75.

##### Type locality.

“Peru” [no locality indicated].

##### Remarks.

Junior synonym of *Hemisinus osculati* (Villa in Villa & Villa, 1854) (Thiaridae) after Ramírez et al. (2003: 272).

#### † Melanopsis magna

Taxon classificationAnimaliaSorbeoconchaMelanopsidae

Pallary, 1916 [invalid]

##### Original source.

Pallary 1916: 78.

##### Type horizon.

Burdigalian, early Miocene (?).

##### Type locality.

“Dax”, France.

##### Remarks.

Pallary (1916) erroneously treated “*magna*” as available name attributed to Férussac (1823), who solely used the word as descriptive term (Latin “big”), which he applied to many of his varieties. The name became nevertheless available from Pallary (1916) who associated the name with an illustration in Férussac (1823: pl. 7, fig. 16). The illustrated specimen is, however, the holotype (by monotypy) of *Melanopsis dufourii* Férussac, 1822 (Férussac used the same plates in both of his works and thus Pallary actually referred to the specimen illustrated as *Melanopsis dufourii* in the “Histoire naturelle”; see also introduction and Table 1 for details of Férussac’s publications). Therefore, *Melanopsis magna* Pallary, 1916 is a junior objective synonym of *Melanopsis dufourii*.

#### Melanopsis letourneuxi var. magna

Taxon classificationAnimaliaSorbeoconchaMelanopsidae

Pallary, 1928 [invalid]

##### Original source.

Pallary 1928a: 273, pl. 5, fig. 6.

##### Type locality.

“Berguent” [Aïn Beni Mathar], Morocco.

##### Remarks.

Junior homonym of *Melanopsis magna* Pallary, 1916.

#### † Melanopsis (Lyrcaea) [sic] magna

Taxon classificationAnimaliaSorbeoconchaMelanopsidae

Lubenescu, 1985 [invalid]

##### Original source.

Lubenescu 1985: 78, pl. 2, fig. 8.

##### Type horizon.

Middle Pannonian, late Miocene.

##### Type locality.

“Village Cut, district Alba”, Romania.

##### Types.

Institute of Geology and Geophysics, University of Bucharest, coll. no. 14655.

##### Remarks.

Originally the gender was indicated as masculine (“*magnus*”), but *Melanopsis* is feminine, which is why the name must be “*magna*”. Junior homonym of *Melanopsis magna* Pallary, 1916.

#### Melanopsis magnifica

Taxon classificationAnimaliaSorbeoconchaMelanopsidae

Bourguignat, 1884

##### Original source.

Bourguignat 1884: 152.

##### Type locality.

“Environs de Fez” [surroundings of Fes], Morocco.

#### † Melanopsis magyari

Taxon classificationAnimaliaSorbeoconchaMelanopsidae

Neubauer, Harzhauser, Georgopoulou, Mandic & Kroh, 2014

##### Original source.

Neubauer et al. 2014a: 457.

##### Type horizon.

Middle Pannonian, late Miocene.

##### Type locality.

“Begaljica” (Brusina 1897: 8), Serbia.

##### Types.

Milan et al. (1974: 93) indicated a holotype, but it is uncertain whether the specimen was the only one Brusina had at hand when describing *Melanopsis klerici inermis* (holotype by monotypy, Art. 73.1.2). The specimen is stored in the Croatian Natural History Museum, Zagreb, coll. no. 3020-666.

##### Remarks.

Replacement name for *Melanopsis klerici inermis* Brusina, 1897, non Handmann, 1882 (see Note 1).

#### † “ Melanopsis major

Taxon classificationAnimaliaSorbeoconchaMelanopsidae

Férussac, 1823” mentioned in Wenz (1929: 2773) [unavailable]

##### Horizon.

Burdigalian, early Miocene.

##### Locality.

“De Mandillot, près de Dax” [Mandillot, near Dax], France.

##### Remarks.

This name has appeared several times in the literature (e.g., Bronn 1848: 717; Wenz 1929: 2773), but it is based on an error. Férussac (1823: 154) actually described *Melanopsis dufourii* var. ε and listed “*Fossilis*, major. Férussac” in synonymy referring to plate 1 of the “Mélanopsides fossiles” of his “Histoire naturelle” (Férussac 1819–1851; see also introduction for details about this work). However, in the captions of plate 1, it is given as “*MelanopsisDufourii*, var. ε). *Fossilis*, *maxima*”. Obviously neither *major* nor *maxima* was intended as species-group name by Férussac. Later, the name would have been a junior homonym of *Melania buccinoidea major* Grateloup, 1838.

#### † Melanopsis buccinoidea var. major

Taxon classificationAnimaliaSorbeoconchaMelanopsidae

Grateloup, 1838

##### Original source.

Grateloup 1838: 146, pl. 4, figs 52–53.

##### Type horizon.

Burdigalian, early Miocene.

##### Type locality.

“Dax. [...] Mandillot; à Saint-Paul”, France.

#### Melanopsis acicularis var. major

Taxon classificationAnimaliaSorbeoconchaMelanopsidae

Rossmässler, 1839 [invalid]

##### Original source.

Rossmässler 1838–1844: 41, pl. 50, figs 673–675.

##### Type locality.

“In der Lachina bei Tschernembl, [...] in dem Bug” [in the Lahinja river near Črnomelj (Slovenia) and in the Bug river (Ukraine)].

##### Remarks.

Junior homonym of *Melanopsis buccinoidea major* Grateloup, 1838 (see Note 1).

#### Melanopsis maroccana var. major

Taxon classificationAnimaliaSorbeoconchaMelanopsidae

Bourguignat, 1864 [invalid]

##### Original source.

Bourguignat 1864: 260.

##### Type locality.

“Mostaghanem” [Mostaganem], Algeria.

##### Remarks.

Junior homonym of *Melanopsis buccinoidea major* Grateloup, 1838 (see Note 1).

#### Melanopsis cariosa var. major

Taxon classificationAnimaliaSorbeoconchaMelanopsidae

Bourguignat, 1884 [invalid]

##### Original source.

Bourguignat 1884: 151.

##### Type locality.

“Dans les aqueducs de Séville et dans le Guadalquivir” [in the aqueducts of Sevilla and in the Guadalquivir river], Spain.

##### Remarks.

Junior homonym of *Melanopsis buccinoidea major* Grateloup, 1838 (see Note 1).

#### Melanopsis macrostoma var. major

Taxon classificationAnimaliaSorbeoconchaMelanopsidae

Bourguignat, 1884 [invalid]

##### Original source.

Bourguignat 1884: 157.

##### Type locality.

“Le Guadalquivir aux environs de Séville” [in the Guadalquivir river, around Sevilla], Spain.

##### Remarks.

Junior homonym of *Melanopsis buccinoidea major* Grateloup, 1838 (see Note 1).

#### Melanopsis subscalaris var. major

Taxon classificationAnimaliaSorbeoconchaMelanopsidae

Bourguignat, 1884 [invalid]

##### Original source.

Bourguignat 1884: 108.

##### Type locality.

“Environs de Fez (Maroc)” [surroundings of Fes], Morocco.

##### Remarks.

Junior homonym of *Melanopsis buccinoidea major* Grateloup, 1838 (see Note 1).

#### Melanopsis pseudoferussaci var. major

Taxon classificationAnimaliaSorbeoconchaMelanopsidae

Pallary, 1899 [invalid]

##### Original source.

Pallary 1899: 140.

##### Type locality.

“Environs de Tétouan” [surrondings of Tétouan], Morocco.

##### Remarks.

Junior homonym of *Melanopsis buccinoidea major* Grateloup, 1838 (see Note 1).

#### Melanopsis tingitana var. major

Taxon classificationAnimaliaSorbeoconchaMelanopsidae

Pallary, 1899 [invalid]

##### Original source.

Pallary 1899: 147.

##### Type locality.

“L’O.[ued] Ida ou Guert, près de Mogador (p.); O. Aït Ouadel (p. 163)” [river (?) Ida Ou Gourdh at Essaouira;] , Morocco.

##### Remarks.

Junior homonym of *Melanopsis buccinoidea major* Grateloup, 1838 (see Note 1).

#### † Melanopsis hammamensis var. major

Taxon classificationAnimaliaSorbeoconchaMelanopsidae

Pallary, 1901 [invalid]

##### Original source.

Pallary 1901a: 178, pl. 2, fig. 21.

##### Type horizon.

Pleistocene.

##### Type locality.

“De l’oued El Biod (Géryville)” [Oudeï el Biod near El Bayadh], Algeria.

##### Remarks.

Junior homonym of *Melanopsis buccinoidea major* Grateloup, 1838 (see Note 1).

#### Melanopsis vespertina var. major

Taxon classificationAnimaliaSorbeoconchaMelanopsidae

Pallary, 1911 [invalid]

##### Original source.

Pallary 1911: 130, [unnumbered plate], fig. 3.

##### Type locality.

“Tout près d’Oudjda, à 4 kilom. S.-E., sourdent les belles sources de Sidi-Yahia qui alimentent une véritable oasis, puis la ville d’Oudjda, et vont finalement se déverser dans l’oued Isly” [near Oujda, 4 km southeast, at the sources of Sidi Yahya that feed an oasis and the city of Oujda, and ultimately will flow into the Oued Isly], Morocco.

##### Remarks.

Junior homonym of *Melanopsis buccinoidea major* Grateloup, 1838 (see Note 1).

#### Melanopsis tunetana var. major

Taxon classificationAnimaliaSorbeoconchaMelanopsidae

Pallary, 1912 [invalid]

##### Original source.

Pallary 1912: 20, fig. 30.

##### Type locality.

“A Tozeur, sur les bords du Chott [Djerid?]” [banks of the Chott el Djérid at Tozeur], Tunisia.

##### Remarks.

Junior homonym of *Melanopsis buccinoidea major* Grateloup, 1838 (see Note 1).

#### Melanopsis douttei var. major

Taxon classificationAnimaliaSorbeoconchaMelanopsidae

Pallary, 1920 [invalid]

##### Original source.

Pallary 1920c: 150.

##### Type locality.

“La Makina”, Morocco.

##### Remarks.

Junior homonym of *Melanopsis buccinoidea major* Grateloup, 1838 (see Note 1).

#### Melanopsis ricardi var. major

Taxon classificationAnimaliaSorbeoconchaMelanopsidae

Pallary, 1920 [invalid]

##### Original source.

Pallary 1920c: 141, pl. 3, fig. 7.

##### Type locality.

“Aïn Allou”, Morocco.

##### Remarks.

Junior homonym of *Melanopsis buccinoidea major* Grateloup, 1838 (see Note 1).

#### Melanopsis penchinati var. major

Taxon classificationAnimaliaSorbeoconchaMelanopsidae

Pallary, 1924 [invalid]

##### Original source.

Pallary 1924: 248, pl. 15, fig. 10.

##### Type locality.

“Eaux de Mathen, Alhama de Aragon” [waters of Mathen (?), Alhama de Aragón], Spain.

##### Remarks.

Junior homonym of *Melanopsis buccinoidea major* Grateloup, 1838 (see Note 1).

#### Melanopsis turrita var. major

Taxon classificationAnimaliaSorbeoconchaMelanopsidae

Pallary, 1924 [invalid]

##### Original source.

Pallary 1924: 247, pl. 15, fig. 5.

##### Type locality.

“Guadalquivir”, Spain.

##### Remarks.

Junior homonym of *Melanopsis buccinoidea major* Grateloup, 1838 (see Note 1).

#### † Melanopsis gorceixi var. major

Taxon classificationAnimaliaSorbeoconchaMelanopsidae

Magrograssi, 1928 [invalid]

##### Original source.

Magrograssi 1928: 259, pl. 6, fig. 12.

##### Type horizon.

Plio-Pleistocene.

##### Type locality.

“Coo: molto frequente in tutte e due le zone fossilifere” [Kos island: very common in both areas rich in fossils, i.e., between Antimáchei and Pýli and in the northeast of the island, near Ágios Fokás], Greece.

##### Remarks.

Junior homonym of *Melanopsis buccinoidea major* Grateloup, 1838 (see Note 1).

#### Melanopsis seurati var. major

Taxon classificationAnimaliaSorbeoconchaMelanopsidae

Pallary, 1928 [invalid]

##### Original source.

Pallary 1928a: 267.

##### Type locality.

Not explicitly stated but probably the same as for the species (“La Zousfana, à la hauteur de Figuig et à Beni Ounif” [Oued Zousfana, at the height of Figuig, and at Beni Ounif], Algeria).

##### Remarks.

Junior homonym of *Melanopsis buccinoidea major* Grateloup, 1838 (see Note 1).

#### Melanopsis subimpressa var. major

Taxon classificationAnimaliaSorbeoconchaMelanopsidae

Pallary, 1928 [invalid]

##### Original source.

Pallary 1928a: 263, pl. 6, fig. 1.

##### Type locality.

Not explicitly stated but probably the same as for the species (“Guefaït (Maroc oriental)”, Morocco).

##### Remarks.

Junior homonym of *Melanopsis buccinoidea major* Grateloup, 1838 (see Note 1).

#### Melanopsis tutulata var. major

Taxon classificationAnimaliaSorbeoconchaMelanopsidae

Pallary, 1928 [invalid]

##### Original source.

Pallary 1928a: 272.

##### Type locality.

Not stated but probably the same or partly as for the species (“Berguent; Aoûllout; Ras el Mâ de Fès; O. Chkef près Fès” [Aïn Beni Mathar, Aïn Aoullout, Ras El Ma, Oued Aïn Chkef in Fes], Morocco).

##### Remarks.

Junior homonym of *Melanopsis buccinoidea major* Grateloup, 1838 (see Note 1).

#### Melanopsis liocephala var. major

Taxon classificationAnimaliaSorbeoconchaMelanopsidae

Pallary, 1936 [invalid]

##### Original source.

Pallary 1936: 57.

##### Type locality.

Not indicated, but probably in Morocco.

##### Remarks.

Junior homonym of *Melanopsis buccinoidea major* Grateloup, 1838 (see Note 1).

#### Melanopsis saulcyi var. major

Taxon classificationAnimaliaSorbeoconchaMelanopsidae

Pallary, 1939 [invalid]

##### Original source.

Pallary 1939: 97, pl. 6, fig. 15.

##### Type locality.

“Lac d’Antioche, lac de Homs, Nahr el Kébir, Markieh, Yeni Chehir” [Lake Anuk (also as Amik), Yenişehir (Turkey), Lake Homs, Nahr el Kebir, river Marqīyah (Syria)].

##### Remarks.

Junior homonym of *Melanopsis buccinoidea major* Grateloup, 1838 (see Note 1).

#### † Melanopsis kleini mut. major

Taxon classificationAnimaliaSorbeoconchaMelanopsidae

Jodot, 1958 [unavailable]

##### Original source.

Jodot 1958: 65, pl. 11, figs 2–5.

##### Type horizon.

Middle Miocene.

##### Type locality.

“Route de Caravaca (Murcie)” [road to Caravaca, prov. Murcia], Spain.

##### Remarks.

Introduced as “mut. nov.” which is not ruled by the provisions of the Code.

#### † “ Melanopsis majoricensis

Taxon classificationAnimaliaSorbeoconchaMelanopsidae

” mentioned in Hermite (1879: 184, 328) [unavailable]

##### Horizon.

Early Eocene.

##### Locality.

“De Binisalem et de Selva” [from Binissalem and Selva, Mallorca], Spain.

##### Remarks.

Nomen nudum, listed by Hermite in a section called “Espèces nouvelles citées et non décrites” [= “new species identified and not described”], where he listed 18 new names that he intended to describe in the second volume of his “Études géologiques sur les îles Baléares”. That part, however, has never been published, probably because Hermite died in 1880.

#### † Melanopsis malladae

Taxon classificationAnimaliaSorbeoconchaMelanopsidae

Cossmann, 1906

##### Original source.

Cossmann 1906: 149, pl. C, figs 5–7.

##### Type horizon.

Ypresian, Eocene.

##### Type locality.

“Perauba” [section Peralba near Àger], Spain.

#### † Melanopsis mansiana

Taxon classificationAnimaliaSorbeoconchaMelanopsidae

Noulet, 1854

##### Original source.

Noulet 1854: 50.

##### Type horizon.

Ludian?–Sannoisian, Priabonian–early Rupelian, late Eocene–early Oligocene.

##### Type locality.

“Au Mas-Saintes-Puelles (Aude); [...] de la Massale, près de Castres; [...] à Labruguière; [...] à Saint-Genest-de-Contest, à Lautrec (Tarn)” [at Mas-Saintes-Puelles (Dép. Aude); from Massale near Castres; at Labruguière; at Saint-Genest-de-Contest, at Lautrec (Dép. Tarn)], France.

##### Remarks.

The name “*masensis*” as mentioned in Dollfus (1906: 292) is an incorrect subsequent spelling.

#### † Melanopsis maresi

Taxon classificationAnimaliaSorbeoconchaMelanopsidae

Bourguignat, 1862

##### Original source.

Bourguignat 1862: 106, pl. 6, figs 1–4.

##### Type horizon.

Pleistocene.

##### Type locality.

“Dans la daya de Habessa, ancien lac desséché, situé à plus de 200 lieues environ au sud d’Oran” [in the Daïa Habessa, an old, desiccated lake, located more than 800 km south of Oran], Algeria.

#### † Melanopsis margili

Taxon classificationAnimaliaSorbeoconchaMelanopsidae

Robles, 1975

##### Original source.

Robles 1975: 360, pl. 2, fig. 1.

##### Type horizon.

Mammal zone MN 13–15, late Miocene–Pliocene.

##### Type locality.

“Fuente del Viso (Albacete)” [near Villatoya], Spain.

##### Types.

Museo Nacional de Ciencias Naturales, Madrid, coll. no. M-435.

#### † Melanopsis marginata

Taxon classificationAnimaliaSorbeoconchaMelanopsidae

Braun in Walchner, 1851

##### Original source.

Braun in Walchner 1851: 1127.

##### Type horizon.

Early Miocene.

##### Type locality.

“Mainzer Becken” [Mainz Basin, no locality indicated], Germany.

##### Remarks.

Wenz (1929: 2726) considered the taxon as a junior synonym of *Melanopsis fritzei* Thomä, 1845.

#### Melanopsis mariei

Taxon classificationAnimaliaSorbeoconchaMelanopsidae

Crosse, 1869

##### Original source.

Crosse 1869a: 69 (Latin) [January].

##### Type locality.

“In loco ‘Baie du Sud’” [Baie Sud], New Caledonia.

##### Remarks.

Re-described in French by Crosse (1869b: 280, pl. 8, fig. 5) [October].

#### † Melanopsis haueri markusevecensis

Taxon classificationAnimaliaSorbeoconchaMelanopsidae

Wenz, 1930

##### Original source.

Wenz 1930: 65.

##### Type horizon.

Middle Pannonian, late Miocene.

##### Type locality.

“Markuševec” (Brusina 1902: pl. 6, figs 71–72), Croatia.

##### Remarks.

Replacement name for *Melanopsis austriaca croatica* Brusina, 1902, non Brusina, 1884 (see Note 1).

#### Melanopsis maroccana

Taxon classificationAnimaliaSorbeoconchaMelanopsidae

Morelet, 1853

##### Original source.

Morelet 1853: 297.

##### Type locality.

“Prov. Oranensem” [province of Oran; no locality indicated], Morocco.

##### Remarks.

The name was first mentioned by Chemnitz (1795) as “*Buccina Maroccana*”. However, according to Opinion 184 (ICZN 1944), species names introduced in volumes 1–11 of Martini and Chemnitz’ “Neues Systematischer Conchylien Cabinet” (1769–1795) have no status in nomenclature and the name is therefore not available from this work. The name was again listed by Férussac (1823) in synonymy of two different species, i.e., *Melanopsis dufourii* Férussac, 1822 (referring to Chemnitz 1795: pl. 210, figs 2078–2081) and *Melanopsis costellata* (referring to Chemnitz 1795: pl. 210, figs 2082–2083). A mention in synonymy, however, does not make the name available (see Note 2).

The first to adopt *Melanopsis maroccana* as a valid name was Morelet (1853). (Note that in the same year Bourguignat mentioned the name in his “Catalogue raisonné des mollusques terrestres et fluviatiles [...]”, which, according to the preface was published not before 1 December 1853, while Morelet’s paper was issued on 1 August 1853.) Morelet (as well as Bourguignat) referred to Chemnitz’ work – but only to figs 2080–2081 – and listed *Melanopsis dufourei* [sic] as a synonym. Morelet did not provide a description or illustrations on his own; the brief Latin description he gave below the synonymy list refers to the unnamed variety β. The reference to the illustrations in Chemnitz nonetheless suffices as indication of a new taxon; the correct name is therefore *Melanopsis maroccana* Morelet, 1853. Although Morelet (1853) considered the older *Melanopsis dufourii* as a junior synonym of *Melanopsis maroccana*, the two names are no objective synonyms because Morelet referred to more specimens than just the holotype of *Melanopsis dufourii* (fig. 16). Note, moreover, that Morelet’s synonymization is likely based on a wrong concept of *Melanopsis dufourii*, which is a fossil species described from the Miocene of France.

The name “*marocana*” as mentioned in Pallary (1920c: 145) is an incorrect subsequent spelling.

#### Melanopsis marteli

Taxon classificationAnimaliaSorbeoconchaMelanopsidae

Pallary, 1920

##### Original source.

Pallary 1920a: 32.

##### Type locality.

“Près de Taforalt; Oued Cherâa à Berkane” [near Taforhalt; Oued Cherraa at Berkane], Morocco.

#### † Melanopsis marticensis

Taxon classificationAnimaliaSorbeoconchaMelanopsidae

Matheron, 1842

##### Original source.

Matheron 1842: 292, pl. 37, fig. 7.

##### Type horizon.

Early Campanian, Cretaceous.

##### Type locality.

“Les Martigues”, France.

#### † Melanopsis martiniana

Taxon classificationAnimaliaSorbeoconchaMelanopsidae

Férussac, 1823

##### Original source.

Férussac 1819–1851: Mélanopsides fossiles, pl. 2 (1823), figs 11–13 or Férussac 1823: 155, pl. 8, figs 11–13 (precedence not established; see introduction for discussion).

##### Type horizon.

Pannonian, late Miocene.

##### Type locality.

“Dans les environs de Bisentz et de Scharditz, en Moravie, dans la vallée de la Marsch, affluent du Danube” [in the surrondings of Bzenec and Šardice in Moravia, in the valley of the March river, a tributary of the Danube], Czech Republic.

##### Remarks.

Junior objective synonym of *Melanopsis fossilis* (for details see there). The name “*martinii*” as mentioned by numerous authors (e.g., Chenu 1859: 297) is an incorrect subsequent spelling.

#### † Melanopsis matheroni

Taxon classificationAnimaliaSorbeoconchaMelanopsidae

Mayer, 1871

##### Original source.

Mayer 1871: 201.

##### Type horizon.

Messinian, late Miocene.

##### Type locality.

“Narzole?” (p. 189; apparently the locality was uncertain), Italy.

##### Remarks.

Wenz (1929: 2783) considered this taxon as a junior synonym of *Melanopsis narzolina* d’Archiac in Viquesnel, 1846.

#### Melanopsis letourneuxi var. mattarica

Taxon classificationAnimaliaSorbeoconchaMelanopsidae

Pallary, 1911

##### Original source.

Pallary 1911: 132, [unnumbered plate], figs 13–14.

##### Type locality.

“Berguent, le Ras al Aïqun des Beni-Mattar” [Ras el Aïn at Aïn Beni Mathar], Morocco.

##### Remarks.

Introduced for *Melanopsis letourneuxi* sensu Pallary, 1899 (pl. 8, fig. 6), non Bourguignat, 1884.

#### Melanopsis mauretanica

Taxon classificationAnimaliaSorbeoconchaMelanopsidae

Pallary, 1922 [invalid]

##### Original source.

Pallary 1922: 207.

##### Remarks.

Unjustified emendation and therefore junior objective synonym of *Melanopsis mauritanica* Bourguignat, 1884.

#### Melanopsis mauritanica

Taxon classificationAnimaliaSorbeoconchaMelanopsidae

Bourguignat, 1884

##### Original source.

Bourguignat 1884: 102.

##### Type locality.

“Çà et là de Maroc” [here and there in Morocco; no locality indicated].

#### † Melanopsis mausseneti

Taxon classificationAnimaliaSorbeoconchaMelanopsidae

Cossmann, 1888

##### Original source.

Cossmann 1888: 284, pl. 11, fig. 10.

##### Type horizon.

Sparnacian, early Ypresian, Eocene.

##### Type locality.

“Mont Bernon” [near Épernay], France.

#### Melanopsis saulcyi var. maxima

Taxon classificationAnimaliaSorbeoconchaMelanopsidae

Dautzenberg, 1894

##### Original source.

Dautzenberg 1894: 345.

##### Type locality.

“Bir Jaloûd” [not found, in the Middle East].

#### Melanopsis praemorsa maximalis

Taxon classificationAnimaliaSorbeoconchaMelanopsidae

Schütt & Bilgin, 1974

##### Original source.

Schütt and Bilgin 1974: 63, fig. 6.

##### Type locality.

“Sakarya başi, main spring of Sakarya river near village Çifteler, 60 km SE Eskişehir, 160 km WSW of Ankara”, Turkey.

##### Types.

Senckenberg Forschungsinstitut und Naturmuseum Frankfurt, coll. no. SMF 232011.

#### Melanopsis maroccana var. media

Taxon classificationAnimaliaSorbeoconchaMelanopsidae

Blanckenhorn, 1897

##### Original source.

Blanckenhorn 1897: 124, pl. 10, fig. 4.

##### Type locality.

“Dans les eaux le l’ancien Léonthes” (Mousson 1854: 51) [in the Litani river], Lebanon.

##### Remarks.

Replacement name for *Melanopsis brevis* Parreyss in Mousson, 1854, non Sowerby, 1826 (see also Pallary 1916). Blanckenhorn (1897) denoted the authority as “Bourg.”, but from the discussion it is clear that Blanckenhorn must be the author.

#### Melanopsis marocana [sic] var. media

Taxon classificationAnimaliaSorbeoconchaMelanopsidae

Pallary, 1920 [invalid]

##### Original source.

Pallary 1920c: 146, pl. 4, fig. 14.

##### Type locality.

“Taza. Fes (dar el Maghzen)”, Morocco.

##### Remarks.

Junior homonym of *Melanopsis maroccana media* Blanckenhorn, 1897 (see Note 1).

#### Melanopsis sevillensis var. media

Taxon classificationAnimaliaSorbeoconchaMelanopsidae

Pallary, 1924 [invalid]

##### Original source.

Pallary 1924: 252.

##### Type locality.

“De la petite rivière de Guadaira qui se jette dans le Guadalquivir” [in the little river Guadaira which flows into the Guadalquivir], Spain.

##### Remarks.

Junior homonym of *Melanopsis maroccana media* Blanckenhorn, 1897 (see Note 1).

#### † Melanopsis medinae

Taxon classificationAnimaliaSorbeoconchaMelanopsidae

Neubauer, Mandic, Harzhauser & Hrvatović, 2013

##### Original source.

Neubauer et al. 2013: 135, figs 5A–D.

##### Type horizon.

Langhian, middle Miocene.

##### Type locality.

“Žepj” (Neumayr 1880c: 480) [Džepi], Bosnia and Herzegovina.

##### Remarks.

Replacement name for the junior secondary homonym *Melanoptychia bittneri* Neumayr, 1880, non *Melanopsis bittneri* Fuchs, 1877. Neubauer et al. (2016c: 275–276) considered the species as a junior synonym of *Melanopsis carusi* (Brusina, 1902).

#### † Melanopsis (Canthidomus) bouei var. megacantha

Taxon classificationAnimaliaSorbeoconchaMelanopsidae

Handmann, 1887

##### Original source.

Handmann 1887: 36, pl. 8, figs 13–15.

##### Type horizon.

Pannonian, zone B–D, late Miocene.

##### Type locality.

“Leobersdorf”, Austria.

##### Remarks.

Originally the gender was indicated as masculine (“*megacanthus*”), but *Melanopsis* is feminine, which is why the name must be corrected to “*megacantha*”.

#### † Melanopsis themaki var. megalostoma

Taxon classificationAnimaliaSorbeoconchaMelanopsidae

Brusina, 1903

##### Original source.

Brusina 1903: 111.

##### Type horizon.

Late Pleistocene–early Holocene.

##### Type locality.

“Bischofsbad” [Püspökfürdő, Băile 1 Mai, Lake Pețea], Romania.

##### Remarks.

Neubauer et al. (2014d: 125) considered this taxon as a junior synonym of *Microcolpia parreyssii sikorai* (Brusina, 1903).

#### † “ Melanopsis sikorai var. megaotyla

Taxon classificationAnimaliaSorbeoconchaMelanopsidae

” mentioned in Brusina (1903: 112) [unavailable]

##### Horizon.

Late Pleistocene–early Holocene.

##### Locality.

“Bischofsbad” [Püspökfürdő, Băile 1 Mai, Lake Pețea], Romania.

##### Remarks.

Nomen nudum. If available, it would be a junior objective synonym of *Melanopsis sikorai*: Brusina (1903) indicated it as the typical form of the species. Neubauer et al. (2014d: 125) considered this taxon as a junior synonym of *Microcolpia parreyssii sikorai* (Brusina, 1903).

#### † Melanopsis hazayi var. megatyla

Taxon classificationAnimaliaSorbeoconchaMelanopsidae

Brusina, 1903

##### Original source.

Brusina 1903: 112.

##### Type horizon.

Late Pleistocene–early Holocene.

##### Type locality.

“Bischofsbad” [Püspökfürdő, Băile 1 Mai, Lake Pețea], Romania.

##### Remarks.

Neubauer et al. (2014d: 125) considered this taxon as a junior synonym of *Microcolpia parreyssii sikorai* (Brusina, 1903).

#### Melanopsis meiostoma

Taxon classificationAnimaliaSorbeoconchaMelanopsidae

Heller & Sivan, 2000

##### Original source.

Heller and Sivan 2000: 1, fig. 1.

##### Type locality.

“En Haruv [...], a small spring on the Golan Heights that pours into a small cement pool” [near Kefar Haruv], Syria.

##### Types.

National mollusc collection of the Hebrew University, Jerusalem, coll. no. HUJ 7966.

#### Melanopsis crenocarinata var. melanostoma

Taxon classificationAnimaliaSorbeoconchaMelanopsidae

Moricand, 1841

##### Original source.

Moricand 1841: 62.

##### Type locality.

“Rio de Pedra Branca, procince de Bahia” [Pedra Branca river, province Bahia], Brazil.

##### Remarks.

Although not explicitly stated, this variety was apparently considered to belong to the new genus *Verena* by Adams and Adams (1854) (Thiaridae), of which *Melanopsis crenocarinata* is the type species (see Nuttall 1990: 253).

#### Melanopsis mellalensis

Taxon classificationAnimaliaSorbeoconchaMelanopsidae

Pallary, 1928

##### Original source.

Pallary 1928b: 18, pl. 2, figs 18–23.

##### Type locality.

“L’Oued Taguenout à Beni Mellal” [Beni Mellal, in the Oued Taguenout (?)], Morocco.

#### Melanopsis (Mesopotamia) mesopotamica

Taxon classificationAnimaliaSorbeoconchaMelanopsidae

Pallary, 1939

##### Original source.

Pallary 1939: 99, pl. 5, figs 1–6.

##### Type locality.

“‘Ain Arouss (la source de la fiancée), près de Tell Abiad, d’où naît le Nahr Bâhlik, qui se jette dans Euphrate, un peu au-dessous de Rakka, la métropole de Haroun el Rachid” [‘Ayn al ‘Arūs (source of the bride), near Tall Abyaḑ, from which arises the Nahr al Balīkh, which flows into the Euphrates, a little below Ar Raqqah, the metropolis of Harun al-Rashid], Syria.

##### Remarks.

Heller et al. (2005: 254) considered the species as a junior synonym of *Melanopsis infracincta* Martens, 1874.

#### † Melanopsis metochiana

Taxon classificationAnimaliaSorbeoconchaMelanopsidae

Pavlović, 1932

##### Original source.

Pavlović 1932: 239, 247, pl. 1, figs 3–5.

##### Type horizon.

Pontian (Dacian Basin), late Miocene–Pliocene.

##### Type locality.

“Села Дрсника” [village Drsnik], Kosovo.

##### Types.

The illustrated syntype is stored in the Natural History Museum, Belgrade, coll. no. 1196 (Milošević 1962: 24).

#### Melanopsis cerithiopsis var. mezeribensis

Taxon classificationAnimaliaSorbeoconchaMelanopsidae

Pallary, 1939

##### Original source.

Pallary 1939: 95, pl. 6, figs 37–39.

##### Type locality.

“Mézérib” [Muzayrib], Syria.

#### † Pseudofagotia michailowskii

Taxon classificationAnimaliaSorbeoconchaMelanopsidae

Anistratenko, 1993

##### Original source.

Anistratenko 1993: 73, textfig. 2.

##### Type horizon.

Duab Beds, middle to late Kimmerian, Pliocene.

##### Type locality.

“Окр. с. Мокви, Очамчирский р-н” [near the village Mok’vi, Ochamchirskiy rayon], Georgia.

##### Types.

Schmalhausen Institute of Zoology of National Academy of Sciences of Ukraine, Kiev; no number indicated.

#### † Melanopsis michelottiana

Taxon classificationAnimaliaSorbeoconchaMelanopsidae

Pallary, 1916

##### Original source.

Pallary 1916: 81.

##### Type horizon.

Late Miocene.

##### Type locality.

“À St. Agata près de Tortone” (Michelotti 1847: 191; the other localities Michelotti apparently adopted from other works) [at Sant’Agata Fossili near Tortona], Italy.

##### Remarks.

Introduced for *Melanopsis carinata* sensu Michelotti, 1847, non Sowerby, 1826. Wenz (1929: 2671) considered the taxon as a junior synonym of *Melanopsis bonellii* Manzoni, 1870.

#### Melanopsis microcolpia

Taxon classificationAnimaliaSorbeoconchaMelanopsidae

Bourguignat, 1884

##### Original source.

Bourguignat 1884: 81.

##### Type locality.

“Près de Jéricho, dans la fontaine de Jérémie (Palestine)” [near Jericho, in the spring of Jeremiah (?)], Palestine.

#### Melanopsis microstoma

Taxon classificationAnimaliaSorbeoconchaMelanopsidae

Bourguignat, 1884

##### Original source.

Bourguignat 1884: 159.

##### Type locality.

“Ruisseau de la source de la Moulouiah, près de Lalla-Maghnia sur la frontière marocaine (prov. d’Oran)” [in the source of the river Moulouya, near Maghnia at the border to Morocco], Algeria.

#### † “ Melanopsis microstoma

Taxon classificationAnimaliaSorbeoconchaMelanopsidae

” mentioned in Newton (1891: 203) [unavailable]

##### Horizon.

Woolwich Beds, early Eocene.

##### Locality.

Woolwich, United Kingdom.

##### Remarks.

Nomen nudum. If available, it would be a junior homonym of *Melanopsis microstoma* Bourguignat, 1884.

#### Melanopsis mingrelica

Taxon classificationAnimaliaSorbeoconchaMelanopsidae

Mousson, 1863

##### Original source.

Mousson 1863: 411.

##### Type locality.

“De l’intérieur de la Mingrélie [...], puis de Réduktaleh” [from the interior of Samegrelo; from Q’ulevi (also read as Kulevi, former Redut-Kale)], Georgia.

##### Remarks.

Mousson attributed the authority to Bayer, but there is no evidence that the description really derived from that author. Bayer only seems to have collected a part of the material.

#### † Melanopsis minima

Taxon classificationAnimaliaSorbeoconchaMelanopsidae

Blanckenhorn, 1897

##### Original source.

Blanckenhorn 1897: 114, pl. 9, figs 2–5.

##### Type horizon.

Plio-Pleistocene.

##### Type locality.

“In der tiefsten Thonbank des linken Orontesufers bei Dschisr esch-Schurr” [in the lowest clay bank at the left riverside of the Orontes near Jisr Ash-Shughur], Syria.

##### Remarks.

Introduced as “n. mut.” but clearly as a binomen and hence not infrasubspecific in the sense of ICZN Art. 45.6.

#### † Melanopsis buccinoidea var. minor

Taxon classificationAnimaliaSorbeoconchaMelanopsidae

Grateloup, 1838

##### Original source.

Grateloup 1838: 146.

##### Type horizon.

Burdigalian, early Miocene.

##### Type locality.

“Dax. [...] Mandillot; à Saint-Paul”, France.

#### Melanopsis acicularis var. minor

Taxon classificationAnimaliaSorbeoconchaMelanopsidae

Rossmässler, 1839 [invalid]

##### Original source.

Rossmässler 1838–1844: 41, pl. 50, fig. 672.

##### Type locality.

“In schwach schwefeligen Quellen [...] bei Vöslau unweit Baden” [in weakly sulfurous springs stones and sands at Vöslau near Baden], Austria.

##### Remarks.

Junior objective synonym of *Melanopsis daudebartii* [Prevost], 1821, which Rossmässler listed in synonymy. Moreover, the name is a junior homonym of *Melanopsis buccinoidea minor* Grateloup, 1838 (see Note 1).

#### Melanopsis costata var. minor

Taxon classificationAnimaliaSorbeoconchaMelanopsidae

Martens, 1874 [invalid]

##### Original source.

Martens 1874: 33, p. 5, fig. 40.

##### Type locality.

“Quellen des Chabur bei Ras-el-Ain” [source of Chabur river near Ra’s al ‘Ayn], Syria.

##### Remarks.

Junior homonym of *Melanopsis buccinoidea minor* Grateloup, 1838 (see Note 1).

#### † Melanopsis bonellii var. minor

Taxon classificationAnimaliaSorbeoconchaMelanopsidae

Locard, 1878 [invalid]

##### Original source.

Locard 1878: 56.

##### Type horizon.

Tortonian, late Miocene.

##### Type locality.

“À Tersannes près de Hauterives (Drôme)” [at Tersanne near Hauterives], France.

##### Remarks.

Junior homonym of *Melanopsis buccinoidea minor* Grateloup, 1838 (see Note 1). Wenz (1929: 2669) considered the taxon as a junior synonym of *Melanopsis bonellii* Manzoni, 1870.

#### Melanopsis belonidaea var. minor

Taxon classificationAnimaliaSorbeoconchaMelanopsidae

Bourguignat, 1884 [invalid]

##### Original source.

Bourguignat 1884: 111.

##### Type locality.

“Ruisseau d’eau chaude à Ouargla (prov. de Constantine) et eaux thermales du Djérid, au nord du chott Tiraoun (sud de la Tunisie)” [in warm waters at Ouargla (Algeria) and thermal water of Djérid, north of chott Tiraoun (Tunisia)].

##### Remarks.

Junior homonym of *Melanopsis buccinoidea minor* Grateloup, 1838 (see Note 1).

#### Melanopsis prophetarum var. minor

Taxon classificationAnimaliaSorbeoconchaMelanopsidae

Bourguignat, 1884 [invalid]

##### Original source.

Bourguignat 1884: 82.

##### Type locality.

“Lac d’Antioche” [Lake Anuk (also as Amik)], Turkey.

##### Remarks.

Junior homonym of *Melanopsis buccinoidea minor* Grateloup, 1838 (see Note 1). Blanckenhorn (1897) indicated himself as author of the taxon, but he clearly cited the record by Bourguignat (1884) in the synonymy list. Wenz (1929) introduced *Melanopsis blanckenhorni* as replacement name.

#### Melanopsis salomonis var. minor

Taxon classificationAnimaliaSorbeoconchaMelanopsidae

Bourguignat, 1884 [invalid]

##### Original source.

Bourguignat 1884: 96.

##### Type locality.

“Environs d’Alep, à Sadjour-Sou, à quatre kilom. en aval d’Aïn-Taïb; ruisseaux à Doumar, sur l’oued Baradah, près Aïn-Fidji, et à Banias, en Syrie” [Surroundings of Aleppo (Syria), at Sadjour-Sou, 4 km downstream of Gaziantep (Turkey); streams at Dummar, at the river Barada, near Aïn al-Fiji and at Bāniyās (Syria)].

##### Remarks.

Junior homonym of *Melanopsis buccinoidea minor* Grateloup, 1838 (see Note 1).

#### Microcolpia letourneuxi var. minor

Taxon classificationAnimaliaSorbeoconchaMelanopsidae

Bourguignat, 1884

##### Original source.

Bourguignat 1884: 65.

##### Type locality.

Not indicated, but probably as for the species (“Dans la Save, au-dessous d’Agram” [in the Sava river below Zagreb], Croatia).

##### Remarks.

Starobogatov et al. (1992: 65) considered the taxon as a junior synonym of *Microcolpia cornea* (Pfeiffer, 1828).

Melania (Amphimelania) holandri f. minor Westerlund, 1886 [invalid]

##### Original source.

Westerlund 1886: 104.

##### Type locality.

Not indicated.

##### Remarks.

Junior homonym of *Melania tuberculata minor* Brot, 1877 from Sri Lanka (see Note 1).

#### Melanopsis gracilenta var. minor

Taxon classificationAnimaliaSorbeoconchaMelanopsidae

Pallary, 1911 [invalid]

##### Original source.

Pallary 1911: 130, [unnumbered plate], fig. 20.

##### Type locality.

“Tout près d’Oudjda, à 4 kilom. S.-E., sourdent les belles sources de Sidi-Yahia qui alimentent une véritable oasis, puis la ville d’Oudjda, et vont finalement se déverser dans l’oued Isly” [near Oujda, 4 km southeast, at the sources of Sidi Yahya that feed an oasis and the city of Oujda, and ultimately will flow into the Oued Isly], Morocco.

##### Remarks.

Junior homonym of *Melanopsis buccinoidea minor* Grateloup, 1838 (see Note 1).

#### Melanopsis letourneuxi var. minor

Taxon classificationAnimaliaSorbeoconchaMelanopsidae

Pallary, 1911 [invalid]

##### Original source.

Pallary 1911: 132, [unnumbered plate], fig. 16.

##### Type locality.

“Berguent, le Ras al Aïqun des Beni-Mattar” [Ras el Aïn at Aïn Beni Mathar], Morocco.

##### Remarks.

Junior homonym of *Melanopsis buccinoidea minor* Grateloup, 1838 (see Note 1).

#### Melanopsis nobilis var. minor

Taxon classificationAnimaliaSorbeoconchaMelanopsidae

Pallary, 1912 [invalid]

##### Original source.

Pallary 1912: 21, fig. 42.

##### Type locality.

“Des bords du Chott Djerid, à Tozeur” [banks of the Chott el Djérid at Tozeur], Tunisia.

##### Remarks.

Junior homonym of *Melanopsis buccinoidea minor* Grateloup, 1838 (see Note 1).

#### Melanopsis attenuata var. minor

Taxon classificationAnimaliaSorbeoconchaMelanopsidae

Pallary, 1920 [invalid]

##### Original source.

Pallary 1920a: 29.

##### Type locality.

“Tétouan”, Morocco.

##### Remarks.

Junior homonym of *Melanopsis buccinoidea minor* Grateloup, 1838 (see Note 1).

#### Melanopsis douttei var. decorata minor

Taxon classificationAnimaliaSorbeoconchaMelanopsidae

Pallary, 1920 [unavailable]

##### Original source.

Pallary 1920c: 150.

##### Type locality.

Not indicated, but probably as for the variety (“La Makina”, Morocco).

##### Remarks.

Introduced as infrasubspecific taxon, which is not ruled by the provisions of the Code.

#### Melanopsis fasesnsis var. minor

Taxon classificationAnimaliaSorbeoconchaMelanopsidae

Pallary, 1920 [invalid]

##### Original source.

Pallary 1920c: 148.

##### Type locality.

“Dar Batha” [near Fes], Morocco.

##### Remarks.

Junior homonym of *Melanopsis buccinoidea minor* Grateloup, 1838 (see Note 1).

#### Melanopsis pseudoferussaci var. minor

Taxon classificationAnimaliaSorbeoconchaMelanopsidae

Pallary, 1920 [invalid]

##### Original source.

Pallary 1920c: 147, pl. 4, figs 15–16.

##### Type locality.

“Fès: source près de la ferme proche du pont neuf, à dar Mahrès; Sidi Harazen (source chaude); Moulai Idriss du Zehroun, source sulfureuse chaude; El Menzel; Tazouta” [Fes: source near the farm at the new bridge, at Dar Mahres; Sidi Harazem (hot spring); Moulay Driss Zerhoun, hot sulfur springs; El Menzel; Tazouta], Morocco.

##### Remarks.

Junior homonym of *Melanopsis buccinoidea minor* Grateloup, 1838 (see Note 1).

#### Melanopsis dufourii var. minor

Taxon classificationAnimaliaSorbeoconchaMelanopsidae

Pallary, 1924 [invalid]

##### Original source.

Pallary 1924: 249.

##### Type locality.

“Fortuna et Caravala [Caravaca?] (Murcia). Lorca, Valencia et Alicante”, Spain.

##### Remarks.

Junior homonym of *Melanopsis buccinoidea minor* Grateloup, 1838 (see Note 1).

#### Melanopsis lorcana f. minor

Taxon classificationAnimaliaSorbeoconchaMelanopsidae

Pallary, 1924 [invalid]

##### Original source.

Pallary 1924: 247, pl. 15, fig. 8.

##### Type locality.

Spain [no locality indicated].

##### Remarks.

Junior homonym of *Melanopsis buccinoidea minor* Grateloup, 1838 (see Note 1).

#### Melanopsis hadiensis var. minor

Taxon classificationAnimaliaSorbeoconchaMelanopsidae

Pallary, 1928 [invalid]

##### Original source.

Pallary 1928a: 269.

##### Type locality.

“Bel Hadi-Kenadsa (Sud Oranais)” [Bel Hadi, near Kenadsa], Algeria.

##### Remarks.

Junior homonym of *Melanopsis buccinoidea minor* Grateloup, 1838 (see Note 1).

#### Melanopsis macilenta var. minor

Taxon classificationAnimaliaSorbeoconchaMelanopsidae

Pallary, 1928 [invalid]

##### Original source.

Pallary 1928b: 18, pl. 2, fig. 17.

##### Type locality.

“O. Taguenout” [Oued Taguenout (?), said to be near Beni Mellal], Morocco.

##### Remarks.

Junior homonym of *Melanopsis buccinoidea minor* Grateloup, 1838 (see Note 1).

#### Melanopsis mellalensis var. minor

Taxon classificationAnimaliaSorbeoconchaMelanopsidae

Pallary, 1928 [invalid]

##### Original source.

Pallary 1928b: 19, pl. 2, fig. 21.

##### Type locality.

“Dans l’oued Daï” [not found], Morocco.

##### Remarks.

Junior homonym of *Melanopsis buccinoidea minor* Grateloup, 1838 (see Note 1).

#### Melanopsis seurati var. minor

Taxon classificationAnimaliaSorbeoconchaMelanopsidae

Pallary, 1928 [invalid]

##### Original source.

Pallary 1928a: 267.

##### Type locality.

Not explicitly stated but probably the same as for the species (“La Zousfana, à la hauteur de Figuig et à Beni Ounif” [Oued Zousfana, at the height of Figuig, and at Beni Ounif], Algeria).

##### Remarks.

Junior homonym of *Melanopsis buccinoidea minor* Grateloup, 1838 (see Note 1).

#### Melanopsis turgida var. minor

Taxon classificationAnimaliaSorbeoconchaMelanopsidae

Pallary, 1928 [invalid]

##### Original source.

Pallary 1928a: 259, pl. 5, fig. 12.

##### Type locality.

“Moulaï Taïeb” [Moulay Taïeb], Morocco.

##### Remarks.

Name appears only in the plate captions. Junior homonym of *Melanopsis buccinoidea minor* Grateloup, 1838 (see Note 1).

#### Melanopsis tutulata var. minor

Taxon classificationAnimaliaSorbeoconchaMelanopsidae

Pallary, 1928 [invalid]

##### Original source.

Pallary 1928a: 272.

##### Type locality.

Not stated but probably the same or partly as for the species (“Berguent; Aoûllout; Ras el Mâ de Fès; O. Chkef près Fès” [Aïn Beni Mathar, Aïn Aoullout, Ras El Ma, Oued Aïn Chkef in Fes], Morocco).

##### Remarks.

Junior homonym of *Melanopsis buccinoidea minor* Grateloup, 1838 (see Note 1).

#### Melanopsis vondeli var. minor

Taxon classificationAnimaliaSorbeoconchaMelanopsidae

Pallary, 1928 [invalid]

##### Original source.

Pallary 1928b: 16.

##### Type locality.

“Dans l’oued Daï” [not found], Morocco.

##### Remarks.

Junior homonym of *Melanopsis buccinoidea minor* Grateloup, 1838 (see Note 1).

#### Melanopsis wagneri var. minor

Taxon classificationAnimaliaSorbeoconchaMelanopsidae

Pallary, 1930 [invalid]

##### Original source.

Pallary 1930: 290.

##### Type locality.

“Dans l’eau tiède du bassin de Diane, à Smyrne” [in warm water basin of Halkapınar at Izmir], Turkey.

##### Remarks.

Junior homonym of *Melanopsis buccinoidea minor* Grateloup, 1838 (see Note 1).

#### Melanopsis (Mesopotamia) khabourensis var. minor

Taxon classificationAnimaliaSorbeoconchaMelanopsidae

Pallary, 1939 [invalid]

##### Original source.

Pallary 1939: 103, pl. 5, fig. 21.

##### Type locality.

“Ras el ‘Ain du Khabour” [Chabur river near Ra’s al ‘Ayn], Syria.

##### Remarks.

Junior homonym of *Melanopsis buccinoidea minor* Grateloup, 1838 (see Note 1).

#### Melanopsis (Mesopotamia) mesopotamica var. minor

Taxon classificationAnimaliaSorbeoconchaMelanopsidae

Pallary, 1939 [invalid]

##### Original source.

Pallary 1939: 100, pl. 4, fig. 6.

##### Type locality.

“Dans le canal de la Butte de tir, à 7 km Sud de Baghdad” [in a trench of a firing hill (?), 7 km south of Baghdad], Iraq.

##### Remarks.

Ranked as a variety of *Melanopsis nodosa* Férussac in the plate captions. Junior homonym of *Melanopsis buccinoidea minor* Grateloup, 1838 (see Note 1).

#### Melanopsis pachya var. minor

Taxon classificationAnimaliaSorbeoconchaMelanopsidae

Pallary, 1939 [invalid]

##### Original source.

Pallary 1939: 87.

##### Type locality.

“Dans les sources de Mézérib, au N. O. de Derâa (Syrie méridionale)” [in the sources of the Muzayrīb, northwest of Dar’ā], Syria.

##### Remarks.

Junior homonym of *Melanopsis buccinoidea minor* Grateloup, 1838 (see Note 1).

#### † Melanopsis (Canthidomus) oroposi minoriformis

Taxon classificationAnimaliaSorbeoconchaMelanopsidae

Papp, 1979

##### Original source.

Papp 1979: 672, pl. 3, figs 11–15.

##### Type horizon.

Mammal zone MN 10, late Miocene.

##### Type locality.

“Milessi” [Milesi], Greece.

##### Types.

Institute of Paleontology, University of Vienna; no number indicated.

#### Melanopsis monteli var. minorstricta

Taxon classificationAnimaliaSorbeoconchaMelanopsidae

Pallary, 1936

##### Original source.

Pallary 1936: 61.

##### Type locality.

Not explicitly stated but probably the same as for the species (“L’Oued Sous, au pont des Aït Melloul, sur la route d’Agadir à Tiznit, à 14 kil. S. O. d’Agadir” [in the Oued Sous, at the bridge of Ait Melloul, at the road from Agadir to Tiznit, 14 km southwest of Agadir], Morocco).

##### Remarks.

Originally spelt as “*minor-stricta*”.

#### † Melanopsis minotauri

Taxon classificationAnimaliaSorbeoconchaMelanopsidae

Willmann, 1980

##### Original source.

Willmann 1980: 285.

##### Type horizon.

Viannos Formation, early Tortonian, late Miocene.

##### Type locality.

“Tongrube 1 km westlich von Limin Chersonisou/Kreta” [claypit 1 km west of Chersonisos, near Agia Anna chapel], Greece.

#### † Melanopsis minuta

Taxon classificationAnimaliaSorbeoconchaMelanopsidae

Pallary, 1916

##### Original source.

Pallary 1916: 77.

##### Type horizon.

Mammal zone MN 10–12, late Miocene.

##### Type locality.

“De Cuiseaux”, France.

##### Remarks.

Used as valid name by Wenz (1929: 2780), but obviously not intended as species-group name by Férussac (1823: 150, pl. 7, fig. 4). The name became nevertheless available from Pallary (1916) who treated it as valid species-group name and associated it with an illustration in Férussac (1823: pl. 7, fig. 4). See introduction for a detailed discussion about the names used by Férussac (1823).

#### Melanopsis minutula

Taxon classificationAnimaliaSorbeoconchaMelanopsidae

Bourguignat, 1884

##### Original source.

Bourguignat 1884: 92.

##### Type locality.

“Fontaine froide du Hammam à Brousse (Anatolie); Nahr-Antalies dans le Liban (Syrie); puits artésien de Tamerna-Kedima, dans le Ziban (Algérie)” [cold springs in a Hamam at Bursa (Turkey); Nahr-Antalies (?) in the Lebanon; artesian well of Tamerna Kedima (Algeria)].

#### † Melanopsis lyrata var. misera

Taxon classificationAnimaliaSorbeoconchaMelanopsidae

Brusina, 1874

##### Original source.

Brusina 1874: 45.

##### Type horizon.

Langhian, middle Miocene.

##### Type locality.

“Vrba; Sinj (Stuparuša); Turiake [Turjaci]”, Croatia.

##### Types.

Milan et al. (1974: 94) indicated a holotype, but it is uncertain whether the specimen is part of the original type series and whether it was the only one Brusina had at hand (holotype by monotypy, Art. 73.1.2). The specimen is stored in the Croatian Natural History Museum, Zagreb, coll. no. 2972-618.

##### Remarks.

Neubauer et al. (2011: 207) considered this taxon as a junior synonym of *Melanopsis lyrata* Neumayr, 1869.

#### † Melanopsis (Lyrcaea) [sic] mitraeformis

Taxon classificationAnimaliaSorbeoconchaMelanopsidae

Andrusov, 1909

##### Original source.

Andrusov 1909: 87, 160, pl. 4, figs 34–35.

##### Type horizon.

Pontian (sensu stricto), late Miocene.

##### Type locality.

“Babadjan” [Babadzhan], Azerbaijan.

#### † Melanopsis (Melanosteira) mitzopoulosi

Taxon classificationAnimaliaSorbeoconchaMelanopsidae

Papp, 1955

##### Original source.

Papp 1955: 126, pl. 20, figs 15–19.

##### Type horizon.

Late Pliocene to early Pleistocene.

##### Type locality.

“Nord-Nordwestlich von der Solfatare bei Susaki” [north-northwest of Solfatara Sousáki], Greece.

##### Types.

Museum of Palaeontology and Geology of the University of Athens; no number indicated.

##### Remarks.

First mentioned as nomen nudum in Papp and Thenius (1952: 4). Junior synonym of *Amphimelania gayi* [sic] after Bandel (2000: 140).

#### † Melanopsis (Canthidomus) mitzopoulosi

Taxon classificationAnimaliaSorbeoconchaMelanopsidae

Kühn, 1963 [invalid]

##### Original source.

Kühn 1963: 386.

##### Type horizon.

Mammal zone MN 10, late Miocene.

##### Type locality.

“Peristeri”, Greece.

##### Types.

Museum of Palaeontology and Geology of the University of Athens, coll. no. 1963/83.

##### Remarks.

The status of this species is unclear. First of all, Kühn did not at all refer to the senior homonym Melanopsis (Melanosteira) mitzopoulosi Papp, 1955 but clearly described the species as new. Neither did Papp (1979: 668), who must have been aware of the issue and who ranked it as a subspecies of *Melanopsis longa* Deshayes in Férussac, 1839. Moreover, Papp stated that Kühn’s species was based on the material collected by Papp (1947) and considered the type locality mentioned by Kühn a mistake. Kühn (1963), however, did not refer to Papp’s material at all.

#### Melanopsis nodosa var. moderata

Taxon classificationAnimaliaSorbeoconchaMelanopsidae

Mousson, 1874

##### Original source.

Mousson 1874: 48.

##### Type locality.

Not explicitly stated but probably the same as for the species (“Au cours de l’Euphrate et du Tigre” [from the Euphrates and Tigris rivers], Iraq).

##### Remarks.

Brot (1874–1879: 432) considered the taxon as a junior synonym of *Melanopsis nodosa* Férussac, 1822, which is actually a fossil species described from the Miocene of France.

#### † “ Melanopsis moderata

Taxon classificationAnimaliaSorbeoconchaMelanopsidae

” mentioned in Bogachev (1938: 13, 33, 53) [unavailable]

##### Horizon.

Miocene.

##### Locality.

“Tori bei Borshomi” [Tori near Borjomi], Georgia.

##### Remarks.

The name was only mentioned in a species list by Bogachev without description or illustration. If available, it would be a junior homonym of *Melanopsis nodosa moderata* Mousson, 1874 (see Note 1).

#### Melanopsis neolithica var. moderna

Taxon classificationAnimaliaSorbeoconchaMelanopsidae

Pallary, 1911

##### Original source.

Pallary 1911: 133.

##### Type locality.

“À Colomb-Béchar” [Bechar], Algeria.

#### † “ Melanopsis modesta

Taxon classificationAnimaliaSorbeoconchaMelanopsidae

” mentioned in Bogachev (1938: 13, 33, 53) [unavailable]

##### Horizon.

Miocene.

##### Locality.

“Tori bei Borshomi” [Tori near Borjomi], Georgia.

##### Remarks.

The name was only mentioned in a species list by Bogachev without description or illustration.

#### Melanopsis modica

Taxon classificationAnimaliaSorbeoconchaMelanopsidae

Pallary, 1928

##### Original source.

Pallary 1928b: 19, pl. 2, figs 24–27.

##### Type locality.

“Aïn Foum el Ançeur et Tirboula, près de Ksiba (Moyen Atlas méridional)” [Foum El Anceur and Tirboula, near Ksiba (southern Middle Atlas)], Morocco.

#### † Melanopsis moesiensis

Taxon classificationAnimaliaSorbeoconchaMelanopsidae

Jekelius, 1944

##### Original source.

Jekelius 1944: 132, pl. 50, figs 1–2.

##### Type horizon.

Early Pannonian, late Miocene.

##### Type locality.

“Turislav-Tal bei Soceni” [Turislav valley near Soceni], Romania.

#### † Melanoptychia moesiensis

Taxon classificationAnimaliaSorbeoconchaMelanopsidae

Jekelius, 1944 [invalid]

##### Original source.

Jekelius 1944: 139, pl. 58, figs 1–10.

##### Type horizon.

Early Pannonian, late Miocene.

##### Type locality.

“Turislav-Tal bei Soceni” [Turislav valley near Soceni], Romania.

##### Remarks.

Junior secondary homonym of *Melanopsis moesiensis* Jekelius, 1944 (same work). Neubauer et al. (2014a: 463) considered both synonymous and gave priority to *Melanopsis moesiensis* (since *Melanoptychia* is considered a synonym of *Melanopsis*).

#### Melanopsis mogadorensis

Taxon classificationAnimaliaSorbeoconchaMelanopsidae

Pallary, 1911

##### Original source.

Pallary 1911: 134, [unnumbered plate], figs 27–30.

##### Type locality.

“Dans les sources et la rivière du jardin du Sultan, à Mogador” [in the sources and the river of the garden of the Sultan, at Essaouira], Morocco.

#### Melanopsis mohammedi

Taxon classificationAnimaliaSorbeoconchaMelanopsidae

Bourguignat, 1884

##### Original source.

Bourguignat 1884: 110.

##### Type locality.

“D’Agadir” (Morelet 1880: 71), Morocco.

##### Remarks.

Introduced for *Melanopsis praerosa* [= *Melanopsis praemorsa*] sensu Morelet, 1880, non Linnaeus, 1758. Note that Bourguignat denoted the authority as “Bourguignat, 1881”.

#### † Melanoptychia mojsisovicsi

Taxon classificationAnimaliaSorbeoconchaMelanopsidae

Neumayr, 1880

##### Original source.

Neumayr 1880c: 481, pl. 7, figs 9–10.

##### Type horizon.

Langhian, middle Miocene.

##### Type locality.

“Žepj” [Džepi], Bosnia and Herzegovina.

##### Types.

The type material, with all specimens studied by Neumayr (1880), is lost. Neubauer et al. (2016c: 278) defined a neotype based on material from the type locality. The specimen is stored in the Geological-Paleontological Department, Natural History Museum Vienna, coll. no. 2014/0364/0013.

#### † Melanopsis molinai

Taxon classificationAnimaliaSorbeoconchaMelanopsidae

Vidal, 1917

##### Original source.

Vidal 1917: 353, pl. 2, figs 9–10.

##### Type horizon.

Cretaceous.

##### Type locality.

“Lignitos de Selva y Binisalém” [lignites of Selva and Binissalem], Spain.

#### † Melanopsis bouei var. monacantha

Taxon classificationAnimaliaSorbeoconchaMelanopsidae

Handmann, 1882

##### Original source.

Handmann 1887: 35, pl. 8, figs 8–9.

##### Type horizon.

Pannonian, zone D, late Miocene.

##### Type locality.

“Kottingbrunn [...] Ziegelei a”, Austria.

##### Remarks.

Wenz (1929: 2675) considered this taxon as a junior synonym of *Melanopsis bouei* Férussac, 1823.

#### † “ Melanopsis monolithos

Taxon classificationAnimaliaSorbeoconchaMelanopsidae

” mentioned in Dollfus (1922: 118) [unavailable]

##### Horizon.

Apolakkia/Monolithos Formation, Pliocene.

##### Locality

(Type locality of *Melanopsis biliottii*). “Rhodos” (locality specified as “Monolithos” in Bukowski 1893), Greece.

##### Remarks.

Dollfus mentioned the name as nomen nudum without explanation alongside the discussion of [*Melania costata*] “Var. *Biliottii* Buk.”. Apparently, he considered it a potential new name should *biliottii* be elevated to the species level. If not already a nomen nudum, *Melanopsis monolithos* would therefore be a junior objective synonym of *Melanopsis biliottii* Bukowski, 1892, which actually was introduced as a distinct species (see also Willmann 1981: 105).

#### † Melanopsis impressa var. monregalensis

Taxon classificationAnimaliaSorbeoconchaMelanopsidae

Sacco, 1889

##### Original source.

Sacco 1889: 66, pl. 2, figs 10–12.

##### Type horizon.

Messinian, late Miocene.

##### Type locality.

“Santuario di Mondovi” [Santuario di Vicoforte], Italy.

#### Melanopsis monteli

Taxon classificationAnimaliaSorbeoconchaMelanopsidae

Pallary, 1936

##### Original source.

Pallary 1936: 60, pl. 2, fig. 10.

##### Type locality.

“L’Oued Sous, au pont des Aït Melloul, sur la route d’Agadir à Tiznit, à 14 kil. S. O. d’Agadir” [in the Oued Sous, at the bridge of Ait Melloul, at the road from Agadir to Tiznit, 14 km southwest of Agadir], Morocco.

#### Melania hollandri [sic] var. montenegrina

Taxon classificationAnimaliaSorbeoconchaMelanopsidae

Walderdorff, 1864

##### Original source.

Walderdorff 1864: 512.

##### Type locality.

“In den Bächen Krains” [in rivers of Carniola, a historical region that comprised parts of present-day Slovenia].

#### Melanopsis moreleti

Taxon classificationAnimaliaSorbeoconchaMelanopsidae

Pallary, 1916

##### Original source.

Pallary 1916: 81.

##### Type locality.

“[Sancta-Maria de Balade]” (Morelet 1857: 32) [Balade], New Caledonia.

##### Remarks.

Replacement name for *Melanopsis brevis* Morelet, 1857, non Sowerby, 1826.

#### † Melanopsis morrisi

Taxon classificationAnimaliaSorbeoconchaMelanopsidae

Wenz, 1928

##### Original source.

Wenz 1928b: 220.

##### Type horizon.

Headon Beds, Priabonian, Eocene.

##### Type locality.

“Headon Hill” (Forbes 1856: 156), United Kingdom.

##### Remarks.

Introduced for *Melanopsis subcarinata* Morris in Forbes, 1856, non Deshayes, 1851.

#### Melanopsis mourebeyensis

Taxon classificationAnimaliaSorbeoconchaMelanopsidae

Pallary, 1901

##### Original source.

Pallary 1901b: 228.

##### Type locality.

“Oued Mourebeï sur la route de Merrakech” [Oued Mourebeï (?) at the road to Marrakech], Morocco.

#### Melanopsis moussoni

Taxon classificationAnimaliaSorbeoconchaMelanopsidae

Pallary, 1916 [invalid]

##### Original source.

Pallary 1916: 81.

##### Type locality.

“Dans les eaux de l’ancien Léonthes” (Mousson 1854: 51) [in the Litani river], Lebanon.

##### Remarks.

Replacement name for *Melanopsis brevis* Parreyss in Mousson, 1854, non Sowerby, 1826, for which already Blanckenhorn (1897) had introduced *Melanopsis media* as replacement name. Thus, *Melanopsis moussoni* is a junior objective synonym of *Melanopsis media*.

#### † Melanopsis pygmaea var. mucronata

Taxon classificationAnimaliaSorbeoconchaMelanopsidae

Handmann, 1887

##### Original source.

Handmann 1887: 13, pl. 1, fig. 1.

##### Type horizon.

Pannonian, zone B–D, late Miocene.

##### Type locality.

“Leobersdorf”, Austria.

#### † Melanopsis mucronifera

Taxon classificationAnimaliaSorbeoconchaMelanopsidae

Kormos, 1905

##### Original source.

Kormos 1905: 395, 442, pl. 2, fig. 1.

##### Type horizon.

Late Pleistocene–early Holocene.

##### Type locality.

“Püspökfürdő” [Băile 1 Mai, Lake Pețea], Romania.

##### Remarks.

Obviously unaware of the fact that variety names are available in nomenclature as species-group names, Kormos (1903) stated in the description of his new variety *Melanopsis sikorai carinata* that, if raised to species, he suggests “*Melanopsis mucronifera*” as name for the taxon. Both names were simultaneously published and are objective synonyms. Since *Melanopsis carinata* Kormos is a junior homonym of *Melanopsis carinata* Sowerby, 1826, *Melanopsis mucronifera* is the valid name of the taxon. Neubauer et al. (2014d) considered the taxon as a junior synonym of *Microcolpia parreyssii sikorai* (Brusina, 1903).

#### † Melanopsis bouei var. multicostata

Taxon classificationAnimaliaSorbeoconchaMelanopsidae

Handmann, 1882

##### Original source.

Handmann 1882: 557.

##### Type horizon.

Pannonian, zone D, late Miocene.

##### Type locality.

“Kottingbrunn [...] Ziegelei a”, Austria.

##### Remarks.

The name “*multicostellata*” as mentioned by Handmann (1887: 33) is an incorrect subsequent spelling. Wenz (1929: 2674) considered this variety as a junior synonym of *Melanopsis bouei* Férussac, 1823.

#### † Melanopsis tothi var. multifilosa

Taxon classificationAnimaliaSorbeoconchaMelanopsidae

Brusina, 1903

##### Original source.

Brusina 1903: 114.

##### Type horizon.

Late Pleistocene–early Holocene.

##### Type locality.

“Bischofsbad” [Püspökfürdő, Băile 1 Mai, Lake Pețea], Romania.

##### Remarks.

Considered as a junior synonym of *Microcolpia parreyssii sikorai* (Brusina, 1903) by Neubauer et al. (2014d: 125).

#### † Melanopsis doboi multifilosa

Taxon classificationAnimaliaSorbeoconchaMelanopsidae

Schréter, 1975 [invalid]

##### Original source.

Schréter 1975: 10, pl. 2, figs 11–12, pl. 3, fig. 13.

##### Type horizon.

Riss/Würm end to early Würm Ice Age, Pleistocene.

##### Type locality.

“Eger, az egri vár Zárkándy bástyájának vasúti átmetszése” [Eger, section at the railway at the Zarkandy bastion of the fortress Eger], Hungary.

##### Types.

Magyar Állami Földtani Intézet (Hungarian Geological Museum), Budapest; no number indicated.

##### Remarks.

Junior homonym of Melanopsis tothi var. multifilosa Brusina, 1903. Neubauer et al. (2016a) attributed the species *Melanopsis doboi* to the genus *Microcolpia*.

#### † Melanopsis multiformis

Taxon classificationAnimaliaSorbeoconchaMelanopsidae

Blanckenhorn, 1897

##### Original source.

Blanckenhorn 1897: 116, pl. 9, figs 14–17.

##### Type horizon.

Plio-Pleistocene.

##### Type locality.

“In der ersten Thonbank des linken Orontesufers bei Dschisr esch-Schurr” [in the first clay bank at the left riverside of the Orontes near Jisr Ash-Shughur], Syria.

##### Remarks.

Introduced as “n. mut.” (see also discussion on p. 117) but clearly as a binomen and hence not infrasubspecific in the sense of ICZN Art. 45.6.

#### † Melanopsis munieri

Taxon classificationAnimaliaSorbeoconchaMelanopsidae

Roule, 1884

##### Original source.

Roule 1884: 317, pl. 5, fig. 5.

##### Type horizon.

Calcaire de Rognac, Maastrichtian, Cretaceous.

##### Type locality.

“À Fuveau, Puyloubier, Ollières”, France.

##### Remarks.

Probably not a Melanopsidae.

#### “ Melanopsis muraldi

Taxon classificationAnimaliaSorbeoconchaMelanopsidae

” mentioned in Brot (1874–1879: 427) [unavailable]

##### Locality.

Not indicated.

##### Remarks.

Nomen nudum; apparently based on an unpublished manuscript name from Ziegler and listed in synonymy of *Melanopsis costata* (Olivier, 1804) by Brot (1879).

#### Melanopsis myosotidaea

Taxon classificationAnimaliaSorbeoconchaMelanopsidae

Bourguignat, 1884

##### Original source.

Bourguignat 1884: 93.

##### Type locality.

“Environs d’Agora et d’Alhama (Aragon), en Espagne; Relizane, dans la plaine du Cheliff, en Algérie” [surroundings of Agora (?) and Alhama de Aragón (Spain); Relizane, in the Plaine du Chelif (Algeria)].

#### Melanopsis mzabica

Taxon classificationAnimaliaSorbeoconchaMelanopsidae

Bourguignat, 1884

##### Original source.

Bourguignat 1884: 89.

##### Type locality.

“Ruisseau du puits artésien de Ngouça; Chetma, près de Biskra, et environs d’Ouargla, dans le sud de la province de Constantine” [creek from the artesian well of N’Goussa; Chetma near Biskra and in the surroundings of Ouargla], Algeria.

#### Melanopsis naini

Taxon classificationAnimaliaSorbeoconchaMelanopsidae

Pallary, 1928

##### Original source.

Pallary 1928a: 274, pl. 5, figs 13–14.

##### Type locality.

“Tiout, dans l’Anti Atlas”, Morocco.

#### Melania (Melanella) holandri var. nana

Taxon classificationAnimaliaSorbeoconchaMelanopsidae

Kobelt, 1881 [invalid]

##### Original source.

Kobelt 1881: 149.

##### Type locality.

“Teplica” [thermal springs Teplice (?)], Slovakia.

##### Remarks.

Kobelt (1881) and Clessin (1890) attributed the name to Brot (1874), who did not name the variety but solely described it (nana = Latin “tiny”). Kobelt (1881) referred the name to Brot’s illustration, making it thereby available. However, the name is a junior homonym of *Melania nana* Lea & Lea, 1850.

#### Melanopsis praemorsa variabilis subvar. nana

Taxon classificationAnimaliaSorbeoconchaMelanopsidae

Nevill, 1884 [unavailable]

##### Original source.

Nevill 1884: 208.

##### Type locality.

“Persia; [...] Shiraz”, Iran.

##### Remarks.

Introduced infrasubspecific taxon (“subvariety”), which is not ruled by the provisions of the Code.

#### Melanopsis costata var. nana

Taxon classificationAnimaliaSorbeoconchaMelanopsidae

Paetel, 1888

##### Original source.

Paetel 1888: 400.

##### Type locality.

“Sea of Galilee” (Nevill 1884: 212), Israel.

##### Remarks.

Originally introduced as infrasubspecific taxon (“subvariety”) by Nevill (1884), but made available by Paetel (1888) who treated it as variety (Art. 45.5.1). Paetel clearly referred to the description of Nevill.

#### † Melanopsis narzolina

Taxon classificationAnimaliaSorbeoconchaMelanopsidae

d’Archiac in Viquesnel, 1846

##### Original source.

Viquesnel 1846: 266.

##### Type horizon.

Late Miocene.

##### Type locality.

“Piémont” [no locality indicated], Italy.

##### Remarks.

The name was based on Bonelli’s unpublished catalogue, but was first described in Viquesnel (1846) as “*M.*[*elanopsis*] *narsolina*” [sic]. However, the name *Melanopsis narzolina* - probably the “correct” spelling sensu Bonelli - has been in prevailing usage since then. For reasons of stability the name *narzolina* should be maintained according to Art. 33.3.1 (see Harzhauser et al. 2015: 10).

#### † Melanopsis narzolinensis

Taxon classificationAnimaliaSorbeoconchaMelanopsidae

Pallary, 1920 [invalid]

##### Original source.

Pallary 1920b: 105, 112–113.

##### Type horizon.

Late Miocene.

##### Remarks.

Unjustified emendation and therefore junior objective synonym of *Melanopsis narzolina* d’Archiac in Viquesnel, 1846.

#### † Melanopsis nassaeformis

Taxon classificationAnimaliaSorbeoconchaMelanopsidae

Neumayr, 1880

##### Original source.

Neumayr 1880b: 293, pl. 1, fig. 20.

##### Type horizon.

Pliocene.

##### Type locality.

“Bei Pylle” [near Pýli], Greece.

##### Remarks.

Willmann (1981: 175) considered this taxon as a junior synonym of *Melanopsis gorceixi proteus* Tournouër, 1875.

#### † Melanopsis navarroi

Taxon classificationAnimaliaSorbeoconchaMelanopsidae

Vidal, 1917

##### Original source.

Vidal 1917: 354, pl. 2, figs 11–14.

##### Type horizon.

Cretaceous.

##### Type locality.

“De Binisalém” [from Binissalem], Spain.

#### Melanopsis neolithica

Taxon classificationAnimaliaSorbeoconchaMelanopsidae

Pallary, 1911

##### Original source.

Pallary 1911: 132, [unnumbered plate], figs 18, 23–24.

##### Type locality.

“À Colomb-Béchar” [at Bechar], Algeria.

#### † Melanopsis nereis

Taxon classificationAnimaliaSorbeoconchaMelanopsidae

d’Orbigny, 1852

##### Original source.

d’Orbigny 1852: 3.

##### Type horizon.

Chattian, Oligocene.

##### Type locality.

“Saint-Geours en Marensin, et ceux d’Abesse à Saint-Paul” (Grateloup 1828: 137) [Saint-Geours-de-Maremne and Abesse near Saint-Paul-lès-Dax], France.

##### Remarks.

Introduced for *Melania costata* sensu Grateloup, 1828, non Olivier, 1804.

#### Melanopsis neritiformis

Taxon classificationAnimaliaSorbeoconchaMelanopsidae

Deshayes, 1832

##### Original source.

Deshayes 1830–1832: 438.

##### Type locality.

“Dans l’Ohio et ses affluens” [in the Ohio river and its tributaries], United States.

#### Melanopsis neritoides

Taxon classificationAnimaliaSorbeoconchaMelanopsidae

Gassies, 1858

##### Original source.

Gassies 1858: 371.

##### Type locality.

“Des petits cours d’eau à Balade” [in small streams near Balade], New Caledonia.

##### Remarks.

Brot (1879: 456) considered the species as a junior synonym of *Melanopsis brevis* Morelet, 1857 (which is a junior homonym of *Melanopsis brevis* Sowerby, 1826; see *Melanopsis moreleti* Pallary, 1916).

#### † Melanopsis nesici

Taxon classificationAnimaliaSorbeoconchaMelanopsidae

Brusina, 1893

##### Original source.

Brusina 1893: 202 (Serbian part), 36 (Italian part).

##### Type horizon.

Pannonian, zone D–E, late Miocene.

##### Type locality.

“Ripanj”, Serbia.

##### Types.

Milan et al. (1974: 95) indicated collection numbers for “syntypes” illustrated in Brusina (1902), but it is uncertain whether the specimens were part of the original type series. They are stored in the Croatian Natural History Museum, Zagreb, coll. no. 2533-179/1-4.

##### Remarks.

The name “*nesicii*” as mentioned in Stepanović (1938) is an incorrect subsequent spelling.

#### † Melanopsis neumayri

Taxon classificationAnimaliaSorbeoconchaMelanopsidae

Tournouër, 1874

##### Original source.

Tournouër 1874: 303, pl. 9, fig. 5.

##### Type horizon.

Mammal zone MN 11, late Miocene.

##### Type locality.

“Visan”, France.

#### Melanopsis nicollei

Taxon classificationAnimaliaSorbeoconchaMelanopsidae

Pallary, 1923

##### Original source.

Pallary 1923: 43, [unnumbered plate], figs 17–18.

##### Type locality.

“Gafsa, dans les eaux refroidies” [Gafsa, in cool waters], Tunisia.

#### Melanopsis doriae var. nigra

Taxon classificationAnimaliaSorbeoconchaMelanopsidae

Biggs, 1934

##### Original source.

Biggs 1934: 60.

##### Type locality.

“Qanat at Ginehkan near Kerman, S. Persia”, Iran.

#### “ Melanopsis nivosa

Taxon classificationAnimaliaSorbeoconchaMelanopsidae

Fer.” mentioned in Paetel (1888: 402) [unavailable]

##### Locality.

“Mesopot.” [Mesopotamia, no locality indicated], Iraq.

##### Remarks.

Nomen nudum, found only in the species list by Paetel (1888). He referred to Bourguignat (1884), but the name could not be found in that work.

#### † Melanopsis nobilis

Taxon classificationAnimaliaSorbeoconchaMelanopsidae

Seninski, 1905

##### Original source.

Seninski 1905: 61, pl. 2, figs 1–2.

##### Type horizon.

Duab Beds, middle to late Kimmerian, Pliocene.

##### Type locality.

“P. Дуабъ” [Duab river], Georgia.

#### Melanopsis nobilis

Taxon classificationAnimaliaSorbeoconchaMelanopsidae

Pallary, 1912 [invalid]

##### Original source.

Pallary 1912: 20, fig. 41.

##### Type locality.

“Des bords du Chott Djerid, à Tozeur” [banks of the Chott el Djérid at Tozeur], Tunisia.

##### Remarks.

Junior homonym of *Melanopsis nobilis* Seninski, 1905.

#### Fagotia nocturna

Taxon classificationAnimaliaSorbeoconchaMelanopsidae

Bourguignat, 1884

##### Original source.

Bourguignat 1884: 44.

##### Type locality.

“La Save à Agram et la rivière entre Plaski et Ostaria (Croatie); le Danube à Ibraïla” [Sava river at Zagreb and river between Plaški and Oštarije (Croatia); Danube river at Brăila (Romania)].

##### Remarks.

Note that Bourguignat denoted the authority as “Bourguignat, 1879”. Starobogatov et al. (1992: 60) considered the species as a junior synonym of *Fagotia* [= *Esperiana*] *esperi* (Férussac, 1823).

#### † Melanopsis nodescens

Taxon classificationAnimaliaSorbeoconchaMelanopsidae

Handmann, 1882

##### Original source.

Handmann 1882: 557.

##### Type horizon.

Pannonian, zone D, late Miocene.

##### Type locality.

“Kottingbrunn [...] Ziegelei a”, Austria.

##### Remarks.

Handmann (1887) listed a *Melanopsis varicosa nodescens*. It is unclear whether he actually introduced a new variety or ranked the species described in 1882 as variety of *Melanopsis varicosa* Handmann, 1882. Wenz (1929: 2674) considered *Melanopsis nodescens* Handmann, 1882 as a junior synonym of *Melanopsis bouei* Férussac, 1823.

#### † Melanopsis nodicincta

Taxon classificationAnimaliaSorbeoconchaMelanopsidae

Pallary, 1920

##### Original source.

Pallary 1920b: 111.

##### Type horizon.

Late Villafranchian, early Pleistocene.

##### Type locality.

“Roccantica presso Poggio Mirteto; Fra Otricoli e le Vigne” (De Stefani 1880: 62–63) [Roccantica near Poggio Mirteto; between Otricoli and Le Vigne], Italy.

##### Remarks.

Introduced for *Melanopsis nodosa* sensu De Stefani, 1880, non Férussac, 1822. Wenz (1929: 2654) considered the taxon as a junior synonym of “*Melanopsis affinis* Férussac”, which is not an available name.

#### † Melanopsis (Canthidomus) nodifera

Taxon classificationAnimaliaSorbeoconchaMelanopsidae

Handmann, 1887

##### Original source.

Handmann 1887: 30, pl. 7, figs 4–6.

##### Type horizon.

Pannonian, zone B–D, late Miocene.

##### Type locality.

“Leobersdorf”, Austria.

#### † Melanopsis nodosa

Taxon classificationAnimaliaSorbeoconchaMelanopsidae

Férussac, 1822

##### Original source.

Férussac 1819–1851: Mélanopsides fossiles, pl. 1 (1822), fig. 13.

##### Type horizon.

Late Villafranchian, early Pleistocene.

##### Type locality.

“Entre Otricoli et le Vigne, route de Rome à Foligno” [between Otricoli and Le Vigne, at the road from Rome to Foligno], Italy.

##### Remarks.

The name first appeared in 1822 on the captions for plate 1 of the “Mélanopsides fossiles” in Férussac’s “Histoire naturelle” (see also introduction for details). While Férussac (1823) included also recent specimens under that name in his monograph on the Melanopsidae, the only specimen illustrated in 1822 in the “Histoire naturelle” was a fossil one from the Villafranchian of Italy. This fact remained widely unknown to biologists and paleontologists alike. The consequence, however, is that probably none of the specimens referred to as *Melanopsis nodosa* in the biological literature (e.g., Pallary 1939, Glaubrecht 1993, 1996) or the IUCN Red List (Van Damme 2014), which reports it from Anatolia and the Middle East, actually correspond to the real *Melanopsis nodosa*.

Fossil Italian specimens are often erroneously referred to as “*Melanopsis affinis* Férussac” or “*Melanopsis antiqua* Fèrussac” (Pallary 1916, Wenz 1929, Esu and Girotti 1975), both of which are unavailable names.

#### † Melanopsis nodosa

Taxon classificationAnimaliaSorbeoconchaMelanopsidae

Pecchioli, 1864 [invalid]

##### Original source.

Pecchioli 1864: 522, pl. 5, figs 19–20.

##### Type horizon.

Villafranchian, Plio-Pleistocene.

##### Type locality.

“Orciano”, Italy.

##### Remarks.

Junior homonym of *Melanopsis nodosa* Férussac, 1822. De Stefani (1877) introduced *Melanopsis semperi* as replacement name.

#### † Melanopsis costata var. nodosa

Taxon classificationAnimaliaSorbeoconchaMelanopsidae

Brusina, 1874 [invalid]

##### Original source.

Brusina 1874: 41.

##### Type horizon.

Cernikian, Pliocene.

##### Type locality.

“Podvinje (Čaplja) [Čaplja trench near Slavonski Brod]; Kovačevac; Novska; Farkašić; Dubranjec”, Croatia.

##### Remarks.

Junior homonym of *Melanopsis nodosa* Férussac, 1822. Wenz (1928a) introduced *Melanopsis nodosula* as replacement name.

#### † Melanopsis nodosa

Taxon classificationAnimaliaSorbeoconchaMelanopsidae

Handmann, 1882 [invalid]

##### Original source.

Handmann 1882: 556.

##### Type horizon.

Pannonian, zone D, late Miocene.

##### Type locality.

“Kottingbrunn [...] Ziegelei a”, Austria.

##### Remarks.

Junior homonym of *Melanopsis nodosa* Férussac, 1822. Pallary (1916: 80) introduced *Melanopsis venusta* as replacement name. Wenz (1929: 2673) considered the taxon as a junior synonym of *Melanopsis bouei* Férussac, 1823.

#### † Melanopsis (Canthidomus) nodosa

Taxon classificationAnimaliaSorbeoconchaMelanopsidae

Doncieux, 1908 [invalid]

##### Original source.

Doncieux 1908: 203, pl. 11, figs 11a–d.

##### Type horizon.

Middle Lutetian, Eocene.

##### Type locality.

“Au Nord d’Albas”, France.

##### Remarks.

Junior homonym of *Melanopsis nodosa* Férussac, 1822.

#### “ Melania nodosa

Taxon classificationAnimaliaSorbeoconchaMelanopsidae

” mentioned in Reeve (1860) and Brot (1874–1879: 13) [unavailable]

##### Locality.

Not indicated.

##### Remarks.

Nomen nudum, apparently based on an unused manuscript name from Parreyss (see Reeve 1860) or Stentz (see Brot 1874). Reeve (1860) considered the taxon as a junior synonym of *Melania hollandri* [sic]. If available, it would be a junior homonym of *Melania nodosa* Münster, 1841. Note that the latter species is certainly no Melanopsidae as it derives from the marine deposits of the Triassic St. Cassian Fm.

#### † Melanopsis nodosula

Taxon classificationAnimaliaSorbeoconchaMelanopsidae

Wenz, 1928

##### Original source.

Wenz 1928a: 119.

##### Type horizon.

Cernikian, Pliocene.

##### Type locality.

“Podvinje (Čaplja) [Čaplja trench near Slavonski Brod]; Kovačevac; Novska; Farkašić; Dubranjec” (Brusina 1874: 41), Croatia.

##### Remarks.

Replacement name for *Melania costata nodosa* Brusina, 1874, non Férussac, 1822 (see Note 1).

#### † Melanopsis inconstans var. nodulosa

Taxon classificationAnimaliaSorbeoconchaMelanopsidae

Brusina, 1874

##### Original source.

Brusina 1874: 39.

##### Type horizon.

Langhian, middle Miocene.

##### Type locality.

“Miočić”, Croatia.

##### Types.

The syntypes are stored in the Croatian Natural History Museum, Zagreb; no number indicated (Milan et al. 1974: 92).

##### Remarks.

Wenz (1929: 2756) and Neubauer et al. (2016b: 23) considered this taxon as a junior synonym of *Melanopsis inconstans* Neumayr, 1869.

#### † Melanopsis (Lyrcea) delessei var. nodulosa

Taxon classificationAnimaliaSorbeoconchaMelanopsidae

Magrograssi, 1928 [invalid]

##### Original source.

Magrograssi 1928: 259, pl. 6, fig. 16.

##### Type horizon.

Plio-Pleistocene.

##### Type locality.

“Coo: V. Armiri” [Kos island: Armiri valley (?)], Greece.

##### Remarks.

Junior homonym of *Melanopsis inconstans nodulosa* Brusina, 1874 (see Note 1).

#### Melanopsis noetlingi

Taxon classificationAnimaliaSorbeoconchaMelanopsidae

Bourguignat in Noetling, 1886

##### Original source.

Noetling 1886: 817, pl. 23, fig. 6.

##### Type locality.

“Aus dem Jarmūk bei el Hammi [...] auch bei el-Hāwijān [fossil]” [from the Yarmuk river at Al Ḩammah and as fossil near Jisr al Ḩāwī], Jordan.

##### Remarks.

Noetling clearly referred to “Bourguignat’s new species”, which is why the authority should read as “Bourguignat in Noetling, 1886”. Heller and Sivan (2002b: 615) considered the taxon as a junior synonym of *Melanopsis multiformis* Blanckenhorn, 1897. Heller et al. (2005: 247) in turn treated it as a junior synonym of *Melanopsis obliqua* Bourguignat, 1884.

#### † Melanopsis constricta nowskaensis

Taxon classificationAnimaliaSorbeoconchaMelanopsidae

Wenz, 1928

##### Original source.

Wenz 1928a: 120.

##### Type horizon.

Cernikian, Pliocene.

##### Type locality.

“Novska (Bukovica)” (Brusina 1897: 7), Croatia.

##### Types.

Milan et al. (1974: 88) indicated a holotype, but it is uncertain whether the specimen was the only one Brusina had at hand when describing *M.* [*constricta*] *subcostata* (holotype by monotypy, Art. 73.1.2). The specimen is stored in the Croatian Natural History Museum, Zagreb, coll. no. 2993-639.

##### Remarks.

Replacement name for *Melanopsis constricta subcostata* Brusina, 1897, non *Melanopsis subcostata* d’Orbigny, 1850. The name “*novskaensis*” as mentioned in Wenz (1929: 2696) is an incorrect subsequent spelling.

#### Melanopsis obediensis

Taxon classificationAnimaliaSorbeoconchaMelanopsidae

Picard, 1934

##### Original source.

Picard 1934: 110, pl. 7, figs 30–44.

##### Type locality.

“Obedieh” [El ‘Ubeidīya], Israel.

#### † Melania obeloides

Taxon classificationAnimaliaSorbeoconchaMelanopsidae

Tausch, 1886

##### Original source.

Tausch 1886: 7, pl. 1, figs 16–19.

##### Type horizon.

Late Santonian–early Campanian, late Cretaceous.

##### Type locality.

“Csingerthal bei Ajka” [Csinger valley near Ajka], Hungary.

##### Remarks.

The species was attributed to the genus *Esperiana* by Bandel and Riedel (1994: 20). That classification was, however, doubted by Neubauer et al. (2016a: 127) based on the large stratigraphic gap between this Cretaceous species and other representatives that appeared not before the late Miocene.

#### Melanopsis praemorsa var. obesa

Taxon classificationAnimaliaSorbeoconchaMelanopsidae

Gassies, 1856

##### Original source.

Gassies 1856: 12, figs 11–12.

##### Type locality.

“Dans l’Oued-Lisser, sur la route de Sidi-bel-Abess, à Tlemcen” [in the Oued Lisser (?), on the road to Sidi Bel Abbès, at Tlemcen], Algeria.

##### Remarks.

Bourguignat (1884) attributed the authority to himself, but even listed Gassies’ record in synonymy. Apparently, he did not consider a variety as a valid name that can result in homonymy (see also the introduction).

#### Melanopsis obesa

Taxon classificationAnimaliaSorbeoconchaMelanopsidae

Brot, 1868 [invalid]

##### Original source.

Brot 1868: 57, pl. 1, figs 14–15.

##### Type locality.

“Prope Cehejin Prov. Murcia” [near Cehegín, prov. Murcia], Spain.

##### Remarks.

The species was described as “*Melanopsis obesa* Guirao mss.”. Although Brot (1868) denoted “(Guirao)” after the description of the species as well, it is clear from the text that Brot described it. Junior homonym of *Melanopsis obesa* Gassies, 1856. Bourguignat (1884) introduced *Melanopsis guiraoi* as replacement name.

#### “ Melanopsis costata var. obesa

Taxon classificationAnimaliaSorbeoconchaMelanopsidae

” mentioned in Locard (1883a: 202, 230) and Bourguignat (1884: 141) [unavailable]

##### Locality.

“Le Jourdain et le lac de Tibériade” [river Jordan and Sea of Galilee], Israel.

##### Remarks.

Nomen nudum. If available, it would be a junior homonym of *Melanopsis obesa* Gassies, 1856.

#### † Melanopsis (Smendovia) doumerguei var. obesa

Taxon classificationAnimaliaSorbeoconchaMelanopsidae

Pallary, 1901 [invalid]

##### Original source.

Pallary 1901a: 177, pl. 2, fig. 29.

##### Type horizon.

Late Miocene.

##### Type locality.

“Smendou” [Zighoud Youcef], Algeria.

##### Remarks.

Junior homonym of *Melanopsis obesa* Gassies, 1856. Wenz (1928b) introduced *Melanopsis doumerguei plena* as replacement name.

#### † Melanopsis pygmaea obesa

Taxon classificationAnimaliaSorbeoconchaMelanopsidae

Brusina, 1902 [invalid]

##### Original source.

Brusina 1902: pl. 5, figs 39–41.

##### Type horizon.

Middle Pannonian, late Miocene.

##### Type locality.

“Markuševec”, Croatia.

##### Types.

The illustrated syntypes are stored in the Croatian Natural History Museum, Zagreb, coll. no. 2492-138, 2493-139 (Milan et al. 1974: 96).

##### Remarks.

Junior homonym of *Melanopsis obesa* Gassies, 1856. It was considered as a junior synonym of *Melanopsis pygmaea* Hörnes, 1856 by Wenz (1929: 2814).

#### Melanopsis laevigata var. obesula

Taxon classificationAnimaliaSorbeoconchaMelanopsidae

Pallary, 1904

##### Original source.

Pallary 1904: 37.

##### Type locality.

“De l’Aïn bou Smelal près de Tétouan” [‘Aïn bou Smelal (?) near Tetouan], Morocco.

#### Melanopsis obliqua

Taxon classificationAnimaliaSorbeoconchaMelanopsidae

Bourguignat, 1884

##### Original source.

Bourguignat 1884: 138.

##### Type locality.

“Le Bélus, près de Saint-Jean-d’Acre” [in the Na’aman river, near Acre], Israel.

##### Remarks.

Bourguignat (1884) denoted the authority as “Letourneux, 1882”, but there is no evidence that the description really derived from that author.

#### Melanopsis costata var. obliquata

Taxon classificationAnimaliaSorbeoconchaMelanopsidae

Pallary, 1939

##### Original source.

Pallary 1939: 91, pl. 5, fig. 34.

##### Type locality.

“Djishr ech Chegour” [Jisr Ash-Shughur], Syria.

##### Remarks.

Heller and Sivan (2002a: 49) considered the taxon as a junior synonym of *Melanopsis multiformis* Blanckenhorn, 1897. Heller et al. (2005: 244) in turn treated it as a junior synonym of *Melanopsis costata* (Olivier, 1804).

#### † Melanopsis oblonga

Taxon classificationAnimaliaSorbeoconchaMelanopsidae

Blanckenhorn, 1897

##### Original source.

Blanckenhorn 1897: 137, pl. 10, fig. 25.

##### Type horizon.

Plio-Pleistocene.

##### Type locality.

“In der Dreissensiaschicht von Dschisr esch-Schurr” [in the *Dreissena* layers at Jisr Ash-Shughur], Syria.

#### † Melanopsis obsoleta

Taxon classificationAnimaliaSorbeoconchaMelanopsidae

Fuchs, 1873

##### Original source.

Fuchs 1873: 20, pl. 4, figs 14–15.

##### Type horizon.

Transdanubian, Pannonian, late Miocene.

##### Type locality.

“Radmanest” [Rădmănești], Romania.

#### Melanopsis costata var. obsoleta

Taxon classificationAnimaliaSorbeoconchaMelanopsidae

Martens, 1874 [invalid]

##### Original source.

Martens 1874: 33, p. 5, fig. 39.

##### Type locality.

“Quellen des Chabur bei Ras-el-Ain” [source of Chabur river near Ra’s al ‘Ayn], Syria.

##### Remarks.

Junior homonym of *Melanopsis obsoleta* Fuchs, 1873.

#### Melanopsis saulcyi var. obsoleta

Taxon classificationAnimaliaSorbeoconchaMelanopsidae

Dautzenberg, 1894 [invalid]

##### Original source.

Dautzenberg 1894: 345.

##### Type locality.

“Palmyre, dans un ruisseau et dans la rivière Ephéca” [Palmyra, in a stream and the river Epheca (?)], Syria.

##### Remarks.

Junior homonym of *Melanopsis obsoleta* Fuchs, 1873.

#### † Melanopsis obtusa

Taxon classificationAnimaliaSorbeoconchaMelanopsidae

Deshayes, 1825

##### Original source.

Deshayes 1824–1837: 123, pl. 14, figs 22–23.

##### Type horizon.

Cuisian, late Ypresian, Eocene.

##### Type locality.

“Retheuil près de Pierre-Fonds” [Retheuil near Pierrefonds, Dép. Aisne], France.

##### Remarks.

Sandberger (1872: 202) classified the species within his new genus *Coptostylus* (Thiaridae), which was followed by Wenz (1929: 2570).

#### Melanopsis obtusa

Taxon classificationAnimaliaSorbeoconchaMelanopsidae

Pallary, 1920 [invalid]

##### Original source.

Pallary 1920a: 30.

##### Type locality.

“Meknès, près de anciennes écuries” [Meknes, near the old stables], Morocco.

##### Remarks.

Junior homonym of *Melanopsis obtusa* Deshayes, 1824.

#### Melanopsis praerosa [sic] var. ocuticarinata

Taxon classificationAnimaliaSorbeoconchaMelanopsidae

Paetel, 1888

##### Original source.

Paetel 1888: 403.

##### Type locality.

“Persia” (Nevill 1884: 209), Iran.

##### Remarks.

Nevill (1884) first mentioned the name “*acuticarinata*” [sic] for an infrasubspecific taxon (“subvariety”), which he briefly described. Paetel (1888) made the name available by ranking it as species-group name and referring to the description of Nevill (Art. 45.5.1). Although he obviously “misspelt” the name, *ocuticarinata* remains the valid spelling because Nevill’s “*acuticarinata*” is not available as species-group name.

#### † Melanopsis ogerieni

Taxon classificationAnimaliaSorbeoconchaMelanopsidae

Locard, 1883

##### Original source.

Locard 1883b: 92, 140, pl. 3, figs 3–4.

##### Type horizon.

Mammal zone MN 9–12, late Miocene.

##### Type locality.

“Priay; [...] Niquedet” (p. 150), France.

#### † “ Melanopsis oliva

Taxon classificationAnimaliaSorbeoconchaMelanopsidae

” mentioned in Hoeninghaus (1831: 143) [unavailable]

##### Horizon.

Burdigalian, early Miocene (?).

##### Locality.

“Dax”, France.

##### Remarks.

Nomen nudum. If available, it would be a junior homonym of *Melanopsis oliva* De Cristofori & Jan, 1832.

#### Melanopsis oliva

Taxon classificationAnimaliaSorbeoconchaMelanopsidae

De Cristofori & Jan, 1832

##### Original source.

De Cristofori and Jan 1832: 4.

##### Type locality.

“Am. mer.” [South America], indicated in the previous part of the same work (“Conchylia terrestria et fluviatilia [...]”, p. 7; there, the name is a nomen nudum).

##### Remarks.

Certainly not a Melanopsidae.

#### Melanopsis olivieri

Taxon classificationAnimaliaSorbeoconchaMelanopsidae

Bourguignat, 1884

##### Original source.

Bourguignat 1884: 98.

##### Type locality.

“Entre Ain-Taib et Alep, à Sadjour-Sou; [...] du Nahr-el-Kelb, près Beyrouth; de divers cours d’eau du Liban; de Serghaia dans l’ouady Baradah près de Damas; de la fontaine Jérémie près de Jéricho; environs de Constantinople” [Gaziantep (Turkey) and Aleppo (Syria), at Sadjour-Sou (?); in Nahr el-Kalb near Beirut (Lebanon); in various streams of Lebanon; in Serghaia (?) in River Barada near Damascus (Syria); Jeremiah Fountain (?) near Jericho (Palestine); near Istanbul (Turkey)].

#### † Melanopsis buccinoidea var. olivula

Taxon classificationAnimaliaSorbeoconchaMelanopsidae

Grateloup, 1828

##### Original source.

Grateloup 1828: 137.

##### Type horizon.

Burdigalian, early Miocene.

##### Type locality.

“Mandillot”, France.

#### Melanopsis olivula

Taxon classificationAnimaliaSorbeoconchaMelanopsidae

Letourneux & Bourguignat, 1887 [invalid]

##### Original source.

Letourneux and Bourguignat 1887: 156.

##### Type locality.

“À Nefta, dans les canaux d’arrosement et à El-Hammam, près Tozer” [at Nefta, in the irrigation channel, and at El-Hamma-Djerid near Tozeur], Tunisia.

##### Remarks.

Junior homonym of *Melanopsis olivula* Grateloup, 1838. Pallary (1916: 84) was aware of the homonymy issue but did not introduce a replacement name for he considered the taxon as a junior synonym of *Melanopsis doumeti* Letourneux & Bourguignat, 1887.

#### † Melanopsis oltszakadatensis

Taxon classificationAnimaliaSorbeoconchaMelanopsidae

Halaváts, 1914

##### Original source.

Halaváts 1914: 421, six unnumbered textfigures.

##### Type horizon.

Sarmatian (sensu stricto), middle Miocene.

##### Type locality.

“Graben unterhalb der Gemeinde-Baumschule östlich von Oltszakadát” [trench below communal tree nursery, east of Săcădate], Romania.

#### † Melanopsis (Lyrcea) onusta

Taxon classificationAnimaliaSorbeoconchaMelanopsidae

Stefanescu, 1896

##### Original source.

Stefanescu 1896: 131, pl. 11, figs 38–41.

##### Type horizon.

Pliocene.

##### Type locality.

“À Plostina, à Leurda et à Stângaceana, dans la vallée de Motru; à Valea-lui-Câne, dans la vallée de Gilortu; à Breasta, à Bocovatz et à Bâzdâna, dans la vallée de Jiu; à Beceni, dans la vallée de Slanic de Buzau; à Plaiu et à Chiojdeni, dans la vallée de Râmnicu-Sarat” [at Ploștina, at Leurda and at Stângăceaua, in the valley of the river Motru; at Gilortu in the valley of the river Gilortu; at Breasta, at Bucovăț and at Bâzdâna, in the valley of the river Jiu; at Beceni, in the valley of the river Slănic; at Plaiu and at Chiojdeni, in the valley of the river Râmnic], Romania.

#### † Melanopsis onychia

Taxon classificationAnimaliaSorbeoconchaMelanopsidae

Brusina, 1874

##### Original source.

Brusina 1874: 30, pl. 1, figs 3–4.

##### Type horizon.

Cernikian, Pliocene.

##### Type locality.

“Bečić; Podvinje (Čaplja) [Čaplja trench near Slavonski Brod]; Sibinj”, Croatia.

##### Types.

Milan et al. (1974: 95) indicated a holotype, but it is uncertain whether the specimen was the only one Brusina had at hand (holotype by monotypy, Art. 73.1.2). The specimen is stored in the Croatian Natural History Museum, Zagreb, coll. no. 3206-852/1.

#### † Melanopsis oomorpha

Taxon classificationAnimaliaSorbeoconchaMelanopsidae

De Stefani, 1877

##### Original source.

De Stefani 1877: 284.

##### Type horizon.

Villafranchian, Plio-Pleistocene.

##### Type locality.

“Spoleto [...], Orciano [...], fra S. Gemini e Carsoli” [Spoleto, Orciano Pisano, Carsoli near San Gemini], Italy.

##### Remarks.

Introduced for *Melania buccinoidea* sensu Férussac, 1823, non Olivier, 1801 (partim) as well as for formerly misidentified *Melanopsis narzolina*. Esu and Girotti (1975: 251) considered this taxon as a junior synonym of “*Melanopsis affinis* Férussac”, which is not an available name.

#### † Melanopsis oranensis

Taxon classificationAnimaliaSorbeoconchaMelanopsidae

Pallary, 1926

##### Original source.

Pallary 1926c: 286, figs 1–4.

##### Type horizon.

Pliocene?

##### Type locality.

“D’un puits, profond de 25 mètres, situé dans la propriété Lamur, sur la rive gauche d’un ravinement creusé par les eaux pluviales dans une dépression de terrain, perpendiculaire au chemin d’Aïn Beïda” (p. 284) [from a well, 25 m deep, located in the village Lamur (near Oran) on the left bank of a ravine carved by rainwater in a depression in the ground, perpendicular to the path from ‘Aïn el Beïda], Algeria.

##### Remarks.

Pallary cited in the synonymy list his paper on the fauna of the “Berbérie”, which appeared in the Journal de Conchyliologie. That work, however, was not published before March 1928.

#### † Melanopsis orientalis

Taxon classificationAnimaliaSorbeoconchaMelanopsidae

Bukowski, 1892

##### Original source.

Bukowski 1892: 249.

##### Type horizon.

Apolakkia/Monolithos Formation, Pliocene.

##### Type locality.

“Rhodos” (locality specified as “Monolithos” in Bukowski 1893), Greece.

#### “ Melanopsis orientalis

Taxon classificationAnimaliaSorbeoconchaMelanopsidae

Von dem Busch” mentioned in Mousson (1854: 50) [unavailable]

##### Locality.

Not indicated (except from “de l’Orient”).

##### Remarks.

Nomen nudum, listed by Mousson without any explanation.

#### † Melanopsis ornata

Taxon classificationAnimaliaSorbeoconchaMelanopsidae

Deshayes, 1862

##### Original source.

Deshayes 1861–1864: 474, pl. 31, figs 27–28.

##### Type horizon.

Sparnacian, early Ypresian, Eocene.

##### Type locality.

“Sainceny”, France.

##### Remarks.

Type species of *Eginea* Pacaud & Harzhauser, 2012 (Pachychilidae).

#### † Paludina (Vivipara) ornata

Taxon classificationAnimaliaSorbeoconchaMelanopsidae

Fuchs, 1877

##### Original source.

Fuchs 1877: 6, pl. 1, fig. 2.

##### Type horizon.

Pliocene.

##### Type locality.

“Kalamaki” [near Corinth], Greece.

##### Remarks.

Since *Paludina* Férussac, 1812 is a junior objective synonym of *Viviparus* Montfort, 1810, this species is a junior homonym of *Vivipara ornata* Neumayr in Neumayr & Paul, 1875. Wenz (1928a) recombined the species with *Amphimelania* and introduced *Amphimelania fuchsi* as replacement name.

#### Melanopsis orontis

Taxon classificationAnimaliaSorbeoconchaMelanopsidae

Pallary, 1939

##### Original source.

Pallary 1939: 92, pl. 6, figs 70–72.

##### Type locality.

“L’Oronte à Djisr ech Chogour” [in the Orontes at Jisr Ash-Shughur], Syria.

#### Melanopsis orophila

Taxon classificationAnimaliaSorbeoconchaMelanopsidae

Pallary, 1920

##### Original source.

Pallary 1920b: 117.

##### Type locality.

“M. Mario: Farnesina” (Cerulli-Irelli 1914: 183), Italy.

##### Remarks.

Introduced for *Melanopsis praemorsa* sensu Cerulli-Irelli, 1914, non Linnaeus, 1758.

#### † Melanopsis (Canthidomus) oroposi

Taxon classificationAnimaliaSorbeoconchaMelanopsidae

Papp, 1979

##### Original source.

Papp 1979: 672, pl. 3, figs 1–15.

##### Type horizon.

Mammal zone MN 10, late Miocene.

##### Type locality.

“Milessi” [Milesi], Greece.

##### Types.

Institute of Paleontology, University of Vienna; no number indicated.

#### † Melanopsis buccinoidea var. ovalis

Taxon classificationAnimaliaSorbeoconchaMelanopsidae

Grateloup, 1838

##### Original source.

Grateloup 1838: 146.

##### Type horizon.

Sparnacian, early Ypresian, Eocene.

##### Type locality.

“Épernay”, France.

##### Remarks.

Introduced for “*Melania buccinoidea* var. a” in Férussac (1823: 149, pl. 1 [= 7], fig. 1), non Olivier, 1801.

#### Melanopsis ovata

Taxon classificationAnimaliaSorbeoconchaMelanopsidae

Dunker, 1862

##### Original source.

Dunker 1862: 150.

##### Type locality.

“Rotoitisee” [Lake Rotoiti], New Zealand.

##### Remarks.

Suter (1893: 139) considered the taxon as a junior synonym of *Zemelanopsis trifasciata* (Gray in Dieffenbach, 1843).

#### † Melanopsis ovicona

Taxon classificationAnimaliaSorbeoconchaMelanopsidae

Deffner & Fraas, 1877

##### Original source.

Deffner and Fraas 1877: 16.

##### Type horizon.

Mammal zone MN 6, middle Miocene.

##### Type locality.

“Trendel” [in Nördlinger Ries], Germany.

##### Remarks.

Wenz (1929: 2763), who erroneously considered the taxon as a nomen nudum, listed it in synonymy of *Melanopsis kleinii* Kurr, 1856.

#### Melanella ovoidaea

Taxon classificationAnimaliaSorbeoconchaMelanopsidae

Bourguignat, 1884

##### Original source.

Bourguignat 1884: 22.

##### Type locality.

“Le Danube au-dessus de Routschouk (Bulgarie)” [in the Danube river below Ruse], Bulgaria.

##### Remarks.

Note that Bourguignat denoted the authority as “Bourguignat, 1879”.

#### Melanopsis nodosa var. ovoidalis

Taxon classificationAnimaliaSorbeoconchaMelanopsidae

Cerulli-Irelli, 1914

##### Original source.

Cerulli-Irelli 1914: 184, pl. 15 (47), figs 5–6.

##### Type locality.

“M. Mario: Farnesina; Acuatraversa”, Italy.

##### Remarks.

Girotti (1972: 232) considered the taxon as a junior synonym of “*Melanopsis affinis* Férussac”, which is not an available name.

#### † Melanopsis ovosimilis

Taxon classificationAnimaliaSorbeoconchaMelanopsidae

Willmann, 1981

##### Original source.

Willmann 1981: 143.

##### Type horizon.

Vasilios Formation, middle Tortonian, late Miocene.

##### Type locality.

“Dermen Deressi 400 m westlich des Asklepion, ca. 3 km westlich von Kos-Ort/Kos” [Dermen Deressi 400 m west of Asklepion, ca. 3 km west of Kos city, Kos Island], Greece.

##### Types.

Geological-Paleontological Institute, University of Kiel, Germany; no number indicated.

#### Melanopsis ovula

Taxon classificationAnimaliaSorbeoconchaMelanopsidae

Bourguignat, 1884

##### Original source.

Bourguignat 1884: 162.

##### Type locality.

“Le Guadalquivir entre Séville et Cordoue” [in the Guadalquivir river between Sevilla and Córdoba], Spain.

#### † Melanopsis ovularis

Taxon classificationAnimaliaSorbeoconchaMelanopsidae

Watelet, 1853

##### Original source.

Watelet 1853: 18, pl. 2, figs 18–19.

##### Type horizon.

Cuisian, late Ypresian, Eocene.

##### Type locality.

“Mercin” [Mercin-et-Vaux], France.

##### Remarks.

Watelet attributed the authority to Deshayes, but there is no evidence that the description really derived from that author.

#### Melanopsis ovum

Taxon classificationAnimaliaSorbeoconchaMelanopsidae

Bourguignat, 1884

##### Original source.

Bourguignat 1884: 143.

##### Type locality.

“See Tiberias” (Kobelt 1880: 17) [Sea of Galilee], Israel.

##### Remarks.

Appeared first as a nomen nudum in Locard (1883a: 202). Introduced for “*Melanopsis costata* var.” in Kobelt (1880: 17, pl. 188, fig. 1906). Heller et al. (2005: 246) considered the species as a junior synonym of *Melanopsis jordanica* Roth, 1839.

#### † Melanopsis oxyacantha

Taxon classificationAnimaliaSorbeoconchaMelanopsidae

Brusina, 1902

##### Original source.

Brusina 1902: pl. 7, figs 6–7.

##### Type horizon.

Transdanubian, Pannonian, late Miocene.

##### Type locality.

“Tihany”, Hungary.

##### Types.

The illustrated syntypes are stored in the Croatian Natural History Museum, Zagreb, coll. no. 2534-180/1-2 (Milan et al. 1974: 95).

##### Remarks.

The name “*oxicanthe*” as mentioned in Miletić-Spajić (1961: 252) is an incorrect subsequent spelling.

#### † Melanopsis (Lyrcea) pachecoi

Taxon classificationAnimaliaSorbeoconchaMelanopsidae

Royo Gómez, 1922

##### Original source.

Royo Gómez 1922: 108.

##### Type horizon.

Late Miocene.

##### Type locality.

“Sierra de la Pinada en Sayatón (Guadalajara)”, Spain.

#### Melanopsis pachya

Taxon classificationAnimaliaSorbeoconchaMelanopsidae

Pallary, 1939

##### Original source.

Pallary 1939: 86, pl. 5, figs 10, 12.

##### Type locality.

“Dans les sources de Mézérib, au N. O. de Derâa (Syrie méridionale)” [in the sources of the Muzayrīb, northwest of Dar’ā], Syria.

#### Microcolpia pachystoma

Taxon classificationAnimaliaSorbeoconchaMelanopsidae

Bourguignat, 1884

##### Original source.

Bourguignat 1884: 66.

##### Type locality.

“La Save près d’Agram” [Sava river at Zagreb], Croatia.

##### Remarks.

Starobogatov et al. (1992: 65) considered the species as a junior synonym of *Microcolpia cornea* (Pfeiffer, 1828).

#### † Melanopsis (Melanosteira) pagodaeformis

Taxon classificationAnimaliaSorbeoconchaMelanopsidae

Pavlović, 1927

##### Original source.

Pavlović 1927: 62, pl. 7, fig. 11.

##### Type horizon.

Middle Pannonian, late Miocene.

##### Type locality.

“Из Карагача” [from Karagača near Vrčin], Serbia.

##### Types.

The illustrated syntype is stored in the Natural History Museum, Belgrade, coll. no. 213 (Milošević 1962: 23).

#### † Melanopsis pallaryi

Taxon classificationAnimaliaSorbeoconchaMelanopsidae

Wenz, 1919

##### Original source.

Wenz 1919a: 73.

##### Type horizon.

Early Pleistocene.

##### Type locality.

“Du puits Karoubi” (Pallary 1901a: 178) [from the well Karoubi], Algeria.

##### Remarks.

Replacement name for *Melanopsis acuminata* Pallary, 1901, non Gümbel, 1861.

#### Melanopsis doriaepallida

Taxon classificationAnimaliaSorbeoconchaMelanopsidae

Biggs, 1962

##### Original source.

Biggs 1962: 71.

##### Type locality.

“Bagh-i-Zerisf near Kerman”, Iran.

#### Melanopsis palmyrensis

Taxon classificationAnimaliaSorbeoconchaMelanopsidae

Pallary, 1939

##### Original source.

Pallary 1939: 96, pl. 6, figs 60–62.

##### Type locality.

“Palmyre”, Syria.

##### Remarks.

Heller et al. (2005: 248) considered the species as a junior synonym of *Melanopsis saulcyi* Bourguignat, 1853.

#### † Melanopsis panciciana

Taxon classificationAnimaliaSorbeoconchaMelanopsidae

Brusina, 1874

##### Original source.

Brusina 1874: 44.

##### Type horizon.

Langhian, middle Miocene.

##### Type locality.

“Ribarić”, Croatia.

##### Types.

The illustrated syntypes are stored in the Croatian Natural History Museum, Zagreb, coll. no. 3660-1300/1-2 (Milan et al. 1974: 95).

##### Remarks.

Neubauer et al. (2013: 135) considered this taxon as a junior synonym of *Melanopsis lyrata* Neumayr, 1869.

#### † Melanopsis neumayri var. papiolensis

Taxon classificationAnimaliaSorbeoconchaMelanopsidae

Almera & Bofill y Poch in Almera, 1894

##### Original source.

Almera 1894: 171, pl. 4, figs 5a–b.

##### Type horizon.

Late Messinian–early Zanclean, late Miocene–early Pliocene.

##### Type locality.

“Castellbisbal, Papiol”, Spain.

#### † Melanopsis papkesiensis

Taxon classificationAnimaliaSorbeoconchaMelanopsidae

Bandel, 2000

##### Original source.

Bandel 2000: 162, figs 52–85.

##### Type horizon.

Late Pannonian, late Miocene.

##### Type locality.

“Sand pit near Papkesi [Papkeszi]”, Hungary.

##### Types.

Geological-Palaeontological Institute and Museum University of Hamburg, coll. no. 4269.

#### † Melanoptychia paradoxa

Taxon classificationAnimaliaSorbeoconchaMelanopsidae

Brusina, 1892

##### Original source.

Brusina 1892: 144.

##### Type horizon.

Middle Pannonian, late Miocene.

##### Type locality.

“Markuševec”, Croatia.

##### Types.

The illustrated syntypes are stored in the Croatian Natural History Museum, Zagreb, coll. no. 2540-186/1-4 (Milan et al. 1974: 100).

#### Melanopsis pardalis

Taxon classificationAnimaliaSorbeoconchaMelanopsidae

Brot, 1878

##### Original source.

Brot 1874–1879: 373, pl. 38, fig. 5.

##### Type locality.

“Krain, im Gurkfluss” (Clessin 1890: 686) [in the river Gurk], Austria.

##### Remarks.

Brot (1878) introduced it as “*Melanopsis pardalis*. Mhlf. Mss.” in synonymy of *Hemisinus esperi* (Férussac, 1823) [currently in *Esperiana*]. Clessin (1890) made the name available by treating it as valid name (see Note 2).

#### † Melanopsis parkinsoni

Taxon classificationAnimaliaSorbeoconchaMelanopsidae

Deshayes, 1825

##### Original source.

Deshayes 1824–1837: 123, pl. 17, figs 3–4.

##### Type horizon.

Cuisian, late Ypresian, Eocene.

##### Type locality.

“Cuise-la-Mothe” [Cuise-la-Motte], France.

##### Remarks.

Wenz (1929: 2566) considered the species as a junior synonym of *Coptostylus albidus* (Lamarck, 1822) (Thiaridae).

#### Melania (Melanopsis) parreyssii

Taxon classificationAnimaliaSorbeoconchaMelanopsidae

Philippi, 1847

##### Original source.

Philippi 1847: 176, pl. 4, fig. 15.

##### Type horizon.

Late Pleistocene to Recent.

##### Type locality.

“Hungaria” [no locality indicated], Hungary? (at that time, the kingdom Hungary covered large parts of Romania and northern Croatia).

##### Remarks.

Philippi attributed the authority to Johann Georg Megerle von Mühlfeld, apparently based on an “in schedis” determination. The name “*parreyssi*” as mentioned in Brusina (1903), Kormos (1905) and Paucă (1937) is an incorrect subsequent spelling (see also Neubauer et al. 2014d).

#### † Melanopsis parva

Taxon classificationAnimaliaSorbeoconchaMelanopsidae

Pallary, 1916

##### Original source.

Pallary 1916: 78.

##### Type horizon.

Burdigalian, early Miocene (?).

##### Type locality.

“Dax”, France.

##### Remarks.

Pallary (1916, 1920) erroneously treated “*parva*” as available name attributed to Férussac (1823), who solely used the word as descriptive term (Latin “small”). The name became nevertheless available from Pallary (1916) who associated the name with an illustration in Férussac (1823: pl. 8, fig. 5). Cossmann and Peyrot (1918: 485) considered the taxon as a junior synonym of *Melanopsis subbuccinoides* d’Orbigny, 1852.

#### † Melanopsis olivula var. parvula

Taxon classificationAnimaliaSorbeoconchaMelanopsidae

Grateloup, 1838

##### Original source.

Grateloup 1838: 143, pl. 4, fig. 56.

##### Type horizon.

Burdigalian, early Miocene.

##### Type locality.

“Dax. [...] Mandillot”, France.

#### Melania parvula

Taxon classificationAnimaliaSorbeoconchaMelanopsidae

Brot, 1874

##### Original source.

Brot 1874–1879: 13, pl. 1, figs 2–2a.

##### Type locality.

“Krain” [Carniola, a historical region that comprised parts of present-day Slovenia; no locality indicated].

##### Remarks.

Appeared first as a nomen nudum in Brot (1862). The name was not found in Rossmässler (1839), to whom Brot (1862, 1874) referred.

#### Melanopsis jordanica var. parvula

Taxon classificationAnimaliaSorbeoconchaMelanopsidae

Bourguignat, 1884 [invalid]

##### Original source.

Bourguignat 1884: 142.

##### Type locality.

“Le Jourdain, à 4 kilomètres au-dessus de la Mer Morte; Ain-el-Mellaha, dans la plaine du Bahr-el-Houlé” [Jordan river, 4 km north of the Dead Sea; Aïn Mallahah, in the plains of the Hula valley], Israel.

##### Remarks.

Junior homonym of *Melanopsis olivula parvula* Grateloup, 1838 (see Note 1). Heller et al. (2005: 245) considered the variety a synonym of *Melanopsis lampra* Bourguignat, 1884.

#### † Melania patula

Taxon classificationAnimaliaSorbeoconchaMelanopsidae

Bellardi & Michelotti, 1841

##### Original source.

Bellardi and Michelotti 1841: 163, pl. 7, figs 8–9.

##### Type horizon.

Late Miocene.

##### Type locality.

“Del Tortonese” [from the region of Tortona], Italy.

##### Remarks.

Bellardi and Michelotti attributed the authority to Bonelli (1827), which is an unpublished collection catalogue. Considered to belong in the genus *Amphimelania* by Wenz (1929: 2876).

#### † Melanopsis (Lyrcaea) [sic] vindobonensispaucai

Taxon classificationAnimaliaSorbeoconchaMelanopsidae

Lubenescu, 1981

##### Original source.

Lubenescu 1981: 176, pl. 12, figs 8–12.

##### Type horizon.

Middle Pannonian, late Miocene.

##### Type locality.

“Comuna Vingard, 15 km NE de Sebeș-Alba”, Romania.

##### Types.

Institute of Geology and Geophysics, University of Bucharest; no number indicated.

#### † Melanopsis pauli

Taxon classificationAnimaliaSorbeoconchaMelanopsidae

Bandel & Riedel, 1994

##### Original source.

Bandel and Riedel 1994: 19, pl. 12, figs 2–6.

##### Type horizon.

Late Santonian–early Campanian, late Cretaceous.

##### Type locality.

“Csingertal, near Ajka (Bakony Mountains)”, Hungary.

##### Types.

Geological-Paleontological Department, Natural History Museum Vienna, Austria, coll. no. 1994/148.

#### † Melanopsis paulovici

Taxon classificationAnimaliaSorbeoconchaMelanopsidae

Bourguignat, 1880 

##### Original source.

Bourguignat 1880: 37.

##### Type horizon.

Langhian, middle Miocene.

##### Type locality.

“Vallée de la Cettina” [Cetina river valley], Croatia.

#### † Melanopsis pavlovici

Taxon classificationAnimaliaSorbeoconchaMelanopsidae

Brusina, 1902

##### Original source.

Brusina 1902: pl. 5, figs 45–47.

##### Type horizon.

Pannonian, zone D–E, late Miocene.

##### Type locality.

“Ripanj”, Serbia.

##### Types.

The illustrated syntypes are stored in the Croatian Natural History Museum, Zagreb, coll. no. 2495-141/1-3 (Milan et al. 1974: 95).

##### Remarks.

Appeared first as a nomen nudum in Brusina (1893). Not a homonym of *Melanopsis paulovici* [sic] as considered by Pallary (1916), who invalidly introduced *Melanopsis ripajensis* as replacement name. Note that Art. 58.4. (regarding the use of u or v for the same Latin letter) does not apply here, because the names are not based on Latin words but on the names of two different persons.

#### † Melania pecchiolii

Taxon classificationAnimaliaSorbeoconchaMelanopsidae

Hörnes, 1856

##### Original source.

Hörnes 1851–1856: 604, pl. 49, fig. 24.

##### Type horizon.

Badenian, middle Miocene.

##### Type locality.

“Forchtenau in Ungarn” [Forchtenstein, Burgenland], Austria.

##### Remarks.

Considered to belong in the genus *Amphimelania* by Wenz (1929: 2877). Note that Wenz gave the name as “*pecchioli*”, which is an incorrect subsequent spelling.

#### Melanopsis pechaudi

Taxon classificationAnimaliaSorbeoconchaMelanopsidae

Bourguignat, 1884

##### Original source.

Bourguignat 1884: 160.

##### Type locality.

“De la source de la Moulouiah, au nord de Lalla-Maghnia, près des frontières du Maroc” [in the source of the river Moulouya, north of Maghnia, near the border to Morocco], Morocco or Algeria.

##### Remarks.

Note that Bourguignat denoted the authority as “Bourguignat, 1882”.

#### † Melanopsis pedemontana

Taxon classificationAnimaliaSorbeoconchaMelanopsidae

Sacco, 1889

##### Original source.

Sacco 1889: 66, pl. 2, figs 1–8.

##### Type horizon.

Late Burdigalian, early Miocene.

##### Type locality.

“Colli torinesi, [...] presso Tetti Varetti” [Torino hills, near Tetti Varetti], Italy.

#### Melanopsis penchinati

Taxon classificationAnimaliaSorbeoconchaMelanopsidae

Bourguignat, 1868

##### Original source.

Bourguignat 1868: 432.

##### Type locality.

“Agora, en Aragon (Espagne)”, Spain.

##### Remarks.

The name “*pinchinati*” as mentioned in Brot (1879: 440) is an incorrect subsequent spelling.

#### † Melanopsis pentagona

Taxon classificationAnimaliaSorbeoconchaMelanopsidae

Brusina, 1892

##### Original source.

Brusina 1892: 138.

##### Type horizon.

Middle Pannonian, late Miocene.

##### Type locality.

“Markuševec”, Croatia.

##### Types.

The illustrated syntypes are stored in the Croatian Natural History Museum, Zagreb, coll. no. 2532-178/1-4 (Milan et al. 1974: 95).

#### Microcolpia peracuta

Taxon classificationAnimaliaSorbeoconchaMelanopsidae

Bourguignat, 1884

##### Original source.

Bourguignat 1884: 63.

##### Type locality.

“Environs de Krapina-Toeplitz (Croatie)” [surroundings of Krapinske toplice], Croatia.

##### Remarks.

Starobogatov et al. (1992: 65) considered the species as a junior synonym of *Microcolpia acicularis* (Férussac, 1823).

#### Melanopsis praemorsa f. perbrevis

Taxon classificationAnimaliaSorbeoconchaMelanopsidae

Pérès, 1946 [invalid]

##### Original source.

Pérès 1946: 113.

##### Type locality.

Not indicated.

##### Remarks.

First mentioned as nomen nudum in Pérès (1939), but described and made available by Pérès (1946). In both works Pérès obviously used the name not as a separate taxon but rather as descriptive term to fit existing species into his morphological concept. The name is in fact a junior objective synonym of several different species.

#### † Melanopsis percallosa

Taxon classificationAnimaliaSorbeoconchaMelanopsidae

Sandberger, 1875

##### Original source.

Sandberger 1870–1875: 574.

##### Type horizon.

Middle Miocene.

##### Type locality.

“Locle”, Switzerland.

##### Remarks.

Wenz (1929: 2763) considered the taxon as a junior synonym of *Melanopsis kleinii* Kurr, 1856.

#### † Melanopsis percarinata

Taxon classificationAnimaliaSorbeoconchaMelanopsidae

Förster, 1892

##### Original source.

Förster 1892: 225, pl.11, fig. 6.

##### Type horizon.

Early Rupelian, Oligocene.

##### Type locality.

“Kleinkems, [...] Tagolsheim”, Germany.

#### Melanopsis percarinata

Taxon classificationAnimaliaSorbeoconchaMelanopsidae

Pallary, 1918 [invalid]

##### Original source.

Pallary 1918: 150.

##### Type locality.

“Dans les séguias du Guers” [in the irrigation channel of Guers], Morocco.

##### Remarks.

Junior homonym of *Melanopsis percarinata* Förster, 1892.

#### † Melanopsis pergamena

Taxon classificationAnimaliaSorbeoconchaMelanopsidae

Calvert & Neumayr, 1880

##### Original source.

Calvert and Neumayr 1880: 375, pl. 2, figs 18–19.

##### Type horizon.

Late Sarmatian (sensu lato), Khersonian, late Miocene.

##### Type locality.

“Renkiöi” [north of İntepe], Turkey.

##### Remarks.

The name “*pergamenica*” as mentioned in Wenz (1929: 2804) is an incorrect subsequent spelling.

#### † Melanopsis permutabilis

Taxon classificationAnimaliaSorbeoconchaMelanopsidae

Pallary, 1920

##### Original source.

Pallary 1920b: 118.

##### Type horizon.

Cernikian, Pliocene.

##### Type locality.

“Bečić; Podvinje (Čaplja) [Čaplja trench near Slavonski Brod]; Sibinj; Kovačevac; Novska; Moslavina” (Brusina 1874: 41–42), Croatia.

##### Remarks.

Probably a junior synonym: introduced for *Melania costata* sensu Brusina, 1874, non Olivier, 1804, which has been considered to be the same species as *Melania costata* sensu Neumayr, 1869 (e.g., Neumayr and Paul 1875: 41). For that misidentified record, Brusina (1884a: 168) had already introduced *Melanopsis croatica* as new name (see also discussion of *Melanopsis pseudocostata* Oppenheim, 1890). Not included in the Fossilium Catalogus of Wenz (1929).

#### Melanopsis douttei var. perornata

Taxon classificationAnimaliaSorbeoconchaMelanopsidae

Pallary, 1920 

##### Original source.

Pallary 1920c: 150, pl. 4, fig. 3.

##### Type locality.

“Oued Fès, à 1.500 mètres en amont de la ville” [Oued Fes, at 1.500 m above the city], Morocco.

#### † Melanopsis petkovici

Taxon classificationAnimaliaSorbeoconchaMelanopsidae

Pavlović, 1931

##### Original source.

Pavlović 1931: 19, pl. 10, figs 19–20.

##### Type horizon.

Badenian, middle Miocene.

##### Type locality.

“Из села Врмџе” [Vrmdža], Serbia.

##### Types.

The illustrated syntype is stored in the Natural History Museum, Belgrade, coll. no. 2888 (Milošević 1962: 24).

##### Remarks.

Appeared first as a nomen nudum in Pavlović (1922: 49).

#### † Melanopsis petrovici

Taxon classificationAnimaliaSorbeoconchaMelanopsidae

Brusina, 1893

##### Original source.

Brusina 1893: 58 (only described in Italian part).

##### Type horizon.

Transdanubian, Pannonian, late Miocene.

##### Type locality.

“Orešac”, Serbia.

##### Types.

Lectotype designation by Milan et al. (1974: 84) invalid: it is uncertain whether the specimen illustrated by Brusina (1902) was part of the original type series. The specimen is stored in the Croatian Natural History Museum, Zagreb, coll. no. 2484-130.

#### Fagotia pfeifferi

Taxon classificationAnimaliaSorbeoconchaMelanopsidae

Bourguignat, 1884

##### Original source.

Bourguignat 1884: 36.

##### Type locality.

“Rivières de Carniole; la Save à Steinbrück” [rivers in Carniola, a historical region that comprised parts of present-day Slovenia; in the Sava river near Zidani Most], Slovenia.

##### Remarks.

Note that Bourguignat denoted the authority as “Bourguignat, 1880”. Starobogatov et al. (1992: 60) considered the species as a junior synonym of *Fagotia* [= *Esperiana*] *acroxia* Bourguignat, 1884.

#### † Melanopsis phanesiana

Taxon classificationAnimaliaSorbeoconchaMelanopsidae

Bukowski, 1892

##### Original source.

Bukowski 1892: 249.

##### Type horizon.

Salakos Formation, Pliocene.

##### Type locality.

“Rhodos” (locality specified as “Kalavarda” in Bukowski 1893), Greece.

##### Remarks.

Willmann (1981: 127) considered this taxon as a synonym of *Melanopsis vandeveldi* Bukowski, 1892.

#### Melanopsis phoeniciaca

Taxon classificationAnimaliaSorbeoconchaMelanopsidae

Bourguignat, 1884

##### Original source.

Bourguignat 1884: 133.

##### Type locality.

“Dans le Bélus [près de Saint-Jean-d’Acre, en Syrie]” [in the Na’aman river, near Acre], Israel.

##### Remarks.

The spelling “*phaeniciaca*” on p. 76 is obviously a typesetting mistake regarding the ligature (æ instead of œ). Heller et al. (2002: 596) considered the species (misspelt as “*phoeneciaca*”) a junior synonym of *Melanopsis costata* (Olivier, 1804). Heller et al. (2005: 244) treated it as a junior synonym of *Melanopsis lampra* Bourguignat, 1884.

#### † “ Melanopsis pichleri

Taxon classificationAnimaliaSorbeoconchaMelanopsidae

” mentioned in (Pichler 1856: 735–736)

##### Horizon.

Late Cretaceous.

##### Locality.

“Auf zwei Puncten der Brandenberger Ache. Ungefähr ¾ Stunde nördlich von Binneck [...]. Etwa eine Viertelstunde unter Binneck [...] am linken Ufer der Ache” [At two points along the Brandenberger Ache: about three quarters of an hour north of Pinegg and a quarter of an hour south of Pinegg in Tyrol], Austria.

##### Remarks.

First mentioned as nomen nudum in Pichler (1856), who attributed the authority to Hörnes. It was validly described by Stoliczka (1860: 487, pl. 1, figs 6–9), but not considered a Melanopsidae there or later (see, e.g., Yen 1958: 206).

#### † Melanopsis pictus

Taxon classificationAnimaliaSorbeoconchaMelanopsidae

Hörnes, 1856

##### Original source.

Hörnes 1851–1856: 600, pl. 49, fig. 14.

##### Type horizon.

Badenian, middle Miocene.

##### Type locality.

“Grund”, Austria.

##### Remarks.

After Wenz (1929: 2505) this species belongs in the genus *Semisinus* P. Fischer, 1885, which is an unjustified emendation of *Hemisinus* Swainson, 1840 (Thiaridae).

#### Fagotia pilariana

Taxon classificationAnimaliaSorbeoconchaMelanopsidae

Bourguignat, 1884

##### Original source.

Bourguignat 1884: 46.

##### Type locality.

“Dans la Save à Agram et à Sissek. Dans la rivière, entre Plaski et Ostaria (Croatie)” [Sava river at Zagreb and Sisak. In the river between Plaški and Oštarije], Croatia.

##### Remarks.

Note that Bourguignat denoted the authority as “Bourguignat, 1882”. Starobogatov et al. (1992: 60) considered the species as a junior synonym of *Fagotia* [= *Esperiana*] *servainiana* Bourguignat, 1884.

#### Melanella pilariana

Taxon classificationAnimaliaSorbeoconchaMelanopsidae

Bourguignat, 1884

##### Original source.

Bourguignat 1884: 15.

##### Type locality.

“En Croatie, au pont de la Save à Agram, ainsi qu’à Steinbrück; [...] en Bosnie, dans les rivières de Zenica et de la Migliaska à Sérajewo” [in Croatia, at the bridge of the Sava river in Zagreb, as well as Zidani Most (Slovenia); in Bosnia, in the rivers Zenica and Miljacka near Sarajevo].

##### Remarks.

Appeared first as a nomen nudum in Servain (1884: 379). Note that Bourguignat denoted the authority as “Bourguignat, 1880”.

#### † Melanopsis planoidea

Taxon classificationAnimaliaSorbeoconchaMelanopsidae

Aldrich, 1895

##### Original source.

Aldrich 1895: 15, pl. 2, fig. 7.

##### Type horizon.

Early Eocene.

##### Type locality.

“Gregg’s Landing, Alabama”, United States.

##### Types.

Harris (1899: 77) designated a lectotype, which is stored in the collection of the National Museum of Natural History, Smithsonian Institution, Washington, DC, coll. no. 638788 (Palmer and Brann 1966: 755).

##### Remarks.

Palmer and Brann (1966: 755) considered the taxon as a junior synonym of *Melanopsis anita* Aldrich, 1886. According to Allmon (1990: 63), neither of both species belongs to the Melanopsidae.

#### † Melanopsis doumerguei plena

Taxon classificationAnimaliaSorbeoconchaMelanopsidae

Wenz, 1928

##### Original source.

Wenz 1928b: 219.

##### Type horizon.

Late Miocene.

##### Type locality.

“Smendou” [Zighoud Youcef], Algeria.

##### Remarks.

Replacement name for *Melanopsis doumergueiobesa* Pallary, 1901, non Gassies, 1856 (see Note 1).

#### † Fagotia (Sasykiana) plena

Taxon classificationAnimaliaSorbeoconchaMelanopsidae

Gozhik in Gozhik & Datsenko, 2007

##### Original source.

Gozhik and Datsenko 2007: 98, pl. 89, figs 2–3.

##### Type horizon.

Pliocene.

##### Type locality.

“Oз. Сасык” [Lake Sasyk], Ukraine.

##### Types.

Institute of Geological Sciences of the National Academy of Sciences of Ukraine, coll. no. 4603.

#### † Melanoptychia pleuroplagia

Taxon classificationAnimaliaSorbeoconchaMelanopsidae

Bourguignat, 1880 

##### Original source.

Bourguignat 1880: 32.

##### Type horizon.

Langhian, middle Miocene.

##### Type locality.

“Vallée de la Cettina” [Cetina river valley], Croatia.

#### Melanopsis pleuroplagia

Taxon classificationAnimaliaSorbeoconchaMelanopsidae

Bourguignat, 1884 [invalid]

##### Original source.

Bourguignat 1884: 155.

##### Type locality.

“Dans le Viar et le Carbanès, entre Cordoue et Séville (Espagne)” [in the Río Viar and Río Corbones, between Córdoba and Sevilla], Spain.

##### Remarks.

Junior secondary homonym of *Melanoptychia pleuroplagia* Bourguignat, 1880. It was considered as a junior synonym of *Melanopsis sevillensis* by Pallary (1916: 86).

#### Melanopsis pleurotomoidaea

Taxon classificationAnimaliaSorbeoconchaMelanopsidae

Bourguignat, 1884

##### Original source.

Bourguignat 1884: 105.

##### Type locality.

“Ruisseaux de l’île d’Ivice, dans les Baléares” [stream on the island of Ibiza], Spain.

#### † Melanopsis (Melanoptychia) pleurotomoides

Taxon classificationAnimaliaSorbeoconchaMelanopsidae

Pavlović, 1927

##### Original source.

Pavlović 1927: 60, pl. 7, figs 5–6.

##### Type horizon.

Pannonian, zone D–E, late Miocene.

##### Type locality.

“У засеоку Рамаћи [...] у Рипњу” [from the village Ramača and from Ripanj], Serbia.

##### Types.

The illustrated syntype is stored in the Natural History Museum, Belgrade, coll. no. 794 (Milošević 1962: 24).

#### Melanopsis barbini var. plicata

Taxon classificationAnimaliaSorbeoconchaMelanopsidae

Pallary, 1911

##### Original source.

Pallary 1911: 130, [unnumbered plate], fig. 6.

##### Type locality.

“Tout près d’Oudjda, à 4 kilom. S.-E., sourdent les belles sources de Sidi-Yahia qui alimentent une véritable oasis, puis la ville d’Oudjda, et vont finalement se déverser dans l’oued Isly” [near Oujda, 4 km southeast, at the sources of Sidi Yahya that feed an oasis and the city of Oujda, and ultimately will flow into the Oued Isly], Morocco.

#### Melanopsis subimpressa var. plicata

Taxon classificationAnimaliaSorbeoconchaMelanopsidae

Pallary, 1928 [invalid] 

##### Original source.

Pallary 1928a: 263.

##### Type locality.

“Dans une source tiède à proximité de la Moulouïa” [in a warm source close to the river Moulouya], Morocco.

##### Remarks.

Junior homonym of *Melanopsis plicata* Pallary, 1911.

#### Melanopsis (Mesopotamia) khabourensis var. plicata

Taxon classificationAnimaliaSorbeoconchaMelanopsidae

Pallary, 1939 [invalid]

##### Original source.

Pallary 1939: 103, pl. 5, fig. 19.

##### Type locality.

“Ras el ‘Ain du Khabour” [Chabur river near Ra’s al ‘Ayn], Syria.

##### Remarks.

Junior homonym of *Melanopsis barbini plicata* Pallary, 1911 (see Note 1). Heller et al. (2005: 254) considered the variety as a junior synonym of *Melanopsis infracincta* Martens, 1874.

#### † Melanopsis plicatella

Taxon classificationAnimaliaSorbeoconchaMelanopsidae

Neumayr, 1880

##### Original source.

Neumayr 1880c: 477, pl. 7, fig. 2.

##### Type horizon.

Pannonian, late Miocene; late Burdigalian to early Langhian, early–middle Miocene.

##### Type locality.

“Posušje; [...] Seonica”, Bosnia and Herzegovina.

#### † Melanopsis inconstans var. plicatula

Taxon classificationAnimaliaSorbeoconchaMelanopsidae

Brusina, 1874

##### Original source.

Brusina 1874: 39.

##### Type horizon.

Langhian, middle Miocene.

##### Type locality.

“Miočić”, Croatia.

##### Types.

Milan et al. (1974: 96) indicated a holotype, but it is uncertain whether the specimen is part of the original type series and whether it was the only one Brusina had at hand (holotype by monotypy, Art. 73.1.2). The specimen is stored in the Croatian Natural History Museum, Zagreb, coll. no. 2976-622/1-2.

##### Remarks.

Neubauer et al. (2016b: 23) considered this taxon as a junior synonym of *Melanopsis inconstans* Neumayr, 1869.

#### † “ Melanopsis themaki var. plicatula

Taxon classificationAnimaliaSorbeoconchaMelanopsidae

” mentioned in Brusina (1903: 110) [unavailable]

##### Horizon.

Late Pleistocene–early Holocene.

##### Locality.

“Bischofsbad” [Püspökfürdő, Băile 1 Mai, Lake Pețea], Romania.

##### Remarks.

Nomen nudum. If available, it would be a junior objective synonym of *Melanopsis themaki*, because Brusina (1903) indicated it as the typical form of the species, as well as a junior homonym of *Melanopsis inconstansplicatula* Brusina, 1874 (see Note 1). Neubauer et al. (2014d: 125) considered this taxon as a junior synonym of *Microcolpia parreyssii sikorai* (Brusina, 1903).

#### † Melanopsis vidovici var. plicatula

Taxon classificationAnimaliaSorbeoconchaMelanopsidae

Brusina, 1903 [invalid]

##### Original source.

Brusina 1903: 113.

##### Type horizon.

Late Pleistocene–early Holocene.

##### Type locality.

“Bischofsbad” [Püspökfürdő, Băile 1 Mai, Lake Pețea], Romania.

##### Remarks.

Junior homonym of *Melanopsis inconstans plicatula* Brusina, 1874 (see Note 1). Neubauer et al. (2014d: 125) considered this taxon as a junior synonym of *Microcolpia parreyssii sikorai* (Brusina, 1903).

#### † Melanopsis (Canthidomus) plicatula

Taxon classificationAnimaliaSorbeoconchaMelanopsidae

Handmann, 1887 [invalid]

##### Original source.

Handmann 1887: 30, pl. 7, figs 1–3.

##### Type horizon.

Pannonian, zone B–D, late Miocene.

##### Type locality.

“Leobersdorf”, Austria.

##### Remarks.

Originally the gender was indicated as masculine (“*plicatulus*”), but *Melanopsis* is feminine, which is why the name must be “*plicatula*”. Junior homonym of *Melanopsis inconstans plicatula* Brusina, 1874 (see Note 1). Pallary (1916: 84) introduced *Melanopsis similis* as replacement name. Wenz (1929: 2674) listed both names in synonymy of *Melanopsis bouei* Férussac, 1823.

#### † Melanopsis polita

Taxon classificationAnimaliaSorbeoconchaMelanopsidae

Pallary, 1916

##### Original source.

Pallary 1916: 79.

##### Type horizon.

Sparnacian, early Ypresian, Eocene.

##### Type locality.

“Pourcy” (Cossmann 1909: captions of pl. 3), France.

##### Remarks.

Introduced for *Melania buccinoidea* sensu Cossmann, 1909 (p. 171, pl. 3, figs 21–22) and Cossmann, 1910 (in Cossmann and Pissarro 1906–1913: pl. 19, fig. 118–1). Wenz (1929: 2662) considered it as a junior synonym of *Melanopsis antidiluviana*.

#### Melanopsis vanrossomi var. polita

Taxon classificationAnimaliaSorbeoconchaMelanopsidae

Pallary, 1936 [invalid]

##### Original source.

Pallary 1936: 59.

##### Type locality.

“Tanalt”, Morocco.

##### Remarks.

Junior homonym of *Melanopsis polita* Pallary, 1916.

#### † Melanopsis polyptycha

Taxon classificationAnimaliaSorbeoconchaMelanopsidae

Neumayr, 1880 

##### Original source.

Neumayr 1880b: 294, pl. 1, fig. 26.

##### Type horizon.

Plio-Pleistocene.

##### Type locality.

“Zwischen Pylle und Antimachia” [between Pýli and Antimácheia, Kos Island], Greece.

##### Remarks.

The name “*polyptychia*” as mentioned in Wenz (1929: 2806) is an incorrect subsequent spelling.

#### † Ancillaria (Amalda?) pomahaka

Taxon classificationAnimaliaSorbeoconchaMelanopsidae

Hutton, 1873

##### Original source.

Hutton 1873: 6.

##### Type horizon.

Pomahaka Formation, Duntroonian (? = late Oligocene).

##### Type locality.

“Pomahaka, Otago” [Pomahaka River], New Zealand.

##### Remarks.

The species was attributed to the *Melanopsis* (*Stilospirula*?) by Beu and Maxwell (1990: 141).

#### Melanella ponderosa

Taxon classificationAnimaliaSorbeoconchaMelanopsidae

Bourguignat, 1884

##### Original source.

Bourguignat 1884: 23.

##### Type locality.

“En Carmiole [sic], en Croatie, et en Dalmatie, notamment dans la Cettina” [in Carniola (a historical region that comprised parts of present-day Slovenia), Croatia and Dalmiata, especially in the Cetina river].

##### Remarks.

Appeared first as a nomen nudum in Villa and Villa (1841: 36) and Brusina (1867: 86). Note that Bourguignat denoted the authority as “Bourguignat, 1877”.

#### † Melanopsis (Mingreliciana) pontica

Taxon classificationAnimaliaSorbeoconchaMelanopsidae

Gozhik in Gozhik & Datsenko, 2007

##### Original source.

Gozhik and Datsenko 2007: 92, pl. 96, figs 2–3.

##### Type horizon.

Middle Pontian (Dacian Basin), late Miocene.

##### Type locality.

“Вулканешты” [Vulcăneşti], Moldova.

##### Types.

Institute of Geological Sciences of the National Academy of Sciences of Ukraine, coll. no. 3190.

#### † Melanopsis porumbari

Taxon classificationAnimaliaSorbeoconchaMelanopsidae

Porumbaru, 1881

##### Original source.

Porumbaru 1881: 29, pl. 7, fig. 5, pl. 9, fig. 3.

##### Type horizon.

Early–middle Romanian, Pliocene.

##### Type locality.

“Cretzesci et Podari” [Crețești and Podari], Romania.

##### Remarks.

Porumbaru attributed the authority to Brusina, but there is not clear evidence that the description really derived from that author. The name “*porumbarui*” as mentioned in Fontannes (1887: 336), Stefanescu (1896: 134) and Wenz (1929: 2806) is an incorrect subsequent spelling.

#### † Melanopsis impressaposterior

Taxon classificationAnimaliaSorbeoconchaMelanopsidae

Papp, 1953

##### Original source.

Papp 1953b: 133, pl. 9, figs 19–23.

##### Type horizon.

Pannonian, zone B, late Miocene.

##### Type locality.

“Leobersdorf Sandgrube” [Leobersdorf sand pit], Austria.

##### Remarks.

Appeared first as nomen nudum in Papp (1951: 107, 108, 124, 127). Although he mentioned differences from other melanopsids, he did not state any morphological characters.

#### † Melanopsis eleisposterior

Taxon classificationAnimaliaSorbeoconchaMelanopsidae

Schütt in Symeonidis et al., 1986 [invalid]

##### Original source.

Symeonidis et al. 1986: 342, pl. 4, figs 8–9.

##### Type horizon.

Gelasian, early Pleistocene.

##### Type locality.

“Weganrisse und Regenrisse 3 Km N Antirrion in Akarnanien” [outcrops 3 km north of Antirrio], Greece.

##### Types.

Senckenberg Forschungsinstitut und Naturmuseum Frankfurt; no number indicated.

##### Remarks.

Junior homonym of *Melanopsisimpressaposterior* Papp, 1953 (see Note 1). Neubauer et al. (2014a) introduced *Melanopsis gearyae* as replacement name.

#### Melanopsis potamactebia

Taxon classificationAnimaliaSorbeoconchaMelanopsidae

Bourguignat, 1870

##### Original source.

Bourguignat 1870: 67.

##### Type locality.

“Dans le Danube. [...] non-seulement de Brahilov, mais encore des environs de Belgrade” [in the Danube; not only from Brăila (Romania), but also from the surroundings of Belgrade (Serbia)].

#### † Melanopsis (Coptostylus) pourcyensis

Taxon classificationAnimaliaSorbeoconchaMelanopsidae

Cossmann, 1907

##### Original source.

Cossmann 1907: 236, pl. 7, fig. 118–12.

##### Type horizon.

Sparnacian, early Ypresian, Eocene.

##### Type locality.

“Pourcy”, France.

##### Remarks.

*Coptostylus* is at present considered to belong in the family Thiaridae (Pacaud and Le Renard 1995: 156).

#### Microcolpia praeclara

Taxon classificationAnimaliaSorbeoconchaMelanopsidae

Bourguignat, 1884 

##### Original source.

Bourguignat 1884: 54.

##### Type locality.

“La Save à Agram, à Sissek et près de Belgrade” [Sava river at Zagreb, at Sisak (Croatia) and near Belgrade (Serbia)].

##### Remarks.

Starobogatov et al. (1992: 65) considered the species as a junior synonym of *Microcolpia cornea* (Pfeiffer, 1828).

#### † Melanopsis praecursor

Taxon classificationAnimaliaSorbeoconchaMelanopsidae

Schütt in Schütt & Ortal, 1993

##### Original source.

Schütt and Ortal 1993: 93, pl. 2, fig. 30.

##### Type horizon.

Early Pleistocene.

##### Type locality.

“The type-locality of the Upper Pliocene sediments or the ‘Erq el-Ahmar Formation in the Jordan Valley south of the Sea of Galilee” [i.e. ‘Erq el-Ahmar (also known as Gesher)], Israel.

##### Types.

Paleontology Collection of the Hebrew University of Jerusalem; no number indicated.

#### Buccinum praemorsum

Taxon classificationAnimaliaSorbeoconchaMelanopsidae

Linnaeus, 1758

##### Original source.

Linnaeus 1758: 740.

##### Type locality.

“In Europa australiore” [southern Europe].

##### Remarks.

Sometimes incorrectly listed as the type species of *Melanopsis* (e.g., Wenz 1929: 2647; Heller et al. 1999: 56; Esu and Girotti 2015b: 150).

#### Buccinum praerosum

Taxon classificationAnimaliaSorbeoconchaMelanopsidae

Linnaeus, 1767 [invalid]

##### Original source.

Linnaeus 1767: 1203.

##### Remarks.

The name *Buccinum praerosum*, commonly combined as *Melanopsis praerosa*, is an unjustified emendation and therefore a junior objective synonym of *Buccinum praemorsum* Linnaeus, 1758.

#### † “ Melanopsis pregorceisci

Taxon classificationAnimaliaSorbeoconchaMelanopsidae

” mentioned in Gillet et al. (1978: 58) [unavailable]

##### Horizon.

Khersonian, late Sarmatian, late Miocene.

##### Locality.

“Hauptstraße bei Avcilar, nahe der Abzweigung nach Ambarliköy” [main road at Avcılar, near the diversion to Ambarlı, west of Istanbul], Turkey.

##### Remarks.

Nomen nudum; mentioned as “new species” in Gillet et al. (1978) but left undescribed.

#### Melanopsis princeps

Taxon classificationAnimaliaSorbeoconchaMelanopsidae

Lea, 1837

##### Original source.

Lea 1837: 82, pl. 19, fig. 74.

##### Type locality.

“Cape of Good Hope”, South Africa.

##### Remarks.

Type species of *Faunopsis* Gill, 1863. It was considered as a junior synonym of *Faunus ater* (Linnaeus, 1758), the type species of the genus *Faunus* Montfort, 1810 (Pachychilidae), by Starmühlner (1974: 132).

#### † Melanopsis (Canthidomus) prionodonta

Taxon classificationAnimaliaSorbeoconchaMelanopsidae

Handmann, 1887

##### Original source.

Handmann 1887: 33, pl. 7, fig. 15.

##### Type horizon.

Pannonian, zone B–D, late Miocene.

##### Type locality.

“Leobersdorf”, Austria.

##### Remarks.

Wenz (1929: 2675) considered the taxon as a junior synonym of *Melanopsis bouei* Férussac, 1823.

#### † Melanopsis proboscidea

Taxon classificationAnimaliaSorbeoconchaMelanopsidae

Deshayes, 1862

##### Original source.

Deshayes 1861–1864: 471, pl. 31, figs 18–24.

##### Type horizon.

Bartonian, Eocene.

##### Type locality.

“Chery-Chartreuve”, France.

#### † Melanopsis (Martinia) martiniana var. proclivis

Taxon classificationAnimaliaSorbeoconchaMelanopsidae

Handmann, 1887

##### Original source.

Handmann 1887: 25, pl. 4, figs 1–2.

##### Type horizon.

Pannonian, zone B–D, late Miocene.

##### Type locality.

“Leobersdorf”, Austria.

##### Remarks.

Papp (1953b: 134) considered the taxon as a junior synonym of *Melanopsis fossilis* (Gmelin, 1791).

#### Melanopsis chehirensis var. producta

Taxon classificationAnimaliaSorbeoconchaMelanopsidae

Pallary, 1939

##### Original source.

Pallary 1939: 95, pl. 6, figs 55, 75.

##### Type locality.

“Yeni Chehir” [Yenişehir], Turkey.

#### † Melania (Balanocochlis) propatula

Taxon classificationAnimaliaSorbeoconchaMelanopsidae

Sacco, 1886

##### Original source.

Sacco 1886: 451, pl. 1, fig. 10.

##### Type horizon.

Late Burdigalian, early Miocene.

##### Type locality.

“Collina di Torino” [Torino hills], Italy.

##### Remarks.

Considered to belong in the genus *Amphimelania* by Wenz (1929: 2876).

#### Melanopsis prophetarum

Taxon classificationAnimaliaSorbeoconchaMelanopsidae

Bourguignat in Locard, 1883

##### Original source.

Locard 1883a: 265, pl. 23, figs 52–55.

##### Type locality.

“Dans le eaux de la fontaine de l’Élysée, à Jéricho; [...] des environs de Beyrouth; [...] dans les eaux du lac d’Antioche” [from the spring of Elysium (?) at Jericho (Palestine); from the surroundings of Beirut (Lebanon); from the waters of Lake Anuk (also as Amik) (Turkey)].

#### † Melanopsis (Martinia) martiniana var. propinqua

Taxon classificationAnimaliaSorbeoconchaMelanopsidae

Handmann, 1887

##### Original source.

Handmann 1887: 25, pl. 4, figs 3–4.

##### Type horizon.

Pannonian, zone B–D, late Miocene.

##### Type locality.

“Leobersdorf”, Austria.

##### Remarks.

Wenz (1929: 2719) considered this taxon as a junior synonym of *Melanopsis fossilis* (Gmelin, 1791).

#### † Melanopsis proteus

Taxon classificationAnimaliaSorbeoconchaMelanopsidae

Tournouër, 1875

##### Original source.

Tournouër 1875: 77.

##### Type horizon.

Tafi Formation, early Pleistocene.

##### Type locality.

“Prope vicum Antimaki” [near the village Antimácheia, Kos Island], Greece.

##### Remarks.

The species epithet is a noun in apposition (named after the Greek God Proteus) and needs not to agree in gender with the generic name (Art. 31.2.1).

#### † Melanopsis protopygmaea

Taxon classificationAnimaliaSorbeoconchaMelanopsidae

Halaváts, 1914

##### Original source.

Halaváts 1914: 422, six unnumbered textfigures.

##### Type horizon.

Sarmatian (sensu stricto), middle Miocene.

##### Type locality.

“Graben unterhalb der Gemeinde-Baumschule östlich von Oltszakadát” [trench below communal tree nursery, east of Săcădate], Romania.

#### † Fagotia prysjazhnjuki

Taxon classificationAnimaliaSorbeoconchaMelanopsidae

Gozhik, 2002

##### Original source.

Gozhik 2002: 54, pl. 3, figs 15, 17.

##### Type horizon.

Middle Pontian (Dacian Basin), late Miocene.

##### Type locality.

“Виноградівка” [Vynohradivka, Odes’ka Oblast’], Ukraine.

##### Types.

Institute of Geological Sciences of the National Academy of Sciences of Ukraine, coll. no. 3186.

#### † Melanopsis pseudoaustriaca

Taxon classificationAnimaliaSorbeoconchaMelanopsidae

Sauerzopf, 1952

##### Original source.

Sauerzopf 1952: 13, pl. 2, fig. 5.

##### Type horizon.

Pannonian, zone D, late Miocene.

##### Type locality.

“Burgau”, Austria.

##### Remarks.

The name “*pseudaustriaca*” as mentioned in Lueger (1980: 117) is an incorrect subsequent spelling.

#### † Melanopsis pseudobesa

Taxon classificationAnimaliaSorbeoconchaMelanopsidae

Bandel, 2000

##### Original source.

Bandel 2000: 155, figs 33–36.

##### Type horizon.

Late Pannonian, late Miocene.

##### Type locality.

“Papkesi [Papkeszi] near the eastern shore of Lake Balaton”, Hungary.

##### Types.

Geological-Palaeontological Institute and Museum University of Hamburg, coll. no. 4266.

#### † Melanopsis pseudocostata

Taxon classificationAnimaliaSorbeoconchaMelanopsidae

Oppenheim, 1890

##### Original source.

Oppenheim 1890b: 591.

##### Type horizon.

Cernikian, Pliocene.

##### Type locality.

“Cigelnik; [...] Graben zwischen Čapla und der Podwiner Kirche; [...] Thal hinter der Podwiner Kirche; [...] Sibin; [...] Repušnica” (Neumayr and Paul 1875: 41) [Ciglenik; trench between Čaplja and the church of Podvinje; valley behind the church of Podvinje; Sibinj; Repušnica], Croatia.

##### Remarks.

Different concepts of this species were applied in the literature and created considerable confusion. Oppenheim introduced the name clearly for the misidentified record of *Melania costata* sensu Neumayr in Neumayr & Paul, 1875 (p. 41), non Olivier, 1804 from Slavonia, despite referring about Greek material. Later, however, he considered the name as rectification also for *Melania costata* sensu Fuchs, 1877 from Megara and sensu Tournouër, 1876 from Kos Island (Oppenheim 1891: 465). This fact remained unnoticed by Wenz (1929: 2808), who treated *Melanopsis pseudocostata* as replacement name of the latter two records (Fuchs, Tournouër) but not of Neumayr and Paul’s species.

In addition, there are three other new names proposed for misidentified *Melania costata* from the Pliocene of Slavonia, i.e., *Melanopsis croatica* Brusina, 1884 (for *Melania costata* sensu Neumayr, 1869), *Melanopsis cosmanni* Pallary, 1916 [= *cossmanni*, just. emend.] (for *Melania costata* sensu Cossmann, 1909) and *Melanopsis permutabilis* Pallary, 1920 (for *Melania costata* sensu Brusina, 1874). Note that Wenz (1929) erroneously considered *Melanopsis cosmanni* a replacement name of Neumayr’s (1869) record.

Wenz obviously considered all erroneous “*Melania costata*” records from Slavonia to represent the same species and synonymized them with *Melanopsis cosmanni*, but was unaware that *Melanopsis croatica* Brusina, 1884 is the first available name for them. Although the four names (*croatica*, *pseudocostata*, *cosmanni*, *permutabilis*) likely refer to the very same species they are based on different specimens and thus no objective synonyms.

For the record by Tournouër (1876) from Kos Island, Pallary (1929: 119) introduced *Melanopsis sculptilis* as replacement name. The misidentified *Melanopsis costata* sensu Fuchs, 1877 from Megara, however, is still nameless.

The name “*pseudodecorata*” mentioned by Cossmann (1909: 178) is an incorrect subsequent spelling.

#### † Melanopsis praemorsa var. pseudofallax

Taxon classificationAnimaliaSorbeoconchaMelanopsidae

Sacco, 1895

##### Original source.

Sacco 1895: 9, pl. 1, fig. 14.

##### Type horizon.

Early Messinian, late Miocene.

##### Type locality.

“S. Marzano Oliveto”, Italy.

##### Remarks.

Wenz (1929: 2733) considered the taxon as a junior synonym of *Melanopsis fusulatina* Sacco, 1895.

#### Melanopsis pseudoferussaci

Taxon classificationAnimaliaSorbeoconchaMelanopsidae

Pallary, 1899

##### Original source.

Pallary 1899: 139, pl. 9, figs 11–12.

##### Type locality.

“Environs de Tétouan” [surrondings of Tétouan], Morocco.

#### † Melanopsis fossilispseudoimpressa

Taxon classificationAnimaliaSorbeoconchaMelanopsidae

Papp, 1953

##### Original source.

Papp 1953b: 135, pl. 11, figs 5–8.

##### Type horizon.

Pannonian, zone E, late Miocene.

##### Type locality.

“Vösendorf (Sandriff)” [Vösendorf (sandbar)], Austria.

##### Remarks.

Appeared first as a nomen nudum in Papp (1951: 115, 130 [as species], 147–148, 150 [as subspecies]).

#### † Melanopsis impressapseudonarzolina

Taxon classificationAnimaliaSorbeoconchaMelanopsidae

Papp, 1953

##### Original source.

Papp 1953b: 132, pl. 9, figs 14–18.

##### Type horizon.

Pannonian, zone B, late Miocene.

##### Type locality.

“Leobersdorf Sandgrube” [Leobersdorf sand pit], Austria.

##### Remarks.

Appeared first as nomen nudum in Papp (1951: 107, 123, 124, 127).

#### † Melanopsis pseudopigmea

Taxon classificationAnimaliaSorbeoconchaMelanopsidae

Magrograssi, 1928

##### Original source.

Magrograssi 1928: 260, pl. 6, fig. 21.

##### Type horizon.

Plio-Pleistocene.

##### Type locality.

“Coo: vicino a Cardamena” [Kos island: close to Kardámaina], Greece.

#### † Melanopsis pseudopraerosa

Taxon classificationAnimaliaSorbeoconchaMelanopsidae

Sacco, 1889

##### Original source.

Sacco 1889: 67, pl. 2, fig. 14.

##### Type horizon.

Late Burdigalian, early Miocene.

##### Type locality.

“Colli torinesi” [Torino hills], Italy.

#### † Melanopsis pseudopygmaea

Taxon classificationAnimaliaSorbeoconchaMelanopsidae

Jekelius, 1944

##### Original source.

Jekelius 1944: 131, pl. 49, figs 11–17.

##### Type horizon.

Early Pannonian, late Miocene.

##### Type locality.

“Turislav-Tal bei Soceni” [Turislav valley near Soceni], Romania.

#### † Melanopsis pseudoscalaria

Taxon classificationAnimaliaSorbeoconchaMelanopsidae

Sandberger, 1886

##### Original source.

Sandberger 1886: 119.

##### Type horizon.

*Oncophora* Beds, middle Burdigalian, early Miocene.

##### Type locality.

Rather unprecisely given as “Kirchberger Schichten Mährens” [Kirchberg Formation in Moravia] by Sandberger (1886). Later specified as “Oslawan” [Oslavany] by Rzehak (1893: 174), Czech Republic.

#### † Melanopsis pseudosubulata

Taxon classificationAnimaliaSorbeoconchaMelanopsidae

Newton, 1891

##### Original source.

Newton 1891: 203.

##### Type horizon.

Bartonian?–Rupelian, late Eocene–early Oligocene.

##### Type locality.

“Hempstead [...]. Headon Hill; Hordwell. [...] High Cliff”, United Kingdom.

##### Remarks.

Introduced (as “*pseudo-subulata*”) for a part of Sowerby’s material for *Melanopsis fusiformis* Sowerby, 1822 (Sowerby 1821–1823: pl. 332, figs 6–7) as well as for misidentified *Melanopsis subulata* sensu Forbes, 1856, non Sowerby, 1822.

#### † Melanopsis pterochila

Taxon classificationAnimaliaSorbeoconchaMelanopsidae

Brusina, 1874

##### Original source.

Brusina 1874: 30, pl. 1, figs 5–6.

##### Type horizon.

Cernikian, Pliocene.

##### Type locality.

“Podvinje (Čaplja) [Čaplja trench near Slavonski Brod]; Kovačevac”, Croatia.

##### Types.

Milan et al. (1974: 96) indicated a holotype, but it is uncertain whether the specimen was the only one Brusina had at hand (holotype by monotypy, Art. 73.1.2). The specimen is stored in the Croatian Natural History Museum, Zagreb, coll. no. 3741-1381.

##### Remarks.

The name “*pterochyla*” as mentioned in Pană (2003: 322) is an incorrect subsequent spelling.

#### Melanopsis costata var. pulchella

Taxon classificationAnimaliaSorbeoconchaMelanopsidae

Bourguignat, 1884 [unresolved]

##### Original source.

Bourguignat 1884: 141.

##### Type locality.

“Du Lac d’Homs” (Lorcard 1883: 288) [in Lake Homs], Syria.

##### Remarks.

Introduced to replace - for whatever reason - the variety “*gracilis*” mentioned by Locard (1883a: 288), making *pulchella* its junior objective synonym. *Melanopsis gracilis* Locard, 1883 is a junior homonym of *Melanopsis gracilis* Brusina, 1874, which makes *pulchella* the next available name. However, *Melanopsis costatapulchella* is a homonym of the simultaneously introduced *Melanopsis seignetti pulchella* Bourguignat, 1884 (see Note 1). The action of a First Reviser is required to determine which of both *pulchella* is to be treated as valid.

#### Melanopsis seignetti var. pulchella

Taxon classificationAnimaliaSorbeoconchaMelanopsidae

Bourguignat, 1884 [unresolved]

##### Original source.

Bourguignat 1884: 104.

##### Type locality.

“[Dans les oasis du sud de la province d’Oran; [...] l’oasis Sidi Yousef, à l’extrême sud de la frontière du Maroc; [...] ruisseau d’eau chaude à Ouargla]” [in the oases in the south of the province Oran; oasis Sidi Youcef, at the far southern border of Morocco; hot water stream at Ouargla], Algeria.

##### Remarks.

Homonym of the simultaneously introduced *Melania costata pulchella* Bourguignat, 1884 (see Note 1). The action of a First Reviser is required to determine which of both *pulchella* is to be treated as valid.

#### Melanopsis edrissiana var. pulchella

Taxon classificationAnimaliaSorbeoconchaMelanopsidae

Pallary, 1920 [invalid]

##### Original source.

Pallary 1920a: 33.

##### Type locality.

“Dans une source entre Sidi Abdallah et Koudiat (région de Taza) et dans l’Innaouen, à Sidi Abdallah” [in a source between Douar Sidi Abdellah and Douar El Koudiat (Taza region) and in the Oued Abiod (?) in Douar Sidi Abdellah], Morocco.

##### Remarks.

Junior homonym of *Melanopsis costata pulchella* Bourguignat, 1884 and *Melanopsis seignetti pulchella* Bourguignat, 1884 (simultaneously published; no priority fixed yet; see Note 1).

#### † Lyrcaea [sic] pumila

Taxon classificationAnimaliaSorbeoconchaMelanopsidae

Brusina, 1902

##### Original source.

Brusina 1902: pl. 5, figs 37–38.

##### Type horizon.

Middle Pannonian, late Miocene.

##### Type locality.

“Markuševec”, Croatia.

##### Types.

The illustrated syntypes are stored in the Croatian Natural History Museum, Zagreb, coll. no. 2491-137/1-2 (Milan et al. 1974: 84).

#### † Melanopsis punctata

Taxon classificationAnimaliaSorbeoconchaMelanopsidae

Stoliczka, 1860

##### Original source.

Stoliczka 1860: 485, pl. 1, figs 5a–b.

##### Type horizon.

Late Turonian–Coniacian, late Cretaceous.

##### Type locality.

“Abtenau”, Austria.

##### Remarks.

Kollmann (1984: 57) considered this species as a junior synonym of *Megalonoda reussi* (Hörnes, 1855).

#### † Melanopsis pusilla

Taxon classificationAnimaliaSorbeoconchaMelanopsidae

Handmann, 1882

##### Original source.

Handmann 1882: 561.

##### Type horizon.

Pannonian, zone D, late Miocene.

##### Type locality.

“Kottingbrunn [...] Ziegelei a”, Austria.

#### Fagotia pusilla

Taxon classificationAnimaliaSorbeoconchaMelanopsidae

Bourguignat, 1884

##### Original source.

Bourguignat 1884: 37.

##### Type locality.

“Rives de la Save près Sissek, en Slavonie” [Sava river near Sisak in Slavonia], Croatia.

##### Remarks.

Bourguignat denoted the authority as “Servain, 1884”, but there is no evidence that the description really derived from that author. Starobogatov et al. (1992: 60) considered the species as a junior synonym of *Fagotia* [= *Esperiana*] *acroxia* Bourguignat, 1884.

#### † Melanopsis pygmaea

Taxon classificationAnimaliaSorbeoconchaMelanopsidae

Hörnes, 1856

##### Original source.

Hörnes 1851–1856: 599, pl. 49, fig. 13.

##### Type horizon.

Early–middle Pannonian, late Miocene.

##### Type locality.

“Brunn, Gumpoldskirchen, Guntramsdorf, Inzersdorf, Arsenal in Wien, Kroisbach bei Oedenburg” [Brunn am Gebirge, Gumpoldskirchen, Guntramsdorf, Inzersdorf and Arsenal in Vienna (Austria), Fertőrákos (Hungary)].

##### Remarks.

Hörnes attributed the authority to Partsch, apparently based on an “in schedis” determination.

#### Microcolpia pyramidalis

Taxon classificationAnimaliaSorbeoconchaMelanopsidae

Bourguignat, 1884

##### Original source.

Bourguignat 1884: 61.

##### Type locality.

“Dans le Danube, où elle a été rencontrée çà et là depuis Buda-Pesth jusqu’à Ibraila; [...] Pregrada, près de Krapina, en Croatie” [in the Danube river, where it was found here and there between Budapest (Hungary) and Brăila (Romania); Pregrada, near Krapina (Croatia)].

##### Remarks.

Bourguignat (1884) referred to a publication by Lang (1823: 430) [not 1833 as stated by Brot 1879], who mentioned “*Melanopsis pyramidalis* (mihi)” [nomen nudum] in a species list. Starobogatov et al. (1992: 65) considered the species as a junior synonym of *Microcolpia acicularis* (Férussac, 1823).

#### † Melanopsis pyramidalis

Taxon classificationAnimaliaSorbeoconchaMelanopsidae

Pallary, 1920

##### Original source.

Pallary 1920b: 117.

##### Type horizon.

Early Rupelian, Oligocene.

##### Type locality.

“Grossalmerode, Oberzwehren, Mardorf bei Wabern, Frielendorf, Kirchhain, Dannerod, Ofleiden, Mardorf an der Ohm” (Ludwig 1865: 71), Germany.

##### Remarks.

Introduced for *Melanopsis praerosa* [= *Melanopsis praemorsa*] sensu Ludwig, 1865, non Linnaeus, 1758. It was regarded a junior synonym by Wenz (1929: 2740) because Sandberger (1873: 315) had already introduced *Melanopsis hassiaca* as replacement name for misidentified *Melanopsis praemorsa* from that region and time period. Since Sandberger did not explicitly refer to Ludwig’s material, *pyramidalis* and *hassiaca* are not objective synonyms.

#### † “ Melanopsis pyrguloeformis

Taxon classificationAnimaliaSorbeoconchaMelanopsidae

” mentioned in Hermite (1879: 184) [unavailable] 

##### Horizon.

Early Eocene.

##### Locality.

“De Binisalem et de Selva” [from Binissalem and Selva, Mallorca], Spain.

##### Remarks.

Nomen nudum. Given as “*Melania pyrguloeformis*” on p. 328. The name was probably intended as “*pyrgulaeformis*”, but the ligature was mixed up during typesetting. Hermite listed the name in a section called “Espèces nouvelles citées et non décrites” [= “new species identified and not described”], where he listed 18 new names that he intended to describe in the second volume of his “Études géologiques sur les îles Baléares”. That part, however, has never been published, probably because Hermite died in 1880.

#### † Melanopsis (Homalia) pyrula

Taxon classificationAnimaliaSorbeoconchaMelanopsidae

Handmann, 1887

##### Original source.

Handmann 1887: 16, pl. 1, figs 16–24.

##### Type horizon.

Pannonian, zone B–D, late Miocene.

##### Type locality.

“Leobersdorf”, Austria.

##### Remarks.

Wenz (1929: 2758) considered the taxon as a junior synonym of *Melanopsis inermis* Handmann, 1882.

#### † Melanopsis impressa var. pyrulaeformis

Taxon classificationAnimaliaSorbeoconchaMelanopsidae

Pavlović, 1927

##### Original source.

Pavlović 1927: 79, pl. 10, figs 1–4.

##### Type horizon.

Middle Pannonian, late Miocene.

##### Type locality.

“Из Карагача” [from Karagača near Vrčin], Serbia.

##### Types.

The illustrated syntype is stored in the Natural History Museum, Belgrade, coll. no. 222 (Milošević 1962: 22).

#### † Melanopsis pyrum

Taxon classificationAnimaliaSorbeoconchaMelanopsidae

Neumayr in Neumayr & Paul, 1875

##### Original source.

Neumayr and Paul 1875: 48, pl. 8, fig. 33.

##### Type horizon.

Cernikian, Pliocene.

##### Type locality.

“Čaplathal bei Podwin” [Čaplja trench near Slavonski Brod], Croatia.

##### Remarks.

The species epithet is a noun in apposition (*pyrum* = Latin “pear”) and needs not to agree in gender with the generic name (Art. 31.2.1).

#### † “ Melanopsis tothi var. quadrifilosa

Taxon classificationAnimaliaSorbeoconchaMelanopsidae

” mentioned in Brusina (1903: 114) [unavailable]

##### Horizon.

Late Pleistocene–early Holocene.

##### Locality.

“Bischofsbad” [Püspökfürdő, Băile 1 Mai, Lake Pețea], Romania.

##### Remarks.

Nomen nudum (Brusina apparently considered the term self-explanatory). Neubauer et al. (2014d: 125) considered this taxon as a junior synonym of *Microcolpia parreyssii sikorai* (Brusina, 1903).

#### † Melanopsis raphidia

Taxon classificationAnimaliaSorbeoconchaMelanopsidae

Pallary, 1920 [invalid]

##### Original source.

Pallary 1920b: 110.

##### Type horizon.

Plio-Pleistocene.

##### Type locality.

“Du puits Karoubi” (Pallary 1901a: 178) [from the well Karoubi], Algeria.

##### Remarks.

Introduced as replacement name for *Melanopsis acuminata* Pallary, 1901, non “Sandberger” [act. Gümbel, 1861], for which Wenz (1919a: 73) had already introduced the replacement name *Melanopsis pallaryi*. Thus, *Melanopsis raphidia* is a junior objective synonym of *Melanopsis pallaryi*.

#### † Melanopsis (Macrospira) rapiformis

Taxon classificationAnimaliaSorbeoconchaMelanopsidae

Sandberger, 1871

##### Original source.

Sandberger 1870–1875: pl. 13, figs 3–3b.

##### Type horizon.

Bartonian, Eocene.

##### Type locality.

“Castres (Tarn)”, France.

##### Remarks.

Plate 13 of Sandberger’s monograph appeared in 1871, whereas the description on p. 222 was issued in 1872 (Woodward 1906). Erroneously given as “*Melanopsis proboscidea* Desh.” in plate captions.

#### † Microcolpia rara

Taxon classificationAnimaliaSorbeoconchaMelanopsidae

Gozhik in Gozhik & Datsenko, 2007

##### Original source.

Gozhik and Datsenko 2007: 95, pl. 89, fig. 6.

##### Type horizon.

Late Pleistocene.

##### Type locality.

“Aллювия VII террасы р. Днестр у с. Роги” [Alluvial terrace VII of the Dniestr river near Rogi], Ukraine.

##### Types.

Institute of Geological Sciences of the National Academy of Sciences of Ukraine, coll. no. 5243.

#### † Melanoptychia rarinodosa

Taxon classificationAnimaliaSorbeoconchaMelanopsidae

Brusina, 1892

##### Original source.

Brusina 1892: 145.

##### Type horizon.

Middle Pannonian, late Miocene.

##### Type locality.

“Markuševec”, Croatia.

##### Types.

The illustrated syntypes are stored in the Croatian Natural History Museum, Zagreb, coll. no. 2541-187/1-2 (Milan et al. 1974: 100).

#### † Melanopsis rarispina

Taxon classificationAnimaliaSorbeoconchaMelanopsidae

Lörenthey, 1902

##### Original source.

Lörenthey 1902: 215, pl. 17, figs 18–30.

##### Type horizon.

Transdanubian, Pannonian, late Miocene.

##### Type locality.

“Budapest-Köbánya und Tinnye”, Hungary.

##### Remarks.

Papp (1953b) gave *Melanopsis bouei spinosa* Handmann, 1882 and *Melanopsis bouei ventricosa* Handmann, 1882 in synonymy of *Melanopsis rarispina*, although both were published earlier. The name *ventricosa* Handmann is a junior homonym of *Melanopsis ventricosa* Neumayr, 1880 and thus invalid, but *spinosa* is available and has priority over *rarispina*.

#### Melanopsis sevillensis var. rectituda

Taxon classificationAnimaliaSorbeoconchaMelanopsidae

Pallary, 1924

##### Original source.

Pallary 1924: 252, pl. 25, fig. 24.

##### Type locality.

“De la petite rivière de Guadaira qui se jette dans le Guadalquivir” [in the little river Guadaira which flows into the Guadalquivir], Spain.

#### † Melanopsis recurrens

Taxon classificationAnimaliaSorbeoconchaMelanopsidae

Brusina, 1874

##### Original source.

Brusina 1874: 42.

##### Type horizon.

Cernikian, Pliocene.

##### Type locality.

“Bečić; Podvinje (Čaplja) [Čaplja trench near Slavonski Brod]; Sibinj; Nova Gradiška; Kovačevac; Moslavina; Farkašić”, Croatia.

#### † Melanopsis gorceixi reducta

Taxon classificationAnimaliaSorbeoconchaMelanopsidae

Willmann, 1981

##### Original source.

Willmann 1981: 178.

##### Type horizon.

Upper Elia Formation, early Pleistocene.

##### Type locality.

“Oberes Vokasia-Tal südöstlich von Kos-Ort/Kos (Profil K 1)” [upper Vokasia valley, section K 1 (near Paradeísi)], Greece.

##### Types.

Geological-Paleontological Institute, University of Kiel, Germany; no number indicated.

#### † ? Melanopsis reineri

Taxon classificationAnimaliaSorbeoconchaMelanopsidae

Penecke, 1885

##### Original source.

Penecke 1885: 363, pl. 4, figs 8–9.

##### Type horizon.

Paleocene.

##### Type locality.

“Im südlichen Muldenflügel des Sonnberges (p. 334–335) [on the southern mountainside of the Sonnberg (near Guttaring, Carinthia)], Austria.

#### † Melanopsis requenensis

Taxon classificationAnimaliaSorbeoconchaMelanopsidae

Pallary, 1925

##### Original source.

Pallary 1925: 257.

##### Type horizon.

Late Miocene.

##### Type locality.

“Meseta de Requena y Ayora (Valencia)” (Royo Gomez 1922: 110) [Plateau of Requena and Ayora], Spain.

##### Remarks.

Introduced for *Melania costata* sensu Royo Gomez, 1922, non Olivier, 1804.

#### Melanopsis retoutiana

Taxon classificationAnimaliaSorbeoconchaMelanopsidae

Gassies, 1861

##### Original source.

Gassies 1861: 293, pl. 6, fig. 9.

##### Type locality.

“L’intérieur de la Nouvelle-Calédonie” [the interior of New Caledonia].

##### Remarks.

Brot (1879: 454) considered the species as a junior synonym of *Melanopsis carinata* Gassies, 1861, which is a junior homonym of *Melanopsis carinata* Sowerby, 1826 (see *Melanopsis ducosi* Pallary, 1916).

#### † Melanopsis retusa

Taxon classificationAnimaliaSorbeoconchaMelanopsidae

Brusina, 1904

##### Original source.

Brusina 1904: 495, pl. 1, fig. 3.

##### Type horizon.

Langhian, middle Miocene.

##### Type locality.

“Varcar-Vakufa” [Mrkonjić Grad], Bosnia and Herzegovina.

#### † Purpuroidea reussi

Taxon classificationAnimaliaSorbeoconchaMelanopsidae

Hörnes, 1855

##### Original source.

Hörnes 1855: 177, pl. 2, figs 1a–b.

##### Type horizon.

Late Turonian, late Cretaceous.

##### Type locality.

“Aus der Gams, nordwestlich von Hieflau in Steiermark” [from Gams, northwest of Hieflau in Styria], Austria.

##### Types.

Kollmann (1984: 57) designated a lectotype, which is stored in the collection of the Geological Survey Austria, Vienna (coll. no. 1856/3/4).

##### Remarks.

Type species of the melanopsid genus *Megalonoda* Kollmann, 1984.

#### † Melanopsis revelata

Taxon classificationAnimaliaSorbeoconchaMelanopsidae

Pallary, 1920 

##### Original source.

Pallary 1920b: 110.

##### Type horizon.

Pliocene.

##### Type locality.

“Megara” (Fuchs 1877: 11), Greece.

##### Remarks.

Replacement name for *Melanopsis incerta* Fuchs, 1877, non Férussac, 1822.

#### † Melanopsis rhodanica

Taxon classificationAnimaliaSorbeoconchaMelanopsidae

Locard, 1883

##### Original source.

Locard 1883b: 44, pl. 3, fig. 6.

##### Type horizon.

Mammal zone MN 10–12, late Miocene.

##### Type locality.

“Boulées” [brook Boulées near Miribel], France.

##### Remarks.

Appeared first as a nomen nudum in Falsan and Locard (1879).

#### Melanopsis ricardi

Taxon classificationAnimaliaSorbeoconchaMelanopsidae

Pallary, 1918

##### Original source.

Pallary 1918: 150.

##### Type locality.

“Fès, dans les séguias. Ras el Mâ, à 16 kilom. de Fès” [Fes, in irrigiation channels; Ras el Ma, 16 km from Fes], Morocco.

#### † Melania ricinus

Taxon classificationAnimaliaSorbeoconchaMelanopsidae

Neumayr in Neumayr & Paul, 1875

##### Original source.

Neumayr and Paul 1875: 36, pl. 7, fig. 34.

##### Type horizon.

Cernikian, Pliocene.

##### Type locality.

“Cigelnik [Ciglenik]; [...] Novska”, Croatia.

##### Remarks.

Neumayr mentioned uncertainties regarding the locality Ciglenik because he had lost the original collection label. The species epithet is a noun in apposition and needs not to agree in gender with the generic name (Art. 31.2.1). Considered to belong in the genus *Amphimelania* by Brusina (1897: 6), Cossmann (1909: 126) and Wenz (1929: 2876).

#### Melanopsis rifi

Taxon classificationAnimaliaSorbeoconchaMelanopsidae

Ahuir Galindo, 2014

##### Original source.

Ahuir Galindo 2014a: 14, unnumbered figure.

##### Type locality.

“Spring from the Southeastern Rich region”, Morocco.

##### Types.

Museo Malacologico di Cupra Marittima, Italy; no number indicated.

#### † Melanopsis ripajensis

Taxon classificationAnimaliaSorbeoconchaMelanopsidae

[sic] Pallary, 1916 [invalid] 

##### Original source.

Pallary 1916: 86.

##### Type horizon.

Pannonian, zone D–E, late Miocene.

##### Type locality.

“Ripanj” (Brusina 1902: captions of pl. 5), Serbia.

##### Remarks.

Introduced by Pallary (1916) for the alleged junior homonym “*Melanopsis paulovici* Brusina, 1902”, non Bourguignat, 1880. However, Brusina (1902) had named his species “*pavlovici*”, which makes *Melanopsis ripajensis* its junior objective synonym. Note that Art. 58.4. (regarding the use of u or v for the same Latin letter) does not apply here, because the names are not based on Latin words but on the names of two different persons.

#### † Melanopsis haueri ripanjensis

Taxon classificationAnimaliaSorbeoconchaMelanopsidae

Neubauer, Harzhauser, Kroh, Georgopoulou & Mandic, 2014

##### Original source.

Neubauer et al. 2014c: 16.

##### Type horizon.

Pannonian, zone D–E, late Miocene.

##### Type locality.

“Ripanj” (Brusina 1902: captions of pl. 6), Serbia.

##### Types.

Syntypes are stored in the Croatian Natural History Museum, Zagreb, coll. no. 2530-176/1-2 (Milan et al. 1974: 86).

##### Remarks.

Replacement name for *Melanopsis haueri serbica* Brusina, 1902, non Brusina, 1893 (see Note 1).

#### Melanopsis robusta

Taxon classificationAnimaliaSorbeoconchaMelanopsidae

Gassies, 1870

##### Original source.

Gassies 1870: 147.

##### Type locality.

“Insula Ouen” [Île Ouen], New Caledonia.

#### Microcolpia rochebruniana

Taxon classificationAnimaliaSorbeoconchaMelanopsidae

Bourguignat, 1884

##### Original source.

Bourguignat 1884: 57.

##### Type locality.

“Lac Sabandja” [Lake Sapanca], Turkey.

##### Remarks.

Starobogatov et al. (1992: 68) considered the species as a junior synonym of *Microcolpia coutagniana* (Pfeiffer, 1828).

#### † Melanopsis subulata var. romejacensis

Taxon classificationAnimaliaSorbeoconchaMelanopsidae

Fontannes, 1884

##### Original source.

Fontannes 1884: 29, pl. 4, figs 3–4.

##### Type horizon.

Early Rupelian, Oligocene.

##### Type locality.

“Roméjac, près de Barjac (Gard)”, France.

#### Melanopsis roseni

Taxon classificationAnimaliaSorbeoconchaMelanopsidae

Izzatullaev & Starobogatov, 1984

##### Original source.

Izzatullaev and Starobogatov 1984: 1479, fig. 1 (11).

##### Type locality.

“Закаспийская область” [Transcaspian Region], Russia.

##### Types.

Zoological Institute of Russian Academy of Sciences, St.-Petersburg; no number indicated.

#### † Fagotia roseni

Taxon classificationAnimaliaSorbeoconchaMelanopsidae

Starobogatov in Starobogatov et al., 1992

##### Original source.

Starobogatov et al. 1992: 62, figs 2 (3), 3 (4–5).

##### Type horizon.

Quaternary.

##### Type locality.

“Кулеви (быв. Редут-Кале)” [Q’ulevi (also read as Kulevi), former Redut-Kale], Georgia.

##### Types.

Zoological Institute of Russian Academy of Sciences, St.-Petersburg; no number indicated.

#### Melanopsis rossiteri

Taxon classificationAnimaliaSorbeoconchaMelanopsidae

Gassies, 1880

##### Original source.

Gassies 1880: 85.

##### Type locality.

“Prope Kanala [...]; insula Ouen” (Gassies 1870: 148) [near Canala; Île Ouen], New Caledonia.

##### Remarks.

Replacement name for *Melanopsis fusiformis* Gassies, 1870, non Sowerby, 1822.

#### Fagotia rossmaessleri

Taxon classificationAnimaliaSorbeoconchaMelanopsidae

Bourguignat, 1884

##### Original source.

Bourguignat 1884: 34.

##### Type locality.

“Ruisseau de Pregrada, près Krapina-Toeplilz, en Croatie” [Pregrada river near Krapinske toplice], Croatia.

##### Remarks.

Note that Bourguignat denoted the authority as “Bourguignat, 1880”. Starobogatov et al. (1992: 60) considered the species as a junior synonym of *Fagotia* [= *Esperiana*] *esperi* (Férussac, 1823).

#### Melanopsis rossmaessleri

Taxon classificationAnimaliaSorbeoconchaMelanopsidae

Bourguignat, 1884

##### Original source.

Bourguignat 1884: 158.

##### Type locality.

“Spanien” [Spain, no locality indicated by Rossmässler 1839: 42].

##### Remarks.

Introduced for *Melanopsis cariosa* sensu Rossmässler, 1839, non Linnaeus, 1767.

#### “ Melanopsis rothii

Taxon classificationAnimaliaSorbeoconchaMelanopsidae

Ziegl.” mentioned in Brot (1874–1879: 419) [unavailable]

##### Locality.

Not indicated.

##### Remarks.

Nomen nudum, apparently based on an unpublished manuscript name from Ziegler and listed in synonymy of *Melanopsis buccinoidea* (Olivier, 1801) by Brot (1879).

#### † Melanopsis hybostoma var. rotundata

Taxon classificationAnimaliaSorbeoconchaMelanopsidae

Magrograssi, 1928

##### Original source.

Magrograssi 1928: 260, pl. 6, fig. 20.

##### Type horizon.

Plio-Pleistocene.

##### Type locality.

“Coo: V. Iracli, V. S. Giorgio” [Kos island: Irakli valley, Ágios Geórgios valley (?)], Greece.

#### † Melanopsis rudis

Taxon classificationAnimaliaSorbeoconchaMelanopsidae

Brusina, 1902

##### Original source.

Brusina 1902: pl. 5, figs 51–54.

##### Type horizon.

Early–middle Pannonian, late Miocene.

##### Type locality.

“Jazvine” [Jazvina], Croatia.

##### Types.

The syntypes are stored in the Croatian Natural History Museum, Zagreb; no number indicated (Milan et al. 1974: 96).

##### Remarks.

Wenz (1929: 2824) considered the taxon as a junior synonym of *Melanopsis senatoria* Handmann, 1887.

#### † Melanopsis rugosa

Taxon classificationAnimaliaSorbeoconchaMelanopsidae

Matheron, 1842

##### Original source.

Matheron 1842: 293, pl. 37, fig. 11.

##### Type horizon.

Early Campanian, Cretaceous.

##### Type locality.

“Les Martigues”, France.

##### Remarks.

D’Orbigny (1850) considered this species a *Melania* and a junior secondary homonym of *Melania rugosa* Lea, 1842 (which in turn is a replacement name for *Melanopsis corrugata* Lea, 1841, non Lamarck, 1804). However, the first one was published earlier (August 1842 vs. November/December 1842; for details on publication dates of Matheron’s work see note at the beginning of the Reference section).

#### † Melanopsis (Martinia) martiniana var. rugosa

Taxon classificationAnimaliaSorbeoconchaMelanopsidae

Handmann, 1887 [invalid] 

##### Original source.

Handmann 1887: 26, pl. 5, figs 5–7.

##### Type horizon.

Pannonian, zone B–D, late Miocene.

##### Type locality.

“Leobersdorf”, Austria.

##### Types.

It is uncertain whether the single specimen from Wittmannsdorf near Leobersdorf stored in the Geological Survey Austria, Vienna, is really the only specimen left of the original type series (i.e., holotype by monotypy), as was considered by Fischer (1996b: 23). The statement by Fischer (1996b) did not suffice for a valid lectotype designation.

##### Remarks.

Junior homonym of *Melanopsis rugosa* Matheron, 1842. Neubauer et al. (2014c: 17) introduced *Melanopsis wolfgangfischeri* as replacement name.

#### † Melanopsis lanzaeana f. rugosa

Taxon classificationAnimaliaSorbeoconchaMelanopsidae

Brusina, 1897 [invalid]

##### Original source.

Brusina 1897: 12.

##### Type horizon.

Langhian, middle Miocene.

##### Type locality.

“Ribarić”, Croatia.

##### Types.

Milan et al. (1974: 94) indicated a holotype, but it is uncertain whether the specimen is part of the original type series and whether it was the only one Brusina had at hand (holotype by monotypy, Art. 73.1.2). The specimen is stored in the Croatian Natural History Museum, Zagreb, coll. no. 2983-629.

##### Remarks.

Junior homonym of *Melanopsis rugosa* Matheron, 1842. Neubauer et al. (2011: 205) considered the taxon as a junior synonym of *Melanopsis lanzaeana* Brusina, 1874.

#### † Melanopsis doboi rugosa

Taxon classificationAnimaliaSorbeoconchaMelanopsidae

Schréter, 1975 [invalid]

##### Original source.

Schréter 1975: 11, pl. 3, figs 15–18.

##### Type horizon.

Riss/Würm end to early Würm Ice Age, Pleistocene.

##### Type locality.

“Eger, az egri vár Zárkándy bástyájának vasúti átmetszése” [Eger, section at the railway at the Zarkandy bastion of the fortress Eger], Hungary.

##### Types.

Magyar Állami Földtani Intézet (Hungarian Geological Museum), Budapest; no number indicated.

##### Remarks.

Junior homonym of *Melanopsis rugosa* Matheron, 1842. Neubauer et al. (2016a) attributed the species *Melanopsis doboi* to the genus *Microcolpia*.

#### † Melanopsis matheroni var. rugosocarinata

Taxon classificationAnimaliaSorbeoconchaMelanopsidae

Fontannes, 1880

##### Original source.

Fontannes 1879–1882: 176.

##### Type horizon.

Miocene or Pliocene.

##### Type locality.

Rhône Basin? (no exact locality given), France.

##### Remarks.

Originally written as “*rugoso-carinata*”. Based on material from the Rhône Basin, not Italy as claimed by Wenz (1929); probably the taxon was mixed up by Italian authors cited by Wenz (1929).

#### † Melanopsis tothi var. rugosula

Taxon classificationAnimaliaSorbeoconchaMelanopsidae

Brusina, 1903 [invalid]

##### Original source.

Brusina 1903: 114.

##### Type horizon.

Late Pleistocene–early Holocene.

##### Type locality.

“Bischofsbad” [Püspökfürdő, Băile 1 Mai, Lake Pețea], Romania.

##### Remarks.

Junior objective synonym of *Melanopsis tothi*: Brusina (1903) indicated it as the typical form of the species. Neubauer et al. (2014d: 125) considered this taxon as a junior synonym of *Microcolpia parreyssii sikorai* (Brusina, 1903).

#### “ Melanopsis ruinarum

Taxon classificationAnimaliaSorbeoconchaMelanopsidae

” mentioned in Brot (1874–1879: 419–420, pl. 45, fig. 4) [unavailable]

##### Locality.

“Balbeck” [Baalbek], Lebanon.

##### Remarks.

Introduced in synonymy of *Melania buccinoidea* by Brot (1879), referring to an “in schedis” name by Tarnier (see Note 2).

#### † Melanopsis rumana

Taxon classificationAnimaliaSorbeoconchaMelanopsidae

Tournouër, 1880

##### Original source.

Tournouër 1880: 97.

##### Type horizon.

Early Romanian, Pliocene.

##### Type locality.

“[In loco Bukovatzu dicto, prope urbem Rumaniae Craiova]” [Bucovăț, near Craiova], Romania.

#### “ Melania sabljari

Taxon classificationAnimaliaSorbeoconchaMelanopsidae

Kucik” mentioned in Brusina (1867: 86) [unavailable]

##### Locality.

Not indicated.

##### Remarks.

Nomen nudum, based on an “in schedis” name in the collection of Kucik (also read as “Kutschig”).

#### † Melanopsis sabolici

Taxon classificationAnimaliaSorbeoconchaMelanopsidae

Brusina, 1902

##### Original source.

Brusina 1902: pl. 6, figs 6–7.

##### Type horizon.

Cernikian, Pliocene.

##### Type locality.

“Kovačevac” [east of Nova Gradiška], Croatia.

##### Types.

The illustrated syntypes are stored in the Croatian Natural History Museum, Zagreb, coll. no. 2505-151/1-2 (Milan et al. 1974: 96).

#### Melanopsis liocephala var. sabulosa

Taxon classificationAnimaliaSorbeoconchaMelanopsidae

Pallary, 1936

##### Original source.

Pallary 1936: 57.

##### Type locality.

“D’Agouraï” [Agourai, south of Meknes], Morocco.

#### Melanopsis maroccana saharica

Taxon classificationAnimaliaSorbeoconchaMelanopsidae

Bourguignat, 1864

##### Original source.

Bourguignat 1864: 260.

##### Type locality.

Rather unspecifically indicated in text as “Cours d’eau du Sahara” [rivers of the Sahara], but more precisely as “D’Aïn-Sidi-Taifour; Ouargla; fontaine d’Oumach près de Biskra” [Sidi Taifour; Ouargla; Oumache spring near Biskra, Algeria] in the plate captions.

#### Fagotia saintsimoniana

Taxon classificationAnimaliaSorbeoconchaMelanopsidae

Bourguignat, 1884

##### Original source.

Bourguignat 1884: 47.

##### Type locality.

“Dans la Save, au-dessous d’Agram, en Croatie” [in the Sava river below Zagreb], Croatia.

##### Remarks.

Originally written as “*Saint-Simoniana*”. Note that Bourguignat denoted the authority as “Bourguignat, 1879”. Starobogatov et al. (1992: 60) considered the species as a junior synonym of *Fagotia* [= *Esperiana*] *acroxia* Bourguignat, 1884.

#### Melanopsis salomonis

Taxon classificationAnimaliaSorbeoconchaMelanopsidae

Bourguignat, 1884

##### Original source.

Bourguignat 1884: 95.

##### Type locality.

“Fossés d’eau stagnante au camp des Pins, et çà et là dans le Liban (Syrie)” [ditches of stagnant water in the “camp des Pins” (not found, said to be near Beirut), and here and there in Lebanon].

##### Remarks.

Appeared first as a nomen nudum in Locard (1883a: 201). Note that Bourguignat denoted the authority as “Bourguignat, 1880”.

#### Melanopsis sancta

Taxon classificationAnimaliaSorbeoconchaMelanopsidae

Bourguignat, 1884

##### Original source.

Bourguignat 1884: 129.

##### Type locality.

“Eliasbrunnen bei Jericho” (Kobelt 1880: 17) [Naẖal Elisha‘ (?) near Jericho], Palestine.

##### Remarks.

Introduced for *Melania costata* sensu Kobelt, 1880 (p. 17, pl. 188, fig. 1901), non Olivier, 1804. Bourguignat denoted authority as “Letourneux, 1882”, but there is no evidence that the description really derived from that author. Heller et al. (2005: 248) considered the species as a junior synonym of *Melanopsis saulcyi* Bourguignat, 1853.

#### † Melanopsis sandbergeri

Taxon classificationAnimaliaSorbeoconchaMelanopsidae

Neumayr, 1869

##### Original source.

Neumayr 1869: 372, pl. 13, fig. 5.

##### Type horizon.

Cernikian, Pliocene.

##### Type locality.

“Repušnica”, Croatia.

#### Fagotia (Fagotia) sangarica

Taxon classificationAnimaliaSorbeoconchaMelanopsidae

Schütt & Bilgin, 1974

##### Original source.

Schütt and Bilgin 1974: 60, figs 1–4.

##### Type locality.

“Sakarya başi, main spring of Sakarya river near village Çifteler, 60 km SE Eskişehir, 160 km WSW of Ankara”, Turkey.

##### Types.

Senckenberg Forschungsinstitut und Naturmuseum Frankfurt, coll. no. SMF 232008.

#### † ? Melanopsis sanmigueli

Taxon classificationAnimaliaSorbeoconchaMelanopsidae

Royo Gómez, 1929

##### Original source.

Royo Gómez 1929: 242, pl. 17, fig. 5.

##### Type horizon.

Lutetian, Eocene.

##### Type locality.

“De Santo Domingo de Silos (Burgos)”, Spain.

##### Remarks.

Originally written as “*san-migueli*”. Probably not a Melanopsidae.

#### Melanopsis saulcyi

Taxon classificationAnimaliaSorbeoconchaMelanopsidae

Bourguignat, 1853

##### Original source.

Bourguignat 1853: 66.

##### Type locality.

“Artouze, en Syrie” [Artouz (or ‘Arţūz), 15 km W of Damascus, Rif Dimashq], Syria.

#### † ? Melanopsis scabrida

Taxon classificationAnimaliaSorbeoconchaMelanopsidae

Youluo, 1978

##### Original source.

Youluo 1978: 155, pl. 25, figs 5–6.

##### Type horizon.

Shahejie Formation (second Member), Eocene.

##### Type locality.

“山东垦利” [Kenli, Shandong Province], China.

##### Remarks.

Originally the gender was indicated as masculine (“*scabridus*”), but *Melanopsis* is feminine, which is why the name must be “*scabrida*”. Probably not a Melanopsidae (perhaps a Hydrobiidae or Pomatiopsidae?).

#### † Melanopsis (Canthidomus) proteus var. scalarata

Taxon classificationAnimaliaSorbeoconchaMelanopsidae

Magrograssi, 1928

##### Original source.

Magrograssi 1928: 260, pl. 6, fig. 18.

##### Type horizon.

Plio-Pleistocene.

##### Type locality.

“Coo: V. Iracli e varie località della zona centrale fossilifera” [Kos island: Irakli valley and different locations around the central fossiliferous zone], Greece.

#### † Melanopsis affinis var. scalariformis

Taxon classificationAnimaliaSorbeoconchaMelanopsidae

Handmann, 1882

##### Original source.

Handmann 1882: 558.

##### Type horizon.

Pannonian, zone D, late Miocene.

##### Type locality.

“Kottingbrunn [...] Ziegelei a”, Austria.

##### Remarks.

Not included in the Fossilium Catalogus by Wenz (1929).

#### † Melanopsis scalariformis

Taxon classificationAnimaliaSorbeoconchaMelanopsidae

Papp, 1953 [invalid]

##### Original source.

Papp 1953b: 153, pl. 3, figs 27–28.

##### Type horizon.

Pannonian, zone C, late Miocene.

##### Type locality.

“Leobersdorf Sandgrube” [Leobersdorf sand pit], Austria.

##### Remarks.

Appeared first as nomen nudum in Papp (1951: 109, 147). Junior homonym of *Melanopsis affinis scalariformis* Handmann, 1882 (see Note 1).

#### Melanopsis scalaris

Taxon classificationAnimaliaSorbeoconchaMelanopsidae

Gassies, 1856

##### Original source.

Gassies 1856: 12, figs 7–8.

##### Type locality.

“L’Aïn-Fekan, source d’eau chaude, située entre Mascara et Saïda [...]; l’Oued-M’Ilouya, frontière du Maroc” [‘Aïn Fekan, hot spring between Mascara and Saïda; river Moulouya, at the border to Morocco], Algeria.

#### † Melanopsis scalaris

Taxon classificationAnimaliaSorbeoconchaMelanopsidae

Handmann, 1882 [invalid] 

##### Original source.

Handmann 1882: 559.

##### Type horizon.

Pannonian, zone D, late Miocene.

##### Type locality.

“Kottingbrunn [...] Ziegelei a”, Austria.

##### Remarks.

Junior homonym of *Melanopsis scalaris* Gassies, 1856. Pallary (1916: 83) introduced *Melanopsis limbata* as replacement name. Wenz (1929: 2741) considered the taxon as a junior synonym of *Melanopsis haueri* Handmann, 1882.

#### Melanella elegans var. scalaris

Taxon classificationAnimaliaSorbeoconchaMelanopsidae

Bourguignat, 1884

##### Original source.

Bourguignat 1884: 15.

##### Type locality.

“Au pont de la Save, près d’Agram” [at the bridge of the Sava river near Zagreb], Croatia.

#### Melanopsis parreyssi f. scalaris

Taxon classificationAnimaliaSorbeoconchaMelanopsidae

Westerlund, 1886 [invalid]

##### Original source.

Westerlund 1886: 123.

##### Type locality.

“Siebenbürgen b. Deva” [Deva], Romania.

##### Remarks.

Not available from Brot (1879: 431), who listed the name (“*Melanopsis scalaris* Parreyss in sched.”) as synonym of *Melanopsis Parreyssii* var. β, which he introduced there with a short Latin description. Further below on p. 431, Brot even associated *scalaris* with an illustration of a specimen from Deva in Romania (pl. 46, fig. 15). However, the name was explicitly referred to as synonym of the (unnamed) variety β, which is not a valid name, and thus the requirements of Art. 11.6 (and therefore Art. 12.1) are not met. Westerlund (1886) was the first to describe it and attributed the authority to Parreyss. It is a junior homonym of *Melanopsis scalaris* Gassies, 1856. Neubauer et al. (2014: 125) considered it as a junior synonym of *Microcolpia parreyssii*.

#### † Melanopsis (Canthidomus) scalaritesta

Taxon classificationAnimaliaSorbeoconchaMelanopsidae

Papp, 1953

##### Original source.

Papp 1953a: 109, pl. 23, figs 9–12.

##### Type horizon.

Gelasian, early Pleistocene.

##### Type locality.

“Rómezi (Elis)” [Romésion near Pyrgos], Greece.

#### † Melania (Campylostylus) galloprovincialis var. scalaroides

Taxon classificationAnimaliaSorbeoconchaMelanopsidae

Oppenheim, 1892 [invalid]

##### Original source.

Oppenheim 1892: 765, pl. 30, fig. 4.

##### Type horizon.

Early Campanian, Cretaceous.

##### Type locality.

“Plan de Campagne bei Septême (B.-du-Rhône)” [Plan de Campagne near Septèmes-les-Vallons], France.

##### Remarks.

Junior homonym of *Melania scalaroides* Briart & Cornet, 1882.

#### Melanopsis foleyi var. scalata

Taxon classificationAnimaliaSorbeoconchaMelanopsidae

Pallary, 1928

##### Original source.

Pallary 1928a: 270.

##### Type locality.

“Aïn Mélias, près de Figuig” [Ain Melias near Figuig], Algeria.

#### † Melanopsis pterochila var. scansoria

Taxon classificationAnimaliaSorbeoconchaMelanopsidae

Stefanescu, 1896

##### Original source.

Stefanescu 1896: 130, pl. 11, figs 26, 28, 30–31.

##### Type horizon.

Pliocene (Dacian?).

##### Type locality.

“À Breasta, dans la vallée de Jiu” [near Breasta, in the valley of the river Jiu], Romania.

##### Remarks.

The name “*scansorie*” as mentioned in Pană et al. (1981: 117) is an incorrect subsequent spelling.

#### Microcolpia schileykoi

Taxon classificationAnimaliaSorbeoconchaMelanopsidae

Starobogatov in Starobogatov et al., 1992

##### Original source.

Starobogatov et al. 1992: 67, fig. 3 (18).

##### Type locality.

“Фёслау близ Вены” [Vöslau near Vienna], Austria.

##### Types.

Zoological Institute of Russian Academy of Sciences, St.-Petersburg; no number indicated.

#### † Melanopsis schmidti

Taxon classificationAnimaliaSorbeoconchaMelanopsidae

Neumayr, 1880

##### Original source.

Neumayr 1880b: 289, pl. 1, figs 7–8.

##### Type horizon.

Plio-Pleistocene.

##### Type locality.

“Zwischen Pylle und Antimachia” [between Pýli and Antimácheia, Kos Island], Greece.

##### Remarks.

Willmann (1981: 171) considered this taxon as a junior synonym of *Melanopsis sporadum* Tournouër, 1876.

#### † Melanopsis scripta

Taxon classificationAnimaliaSorbeoconchaMelanopsidae

Fuchs, 1870

##### Original source.

Fuchs 1870b: 544, pl. 22, figs 1–2.

##### Type horizon.

Middle Pannonian, late Miocene.

##### Type locality.

“Kúp”, Hungary.

#### † Melanopsis sculptilis

Taxon classificationAnimaliaSorbeoconchaMelanopsidae

Pallary, 1920

##### Original source.

Pallary 1920b: 119.

##### Type horizon.

Plio-Pleistocene.

##### Type locality.

“Antimaki” (Tournouër 1876: 449, 455) [Antimácheia], Greece.

##### Remarks.

Introduced for *Melania costata* sensu Tournouër, 1876. It was indirectly considered as a junior synonym by Willmann (1981: 177), who was unaware of Pallary’s replacement name but synonymized *Melania costata* sensu Tournouër, 1876 with *Melanopsis heldreichi* Neumayr, 1880.

#### Melanopsis seignetti

Taxon classificationAnimaliaSorbeoconchaMelanopsidae

Bourguignat, 1884

##### Original source.

Bourguignat 1884: 104.

##### Type locality.

“Dans les oasis du sud de la province d’Oran; [...] l’oasis Sidi Yousef, à l’extrême sud de la frontière du Maroc; [...] ruisseau d’eau chaude à Ouargla” [in the oases in the south of the province Oran; oasis Sidi Youcef, at the far southern border of Morocco; hot water stream at Ouargla], Algeria.

##### Remarks.

Note that Bourguignat denoted the authority as “Bourguignat, 1872”.

#### † Melanoptychia lyrata semicostata

Taxon classificationAnimaliaSorbeoconchaMelanopsidae

Olujić, 1999

##### Original source.

Olujić 1999: 20, 48, pl. 1, figs 2–4.

##### Type horizon.

Langhian, middle Miocene.

##### Type locality.

It is unclear from the original work in which of the studied localities/sections along the valleys of the Sutina, Batarelov and Vojskava rivers (4 km W of Sinj) the taxon occurred and in which not, Croatia.

##### Remarks.

Neubauer et al. (2011: 207) considered the taxon as a junior synonym of *Melanopsis lyrata* Neumayr, 1869.

#### Melanopsis semigranulosa

Taxon classificationAnimaliaSorbeoconchaMelanopsidae

Deshayes, 1832

##### Original source.

Deshayes 1830–1832: 438.

##### Type locality.

“Dans l’Ohio” [in the Ohio river], United States.

#### Melanopsis eximia var. semilaevigata

Taxon classificationAnimaliaSorbeoconchaMelanopsidae

Pallary, 1928 [unresolved]

##### Original source.

Pallary 1928a: 260.

##### Type locality.

Not explicitly stated but probably the same as for the species (Agourai, south of Meknès, Morocco).

##### Remarks.

Homonym of the simultaneously published name *Melanopsis seurati semilaevigata*. This case requires the action of a First Reviser.

#### Melanopsis seurati var. semilaevigata

Taxon classificationAnimaliaSorbeoconchaMelanopsidae

Pallary, 1928 [unresolved]

##### Original source.

Pallary 1928a: 268.

##### Type locality.

Not explicitly stated but probably the same as for the species (“La Zousfana, à la hauteur de Figuig et à Beni Ounif” [Oued Zousfana, at the height of Figuig, and at Beni Ounif], Algeria).

##### Remarks.

Homonym of the simultaneously published name *Melanopsis eximia semilaevigata*. This case requires the action of a First Reviser.

#### Melanopsis vanrossomi var. semilaevigata

Taxon classificationAnimaliaSorbeoconchaMelanopsidae

Pallary, 1936 [invalid]

##### Original source.

Pallary 1936: 59.

##### Type locality.

“Tanalt”, Morocco.

##### Remarks.

Junior homonym of *Melanopsis seurati semilaevigata* Pallary, 1928 and *Melanopsis eximia semilaevigata* Pallary, 1928 (simultaneously published; no priority fixed yet; see Note 1).

#### Melanopsis (Mesopotamia) aroussiana var. semilaevigata

Taxon classificationAnimaliaSorbeoconchaMelanopsidae

Pallary, 1939 [invalid]

##### Original source.

Pallary 1939: 101.

##### Type locality.

Not explicitly stated but probably the same as for the species (“‘Ain Arouss” [‘Ayn al ‘Arūs, near Tall Abyaḑ], Syria).

##### Remarks.

Pallary (1939) indicated an illustration (“Fig. 4”) of this taxon, which, however, does not appear as such in the plate captions. Junior homonym of *Melanopsis seurati semilaevigata* Pallary, 1928 and *Melanopsis eximia semilaevigata* Pallary, 1928 (simultaneously published; no priority fixed yet; see Note 1).

#### Melanella holandri var. semiplicata

Taxon classificationAnimaliaSorbeoconchaMelanopsidae

Brusina, 1870

##### Original source.

Brusina 1870: 14.

##### Type locality.

“Dans la Glina près de Topusko” [in the Glina river near Topusko], Croatia.

#### † Melanopsis semiplicata

Taxon classificationAnimaliaSorbeoconchaMelanopsidae

Neumayr, 1880

##### Original source.

Neumayr 1880b: 292, pl. 1, fig. 18.

##### Type horizon.

Phoka to Elia Formation, Plio-Pleistocene.

##### Type locality.

“Phuka” [Ákra Ágios Fokás], Greece.

#### Melanopsis foucauldiana var. semiplicata

Taxon classificationAnimaliaSorbeoconchaMelanopsidae

Pallary, 1928 [invalid]

##### Original source.

Pallary 1928a: 254, pl. 4, fig. 31.

##### Type locality.

Not explicitly stated but probably the same as for the species (“Ougarta, à 60 kil. sud de Beni-Abbès” [Ougarta, prov. Béchar], Algeria).

##### Remarks.

Junior homonym of *Melanopsis semiplicata* Neumayr, 1880.

#### Melanopsis excoriata var. semisulcata

Taxon classificationAnimaliaSorbeoconchaMelanopsidae

Pallary, 1920

##### Original source.

Pallary 1920c: 144.

##### Type locality.

“Aït Brahim”, Morocco.

#### † Melanopsis semperi

Taxon classificationAnimaliaSorbeoconchaMelanopsidae

De Stefani, 1877

##### Original source.

De Stefani 1877: 310.

##### Type horizon.

Villafranchian, Plio-Pleistocene.

##### Type locality.

“Orciano” (Pecchioli 1864: 524) [Orciano Pisano], Italy.

##### Remarks.

Replacement name for *Melanopsis nodosa* Pecchioli, 1864, non Férussac, 1822.

#### † Melanopsis (Lyrcea) senatoria

Taxon classificationAnimaliaSorbeoconchaMelanopsidae

Handmann, 1887

##### Original source.

Handmann 1887: 19, pl. 2, figs 10–11.

##### Type horizon.

Pannonian, zone B–D, late Miocene.

##### Type locality.

“Leobersdorf”, Austria.

#### “ Melanopsis magnifica m. senilis

Taxon classificationAnimaliaSorbeoconchaMelanopsidae

” mentioned in Pérès (1939) [unavailable]

##### Locality.

“Stations 117 et 117his: Oued Bou Fekrane à 13 kilomètres de Meknès sur la route d’El Hajeb” [Stations 117 and 117His: Oued Boufekrane, 13 km S of Meknes on the road to El Hajeb], Morocco.

##### Remarks.

Introduced as infrasubspecific taxon (“mode”), which is not governed by the provisions of the Code. Moreover, the name is a nomen nudum. Pérès referred to an earlier publication of his (Pérès 1938a), claiming that he had described the taxon there already. In that work, however, he only mentioned a subfossil and a “forme modifiée” of *Melanopsis magnifica* Bourguignat, 1884.

#### † Melanopsis lanzae senilis

Taxon classificationAnimaliaSorbeoconchaMelanopsidae

Olujić, 1999

##### Original source.

Olujić 1999: 22, 50, pl. 3, figs 30–32.

##### Type horizon.

Langhian, middle Miocene.

##### Type locality.

It is unclear from the original work in which of the studied localities/sections along the valleys of the Sutina, Batarelov and Vojskava rivers (4 km W of Sinj) the taxon occurred and in which not, Croatia.

##### Remarks.

Neubauer et al. (2011: 205) considered it as a junior synonym of *Melanopsis lanzaeana* Brusina, 1874.

#### † Melanopsis seninskii

Taxon classificationAnimaliaSorbeoconchaMelanopsidae

Wenz, 1928

##### Original source.

Wenz 1928a: 119.

##### Type horizon.

Duab Beds, middle to late Kimmerian, Pliocene.

##### Type locality.

“Моквинскіе пласты, р. Дуабъ” (Seninski 1905: 62) [Mokvi layers at Duab river], Georgia.

##### Remarks.

Replacement name for *Melanopsis acuminata* Seninski, 1905, non Gümbel, 1861.

#### † Melania serbica

Taxon classificationAnimaliaSorbeoconchaMelanopsidae

Živković, 1893

##### Original source.

Živković 1893: 165.

##### Type horizon.

Early Langhian, middle Miocene.

##### Type locality.

“Zvezdan, Crna Reka oberhalb Zaječar, Vražogrnac, Vrbiza, Sumrakovac, Šarbanovac”, Serbia.

##### Remarks.

Considered to belong in the genus *Amphimelania* by Wenz (1929: 2878).

#### † Melanopsis serbica

Taxon classificationAnimaliaSorbeoconchaMelanopsidae

Brusina, 1893 

##### Original source.

Brusina 1893: 50 (only described in Italian part).

##### Type horizon.

Pannonian, zone D–E, late Miocene.

##### Type locality.

“Begaljica”, Serbia.

##### Types.

Milan et al. (1974: 85) indicated a holotype, but it is uncertain whether the specimen actually derives from the original type series and whether it was the only specimen Brusina had at hand. The specimen is stored in the Croatian Natural History Museum, Zagreb, coll. no. 3013-659.

#### † Melanopsis austriaca serbica

Taxon classificationAnimaliaSorbeoconchaMelanopsidae

Brusina, 1902 [invalid]

##### Original source.

Brusina 1902: pl. 6, figs 73–74.

##### Type horizon.

Pannonian, zone D–E, late Miocene.

##### Type locality.

“Ripanj”, Serbia.

##### Types.

The illustrated syntypes are stored in the Croatian Natural History Museum, Zagreb, coll. no. 2530-176/1-2 (Milan et al. 1974: 86).

##### Remarks.

Junior homonym of *Melanopsis serbica* Brusina, 1893. Neubauer et al. (2014c: 16) introduced *Melanopsis haueri ripanjensis* as replacement name, following Wenz (1929: 2741, 2743) who synonymized *Melanopsis austriaca* Handmann, 1882 with *Melanopsis haueri* Handmann, 1882 and listed *serbica* as a subspecies of *Melanopsis haueri*.

#### † Melanopsis serchensis

Taxon classificationAnimaliaSorbeoconchaMelanopsidae

Vidal, 1874

##### Original source.

Vidal 1874: 236, pl. 3, figs 14–14a.

##### Type horizon.

Maastrichtian, Cretaceous.

##### Type locality.

“En Serchs y en Auzas (Alto Garona), pero principalmente en Isona” [in Cercs (Spain) and Auzas (Dép. Haute-Garonne, France), but mainly in Isona (Spain)].

##### Remarks.

Considered to belong to the subgenus *Stilospirula* by Dominici and Kowalke (2014: 148).

#### Melanopsis magnifica var. serira

Taxon classificationAnimaliaSorbeoconchaMelanopsidae

Pallary, 1920

##### Original source.

Pallary 1920c: 153.

##### Type locality.

“Jardin public de bou Jeloud, oued Masmouda, avant son entrée à Fâs el bâli” [park of Bou Jeloud, river Sakiat Masmouda before entry to Fès al Bali], Morocco.

#### † Melanopsis serrensis

Taxon classificationAnimaliaSorbeoconchaMelanopsidae

Mayer-Eymar, 1903

##### Original source.

Mayer-Eymar 1903: 318, pl. 13, fig. 9.

##### Type horizon.

Sparnacian, early Ypresian, Eocene.

##### Type locality.

“Du village de la Serre” [La Serre (?)], France.

#### Microcolpia servaini

Taxon classificationAnimaliaSorbeoconchaMelanopsidae

Bourguignat, 1884

##### Original source.

Bourguignat 1884: 55.

##### Type locality.

“La Save à Sissek (Slavonie) et dans la rivière de Zenica (Bosnie)” [Sava river at Sisak (Croatia) and in the river at Zenica (Bosnia and Herzegovina)].

##### Remarks.

Appeared first as a nomen nudum in Servain (1884). Starobogatov et al. (1992: 65) considered the species as a junior synonym of *Microcolpia cornea* (Pfeiffer, 1828).

#### Fagotia servainiana

Taxon classificationAnimaliaSorbeoconchaMelanopsidae

Bourguignat, 1884

##### Original source.

Bourguignat 1884: 44.

##### Type locality.

“Rivières entre Plaski et Ostaria (Croatie)” [river between Plaški and Oštarije], Croatia.

##### Remarks.

Note that Bourguignat denoted the authority as “Bourguignat, 1882”.

#### Melanopsis sesteri

Taxon classificationAnimaliaSorbeoconchaMelanopsidae

Bourguignat, 1884

##### Original source.

Bourguignat 1884: 119.

##### Type locality.

“Petit cours d’eau à Sadjour-Sou, entre Aïn-Taïb et Alep [...]; Aïn-el-Bass, dans la plaine du Bahr-el-Houlé (Syrie)” [small brook at Sadjour-Sou between Gaziantep (Turkey) and Aleppo (Syria) [...]; Aïn el Bass, in the plains of the Hula valley (Israel)].

##### Remarks.

Heller et al. (2005: 232) considered the species as a junior synonym of *Melanopsis buccinoidea* (Olivier, 1801).

#### Melanopsis seurati

Taxon classificationAnimaliaSorbeoconchaMelanopsidae

Pallary, 1920

##### Original source.

Pallary 1920a: 33.

##### Type locality.

“La Zousfana, à la hauteur de Figuig et à Beni Ounif” [Oued Zousfana, at the height of Figuig, and at Beni Ounif], Algeria.

#### Melanopsis sevillensis

Taxon classificationAnimaliaSorbeoconchaMelanopsidae

Grateloup, 1840

##### Original source.

Grateloup 1840: 433, pl. 4, figs 10–11.

##### Type locality.

“Séville; les bords de la petite rivière de Guadaira, qui se jette dans le Guadalquivir” [Sevilla; the banks of the small river Guadaira, which flows into the Guadalquivir], Spain.

##### Remarks.

The “var. *minor* [...] de Grateloup” as discussed by Bourguignat (1884) was never declared as such by Grateloup (“var. b”). Brot (1874–1879: 441) considered the taxon as a junior synonym of *Melanopsis cariosa* (Linnaeus, 1767).

#### Melanopsis sharhabili

Taxon classificationAnimaliaSorbeoconchaMelanopsidae

Bandel, 2000

##### Original source.

Bandel 2000: 156, figs 119–123.

##### Type locality.

“Spring and creek next to the Wadi Raiyan Plantation in the Jordan Valley near the town of Wadi Raiyan and close to the mosque of the grave of Sharhabil Ibn Hassana”, Jordan.

##### Types.

Geological-Palaeontological Institute and Museum University of Hamburg, coll. no. 4267.

#### Melanopsis sharpei

Taxon classificationAnimaliaSorbeoconchaMelanopsidae

Pallary, 1922

##### Original source.

Pallary 1922: 211.

##### Type locality.

“Aoullouz” [Alous], Morocco.

#### “ Melanopsis siamensis

Taxon classificationAnimaliaSorbeoconchaMelanopsidae

Mrts.” mentioned in Paetel (1888: 404) [unavailable]

##### Locality.

“Siam”, Thailand.

##### Remarks.

Nomen nudum, appears only in the species list of Paetel (1888). Perhaps it is a lapsus calami of *Melampus siamensis* Martens, 1866 (Ellobiidae).

#### † Melanopsis sigmocorrugata

Taxon classificationAnimaliaSorbeoconchaMelanopsidae

Heller & Sivan, 2001 [unavailable]

##### Original source.

Heller and Sivan 2001: 140, fig. 3G.

##### Type horizon.

Pleistocene.

##### Type locality.

“Gesher Benot Ya’aqov”, Syria.

##### Remarks.

Since Heller and Sivan (2001) did not fix a type, the name is unavailable after Art. 16.4.

#### † Melanopsis sikorai

Taxon classificationAnimaliaSorbeoconchaMelanopsidae

Brusina, 1903

##### Original source.

Brusina 1903: 111.

##### Type horizon.

Late Pleistocene–Holocene.

##### Type locality.

“Bischofsbad” [Püspökfürdő, Băile 1 Mai, Lake Pețea], Romania.

##### Remarks.

Considered a subspecies of *Microcolpia parreyssii* by Neubauer et al. (2014d).

#### † Melanopsis similis

Taxon classificationAnimaliaSorbeoconchaMelanopsidae

Pallary, 1916

##### Original source.

Pallary 1916: 84.

##### Type horizon.

Pannonian, zone B–D, late Miocene.

##### Type locality.

“Leobersdorf” (Handmann 1887), Austria.

##### Remarks.

Replacement name for *Melanopsis plicatula* Handmann, 1887, non Brusina, 1874. Wenz (1929: 2674) considered the taxon as a junior synonym of *Melanopsis bouei* Férussac, 1823.

#### † “ Melanopsis sikorai var. siminina

Taxon classificationAnimaliaSorbeoconchaMelanopsidae

” mentioned in Brusina (1903: 112) [unavailable]

##### Horizon.

Late Pleistocene–early Holocene.

##### Locality.

“Bischofsbad” [Püspökfürdő, Băile 1 Mai, Lake Pețea], Romania.

##### Remarks.

Nomen nudum. Listed in synonymy of *Microcolpia parreyssii sikorai* (Brusina, 1903) by Neubauer et al. (2014d: 125).

#### † Melanopsis simulata

Taxon classificationAnimaliaSorbeoconchaMelanopsidae

Pallary, 1925 [invalid]

##### Original source.

Pallary 1925: 257.

##### Type horizon.

Cernikian, Pliocene.

##### Type locality.

“Malino” (Brusina 1878: 348), Croatia.

##### Remarks.

Pallary (1925) introduced *Melanopsis simulata* as replacement name for the presumed junior homonym *Melanopsis arcuata* Brusina, 1878, non Matheron, 1842. The alleged homonymy is, however, based on a reading error of *Melanopsis armata* Matheron, 1842 by Pallary. Therefore, *Melanopsis simulata* is a junior objective synonym of *Melanopsis arcuata* Brusina, 1897.

#### † Melanopsis sinjana

Taxon classificationAnimaliaSorbeoconchaMelanopsidae

Brusina, 1874

##### Original source.

Brusina 1874: 32, pl. 1, figs 1–2.

##### Type horizon.

Early Langhian, middle Miocene.

##### Type locality.

“Sinj (Stuparuša)”, Croatia.

##### Types.

Milan et al. (1974: 97) defined a “neotype” based on one of the specimens illustrated by Brusina (1897). It is uncertain, however, whether the specimen was part of the original type series, which would qualify it as lectotype. Nonetheless, the type fixation is insufficient with respect to Art. 75.3 of the Code. The specimen is stored in the Croatian Natural History Museum, Zagreb, coll. no. 2974-620/1.

#### † Melanopsis sinzowi

Taxon classificationAnimaliaSorbeoconchaMelanopsidae

Brusina, 1885

##### Original source.

Brusina 1885: 160.

##### Type horizon.

*Spaniodon* Beds, Karaganian, middle Miocene.

##### Type locality.

“Лопушны” (Sinzov 1884: 5) [Lăpuşna], Moldova.

##### Remarks.

Introduced for *Melanopsis sinjana* sensu Sinzov, 1884, non Brusina, 1874.

#### † Melanopsis sinzowi

Taxon classificationAnimaliaSorbeoconchaMelanopsidae

Lörenthey, 1902 [invalid]

##### Original source.

Lörenthey 1902: 213, pl. 17, figs 31–32.

##### Type horizon.

Transdanubian, Pannonian, late Miocene.

##### Type locality.

“Tinnye”, Hungary.

##### Remarks.

Junior homonym of *Melanopsis sinzowi* Brusina, 1885. Wenz (1919a: 73) introduced *Melanopsis tinnyensis* as replacement name. Papp (1953b: 145) considered the taxon as a junior synonym of *Melanopsis rarispina* Lörenthey, 1902. However, Papp (1953b) gave the older names *Melanopsis bouei spinosa* Handmann, 1882 and *Melanopsis bouei ventricosa* Handmann, 1882 in synonymy of *Melanopsis rarispina*. While *ventricosa* Handmann is a junior homonym of *Melanopsis ventricosa* Neumayr, 1880 and thus invalid, the name *spinosa* is available and has priority over *rarispina*.

#### Melanopsis sistanica

Taxon classificationAnimaliaSorbeoconchaMelanopsidae

Izzatullaev & Starobogatov, 1984

##### Original source.

Izzatullaev and Starobogatov 1984: 1477, fig. 1 (15), fig. 2 (6), fig. 3 (5).

##### Type locality.

“Восточная Персия” [Eastern Persia], Iran.

##### Types.

Zoological Institute of Russian Academy of Sciences, St.-Petersburg; no number indicated.

##### Remarks.

Type species of Melanopsis (Sistaniana) Izzatullaev & Starobogatov, 1984.

#### † Melanopsis (Canthidomus) costatiformis skuphoi

Taxon classificationAnimaliaSorbeoconchaMelanopsidae

Papp & Psarianos, 1955

##### Original source.

Papp and Psarianos 1955: 148, pl. 23, figs 6–9.

##### Type horizon.

Early Pleistocene.

##### Type locality.

“Tárapsa” [Vasilákion], Greece.

##### Types.

Museum of Palaeontology and Geology of the University of Athens; no number indicated.

#### † Melanopsis (Melanosteira) skurensis

Taxon classificationAnimaliaSorbeoconchaMelanopsidae

Papp, 1955

##### Original source.

Papp 1955: 130, pl. 20, figs 27–30.

##### Type horizon.

Akchagylian, latest Pliocene–earliest Pleistocene.

##### Type locality.

“Skura bei Sparta” [Skoúra], Greece.

##### Types.

Museum of Palaeontology and Geology of the University of Athens; no number indicated.

#### † Melanopsis slavonica

Taxon classificationAnimaliaSorbeoconchaMelanopsidae

Neumayr in Neumayr & Paul, 1875

##### Original source.

Neumayr and Paul 1875: 45, pl. 8, fig. 25.

##### Type horizon.

Cernikian, Pliocene.

##### Type locality.

“Graben zwischen Podwin und der Čapla” [Čaplja trench near Slavonski Brod], Croatia.

#### † Melanopsis sobrievskii

Taxon classificationAnimaliaSorbeoconchaMelanopsidae

Rosen, 1914

##### Original source.

Rosen 1914: 221, pl. 3, fig. 19.

##### Type horizon.

Pleistocene?

##### Type locality.

“Am Fusse des Černjajev’schen Berges bei Suchum” [at the foot of Mt. Černjajev (?) at Sokhumi], Georgia.

##### Remarks.

On p. 221 Rosen gave the species as “*Melanopsis sobrievskii*” within the subgenus *Fagotia*. In the plate captions it appears as “*Fagotia sobrievskii*”.

#### † Melanopsis soceni

Taxon classificationAnimaliaSorbeoconchaMelanopsidae

Jekelius, 1944

##### Original source.

Jekelius 1944: 73, pl. 16, figs 14–17, pl. 51, figs 1–11.

##### Type horizon.

*Mohrensternia* Zone, early Sarmatian, middle Miocene.

##### Type locality.

“Polițioanătal bei Soceni” [Polițioană valley near Soceni], Romania.

#### † Boistelia soceni

Taxon classificationAnimaliaSorbeoconchaMelanopsidae

Harzhauser, Kowalke & Mandic, 2002

##### Original source.

Harzhauser et al. 2002: 99, pl. 7, fig. 6.

##### Type horizon.

Pannonian, zone C–D, late Miocene.

##### Type locality.

“St. Margarethen (Burgenland)” [more precisely, it is the Zollhaus sandpit near St. Margarethen], Austria.

##### Types.

Geological-Paleontological Department, Natural History Museum Vienna, Austria, coll. no. 2001/0126/0049.

##### Remarks.

Neubauer et al. (2014a: 463) considered the taxon as a junior synonym of *Melanopsis soceni* Jekelius, 1944.

#### † Melanopsis sodalis

Taxon classificationAnimaliaSorbeoconchaMelanopsidae

Deshayes, 1862

##### Original source.

Deshayes 1861–1864: 470, pl. 31, figs 14–15.

##### Type horizon.

Thanetian, Paleocene.

##### Type locality.

“Châlons-sur-Vesles, Gueux, Jonchery, Noailles”, France.

#### † Melanopsis soldaniana

Taxon classificationAnimaliaSorbeoconchaMelanopsidae

De Stefani in Pantanelli, 1877

##### Original source.

Pantanelli 1877: 5.

##### Type horizon.

Late Messinian, late Miocene.

##### Type locality.

“Casino, e specialmente presso le Gallozzole” [Casino, and especially at Gallozzole], Italy.

##### Remarks.

Pantanelli (1879: 315, pl. 2, figs 4–5) re-described the species and provided illustrations.

#### Melanopsis sordida

Taxon classificationAnimaliaSorbeoconchaMelanopsidae

Gassies, 1871

##### Original source.

Gassies 1871: pl. 6, fig. 9.

##### Type locality.

New Caledonia [no locality indicated].

##### Remarks.

Gassies (1871) only illustrated but not described the species. Brot (1879: 444) considered the taxon as a junior synonym of *Melanopsis frustulum* Morelet, 1857.

#### † Melanopsis sostarici

Taxon classificationAnimaliaSorbeoconchaMelanopsidae

Brusina, 1897

##### Original source.

Brusina 1897: 8, pl. 6, figs 9–12.

##### Type horizon.

Latest Burdigalian, early Miocene.

##### Type locality.

“Dugoselo”, Croatia.

##### Types.

Milan et al. (1974: 97) indicated a holotype, but it is uncertain whether the specimen was the only one Brusina had at hand (holotype by monotypy, Art. 73.1.2). The specimen is stored in the Croatian Natural History Museum, Zagreb, coll. no. 2999-645.

#### † Melanopsis soubeirani

Taxon classificationAnimaliaSorbeoconchaMelanopsidae

Porumbaru, 1881

##### Original source.

Porumbaru 1881: 28, pl. 9, fig. 1.

##### Type horizon.

Early–middle Romanian, Pliocene.

##### Type locality.

“Cretzesci, Podari” [Crețești, Podari], Romania.

##### Remarks.

The name “*soubeiranus*” as mentioned in Macarovici (1940: 344) is an incorrect subsequent spelling.

#### Melanopsis souverbieana

Taxon classificationAnimaliaSorbeoconchaMelanopsidae

Gassies, 1870

##### Original source.

Gassies 1870: 148.

##### Type locality.

“In Nova Caledonia” [New Caledonia; no locality indicated].

#### † Melanopsis sowerbyi

Taxon classificationAnimaliaSorbeoconchaMelanopsidae

Pallary, 1920 [invalid]

##### Original source.

Pallary 1920b: 116.

##### Type horizon.

Headon Beds, Priabonian, Eocene.

##### Type locality.

“Hordwell” (Sowerby 1821–1823: 36), United Kingdom.

##### Remarks.

Introduced for one of the illustrated specimens of *Melanopsis fusiformis* Sowerby, 1846 (pl. 221, fig. 9) [sic]. The name is based on an error of Pallary (1920b), who only had the French translation of Sowerby’s “Mineral conchology” from 1845 (not 1846) at hand, where figures were organized in a different way than in the original version. Fig. 9 in the 1845-version corresponds to Fig. 7 on pl. 332 of the original publication (Sowerby 1822). Newton (1891) had introduced the new name *Melanopsis pseudosubulata* for that specimen and another specimen already (Sowerby 1821–1823: pl. 332, figs 6–7). Since Pallary mixed up the figures, he considered Newton’s taxonomic separation misleading and, in order to settle the problem, introduced *Melanopsis sowerbyi*. Consequently, *Melanopsis sowerbyi* is a junior objective synonym of *Melanopsis pseudosubulata*.

#### † Melanopsis sparnacensis

Taxon classificationAnimaliaSorbeoconchaMelanopsidae

Pallary, 1916

##### Original source.

Pallary 1916: 77.

##### Type horizon.

Sparnacian, early Ypresian, Eocene.

##### Type locality.

“Épernay”, France.

##### Remarks.

Based on a specimen illustrated by Férussac (1823: pl. 7, fig. 7) as “*Melanopsis buccinoidea* var. γ) antiqua; *elongata*”. Note that *Melanopsis sparnacensis* was not included in the catalogus of Wenz (1929).

#### † Melanopsis (Martinia) martiniana var. spatiosa

Taxon classificationAnimaliaSorbeoconchaMelanopsidae

Handmann, 1887

##### Original source.

Handmann 1887: 25, pl. 4, figs 5–7.

##### Type horizon.

Pannonian, zone B–D, late Miocene.

##### Type locality.

“Leobersdorf”, Austria.

##### Remarks.

Wenz (1929: 2719) considered this taxon as a junior synonym of *Melanopsis fossilis* (Gmelin, 1791).

#### Melanella speciosa

Taxon classificationAnimaliaSorbeoconchaMelanopsidae

Bourguignat, 1884

##### Original source.

Bourguignat 1884: 28.

##### Type locality.

“Rivière entre Ostaria et Plaski, dans la Croatie méridionale” [river between Plaški and Oštarije], Croatia.

##### Remarks.

Note that Bourguignat denoted the authority as “Bourguignat, 1879”.

#### Melanopsis sphaeroidaea

Taxon classificationAnimaliaSorbeoconchaMelanopsidae

Bourguignat, 1884

##### Original source.

Bourguignat 1884: 78.

##### Type locality.

“Dans l’Oronte (Syrie)” [in the Orontes river], Syria?

##### Remarks.

Spelt as “*sphoeroidoea*” on p. 78 but as “*sphaeroidaea*” on p. 73. From the description it is clear that the name must be “*sphaeroidaea*” (see Art. 33.2.1).

#### † Melanopsis bouei var. spinea

Taxon classificationAnimaliaSorbeoconchaMelanopsidae

Stefanescu, 1896

##### Original source.

Stefanescu 1896: 136, pl. 11, figs 65–68.

##### Type horizon.

Khersonian, late Sarmatian, late Miocene.

##### Type locality.

“À Sacel, dans la vallée de Blahnitza, district de Gorjiu” [at Săcelu, in the valley of the Blahnița river, Gorj county], Romania.

##### Remarks.

Wenz (1929: 2678) considered the taxon as a junior synonym of *Melanopsis bouei* Férussac, 1823.

#### † Melanopsis spinicostata

Taxon classificationAnimaliaSorbeoconchaMelanopsidae

Rolle, 1860

##### Original source.

Rolle 1860: 32, pl. 2, figs 6–8.

##### Type horizon.

Late Pliocene to early Pleistocene.

##### Type locality.

“Skalis prope Schoenstein” [Pesje near Šoštanj], Slovenia.

##### Remarks.

The name “*spinicosta*” as mentioned in Wenz (1929: 2831) is an incorrect subsequent spelling.

#### † Trochus spiniger

Taxon classificationAnimaliaSorbeoconchaMelanopsidae

Sowerby in Sedgwick & Murchison, 1832

##### Original source.

Sedgwick and Murchison 1832: 418, pl. 38, fig. 15.

##### Type horizon.

Late Cretaceous.

##### Type locality.

“Gosau”, Austria.

##### Types.

Holotype (?) stored in the collection of the British Museum, coll. no. G 17908 (Kollmann 1984: 58).

##### Remarks.

Kollmann (1984: 58) classified the species in the melanopsid genus *Megalonoda*, which was there described as new.

#### † Melanopsis spinigera

Taxon classificationAnimaliaSorbeoconchaMelanopsidae

Seninski, 1905

##### Original source.

Seninski 1905: 60, pl. 1, figs 19–22.

##### Type horizon.

Duab Beds, middle to late Kimmerian, Pliocene.

##### Type locality.

“Моквинскіе пласты, р. Дуабъ” [Mokvi layers at Duab river], Georgia.

#### † Melanopsis bouei var. spinosa

Taxon classificationAnimaliaSorbeoconchaMelanopsidae

Handmann, 1882

##### Original source.

Handmann 1882: 557.

##### Type horizon.

Pannonian, zone D, late Miocene.

##### Type locality.

“Kottingbrunn [...] Ziegelei a”, Austria.

##### Remarks.

Papp (1953b) listed this variety together with *Melanopsis bouei ventricosa* Handmann, 1882 in synonymy of *Melanopsis rarispina* Lörenthey, 1902, although Handmann’s taxa were published earlier. The name *ventricosa* Handmann is a junior homonym of *Melanopsis ventricosa* Neumayr, 1880 and thus invalid, but *spinosa* is available and has priority over *rarispina*.

#### † Melanopsis spiralis

Taxon classificationAnimaliaSorbeoconchaMelanopsidae

Handmann, 1882

##### Original source.

Handmann 1882: 555.

##### Type horizon.

Pannonian, zone D, late Miocene.

##### Type locality.

“Kottingbrunn [...] Ziegelei a”, Austria.

##### Remarks.

Wenz (1929: 2718) considered this taxon as a junior synonym of *Melanopsis fossilis* (Gmelin, 1791).

#### Melanopsis spirata

Taxon classificationAnimaliaSorbeoconchaMelanopsidae

Chenu, 1859

##### Original source.

Chenu 1859: 297, fig. 2062.

##### Type horizon.

Not stated; unclear if recent or fossil.

##### Type locality.

Not indicated.

##### Remarks.

The species is based on a single illustration, without description or any kind of explanation.

#### † Melanopsis avellana var. spirata

Taxon classificationAnimaliaSorbeoconchaMelanopsidae

Leymerie, 1881 [invalid]

##### Original source.

Leymerie 1881: 777, pl. O, fig. 2.

##### Type horizon.

Maastrichtian, Cretaceous.

##### Type locality.

“Auzas”, France.

##### Remarks.

Junior homonym of *Melanopsis spriata* Chenu, 1859.

#### † Melanopsis spiridioni

Taxon classificationAnimaliaSorbeoconchaMelanopsidae

Pallary, 1916

##### Original source.

Pallary 1916: 81.

##### Type horizon.

Latest Burdigalian, early Miocene.

##### Type locality.

“Dugoselo” (Brusina 1897: 9), Croatia.

##### Remarks.

Introduced for *Melanopsis praemorsa* sensu Brusina, 1897, non Linnaeus, 1758. Wenz (1929: 2831–2833) listed also other misidentied *Melanopsis praemorsa* from the late Miocene deposits of Lake Pannon and the Pliocene of Greece and Croatia under that name, but it is very unlikely that all of them belong to the same species.

#### † Melanopsis sporadum

Taxon classificationAnimaliaSorbeoconchaMelanopsidae

Tournouër, 1876

##### Original source.

Tournouër 1876: 453, pl. 4, fig. 4.

##### Type horizon.

Phoka to Elia Formation, Plio-Pleistocene.

##### Type locality.

“Fouka” (p. 449) [Ákra Ágios Fokás], Greece.

#### † Melanopsis aetolica var. stamnana

Taxon classificationAnimaliaSorbeoconchaMelanopsidae

Oppenheim, 1891

##### Original source.

Oppenheim 1891: 469, pl. 27, figs 3–4.

##### Type horizon.

Gelasian, early Pleistocene.

##### Type locality.

“Stamna”, Greece.

##### Remarks.

Not available from Oppenheim (1890b), where he gave it as “mutation”. Probably by mistake, Papp (1955, in figure captions) designated a lectotype based on a specimen from Neumayr’s (1880a) (not Oppenheim’s) original material.

#### † Melanopsis (Duabiana) starobogatovi

Taxon classificationAnimaliaSorbeoconchaMelanopsidae

Anistratenko, 1993

##### Original source.

Anistratenko 1993: 70, textfig. 1.

##### Type horizon.

Duab Beds, middle to late Kimmerian, Pliocene.

##### Type locality.

“Окр. с. Мокви, Очамчирский р-н” [near the village Mok’vi, Ochamchirskiy rayon], Georgia.

##### Types.

Schmalhausen Institute of Zoology of National Academy of Sciences of Ukraine, Kiev; no number indicated.

#### Melanopsis starostini

Taxon classificationAnimaliaSorbeoconchaMelanopsidae

Izzatullaev & Starobogatov, 1984

##### Original source.

Izzatullaev and Starobogatov 1984: 1480, fig. 1 (13), fig. 3 (7).

##### Type locality.

“Гермоб (Центральный Копетдаг)” [Germob (central Kopetdag)], Iran.

##### Types.

Zoological Institute of Russian Academy of Sciences, St.-Petersburg; no number indicated.

#### † Melanopsis staubi

Taxon classificationAnimaliaSorbeoconchaMelanopsidae

Brusina, 1903

##### Original source.

Brusina 1903: 115.

##### Type horizon.

Late Pleistocene–Holocene.

##### Type locality.

“Bischofsbad” [Püspökfürdő, Băile 1 Mai, Lake Pețea], Romania.

##### Remarks.

Neubauer et al. (2014d: 125) considered this taxon as a junior synonym of *Microcolpia parreyssii sikorai* (Brusina, 1903).

#### Melanopsis (Mesopotamia) infracincta var. stena

Taxon classificationAnimaliaSorbeoconchaMelanopsidae

Pallary, 1939

##### Original source.

Pallary 1939: 104.

##### Type locality.

“Ras el ‘Ain du Khabour” [Chabur river near Ra’s al ‘Ayn], Syria.

#### Fagotia stenostoma

Taxon classificationAnimaliaSorbeoconchaMelanopsidae

Bourguignat, 1884

##### Original source.

Bourguignat 1884: 42.

##### Type locality.

“La Save au-dessous d’Agram; rivières au sud de Krapina-Toeplitz, et entre Plaski et Ostaria (Croatie)” [Sava river below Zagreb; rivers south of Krapinske toplice, and between Plaški and Oštarije], Croatia.

##### Remarks.

Note that Bourguignat denoted the authority as “Bourguignat, 1879”. Starobogatov et al. (1992: 60) considered the species as a junior synonym of *Fagotia* [= *Esperiana*] *acroxia* Bourguignat, 1884.

#### Melania parvula f. stenostoma

Taxon classificationAnimaliaSorbeoconchaMelanopsidae

Westerlund, 1886

##### Original source.

Westerlund 1886: 108.

##### Type locality.

“Der untere Lauf jenes kleinen Gebirgsbaches, welcher den Abhängen des Berges Kopitnig entspringt, am hochgelegenen Kurhause von Römerbad vorbeieilend, dessen Thermenabfluss aufnimmt, und in starkem Gefälle der den Fuss des Berges umspülenden Sann zufliest” (Tschapeck 1881: 105–106) [in the lower reaches of a small mountain brook, which originates at the slopes of Mt. Kopitnik, passes the kurhaus of Rimske Toplice, takes its thermal water runoff, and flows over a steep gradient into the Savinja river], Slovenia.

##### Remarks.

Introduced for a specimen of *Melania holandri* illustrated by Tschapeck (1881: 106, pl. 5, fig. k).

#### Melanopsis stephanota

Taxon classificationAnimaliaSorbeoconchaMelanopsidae

Bourguignat, 1884

##### Original source.

Bourguignat 1884: 120.

##### Type locality.

Rossmässler, to whom Bourguignat referred, indicated the locality only as “Palästina” [Palestine]. As the species was explicitly introduced for Rossmässler’s material only this is the type locality.

##### Remarks.

Introduced for *Melania costata* sensu Kobelt, 1880 (figs 1899–1900), non Olivier, 1804.

#### Melanopsis (Mesopotamia) stolliana

Taxon classificationAnimaliaSorbeoconchaMelanopsidae

Pallary, 1939

##### Original source.

Pallary 1939: 102, pl. 5, figs 44–48.

##### Type locality.

“‘Ain Arouss” [‘Ayn al ‘Arūs (near Tall Abyaḑ)], Syria.

#### Microcolpia stossichiana

Taxon classificationAnimaliaSorbeoconchaMelanopsidae

Bourguignat, 1884

##### Original source.

Bourguignat 1884: 64.

##### Type locality.

“Carniole” [Carniola, a historical region that comprised parts of present-day Slovenia; no locality indicated].

##### Remarks.

Starobogatov et al. (1992: 65) considered the species as a junior synonym of *Microcolpia acicularis* (Férussac, 1823).

#### Melanopsis strangei

Taxon classificationAnimaliaSorbeoconchaMelanopsidae

Reeve, 1860

##### Original source.

Reeve 1860: Section *Melanopsis*, pl. 1, figs 3a–b.

##### Type locality.

“New Zealand” [no locality indicated].

##### Remarks.

Suter (1893: 139) considered the taxon as a junior synonym of *Zemelanopsis trifasciata* (Gray in Dieffenbach, 1843).

#### † Melanopsis strangulata

Taxon classificationAnimaliaSorbeoconchaMelanopsidae

Brusina, 1902

##### Original source.

Brusina 1902: pl. 6, figs 46–50.

##### Type horizon.

Cernikian, Pliocene.

##### Type locality.

“Kovačevac” [east of Nova Gradiška], Croatia.

##### Types.

The illustrated syntypes are stored in the Croatian Natural History Museum, Zagreb, coll. no. 2521-167/1-5 (Milan et al. 1974: 97).

#### † Melanopsis olivula var. striata

Taxon classificationAnimaliaSorbeoconchaMelanopsidae

Grateloup, 1838 [invalid]

##### Original source.

Grateloup 1838: 143, pl. 4, fig. 55.

##### Type horizon.

Burdigalian, early Miocene.

##### Type locality.

“Dax. [...] Mandillot”, France.

##### Remarks.

Junior objective synonym of *Melania buccinoidea subventricosa* Grateloup, 1828, which Grateloup (1838) listed in synonymy.

#### Melanopsis maroccana var. striata

Taxon classificationAnimaliaSorbeoconchaMelanopsidae

Pantanelli, 1886

##### Original source.

Pantanelli 1886b: 72, pl. 3, figs 26–27.

##### Type locality.

“Accesa”, Italy.

#### Melania parvula f. striata

Taxon classificationAnimaliaSorbeoconchaMelanopsidae

Westerlund, 1886 [invalid]

##### Original source.

Westerlund 1886: 108.

##### Type locality.

“Steinbrück” [Zidani Most], Slovenia.

##### Remarks.

Junior homonym of *Melania striata* Sowerby, 1818, described from the Paleogene (Eocene?) of the United Kingdom.

#### † Melanopsis (Hyphantria) striata

Taxon classificationAnimaliaSorbeoconchaMelanopsidae

Handmann, 1887 [invalid]

##### Original source.

Handmann 1887: 38, pl. 8, fig. 18.

##### Type horizon.

Pannonian, zone B–D, late Miocene.

##### Type locality.

“Leobersdorf”, Austria.

##### Remarks.

Junior homonym of *Melanopsis maroccana striata* Pantanelli, 1886 (see Note 1).

#### Melanopsis maroccana var. striatacarinata

Taxon classificationAnimaliaSorbeoconchaMelanopsidae

Pantanelli, 1886

##### Original source.

Pantanelli 1886b: 72, pl. 3, figs 24–25.

##### Type locality.

“Accesa”, Italy.

##### Remarks.

Originally written as “*striata-carinata*”.

#### Melanopsis maroccana var. striatasulcata

Taxon classificationAnimaliaSorbeoconchaMelanopsidae

Pantanelli, 1886 

##### Original source.

Pantanelli 1886b: 72, pl. 3, fig. 28.

##### Type locality.

“Accesa”, Italy.

##### Remarks.

Originally written as “*striata-sulcata*”.

#### † Melanopsis (Melanosteira) striatula

Taxon classificationAnimaliaSorbeoconchaMelanopsidae

Pavlović, 1927

##### Original source.

Pavlović 1927: 62, pl. 7, figs 9–10.

##### Type horizon.

Middle Pannonian, late Miocene.

##### Type locality.

“У карагачких жутим песковима” [from the yellow sands of Karagača near Vrčin], Serbia.

##### Types.

The illustrated syntype is stored in the Natural History Museum, Belgrade, coll. no. 212 (Milošević 1962: 23).

#### Melanopsis dufourii var. stricta

Taxon classificationAnimaliaSorbeoconchaMelanopsidae

Pallary, 1924

##### Original source.

Pallary 1924: 249, pl. 15, fig. 4.

##### Type locality.

Spain [no locality indicated].

#### Melanopsis buccinoidea var. stricta

Taxon classificationAnimaliaSorbeoconchaMelanopsidae

Pallary, 1939 [invalid]

##### Original source.

Pallary 1939: 85, pl. 6, figs 64–65.

##### Type locality.

“Environs de Beyrouth” [surroundings of Beirut], Lebanon.

##### Remarks.

Junior homonym of *Melanopsis dufourii stricta* Pallary, 1924 (see Note 1).

#### † Melanopsis stricturata

Taxon classificationAnimaliaSorbeoconchaMelanopsidae

Brusina, 1892

##### Original source.

Brusina 1892: 138.

##### Type horizon.

Middle Pannonian, late Miocene.

##### Type locality.

“Markuševec”, Croatia.

##### Types.

The illustrated syntype is stored in the Croatian Natural History Museum, Zagreb, coll. no. 2496-142/1 (Milan et al. 1974: 97).

#### † Boistelia stricturata

Taxon classificationAnimaliaSorbeoconchaMelanopsidae

Jekelius, 1944 [invalid]

##### Original source.

Jekelius 1944: 136, pl. 55, figs 1–14.

##### Type horizon.

Early Pannonian, late Miocene.

##### Type locality.

“Turislav-Tal bei Soceni” [Turislav valley near Soceni], Romania.

##### Remarks.

Junior secondary homonym and junior synonym of *Melanopsis stricturata* Brusina, 1892 (see discussion in Neubauer et al. 2014a: 463).

#### Melanopsis costata var. strigosa

Taxon classificationAnimaliaSorbeoconchaMelanopsidae

Pallary, 1939

##### Original source.

Pallary 1939: 90, pl. 5, figs 35–36.

##### Type locality.

“Djishr ech Chegour” [Jisr Ash-Shughur], Syria.

##### Remarks.

Heller and Sivan (2002a: 49) considered the taxon as a junior synonym of *Melanopsis multiformis* Blanckenhorn, 1897. Heller et al. (2005: 244) in turn considered it as a junior synonym of *Melanopsis costata* (Olivier, 1804).

#### † Melanopsis sturii

Taxon classificationAnimaliaSorbeoconchaMelanopsidae

Fuchs, 1873

##### Original source.

Fuchs 1873: 21, pl. 4, figs 18–19.

##### Type horizon.

Transdanubian, Pannonian, late Miocene.

##### Type locality.

“Moosbrunn bei Wien; Tinnye bei Ofen” [Moosbrunn near Vienna (Austria); Tinnye (Hungary)].

##### Remarks.

The name “*sturi*” as mentioned in Wenz (1929: 2835) is an incorrect subsequent spelling.

#### Fagotia (Microcolpia) daudebartii stussineri

Taxon classificationAnimaliaSorbeoconchaMelanopsidae

Schütt & Bilgin, 1974

##### Original source.

Schütt and Bilgin 1974: 62, fig. 5.

##### Type locality.

“River Pinios [Pineiós] in Thessalia, Greece, between Larissa and Tempe [Tempón] valley”, Greece.

##### Types.

Senckenberg Forschungsinstitut und Naturmuseum Frankfurt, coll. no. SMF 111523a.

##### Remarks.

The name “*situssineri*” as mentioned in Koşal-Şahin and Yıldırım (2007: 51) is an incorrect subsequent spelling.

#### Melanopsis suarezi

Taxon classificationAnimaliaSorbeoconchaMelanopsidae

Ahuir Galindo, 2016

##### Original source.

Ahuir Galindo 2016: 26.

##### Type locality.

“Southeastern of Guelmin, at river Seyad basin”, Morocco.

##### Types.

Museo Malacologico di Cupra Marittima, Italy; no number indicated.

#### † Melanopsis subaffinis

Taxon classificationAnimaliaSorbeoconchaMelanopsidae

Pallary, 1916 [invalid]

##### Original source.

Pallary 1916: 82.

##### Type horizon.

Pannonian, zone D, late Miocene.

##### Type locality.

“Kottingbrunn [...] Ziegelei a” (Handmann 1882: 558), Austria.

##### Remarks.

Replacement name for the alleged junior homonym *Melanopsis affinis* Handmann, 1882, “non Férussac, 1823”. The latter name is, however, unavailable from Férussac (1823) but was introduced validly by Pallary (1916), who referred to an illustration in Férussac (1823) (see discussion of *Melanopsis affinis*). This makes *Melanopsis subaffinis* an objective synonym of *Melanopsis affinis* Handmann, 1882 and *Melanopsis affinis* Pallary, 1916 a junior homonym of *Melanopsis affinis* Handmann, 1882. *Melanopsis subaffinis* was considered as a junior synonym of *Melanopsis bouei* by Wenz (1929: 2681).

#### Melanopsis maroccana var. subangulata

Taxon classificationAnimaliaSorbeoconchaMelanopsidae

Pallary, 1922

##### Original source.

Pallary 1922: 209.

##### Type locality.

“Marrakech”, Morocco.

#### † Melanopsis subangulosa

Taxon classificationAnimaliaSorbeoconchaMelanopsidae

Sandberger, 1875

##### Original source.

Sandberger 1870–1875: 559.

##### Type horizon.

Kirchberg Formation, middle Burdigalian, early Miocene.

##### Type locality.

“Kirchberg” [Illerkirchberg], Germany.

#### † Melanopsis pygmaea f. subaudebardi

Taxon classificationAnimaliaSorbeoconchaMelanopsidae

Soós in Bartha, 1955

##### Original source.

Bartha 1955: 297, pl. 2, fig. 5.

##### Type horizon.

Transdanubian, Pannonian, late Miocene.

##### Type locality.

“Várpalota”, Hungary.

##### Remarks.

Bartha clearly stated that the description was made by Lajos Soós.

#### † Fagotia subbergeroni

Taxon classificationAnimaliaSorbeoconchaMelanopsidae

Gozhik, 2002

##### Original source.

Gozhik 2002: 54, pl. 3, figs 5, 7, 12, 16.

##### Type horizon.

Middle Pontian (Dacian Basin), late Miocene.

##### Type locality.

“Виноградівка” [Vynohradivka, Odes’ka Oblast’], Ukraine.

##### Types.

Institute of Geological Sciences of the National Academy of Sciences of Ukraine, coll. no. 3185.

#### † Melanopsis subbuccinoides

Taxon classificationAnimaliaSorbeoconchaMelanopsidae

d’Orbigny, 1852

##### Original source.

d’Orbigny 1852: 28.

##### Type horizon.

Burdigalian, early Miocene.

##### Type locality.

“Dax, St-Paul. Mandillot” (Grateloup 1840, in captions of plate “Mollusques terrestres et fluviatiles fossiles de Dax”), France.

##### Remarks.

Introduced for *Melanopsis buccinoides* [sic] sensu Grateloup, 1840, non Olivier, 1801. The name “*sublucinoides*” as mentioned in Glibert (1962: 141) is an incorrect subsequent spelling.

#### † Melanopsis pedemontana subcallosa

Taxon classificationAnimaliaSorbeoconchaMelanopsidae

Wenz, 1928 [invalid]

##### Original source.

Wenz 1928b: 220.

##### Type horizon.

Late Burdigalian, early Miocene.

##### Type locality.

“Colle torinesi” (Sacco 1889: 68) [Torino hills], Italy.

##### Remarks.

Invalid replacement name for the homonym *Melanopsis taurinensis* Sacco, 1889, non *Melanopsis clava taurinensis* Sacco, 1889 (same work). According to Art. 24.1 and 57.7 the name proposed at higher rank takes precedence. This makes *subcallosa* an objective synonym of *Melanopsis taurinensis* Sacco, 1889, while the subspecies *Melanopsis clava taurinensis* Sacco, 1889 is still in need of a substitute name.

#### † Melanopsis subcarinata

Taxon classificationAnimaliaSorbeoconchaMelanopsidae

Deshayes in Férussac, 1839

##### Original source.

Férussac 1819–1851: Mélanopsides fossiles, pl. 2, fig. 3 (altered caption by Deshayes delivered with Livraison 29; see introduction for details).

##### Type horizon.

Late Villafranchian, Pleistocene (?).

##### Type locality.

“D’Italie” (in the original captions, Férussac had given the locality as “entre Saint-Germinin et Carsoli” [between San Gemini and Carsoli, Italy]).

#### † Melanopsis subcarinata

Taxon classificationAnimaliaSorbeoconchaMelanopsidae

Morris in Forbes, 1856 [invalid]

##### Original source.

Forbes 1856: 156, pl. 6, figs 5–6.

##### Type horizon.

Headon Beds, Priabonian, Eocene.

##### Type locality.

“Headon Hill”, United Kingdom.

##### Remarks.

The description was evidently performed by Morris (see bottom of p. 156 in Forbes 1856). Junior homonym of *Melanopsis subcarinata* Deshayes in Férussac, 1851.

#### † Melanopsis subcostata

Taxon classificationAnimaliaSorbeoconchaMelanopsidae

d’Orbigny, 1850

##### Original source.

d’Orbigny 1850: 301.

##### Type horizon.

Sparnacian, early Ypresian, Eocene.

##### Type locality.

d’Orbigny (1850) gave “Antheuil (Oise), Soissons”, Pacaud (2007) only “Antheuil”. Deshayes (1825), however, which the new name was based on, had only mentioned material from Soissons (along with occurrences of *Melania costata* mentioned in other works), France.

##### Remarks.

Introduced for *Melania costata* sensu Deshayes, 1825, non Olivier, 1804.

#### Melanopsis subcostata

Taxon classificationAnimaliaSorbeoconchaMelanopsidae

Bourguignat, 1884 [invalid]

##### Original source.

Bourguignat 1884: 137.

##### Type locality.

“Dans l’Oronte” (Lamarck 1822: 168) [in the Orontes river], Syria?

##### Remarks.

Introduced for *Melania costata* sensu Lamarck, 1822, non Olivier, 1804. Junior homonym of *Melanopsis subcostata* d’Orbigny, 1850. Pallary (1920b: 110–111) synonymized the species with *Melania costata* (Olivier, 1804).

#### † Melanopsis constricta f. subcostata

Taxon classificationAnimaliaSorbeoconchaMelanopsidae

Brusina, 1897 [invalid]

##### Original source.

Brusina 1897: 7.

##### Type horizon.

Late Cernikian, late Pliocene–early Pleistocene.

##### Type locality.

“Novska (Bukovica)”, Croatia.

##### Types.

Milan et al. (1974: 88) indicated a holotype, but it is uncertain whether the specimen was the only one Brusina had at hand (holotype by monotypy, Art. 73.1.2). The specimen is stored in the Croatian Natural History Museum, Zagreb, coll. no. 2993-639.

##### Remarks.

Junior homonym of *Melanopsis subcostata* d’Orbigny, 1850. Wenz (1928a: 120) introduced *Melanopsis constricta nowskaensis* as replacement name.

#### Melanopsis laevigata var. subcostulata

Taxon classificationAnimaliaSorbeoconchaMelanopsidae

Pallary, 1904

##### Original source.

Pallary 1904: 37.

##### Type locality.

“Sriratzel Cromfel sur la route de Rabat à Casablanca; Temslott, dans les canaux; Agagour dans l’Atlas” [Sriratzel Cromfel (?) at the road from Rabat to Casablanca; Temslott (?), in the channels; Agagour (?) in the Atlas Mts.], Morocco.

#### Melanopsis saharica var. subcostulata

Taxon classificationAnimaliaSorbeoconchaMelanopsidae

Pallary, 1912 [invalid]

##### Original source.

Pallary 1912: 15, figs 1–2.

##### Type locality.

“De Ngoussa” [N’Goussa], Algeria.

##### Remarks.

Junior homonym of *Melanopsis subcostulata* Pallary, 1904.

#### Melanopsis praemorsa f. subcylindrica

Taxon classificationAnimaliaSorbeoconchaMelanopsidae

Pérès, 1939 

##### Original source.

Pérès 1939: 135.

##### Type locality.

Several collection stations near Meknès, Fès, Aïn El Aouda and Aïn Chkef, Morocco (Pérès 1939: 140).

#### † Melanopsis dufourii var. subfusiformis

Taxon classificationAnimaliaSorbeoconchaMelanopsidae

Grateloup, 1838

##### Original source.

Grateloup 1838: 142.

##### Type horizon.

Burdigalian, early Miocene.

##### Type locality.

“Dax. Mandillot, à Saint-Paul”, France.

##### Remarks.

Introduced for a specimen of *Melanopsis dufourii* Férussac, 1822 from Dax illustrated in Férussac (1823: 154, pl. 8, fig. 5).

#### † Melanopsis subfusiformis

Taxon classificationAnimaliaSorbeoconchaMelanopsidae

Morris in Forbes, 1856

##### Original source.

Forbes 1856: 155, pl. 6, figs 2–3.

##### Type horizon.

Headon Beds, Priabonian, Eocene.

##### Type locality.

“From the Headon series”, United Kingdom.

##### Remarks.

The description was evidently performed by Morris (see bottom of p. 156 in Forbes 1856).

#### Melanopsis maroccana var. subgraellsiana

Taxon classificationAnimaliaSorbeoconchaMelanopsidae

Bourguignat, 1864

##### Original source.

Bourguignat 1864: 260.

##### Type locality.

“Mascara; Oran”, Algeria.

##### Remarks.

The name “*subgraellsi*” as used by Pallary (1920b: 106) is an incorrect subsequent spelling.

#### † Melanopsis subhercynica

Taxon classificationAnimaliaSorbeoconchaMelanopsidae

Mertin, 1939

##### Original source.

Mertin 193: 207, pl. 6, fig. 5.

##### Type horizon.

Heidelberg Formation, late Santonian, late Cretaceous.

##### Type locality.

“Flugplatz Quedlinburg” [airfield at Quedlinburg], Germany.

#### Melanopsis subimpressa

Taxon classificationAnimaliaSorbeoconchaMelanopsidae

Pallary, 1928

##### Original source.

Pallary 1928a: 262, pl. 6, figs 2–3.

##### Type locality.

“Guefaït (Maroc oriental)”, Morocco.

#### Melanella elegans var. sublaevis

Taxon classificationAnimaliaSorbeoconchaMelanopsidae

Bourguignat, 1884

##### Original source.

Bourguignat 1884: 15.

##### Type locality.

“Au pont de la Save, près d’Agram” [at the bridge of the Sava river near Zagreb], Croatia.

#### † Melanopsis sublanceolata

Taxon classificationAnimaliaSorbeoconchaMelanopsidae

Kormos, 1905

##### Original source.

Kormos 1905: 393, 440, pl. 2, fig. 9.

##### Type horizon.

Late Pleistocene–early Holocene.

##### Type locality.

“Püspökfürdő” [Băile 1 Mai, Lake Pețea], Romania.

##### Remarks.

Neubauer et al. (2014d: 125) considered this taxon as a junior synonym of *Microcolpia parreyssii* (Philippi, 1847).

#### † Melanopsis sublongata

Taxon classificationAnimaliaSorbeoconchaMelanopsidae

Pallary, 1916

##### Original source.

Pallary 1916: 82.

##### Type horizon.

Lutetian, Eocene.

##### Type locality.

“Au Nord d’Albas” (Doncieux 1908: 202), France.

##### Remarks.

Replacement name for *Melanopsis elongata* Doncieux, 1908, non Férussac, 1822 (see Note 1). Wenz (1929: 2838) gave it as “*subelongata*”, which is an incorrect subsequent spelling.

#### Melanopsis costata var. subnodata

Taxon classificationAnimaliaSorbeoconchaMelanopsidae

Pallary, 1939

##### Original source.

Pallary 1939: 90, pl. 5, figs 30–32.

##### Type locality.

“Djishr ech Chegour” [Jisr Ash-Shughur], Syria.

##### Remarks.

Heller and Sivan (2002a: 49) considered the taxon as a junior synonym of *Melanopsis multiformis* Blanckenhorn, 1897. Heller et al. (2005: 244) in turn considered the species as a junior synonym of *Melanopsis costata* (Olivier, 1804).

#### Melanopsis iraqensis var. subplicata

Taxon classificationAnimaliaSorbeoconchaMelanopsidae

Pallary, 1939

##### Original source.

Pallary 1939: 89.

##### Type locality.

Not explicitly stated but probably the same as for the species (“Tappah, à 3 km Est de Belad Sindjar et ‘Ain Haglan” [Tappah, 3 km east of Sinjar and Ain Haglan (?)], Iraq).

#### † Melanopsis praemorsa f. subpraemorsa

Taxon classificationAnimaliaSorbeoconchaMelanopsidae

Bogachev, 1908

##### Original source.

Bogachev 1908: 246, 257, pl. 4, figs 8–13.

##### Type horizon.

Late Sarmatian, Khersonian, late Miocene.

##### Type locality.

“Chatma, district de Signakh, gouv. de Tiflis” [Chatma, district of Sighnaghi], Georgia.

##### Remarks.

Introduced originally as “*sub-praemorsa*”.

#### † Melanopsis subpraerosa

Taxon classificationAnimaliaSorbeoconchaMelanopsidae

Andrusov, 1909

##### Original source.

Andrusov 1909: 88, 160, pl. 5, figs 3–4.

##### Type horizon.

Pontian (sensu stricto), late Miocene.

##### Type locality.

“Adjipirdariaki, O. von Marasy, Tscharagan, [...] Sundi, [...] Chinasty” [Mount Adji-pirdariaki, east of Marazy, Çarxan, Syundi, Chinasty canyon], Azerbaijan.

#### † Melanopsis subpyrum

Taxon classificationAnimaliaSorbeoconchaMelanopsidae

Penecke, 1884

##### Original source.

Penecke 1884: 24, pl. 10, figs 19–20.

##### Type horizon.

Cernikian, Pliocene.

##### Type locality.

“Capla-Graben” [Čaplja trench near Slavonski Brod], Croatia.

#### † Melania subrugosa

Taxon classificationAnimaliaSorbeoconchaMelanopsidae

d’Orbigny, 1850 [invalid]

##### Original source.

d’Orbigny 1850: 300.

##### Type horizon.

Early Campanian, Cretaceous.

##### Type locality.

“Martigues” (Matheron 1842: 293), France.

##### Remarks.

D’Orbigny (1850) considered *Melanopsis rugosa* Matheron, 1842 a *Melania* and the species name a secondary homonym of *Melania rugosa* Lea, 1842. However, Matheron’s species was published earlier (August 1842 vs. November/December 1842; for details on publication dates of Matheron’s work see note at the beginning of the Reference section). *Melania subrugosa* is thus a junior objective synonym of *Melanopsis rugosa* Matheron, 1842.

#### Melanopsis subscalaris

Taxon classificationAnimaliaSorbeoconchaMelanopsidae

Bourguignat, 1884

##### Original source.

Bourguignat 1884: 108.

##### Type locality.

“Aïn-Fekan, source d’eau chaude entre Mascara et Saïda; dans Oued-M’Ilouya, sur la frontière du Maroc” (Bourguignat 1864: 260, 262) [‘Aïn Fekan, hot spring between Mascara and Saïda; river Moulouya, at the border to Morocco], Algeria.

##### Remarks.

Introduced for *Melanopsis maroccana scalaris* sensu Bourguignat, 1864, non Gassies, 1856 (see Note 1).

#### † Boistelia substricturata

Taxon classificationAnimaliaSorbeoconchaMelanopsidae

Jekelius, 1944

##### Original source.

Jekelius 1944: 136, pl. 55, figs 15–19.

##### Type horizon.

Early Pannonian, late Miocene.

##### Type locality.

“Turislav-Tal bei Soceni” [Turislav valley near Soceni], Romania.

#### Melanopsis subtilis

Taxon classificationAnimaliaSorbeoconchaMelanopsidae

Brot, 1878

##### Original source.

Brot 1874–1879: 373, pl. 38, fig. 5d.

##### Type locality.

“Perna Fluss in Ungarn” [not found], Hungary?

##### Remarks.

Described and illustrated in synonymy of “*Hemisinus Esperi*” [now in *Esperiana*], based on a manuscript name by Parreyss. Treated as a valid name by Clessin (1890) and made thereby available (see Note 2).

#### † Melanopsis subtilis

Taxon classificationAnimaliaSorbeoconchaMelanopsidae

Pallary, 1920 [invalid]

##### Original source.

Pallary 1920b: 110.

##### Type horizon.

Transdanubian, Pannonian, late Miocene.

##### Type locality.

“Tihany” (Fuchs 1870: 533), Hungary.

##### Remarks.

Introduced for *Melanopsis aquensis* sensu Fuchs, 1870, non Grateloup, 1838. Junior homonym of *Melanopsis subtilis* Brot, 1879. That name was introduced in synonymy but became available from Clessin (1890), who treated it as valid species-group name (see Note 2). Wenz (1929: 2804) considered the taxon as a junior synonym of *Melanopsis petrovici* Brusina, 1893.

#### † Melanopsis gorceixi var. subtilis

Taxon classificationAnimaliaSorbeoconchaMelanopsidae

Magrograssi, 1928 [invalid]

##### Original source.

Magrograssi 1928: 259, pl. 6, fig. 13.

##### Type horizon.

Plio-Pleistocene.

##### Type locality.

“Coo: molto frequente in tutte e due le zone fossilifere” [Kos island: very common in both areas rich in fossils, i.e., between Antimáchei and Pýli and in the northeast of the island, near Ágios Fokás], Greece.

##### Remarks.

Junior homonym of *Melanopsis subtilis* Brot, 1879, which was originally introduced in synonymy but made available by Clessin (1890) who treated it as a valid name (see Note 2).

#### Melanopsis subtingitana

Taxon classificationAnimaliaSorbeoconchaMelanopsidae

Annandale, 1918

##### Original source.

Annandale 1918: 163, pl. 20, figs 1–2.

##### Type locality.

“Basra”, Iraq.

##### Types.

Indian Museum, Calcutta, coll. no. 11390/2M.

##### Remarks.

Annandale attributed the authority to Nevill, apparently based on a manuscript of that author.

#### † Melanopsis subtuberculata

Taxon classificationAnimaliaSorbeoconchaMelanopsidae

Pallary, 1916

##### Original source.

Pallary 1916: 78.

##### Type horizon.

Late Villafranchian, Pleistocene.

##### Type locality.

“D’Italie” (Férussac 1823 had given the locality more precisely as “entre St.-Germini et Carsoli, et entre Otricoli et le Vigne” [between San Gemini and Carsoli, and between Otricoli and Le Vigne, Italy]).

##### Remarks.

The name “*Melanopsis subtuberculata* Férussac” has been used as valid name by several authors (e.g., Wenz 1929: 2839), but was obviously not intended as species-group name by Férussac (1823). Esu and Girotti (1975: 251) considered the taxon as a junior synonym of “*Melanopsis affinis* Férussac, 1823”, which is not available either. The name *subtuberculata* became nevertheless available from Pallary (1916) who treated it as valid species-group name and associated it with an illustration in Férussac (1823: pl. 7, fig. 11). See introduction for detailed discussion about the names used by Férussac (1823).

#### Melanopsis subturrita

Taxon classificationAnimaliaSorbeoconchaMelanopsidae

Azpeitia Moros, 1929

##### Original source.

Azpeitia Moros 1929: 192, pl. 4, figs 91–93, 95, pl. 5, figs 116, 118–120.

##### Type locality.

“Alhama de Aragón, [...] principalmente en la fuente termal de las Dehesillas” [Alhama de Aragón, mainly in the hot spring of Dehesillas], Spain.

#### † Melanopsis subulata

Taxon classificationAnimaliaSorbeoconchaMelanopsidae

Sowerby, 1822

##### Original source.

Sowerby 1821–1823: 36, pl. 332, fig. 8.

##### Type horizon.

Eocene?

##### Type locality.

“Isle of Wight”, United Kingdom.

##### Remarks.

Originally the gender was indicated as masculine (“*subulatus*”), but *Melanopsis* is feminine, which is why the name must be “*subulata*”.

#### † Melanopsis dufourii var. subulata

Taxon classificationAnimaliaSorbeoconchaMelanopsidae

Grateloup, 1838 [invalid]

##### Original source.

Grateloup 1838: 142, pl. 4, fig. 51.

##### Type horizon.

Burdigalian, early Miocene.

##### Type locality.

“Dax. Mandillot, à Saint-Paul”, France.

##### Remarks.

Junior homonym of *Melanopsis subulata* Sowerby, 1822.

#### † Melanopsis buccinoidea var. subventricosa

Taxon classificationAnimaliaSorbeoconchaMelanopsidae

Grateloup, 1828

##### Original source.

Grateloup 1828: 137.

##### Type horizon.

Burdigalian, early Miocene.

##### Type locality.

“Mandillot”, France.

#### † Melanopsis suskalovici

Taxon classificationAnimaliaSorbeoconchaMelanopsidae

Pavlović, 1903

##### Original source.

Pavlović 1903: 156, pl. 3, figs1–2.

##### Type horizon.

Middle Miocene.

##### Type locality.

“Из Бабиног Дола близу Скопља” (p. 155) [Babin Dol near Skopje], Macedonia.

##### Types.

The illustrated syntype is stored in the Natural History Museum, Belgrade, coll. no. 1447 (Milošević 1962: 24).

#### † Melanopsis synaniae

Taxon classificationAnimaliaSorbeoconchaMelanopsidae

Esu & Girotti, 2015

##### Original source.

Esu and Girotti 2015a: 68, figs 5–8.

##### Type horizon.

Synania Formation, Pleistocene.

##### Type locality.

“S-SW of Neos Erineos” [near Aigio], Greece.

##### Types.

Senckenberg Forschungsinstitut und Naturmuseum Frankfurt, coll. no. SMF 345712.

#### † Melanopsis szontaghi

Taxon classificationAnimaliaSorbeoconchaMelanopsidae

Kormos, 1905

##### Original source.

Kormos 1905: 392, 439, pl. 2, fig. 2.

##### Type horizon.

Late Pleistocene–early Holocene.

##### Type locality.

“Püspökfürdő” [Băile 1 Mai, Lake Pețea], Romania.

##### Remarks.

Neubauer et al. (2014d: 125) considered this taxon as a junior synonym of *Microcolpia parreyssii* (Philippi, 1847).

#### † Melanopsis tabulata

Taxon classificationAnimaliaSorbeoconchaMelanopsidae

Hörnes, 1856

##### Original source.

Hörnes 1851–1856: 600, pl. 49, fig. 15.

##### Type horizon.

Badenian, middle Miocene.

##### Type locality.

“Grund”, Austria.

##### Remarks.

After Cossmann (1909: 152) this species belongs in the genus *Semisinus* P. Fischer, 1885, which is an unjustified emendation of *Hemisinus* Swainson, 1840 (Thiaridae).

#### “ Melanopsis tachitensis

Taxon classificationAnimaliaSorbeoconchaMelanopsidae

Fér.” mentioned in De Cristofori and Jan (1832: Conchylia terrestria et fluviatilia, p. 7) [unavailable]

##### Locality.

“Austr.” [Australia].

##### Remarks.

Nomen nudum, found only in the species list of De Cristofori and Jan (1832).

#### Melanopsis tanousi

Taxon classificationAnimaliaSorbeoconchaMelanopsidae

Bourguignat, 1884

##### Original source.

Bourguignat 1884: 137.

##### Type locality.

“Dans les cours d’eau de la plaine du Bahr-el-Houlé, non loin d’Ain-el-Mellaha” [in the rivers of the plains of the Hula valley, near Aïn Mallahah], Israel.

##### Remarks.

Bourguignat (1884) denoted the authority as “Letourneux, 1882”, but there is no evidence that the description really derived from that author. Heller et al. (2005: 244) considered the species as a junior synonym of *Melanopsis lampra* Bourguignat, 1884.

#### † Melanopsis clava var. taurinensis

Taxon classificationAnimaliaSorbeoconchaMelanopsidae

Sacco, 1889 [invalid]

##### Original source.

Sacco 1889: 67, pl. 2, fig. 9.

##### Type horizon.

Late Burdigalian, early Miocene.

##### Type locality.

“Colline torinesi” [Torino hills], Italy.

##### Remarks.

Homonym of the simultaneously described *Melanopsis taurinensis* Sacco, 1889. Wenz (1928b) invalidly selected the variety as valid and introduced a replacement name for the species, but the taxon of higher rank takes precedence (Art. 24.1 and 57.7). No valid replacement name exists for the variety at present.

#### † Melanopsis taurinensis

Taxon classificationAnimaliaSorbeoconchaMelanopsidae

Sacco, 1889

##### Original source.

Sacco 1889: 68, pl. 2, fig. 15.

##### Type horizon.

Late Burdigalian, early Miocene.

##### Type locality.

“Colle torinesi” [Torino hills], Italy.

##### Remarks.

Homonym of the simultaneously described *Melanopsis clavataurinensis* Sacco, 1889. Wenz (1928b) invalidly selected the subspecies as valid and introduced *Melanopsis subcallosa* as replacement name for this species (junior objective synonym; see Art. 24.1 and 57.7).

#### † Balanocochlis patula var. taurostriata

Taxon classificationAnimaliaSorbeoconchaMelanopsidae

Sacco, 1895

##### Original source.

Sacco 1895: 6, pl. 1, fig. 9.

##### Type horizon.

Late Burdigalian, early Miocene.

##### Type locality.

“Colli torinese” [Torino hills], Italy.

##### Remarks.

Considered to belong in the genus *Amphimelania* by Wenz (1929: 2876). Note that Wenz gave the name as “*taurostricta*”, which is an incorrect subsequent spelling.

#### † Melanopsis tchernovi

Taxon classificationAnimaliaSorbeoconchaMelanopsidae

Heller & Sivan, 2002

##### Original source.

Heller and Sivan 2002b: 619, fig. 3F.

##### Type horizon.

Early Pleistocene.

##### Type locality.

“‘Erq el-Ahmar” [locality also known as Gesher], Israel.

##### Types.

Paleontology Collection of the Hebrew University of Jerusalem, coll. no. HUJ 9016.

#### † Melanopsis tenuiplicata

Taxon classificationAnimaliaSorbeoconchaMelanopsidae

Neumayr, 1880

##### Original source.

Neumayr 1880c: 477, pl. 7, fig. 4.

##### Type horizon.

Late Burdigalian–early Langhian, early–middle Miocene.

##### Type locality.

“Seonica”, Bosnia and Herzegovina.

#### † Melanopsis vidovici var. tenuis

Taxon classificationAnimaliaSorbeoconchaMelanopsidae

Brusina, 1903 [invalid]

##### Original source.

Brusina 1903: 114.

##### Type horizon.

Late Pleistocene–early Holocene.

##### Type locality.

“Bischofsbad” [Püspökfürdő, Băile 1 Mai, Lake Pețea], Romania.

##### Remarks.

Junior objective synonym of *Melanopsis vidovici*: Brusina (1903) indicated it as the typical form of the species. Neubauer et al. (2014d: 125) considered this taxon as a junior synonym of *Microcolpia parreyssii sikorai* (Brusina, 1903).

#### † Melanopsis tessellata

Taxon classificationAnimaliaSorbeoconchaMelanopsidae

Brusina, 1902

##### Original source.

Brusina 1902: pl. 6, figs 22–25.

##### Type horizon.

Transdanubian, Pannonian, late Miocene.

##### Type locality.

“Radmanest” [Rădmănești], Romania.

##### Types.

The illustrated syntypes are stored in the Croatian Natural History Museum, Zagreb, coll. no. 2512-158/1-2 (Milan et al. 1974: 98).

##### Remarks.

The name “*tesselata*” as mentioned in Wenz (1929: 2841) is an incorrect subsequent spelling.

#### † Melanopsis (Homalia) textilis

Taxon classificationAnimaliaSorbeoconchaMelanopsidae

Handmann, 1887

##### Original source.

Handmann 1887: 15, pl. 1, figs 12–14.

##### Type horizon.

Pannonian, zone B–D, late Miocene.

##### Type locality.

“Leobersdorf”, Austria.

##### Remarks.

Wenz (1929: 2758) considered the taxon as a junior synonym of *Melanopsis inermis* Handmann, 1882.

#### † Melanopsis themaki

Taxon classificationAnimaliaSorbeoconchaMelanopsidae

Brusina, 1903

##### Original source.

Brusina 1903: 110.

##### Type horizon.

Late Pleistocene–Holocene.

##### Type locality.

“Bischofsbad” [Püspökfürdő, Băile 1 Mai, Lake Pețea], Romania.

##### Remarks.

Neubauer et al. (2014d: 125) considered this taxon as a junior synonym of *Microcolpia parreyssii sikorai* (Brusina, 1903).

#### Hemisinus thermalis

Taxon classificationAnimaliaSorbeoconchaMelanopsidae

Brot, 1868

##### Original source.

Brot 1868: 52, pl. 3, figs 14–15.

##### Type locality.

“Carpazi [...], Miskolz [...]” [Carpazi (Italy); Miskolcz (Hungary)].

##### Remarks.

Brot attributed the authority to “Titius (?) (Parreyss)”, probably based on an “in schedis” determination.

#### † Melanopsis thomasi

Taxon classificationAnimaliaSorbeoconchaMelanopsidae

Tournouër, 1877

##### Original source.

Tournouër 1877: 275.

##### Type horizon.

Late Pannonian, late Miocene.

##### Type locality.

“Smendou” [Zighoud Youcef], Algeria.

#### Melanopsis tiaraeformis

Taxon classificationAnimaliaSorbeoconchaMelanopsidae

Pallary, 1936

##### Original source.

Pallary 1936: 60, pl. 3, fig. 2.

##### Type locality.

“Cascade d’Imouzer d’Agadir” [waterfall of Imouzzer at Agadir], Morocco.

#### “ Melanopsis tigris

Taxon classificationAnimaliaSorbeoconchaMelanopsidae

Féruss.” mentioned in Adams and Adams (1853–1858: 310) [unavailable]

##### Locality.

Not indicated.

##### Remarks.

Nomen nudum, appears only in Adams and Adams (1854) without explanation.

#### † Melanopsis tihanyensis

Taxon classificationAnimaliaSorbeoconchaMelanopsidae

Wenz, 1928

##### Original source.

Wenz 1928b: 219.

##### Type horizon.

Transdanubian, Pannonian, late Miocene.

##### Type locality.

“Tihany” (Fuchs 1870: 533), Hungary.

##### Remarks.

Replacement name for the junior homonym *Melanopsis gradata* Fuchs, 1870. Both taxa were listed as junior synonyms of *Melanopsis brusinai* Lörenthey, 1902 by Gillet and Marinescu (1971: 57).

#### † Melanopsis timacensis

Taxon classificationAnimaliaSorbeoconchaMelanopsidae

Živković, 1893

##### Original source.

Živković 1893: 166.

##### Type horizon.

Badenian, middle Miocene.

##### Type locality.

“Vražogrnac unterhalb des Einflusses des Alapin in den Timok” [Vražogrnac, below confluence of the rivers Alapin and Timok], Serbia.

#### Melanopsis tingitana

Taxon classificationAnimaliaSorbeoconchaMelanopsidae

Morelet, 1864

##### Original source.

Morelet 1864: 155.

##### Type locality.

“In Marocco” [no locality indicated].

#### † Melanopsis tinnyensis

Taxon classificationAnimaliaSorbeoconchaMelanopsidae

Wenz, 1919

##### Original source.

Wenz 1919a: 73.

##### Type horizon.

Middle Pannonian, late Miocene.

##### Type locality.

“Tinnye” (Lörenthey 1902: 214), Hungary.

##### Remarks.

Replacement name for *Melanopsis sinzowi* Lörenthey, 1902, non Brusina, 1885. Papp (1953b: 145) considered the taxon as a junior synonym of *Melanopsis rarispina* Lörenthey, 1902. However, Papp (1953b) gave the older names *Melanopsis bouei spinosa* Handmann, 1882 and *Melanopsis bouei ventricosa* Handmann, 1882 in synonymy of *Melanopsis rarispina*. While *ventricosa* Handmann is a homonym of *Melanopsis ventricosa* Neumayr, 1880 and thus invalid, the name *spinosa* is available and has priority over *rarispina*.

#### † Melanopsis parkinsoni var. titestiensis

Taxon classificationAnimaliaSorbeoconchaMelanopsidae

Popescu-Voitești, 1910

##### Original source.

Popescu-Voitești 1910: 362, pl. 21 (4), fig. 5.

##### Type horizon.

Lutetian, Eocene.

##### Type locality.

“Gropile Vulpilor près Titești” [Gropile Vulpilor (?) near Titești], Romania.

##### Remarks.

Wenz (1929: 2568) ranked the variety as a subspecies of *Coptostylus albidus* (Lamarck, 1822) (Thiaridae).

#### † “ Melanopsis toriensis

Taxon classificationAnimaliaSorbeoconchaMelanopsidae

” mentioned in Bogachev (1938: 13, 33, 53) [unavailable]

##### Horizon.

Miocene.

##### Locality.

“Tori bei Borshomi” [Tori near Borjomi], Georgia.

##### Remarks.

The name was only mentioned in a species list by Bogachev without description or illustration.

#### † Melanopsis oblonga var. torosa

Taxon classificationAnimaliaSorbeoconchaMelanopsidae

Blanckenhorn in Blanckenhorn & Oppenheim, 1927

##### Original source.

Blanckenhorn and Oppenheim 1927: 355, pl. 21, fig. 16.

##### Type horizon.

Plio-Pleistocene.

##### Type locality.

“Aus der pliocänen Dreissensiaschicht von Djisr esch-Schughr” [from the pliocene *Dreissena* layer at Jisr Ash-Shughur], Syria.

#### Melanopsis torquilla

Taxon classificationAnimaliaSorbeoconchaMelanopsidae

Pallary, 1920

##### Original source.

Pallary 1920a: 32.

##### Type locality.

“Ras el Mà de Fès” [Ras el Ma near Fes], Morocco.

#### † Melanopsis tortispina

Taxon classificationAnimaliaSorbeoconchaMelanopsidae

Papp, 1953

##### Original source.

Papp 1953b: 147, pl. 12, figs 18–20.

##### Type horizon.

Pannonian, zone F–G, late Miocene.

##### Type locality.

“Moosbrunn, N.-Ö.”, Austria.

##### Remarks.

Appeared first as nomen nudum (as *Melanopsis bouei tortispina*) in Papp (1951: 167).

#### † Melanopsis tothi

Taxon classificationAnimaliaSorbeoconchaMelanopsidae

Brusina, 1903

##### Original source.

Brusina 1903: 114.

##### Type horizon.

Late Pleistocene–Holocene.

##### Type locality.

“Bischofsbad” [Püspökfürdő, Băile 1 Mai, Lake Pețea], Romania.

##### Remarks.

Neubauer et al. (2014d: 125) considered this taxon as a junior synonym of *Microcolpia parreyssii sikorai* (Brusina, 1903).

#### † Melanopsis tournoueri

Taxon classificationAnimaliaSorbeoconchaMelanopsidae

Pallary, 1920

##### Original source.

Pallary 1920b: 112.

##### Type horizon.

Tafi Formation, early Pleistocene.

##### Type locality.

“Antimaki” (Tournouër 1876: 449) [Antimácheia, Kos Island], Greece.

##### Remarks.

Introduced for *Melanopsis cariosa* sensu Tournouër, 1876, non Linnaeus, 1758.

#### Melanopsis transcaspia

Taxon classificationAnimaliaSorbeoconchaMelanopsidae

Izzatullaev & Starobogatov, 1984

##### Original source.

Izzatullaev and Starobogatov 1984: 1478, fig. 1 (9).

##### Type locality.

“Ручей Гяуре, колхоз им. 1 Мая Ашхабадского р-на” [Gyaure creek, Ashgabat district], Turkmenistan.

##### Types.

Zoological Institute of Russian Academy of Sciences, St.-Petersburg; no number indicated.

#### † Melanopsis transiens

Taxon classificationAnimaliaSorbeoconchaMelanopsidae

Blanckenhorn, 1897

##### Original source.

Blanckenhorn 1897: 135, pl. 10, fig. 19.

##### Type horizon.

Plio-Pleistocene.

##### Type locality.

“In der Dreissensiaschicht von Dschisr esch-Schurr” [in the *Dreissena* layers at Jisr Ash-Shughur], Syria.

#### Melanopsis nodosa var. transiens

Taxon classificationAnimaliaSorbeoconchaMelanopsidae

Cerulli-Irelli, 1914 [invalid] 

##### Original source.

Cerulli-Irelli 1914: 185, pl. 15 (47), figs 8–9.

##### Type locality.

“M. Mario: Farnesina”, Italy.

##### Remarks.

Junior homonym of *Melanopsis transiens* Blanckenhorn, 1897. Pallary (1920b) introduced *Melanopsis cerullii* as replacement name. Girotti (1972: 232) considered the taxon as a junior synonym of “*Melanopsis affinis* Férussac”, which is, however, not an available name.

#### † Melanopsis recurrens var. transitans

Taxon classificationAnimaliaSorbeoconchaMelanopsidae

Brusina, 1874

##### Original source.

Brusina 1874: 42.

##### Type horizon.

Cernikian, Pliocene.

##### Type locality.

“Bečić; Podvinje (Čaplja) [Čaplja trench near Slavonski Brod]; Novska; Kovačevac; Moslavina”, Croatia.

##### Types.

The illustrated syntypes are stored in the Croatian Natural History Museum, Zagreb, coll. no. 2520-166/1-2 (Milan et al. 1974: 98).

#### † Melanopsis trempensis

Taxon classificationAnimaliaSorbeoconchaMelanopsidae

Bandel, 2000

##### Original source.

Bandel 2000: 182, figs 143–145.

##### Type horizon.

Valcarga Formation, Campanian, Cretaceous.

##### Type locality.

“Pumanous slump exposed near Torallola”, Spain.

##### Types.

Geological-Palaeontological Institute and Museum University of Hamburg, coll. no. 4270.

#### Buccinum tricarinatum

Taxon classificationAnimaliaSorbeoconchaMelanopsidae

Bruguière, 1789

##### Original source.

Bruguière 1789: 280.

##### Type locality.

Not indicated.

##### Remarks.

Commonly combined as *Melanopsis tricarinata* or as *Melanopsis dufourii tricarinata* (e.g., Azpeitia Moros 1929, Glaubrecht 1996, Ahuir Galindo 2015).

#### † Melanopsis tricarinata

Taxon classificationAnimaliaSorbeoconchaMelanopsidae

Sowerby in Fitton, 1836 [invalid]

##### Original source.

Fitton 1836: 228, 346, pl. 22, fig. 4.

##### Type horizon.

Weald Clay, early Cretaceous.

##### Type locality.

“Punfield”, United Kingdom.

##### Remarks.

Appeared first as nomen nudum (as “*Melania tricarinata*”) in Fitton (1824: 376). Junior secondary homonym of *Melanopsis tricarinata* (Bruguière, 1789). Sandberger (1870: 55) considered this species as a junior synonym of *Pleurocera strombiforme* (Schlotheim, 1820) (Pleuroceridae).

#### Melanopsis trifasciata

Taxon classificationAnimaliaSorbeoconchaMelanopsidae

Gray in Dieffenbach, 1843

##### Original source.

Gray in Dieffenbach 1843: 263.

##### Type locality.

“New Zealand, Bay of Islands, Waitanga Falls”, New Zealand.

##### Remarks.

Originally the gender was indicated as masculine (“*trifasciatus*”), but *Melanopsis* is feminine, which is why the name must be “*trifasciata*”. Type species of *Zemelanopsis* Finlay, 1926. The name “*bifasciata*” as mentioned in Pallary (1926a: 76) is an incorrect subsequent spelling.

#### † “ Melanopsis themaki var. trifilosa

Taxon classificationAnimaliaSorbeoconchaMelanopsidae

” mentioned in Brusina (1903: 110) [unavailable]

##### Horizon.

Late Pleistocene–early Holocene.

##### Locality.

“Bischofsbad” [Püspökfürdő, Băile 1 Mai, Lake Pețea], Romania.

##### Remarks.

Nomen nudum (Brusina apparently considered the term self-explanatory). Neubauer et al. (2014d: 125) considered this taxon as a junior synonym of *Microcolpia parreyssii sikorai* (Brusina, 1903).

#### † “ Melanopsis tothi var. trifilosa

Taxon classificationAnimaliaSorbeoconchaMelanopsidae

” mentioned in Brusina (1903: 114) [unavailable]

##### Horizon.

Late Pleistocene–early Holocene.

##### Locality.

“Bischofsbad” [Püspökfürdő, Băile 1 Mai, Lake Pețea], Romania.

##### Remarks.

Nomen nudum (Brusina apparently considered the term self-explanatory). Brusina (1903) introduced several varieties with this name, apparently considering it only a descriptive term. Neubauer et al. (2014d: 125) considered this taxon as a junior synonym of *Microcolpia parreyssii sikorai* (Brusina, 1903).

#### † Melanopsis tripaloi

Taxon classificationAnimaliaSorbeoconchaMelanopsidae

Bourguignat, 1880

##### Original source.

Bourguignat 1880: 35.

##### Type horizon.

Langhian, middle Miocene.

##### Type locality.

“Vallée de la Cettina” [Cetina river valley], Croatia.

##### Remarks.

Wenz (1929: 2734) considered this taxon as a junior synonym of *Melanopsis geniculata* Brusina, 1874.

#### “ Melanopsis tripoliana

Taxon classificationAnimaliaSorbeoconchaMelanopsidae

” mentioned in Brot (1874–1879: 419) [unavailable]

##### Locality.

Not indicated.

##### Remarks.

Nomen nudum, “in schedis” name from Tarnier listed in synonymy of *Melania buccinoidea* (Olivier, 1801) by Brot (1879).

#### † Melanopsis boueitrispina

Taxon classificationAnimaliaSorbeoconchaMelanopsidae

Pashko, 1968

##### Original source.

Pashko 1968: 137, 151, pl. 2, figs 1–8.

##### Type horizon.

Tortonian, late Miocene.

##### Type locality.

“Suita e Burrelit, prerja e Zallit të Gërmanit” [at Burrel, outcrop at the river Zalli të Germanit], Albania.

#### † Melanopsis trivortiana

Taxon classificationAnimaliaSorbeoconchaMelanopsidae

Locard, 1883

##### Original source.

Locard 1883b: 59, pl. 3, fig. 5.

##### Type horizon.

Mammal zone MN 14, Pliocene.

##### Type locality.

“Trévoux” (p. 53), France.

#### † Melanopsis defensa var. trochiformis

Taxon classificationAnimaliaSorbeoconchaMelanopsidae

Fuchs, 1870

##### Original source.

Fuchs 1870a: 354, pl. 14, figs 77–78.

##### Type horizon.

Transdanubian, Pannonian, late Miocene.

##### Type locality.

“Radmanest” [Rădmănești], Romania.

#### † Melanopsis trojana

Taxon classificationAnimaliaSorbeoconchaMelanopsidae

Hoernes, 1876

##### Original source.

Hoernes 1876: 18, figs 8–15.

##### Type horizon.

Late Sarmatian, Khersonian, late Miocene.

##### Type locality.

“Renkiöi” [north of İntepe], Turkey.

#### † Melanopsis trstenjaki

Taxon classificationAnimaliaSorbeoconchaMelanopsidae

Brusina, 1884

##### Original source.

Brusina 1884b: 55.

##### Type horizon.

Early Langhian, middle Miocene.

##### Type locality.

“Potravlje” (p. 47), Croatia.

##### Types.

Milan et al. (1974: 98) indicated a holotype, but it is uncertain whether the specimen was the only one Brusina had at hand (holotype by monotypy, Art. 73.1.2). The specimen is stored in the Croatian Natural History Museum, Zagreb, coll. no. 2973-619.

#### “ Melanopsistruncata

Taxon classificationAnimaliaSorbeoconchaMelanopsidae

” mentioned in De Cristofori and Jan (1832: Conchylia terrestria et fluviatilia, p. 7) [unavailable]

##### Locality.

“Am. mer.” [South America].

##### Remarks.

Nomen nudum, only mentioned in a species list by De Cristofori and Jan (1832).

#### Amphimelania (Melania) holandri var. tschapecki

Taxon classificationAnimaliaSorbeoconchaMelanopsidae

Westerlund, 1886

##### Original source.

Westerlund 1886: 105.

##### Type locality.

“Uferstellen der Sann bei Römerbad” (Tschapeck 1881: 102) [riversides of the Savinja river near Rimske Toplice], Slovenia.

##### Remarks.

Introduced for a specimen of *Melania holandri* illustrated by Tschapeck (1881: 102, pl. 5, fig. d).

#### † Melanopsis (Melanoptychia) tuberculata

Taxon classificationAnimaliaSorbeoconchaMelanopsidae

Pavlović, 1927

##### Original source.

Pavlović 1927: 60, pl. 7, figs 1–2.

##### Type horizon.

Middle Pannonian, late Miocene.

##### Type locality.

“У врчинском хатару (долини потока Карагача)” [from Karagača river valley near Vrčin], Serbia.

##### Types.

Perhaps stored in the Natural History Museum, Belgrade, but not mentioned in the catalogue of Milošević (1962).

#### Melanopsis tuberculata

Taxon classificationAnimaliaSorbeoconchaMelanopsidae

Yen, 1939 [invalid]

##### Original source.

Yen 1939: 58, pl. 5, fig. 10.

##### Type locality.

“See von Ta-li-fu (Yünnan)” [Lake Er Hai, Yunnan province], China.

##### Remarks.

Junior homonym of *Melanopsis tuberculata* Pavlović, 1927. Probably not a Melanopsidae.

#### † Melanopsis flammulata var. tuberosa

Taxon classificationAnimaliaSorbeoconchaMelanopsidae

De Stefani, 1880

##### Original source.

De Stefani 1880: 49, pl. 3, fig. 14.

##### Type horizon.

Late Villafranchian, Pleistocene.

##### Type locality.

“Monticiano”, Italy.

##### Remarks.

Esu and Girotti (1975: 251) considered the taxon as a junior synonym of “*Melanopsis affinis* Férussac”, which is not an available name.

#### “ Melanopsis tumida

Taxon classificationAnimaliaSorbeoconchaMelanopsidae

” mentioned in Brot (1874–1879: 420, pl. 45, fig. 3) [unavailable]

##### Locality.

“Taurus-Gebiete” [Taurus region], Turkey.

##### Remarks.

Introduced in synonymy of *Melania buccinoidea* by Brot (1879), referring to an “in schedis” name by Parreyss (see Note 2).

#### Melanopsis tumida

Taxon classificationAnimaliaSorbeoconchaMelanopsidae

Pallary, 1916

##### Original source.

Pallary 1916: 86.

##### Type locality.

Stated to be uncertain by Philippi (1887, p. 40): “vermutlich bei Lebu oder Tubul gefunden” [probably found near Lebu or Tubul, Región del Biobío], Chile.

##### Remarks.

Pallary (1916) introduced this replacement for the presumed secondary homonym *Melanopsis obesa* (Philippi, 1887), non Gassies, 1856. Despite a valid nomenclatural act, the name is hardly useful because Philippi’s species is certainly no Melanopsidae.

#### Melanopsis tunetana

Taxon classificationAnimaliaSorbeoconchaMelanopsidae

Morlet, 1881

##### Original source.

Morlet 1881: 396, pl. 6, figs 3–4.

##### Type locality.

“Tozeur, Kriz”, Tunisia.

##### Remarks.

The name “*tuneata*” as mentioned in Chevallier (1969: 286) is an incorrect subsequent spelling.

#### Melanopsis praemorsa f. turbinata

Taxon classificationAnimaliaSorbeoconchaMelanopsidae

Pérès, 1939

##### Original source.

Pérès 1939: 134.

##### Type locality.

“Station 31. Daïa Afourgagh près d’Annosseur. Station 144. Oued Sidi Raba à 6 kilomètres environ en amont de son confluent avec le Bou-Regreg” [Station 31 at Dayat Afergagh near Anosseur; station 144 at Oued Sidi Raho, approximately 6 km upstream of its confluence with the Bou Regreg], Morocco.

#### Melanopsis costata var. turcica

Taxon classificationAnimaliaSorbeoconchaMelanopsidae

Mousson, 1874 

##### Original source.

Mousson 1874: 33.

##### Type locality.

“Dans le Karasu, affluent du lac d’Antioche (p. 33); environs de Samava (p. 49; there treated as distinct species)” [in the Karasu river, tributary of the Lake Anuk (also as Amik) (Turkey); surroundings of As Samawah (Iraq)].

##### Remarks.

Mousson attributed the authority to Parreyss, but there is no evidence that the description really derived from that author.

#### † Melanopsis turgida

Taxon classificationAnimaliaSorbeoconchaMelanopsidae

Fischer-de-Waldheim, 1837

##### Original source.

Fischer-de-Waldheim 1837: 131, pl. 18 [in text err. as “VXIII”], fig. 13.

##### Type horizon.

Jurassic?

##### Type locality.

“Miatchkova” [Myachikovo?], Russia.

##### Remarks.

Certainly not a *Melanopsis*.

#### Melanopsis turgida

Taxon classificationAnimaliaSorbeoconchaMelanopsidae

Brot, 1878 [invalid]

##### Original source.

Brot 1874–1879: 373, pl. 38, fig. 5c.

##### Type locality.

“Glina Fluss in Ungarn” [Glina river], Croatia.

##### Remarks.

Described and illustrated in synonymy of “*Hemisinus Esperi*” [now in *Esperiana*], based on a manuscript name by Parreyss. Clessin (1890) treated it as valid name and made it available thereby (see Note 2). Junior homonym of *Melanopsis turgida* Fischer-de-Waldheim, 1837. Starobogatov et al. (1992: 60) considered the species as a junior synonym of *Fagotia* [= *Esperiana*] *esperi* (Férussac, 1823).

#### Melanopsis turgida

Taxon classificationAnimaliaSorbeoconchaMelanopsidae

Pallary, 1928 [invalid]

##### Original source.

Pallary 1928a: 259, pl. 5, figs 11–12.

##### Type locality.

“Moulaï Taïeb et Taourirt du Zâ (Maroc oriental)” [Moulay Taïeb and in Oued Za at Taourirt (?) (eastern Morocco)], Morocco.

##### Remarks.

Junior homonym of *Melanopsis turgida* Fischer-de-Waldheim, 1837.

#### † Melanopsis turislavica

Taxon classificationAnimaliaSorbeoconchaMelanopsidae

Jekelius, 1944

##### Original source.

Jekelius 1944: 131, pl. 49, figs 5–6.

##### Type horizon.

Early Pannonian, late Miocene.

##### Type locality.

“Turislav-Tal bei Soceni” [Turislav valley near Soceni], Romania.

#### Melanopsis turkmenica

Taxon classificationAnimaliaSorbeoconchaMelanopsidae

Izzatullaev & Starobogatov, 1984 

##### Original source.

Izzatullaev and Starobogatov 1984: 1478, fig. 1 (10), fig. 2 (4).

##### Type locality.

“Закаспийская область” [Transcaspian Region], Russia.

##### Types.

Zoological Institute of Russian Academy of Sciences, St.-Petersburg; no number indicated.

#### † Melanopsis turricula

Taxon classificationAnimaliaSorbeoconchaMelanopsidae

Matheron, 1842

##### Original source.

Matheron 1842: 294, pl. 37, figs 15–16.

##### Type horizon.

Early Campanian, Cretaceous.

##### Type locality.

Not stated after the description, as Matheron used to do it for the other species. Although he did not denote an exact locality, he mentioned earlier in text the occurrence of the species in deposits situated “vers Vitrolles et Martigues” (p. 148–149) [towards Vitrolles and Martigues, France].

#### Melanopsis (Mesopotamia) mesopotamica var. turricula

Taxon classificationAnimaliaSorbeoconchaMelanopsidae

Pallary, 1939 [invalid]

##### Original source.

Pallary 1939: 100, pl. 5, fig. 7.

##### Type locality.

“‘Ain Arouss” [‘Ayn al ‘Arūs, near Tall Abyaḑ], Syria.

##### Remarks.

Junior homonym of *Melanopsis turricula* Matheron, 1842.

#### † Melanopsis (Canthidomus) phanesiana var. turriculata

Taxon classificationAnimaliaSorbeoconchaMelanopsidae

Magrograssi, 1928

##### Original source.

Magrograssi 1928: 260, pl. 6, fig. 19.

##### Type horizon.

Plio-Pleistocene.

##### Type locality.

“Coo” [Kos island], Greece.

#### Melanopsis turriformis

Taxon classificationAnimaliaSorbeoconchaMelanopsidae

Picard, 1934

##### Original source.

Picard 1934: 123, pl. 8, figs 15–27.

##### Type locality.

“Jarmukmündung” [Yarmouk river mouth], Jordan/Israel.

#### Melanopsis cariosa var. turrita

Taxon classificationAnimaliaSorbeoconchaMelanopsidae

Rossmässler, 1854 [invalid]

##### Original source.

Rossmässler 1854–1859: 33, pl. 68, fig. 846.

##### Type locality.

“Im Gebiet des unteren Guadalquivir” [in the lower Guadalquivir river], Spain.

##### Remarks.

Rossmässler (1854) based this new variety entirely on *Melanopsis sevillensis* Grateloup, 1840, arguing that “Grateloup elevated this variety to species level without reason”. Thus, *Melanopsis turrita* is an objective synonym of *Melanopsis sevillensis*.

#### † Melanopsis (Canthidomus) turrita

Taxon classificationAnimaliaSorbeoconchaMelanopsidae

Handmann, 1887 [invalid]

##### Original source.

Handmann 1887: 32, pl. 7, fig. 13.

##### Type horizon.

Pannonian, zone B–D, late Miocene.

##### Type locality.

“Leobersdorf”, Austria.

##### Remarks.

Junior homonym of *Melanopsis turrita* Rossmässler, 1854. Pallary (1916: 84) introduced *Melanopsis hispidula* as replacement name.

#### † Melanoptychia turrita

Taxon classificationAnimaliaSorbeoconchaMelanopsidae

Jekelius, 1944 [invalid]

##### Original source.

Jekelius 1944: 138, pl. 57, figs 1–21.

##### Type horizon.

Early Pannonian, late Miocene.

##### Type locality.

“Turislav-Tal bei Soceni” [Turislav valley near Soceni], Romania.

##### Remarks.

Junior secondary homonym of *Melanopsis turrita* Rossmässler, 1854 and junior synonym of *Melanopsis turrita* Handmann, 1887, for which Pallary (1916: 84) had introduced *Melanopsis hispidula* as replacement name (see also Neubauer et al. 2014a: 463).

#### † Pseudofagotia turrita

Taxon classificationAnimaliaSorbeoconchaMelanopsidae

Anistratenko, 1993

##### Original source.

Anistratenko 1993: 73, textfig. 2.

##### Type horizon.

Duab Beds, middle to late Kimmerian, Pliocene.

##### Type locality.

“Окр. с. Мокви, Очамчирский р-н” [near the village Mok’vi, Ochamchirskiy rayon], Georgia.

##### Types.

Schmalhausen Institute of Zoology of National Academy of Sciences of Ukraine, Kiev; no number indicated.

#### † Melanopsis conemenosiana var. turritella

Taxon classificationAnimaliaSorbeoconchaMelanopsidae

Pallary, 1920

##### Original source.

Pallary 1920b: 112.

##### Type horizon.

Plio-Pleistocene.

##### Type locality.

“Preveza in Epirus” (Oppenheim 1891: 469), Greece.

##### Remarks.

Replacement name for *Melanopsis boettgeri* Oppenheim, 1891, non Klika, 1891.

#### Melanopsis tutulata

Taxon classificationAnimaliaSorbeoconchaMelanopsidae

Pallary, 1928

##### Original source.

Pallary 1928a: 271, pl. 5, figs 8–10.

##### Type locality.

“Berguent; Aoûllout; Ras el Mâ de Fès; O. Chkef près Fès” [Aïn Beni Mathar, Aïn Aoullout, Ras El Ma, Oued Aïn Chkef in Fes], Morocco.

#### Microcolpia ucrainica

Taxon classificationAnimaliaSorbeoconchaMelanopsidae

Starobogatov, Alexenko & Levina, 1992

##### Original source.

Starobogatov et al. 1992: 69, textfig. 3 (20).

##### Type locality.

“Из Южного Буга” [in the southern Bug river], Ukraine.

##### Types.

Zoological Institute of Russian Academy of Sciences, St.-Petersburg; no number indicated.

#### † Melanopsis themaki var. unicarinata

Taxon classificationAnimaliaSorbeoconchaMelanopsidae

Brusina, 1903

##### Original source.

Brusina 1903: 111.

##### Type horizon.

Late Pleistocene–early Holocene.

##### Type locality.

“Bischofsbad” [Püspökfürdő, Băile 1 Mai, Lake Pețea], Romania.

##### Remarks.

Neubauer et al. (2014d: 125) considered this taxon as a junior synonym of *Microcolpia parreyssii sikorai* (Brusina, 1903).

#### † Melanopsis unicincta

Taxon classificationAnimaliaSorbeoconchaMelanopsidae

Blanckenhorn, 1897

##### Original source.

Blanckenhorn 1897: 119, pl. 9, figs 41–44.

##### Type horizon.

Plio-Pleistocene.

##### Type locality.

“In der obersten Melanopsiden-Thonbank des rechten Orontesufers bei Dschisr esch-Schurr” [in the uppermost clay bank at the right riverside of the Orontes near Jisr Ash-Shughur], Syria.

##### Remarks.

Introduced as “n. mut.” but clearly as a binomen and hence not infrasubspecific in the sense of ICZN Art. 45.6.

#### † Melanopsis tothi var. unicingulata

Taxon classificationAnimaliaSorbeoconchaMelanopsidae

Brusina, 1903

##### Original source.

Brusina 1903: 114.

##### Type horizon.

Late Pleistocene–early Holocene.

##### Type locality.

“Bischofsbad” [Püspökfürdő, Băile 1 Mai, Lake Pețea], Romania.

##### Remarks.

Neubauer et al. (2014d: 125) considered this taxon as a junior synonym of *Microcolpia parreyssii sikorai* (Brusina, 1903).

#### † Melanopsis hazayi var. unifilosa

Taxon classificationAnimaliaSorbeoconchaMelanopsidae

Brusina, 1903 [unresolved]

##### Original source.

Brusina 1903: 113.

##### Type horizon.

Late Pleistocene–early Holocene.

##### Type locality.

“Bischofsbad” [Püspökfürdő, Băile 1 Mai, Lake Pețea], Romania.

##### Remarks.

Brusina (1903) introduced several varieties with this name, apparently considering it only a descriptive term; the homonymy issue needs to be solved by a First Reviser. Currently, all of them are considered junior synonyms of *Microcolpia parreyssii sikorai* (Brusina, 1903) (Neubauer et al. 2014d: 125).

#### † Melanopsis sikorai var. unifilosa

Taxon classificationAnimaliaSorbeoconchaMelanopsidae

Brusina, 1903 [unresolved]

##### Original source.

Brusina 1903: 112.

##### Type horizon.

Late Pleistocene–early Holocene.

##### Type locality.

“Bischofsbad” [Püspökfürdő, Băile 1 Mai, Lake Pețea], Romania.

##### Remarks.

Brusina (1903) introduced several varieties with this name, apparently considering it only a descriptive term; the homonymy issue needs to be solved by a First Reviser. Currently, all of them are considered junior synonyms of *Microcolpia parreyssii sikorai* (Brusina, 1903) (Neubauer et al. 2014d: 125).

#### † Melanopsis themaki var. unifilosa

Taxon classificationAnimaliaSorbeoconchaMelanopsidae

Brusina, 1903 [unresolved]

##### Original source.

Brusina 1903: 110.

##### Type horizon.

Late Pleistocene–early Holocene.

##### Type locality.

“Bischofsbad” [Püspökfürdő, Băile 1 Mai, Lake Pețea], Romania.

##### Remarks.

Brusina (1903) introduced several varieties with this name, apparently considering it only a descriptive term; the homonymy issue needs to be solved by a First Reviser. Currently, all of them are considered junior synonyms of *Microcolpia parreyssii sikorai* (Brusina, 1903) (Neubauer et al. 2014d: 125).

#### † Melanopsis tothi var. unifilosa

Taxon classificationAnimaliaSorbeoconchaMelanopsidae

Brusina, 1903 [unresolved]

##### Original source.

Brusina 1903: 114.

##### Type horizon.

Late Pleistocene–early Holocene.

##### Type locality.

“Bischofsbad” [Püspökfürdő, Băile 1 Mai, Lake Pețea], Romania.

##### Remarks.

Brusina (1903) introduced several varieties with this name, apparently considering it only a descriptive term; the homonymy issue needs to be solved by a First Reviser. Currently, all of them are considered junior synonyms of *Microcolpia parreyssii sikorai* (Brusina, 1903) (Neubauer et al. 2014d: 125).

#### † Melanopsis vacua

Taxon classificationAnimaliaSorbeoconchaMelanopsidae

Vidal, 1874

##### Original source.

Vidal 1874: 236, pl. 3, fig. 15.

##### Type horizon.

Maastrichtian, Cretaceous.

##### Type locality.

“Isona”, Spain.

#### † Melanopsis valdeci

Taxon classificationAnimaliaSorbeoconchaMelanopsidae

Brusina, 1902

##### Original source.

Brusina 1902: pl. 6, figs 18–21.

##### Type horizon.

Early Cernikian, early Pliocene.

##### Type locality.

“Čerević”, Serbia.

##### Types.

The illustrated syntypes are stored in the Croatian Natural History Museum, Zagreb, coll. no. 2511-157/1-4 (Milan et al. 1974: 98).

#### † Melanopsis kleinii var. valentinensis

Taxon classificationAnimaliaSorbeoconchaMelanopsidae

Fontannes, 1881

##### Original source.

Fontannes 1881: 1015, pl. 1, figs 7–9.

##### Type horizon.

Mammal zone MN 10–12, late Miocene.

##### Type locality.

“Montvendre”, France.

#### † Melanopsis vandeveldi

Taxon classificationAnimaliaSorbeoconchaMelanopsidae

Bukowski, 1892

##### Original source.

Bukowski 1892: 249.

##### Type horizon.

Salakos Formation, Pliocene.

##### Type locality.

“Rhodos” (locality specified as “Kalavarda” in Bukowski 1893), Greece.

#### Melanopsis vanrossomi

Taxon classificationAnimaliaSorbeoconchaMelanopsidae

Pallary, 1936

##### Original source.

Pallary 1936: 58, pl. 3, fig. 7.

##### Type locality.

“Souk el Hâd des Aït Souhab, dans l’oued Gough, et Tanalt, à quelques kilomètres au sud du précédent dans l’Anti Atlas occidental” [Souk el Hâd of Aït Souhab (?), in the Oued Gough (?), and Tanalt, a few kilometers south of the former in the western Anti-Atlas], Morocco.

#### Melania (Melanopsis) variabilis

Taxon classificationAnimaliaSorbeoconchaMelanopsidae

Von dem Busch in Philippi, 1847

##### Original source.

Philippi 1847: 175, pl. 4, figs 7–8, 10.

##### Type locality.

“Schiraz et Persepolis Persiae” [Shiraz and Persepolis, Fars Province], Iran.

#### “ Melania crassa var. variceata

Taxon classificationAnimaliaSorbeoconchaMelanopsidae

Sandri” mentioned in Brusina (1866: 106) [unavailable]

##### Locality.

“Cetina”, Croatia.

##### Remarks.

Nomen nudum, listed by Brusina without any explanation.

#### † Melanopsis varicosa

Taxon classificationAnimaliaSorbeoconchaMelanopsidae

Handmann, 1882

##### Original source.

Handmann 1882: 553.

##### Type horizon.

Pannonian, zone D, late Miocene.

##### Type locality.

“Kottingbrunn [...] Ziegelei a”, Austria.

#### Melanopsis variegata

Taxon classificationAnimaliaSorbeoconchaMelanopsidae

Morelet, 1857

##### Original source.

Morelet 1857: 33.

##### Type locality.

“[Ad Sanctam-Mariam de Balade]” [Balade], New Caledonia.

##### Remarks.

Brot (1879: 444) considered the taxon as a junior synonym of *Melanopsis frustulum* Morelet, 1857.

#### “ Melania holandri var. variegata

Taxon classificationAnimaliaSorbeoconchaMelanopsidae

Grimmer” mentioned in Brot (1874–1879: 12) and Clessin (1890: 676) [unavailable]

##### Locality.

Not indicated.

##### Remarks.

Nomen nudum, listed in synonymy of “*Melania holandri*” [sic] (Brot 1874) and “Melania holandri var. aequata” (Clessin 1890), respectively.

#### Melanopsis pseudoferussaci var. vaucheri

Taxon classificationAnimaliaSorbeoconchaMelanopsidae

Pallary, 1899

##### Original source.

Pallary 1899: 140, pl. 9, fig. 12.

##### Type locality.

“Fez” [Fes], Morocco.

#### † Melanopsis ventricosa

Taxon classificationAnimaliaSorbeoconchaMelanopsidae

Neumayr, 1880

##### Original source.

Neumayr 1880b: 290, pl. 1, fig. 9.

##### Type horizon.

Plio-Pleistocene.

##### Type locality.

“Zwischen Pylle und Antimachia” [between Pýli and Antimácheia, Kos Island], Greece.

##### Remarks.

Willmann (1981: 171) considered this taxon as a junior synonym of *Melanopsis sporadum* Tournouër, 1876.

#### † Melanopsis bouei var. ventricosa

Taxon classificationAnimaliaSorbeoconchaMelanopsidae

Handmann, 1882 [invalid]

##### Original source.

Handmann 1882: 557.

##### Type horizon.

Pannonian, zone D, late Miocene.

##### Type locality.

“Kottingbrunn [...] Ziegelei a”, Austria.

##### Remarks.

Junior homonym of *Melanopsis ventricosa* Neumayr, 1880. Wenz (1929: 2674) considered the taxon as a junior synonym of *Melanopsis bouei* Férussac, 1823.

#### “ Melanopsis dufouri var. cossoni f. ventricosior

Taxon classificationAnimaliaSorbeoconchaMelanopsidae

” mentioned in Westerlund (1886: 126) [unavailable]

##### Locality.

“Bei Valencia” [near Valencia], Spain.

##### Remarks.

Introduced as infrasubspecific taxon (forma below a variety), which is not ruled by the provisions of the Code. Moreover, the name is a nomen nudum; Westerlund apparently considered the name a descriptive term (*ventricosior* = Latin “more bulbous”).

#### Melanopsis cariosa var. ventrosa

Taxon classificationAnimaliaSorbeoconchaMelanopsidae

Bourguignat, 1884 [unresolved]

##### Original source.

Bourguignat 1884: 151.

##### Type locality.

“Dans les aqueducs de Séville et dans le Guadalquivir” [in the aqueducts of Sevilla and in the Guadalquivir river], Spain.

##### Remarks.

Homonym of the simultaneously published name *Melanopsiscostataventrosa* Bourguignat, 1884. This case requires the action of a First Reviser.

#### Melanopsis costata var. ventrosa

Taxon classificationAnimaliaSorbeoconchaMelanopsidae

Bourguignat, 1884 [unresolved]

##### Original source.

Bourguignat 1884: 141.

##### Type locality.

Not indicated, but apparently the specimens derive from somewhere along the Jordan river or the Sea of Galilee.

##### Remarks.

Homonym of the simultaneously published name *Melanopsiscariosaventrosa* Bourguignat, 1884. This case requires the action of a First Reviser.

#### † Melanopsis venusta

Taxon classificationAnimaliaSorbeoconchaMelanopsidae

Pallary, 1916

##### Original source.

Pallary 1916: 80.

##### Type horizon.

Pannonian, zone D, late Miocene.

##### Type locality.

“Kottingbrunn [...] Ziegelei a” (Handmann 1882: 557), Austria.

##### Remarks.

Replacement name for *Melanopsis nodosa* Handmann, 1882, non Férussac, 1822. Wenz (1929: 2681) considered this taxon as a junior synonym of *Melanopsis bouei* Férussac, 1823.

#### † “ Melanopsis impressa vera

Taxon classificationAnimaliaSorbeoconchaMelanopsidae

” mentioned in Schréter (1912: 155) [unavailable]

##### Horizon.

Miocene.

##### Locality.

“Boesciwiese, östlich von Kohldorf [...] in dem von Norden, vom Balomberge hinablaufenden Seitengraben” [Poiana Boistii (?), east of Cărbunari, in the trench running down from the Mt. Balom (?) in the north], Hungary.

##### Remarks.

Nomen nudum, appears without explanation in the text. Possibly, Schréter wanted to denote that it is the “real” *Melanopsis impressa* and not a variety. Wenz (1929: 2753) listed the record as a junior synonym of *Melanopsis impressa*.

#### Melanopsis vespertina

Taxon classificationAnimaliaSorbeoconchaMelanopsidae

Bourguignat, 1884

##### Original source.

Bourguignat 1884: 124.

##### Type locality.

“Lalla Maghnia, sur la frontière du Maroc” [Maghnia, at the border to Morocco], Morocco or Algeria.

#### † “ Melanopsis vetusta

Taxon classificationAnimaliaSorbeoconchaMelanopsidae

” mentioned in Sandberger (1870: 88–89) [unavailable]

##### Horizon.

Unknown.

##### Locality.

“Plan d’Aups (Var)” [Plan-d’Aups-Sainte-Baume, Dép. Var].

##### Remarks.

Nomen nudum, used by Matheron in correspondence with Sandberger (1870), who gave it as a junior synonym of Paludomus lyra var. calva (Paludomidae), which he described there as new.

#### † Melanopsis vicentina

Taxon classificationAnimaliaSorbeoconchaMelanopsidae

Oppenheim, 1890

##### Original source.

Oppenheim 1890a: 135, pl. 4, figs 1–1b.

##### Type horizon.

Ronca Beds, Bartonian, Eocene.

##### Type locality.

“Lovara di Tressino, Mussolon, Monte Pulli bei Valdagno” [Lovara, Muzzolon, Monte Pulli near Valdagno], Italy.

#### † Melanopsis vidovici

Taxon classificationAnimaliaSorbeoconchaMelanopsidae

Brusina, 1903

##### Original source.

Brusina 1903: 113.

##### Type horizon.

Late Pleistocene–Holocene.

##### Type locality.

“Bischofsbad” [Püspökfürdő, Băile 1 Mai, Lake Pețea], Romania.

##### Remarks.

Neubauer et al. (2014d: 125) considered this taxon as a junior synonym of *Microcolpia parreyssii sikorai* (Brusina, 1903).

#### † Melanopsis vilicici

Taxon classificationAnimaliaSorbeoconchaMelanopsidae

Brusina, 1902

##### Original source.

Brusina 1902: pl. 6, fig. 1.

##### Type horizon.

Early–middle Miocene.

##### Type locality.

“Kirin (Stipan)”, Croatia.

##### Types.

Milan et al. (1974: 98) indicated a holotype, but it is uncertain whether the specimen was the only one Brusina had at hand (holotype by monotypy, Art. 73.1.2). The specimen is stored in the Croatian Natural History Museum, Zagreb, coll. no. 2502-148.

#### Microcolpia villeserriana

Taxon classificationAnimaliaSorbeoconchaMelanopsidae

Bourguignat, 1884

##### Original source.

Bourguignat 1884: 58.

##### Type locality.

“Dans les cours d’eau aux environs d’Ismidt, en Anatolie” [in rivers around İzmit], Turkey.

#### Melanopsis liocephala var. villica

Taxon classificationAnimaliaSorbeoconchaMelanopsidae

Pallary, 1936

##### Original source.

Pallary 1936: 57.

##### Type locality.

Not indicated, but probably in Morocco.

#### † Melanopsis vincta

Taxon classificationAnimaliaSorbeoconchaMelanopsidae

Blanckenhorn, 1897

##### Original source.

Blanckenhorn 1897: 115, pl. 9, figs 6–7.

##### Type horizon.

Plio-Pleistocene.

##### Type locality.

“In der ersten Thonbank des linken Orontesufers bei Dschisr esch-Schurr” [in the first clay bank at the left riverside of the Orontes near Jisr Ash-Shughur], Syria.

##### Remarks.

Introduced as “n. mut.” but clearly as a binomen and hence not infrasubspecific in the sense of ICZN Art. 45.6.

#### † Melanopsis vindobonensis

Taxon classificationAnimaliaSorbeoconchaMelanopsidae

Fuchs in Fuchs & Karrer, 1870

##### Original source.

Fuchs and Karrer 1870: 140, textfig. 5.

##### Type horizon.

Pannonian, zone E, late Miocene.

##### Type locality.

“Zu Brunn, Inzersdorf, Rothneusiedel und Wien” [in Brunn am Gebirge, Inzersdorf and Rothneusiedl (today both localities lie within Vienna), and Vienna], Austria.

#### † Melanopsis viquesneli

Taxon classificationAnimaliaSorbeoconchaMelanopsidae

Pavlović, 1932

##### Original source.

Pavlović 1932: 240, 247, pl. 1, figs 6–7.

##### Type horizon.

Pontian (Dacian Basin), late Miocene–Pliocene.

##### Type locality.

“Села Дрсника” [village Drsnik], Kosovo.

##### Types.

Perhaps stored in the Natural History Museum, Belgrade, but not mentioned in the catalogue of Milošević (1962).

#### † Melanopsis visianiana

Taxon classificationAnimaliaSorbeoconchaMelanopsidae

Brusina, 1874

##### Original source.

Brusina 1874: 37, pl. 1, figs 7–8.

##### Type horizon.

Langhian, middle Miocene.

##### Type locality.

“Miočić”, Croatia.

##### Types.

Milan et al. (1974: 99) indicated a holotype, but it is uncertain whether the specimen was the only one Brusina had at hand (holotype by monotypy, Art. 73.1.2). The specimen is stored in the Croatian Natural History Museum, Zagreb, coll. no. 3205-851.

#### † Melanopsis vitalisi

Taxon classificationAnimaliaSorbeoconchaMelanopsidae

Strausz, 1942

##### Original source.

Strausz 1942: 92, pl. 5, figs 14, 20, 29.

##### Type horizon.

Transdanubian, Pannonian, late Miocene.

##### Type locality.

The locality was indicated as number “85” by Strausz (1942: 63, 101), which refers to the *Canthidomus balatonica* horizon at Nyárád, Hungary (p. 23).

#### † Melanopsis vitezovici

Taxon classificationAnimaliaSorbeoconchaMelanopsidae

Brusina, 1902

##### Original source.

Brusina 1902: pl. 5, figs 61–64.

##### Type horizon.

Langhian, middle Miocene.

##### Type locality.

“Džepe” [Džepi], Bosnia and Herzegovina.

##### Types.

Neubauer et al. (2016c: 280) designated a lectotype, which is stored in the Croatian Natural History Museum, Zagreb, coll. no. 2501-147/1-4.

#### † Melanopsis vittata

Taxon classificationAnimaliaSorbeoconchaMelanopsidae

Pallary, 1916

##### Original source.

Pallary 1916: 86.

##### Type horizon.

Pannonian, zone D, late Miocene.

##### Type locality.

“Kottingbrunn [...] Ziegelei a” (Handmann 1882: 560), Austria.

##### Remarks.

Replacement name for *Melanopsis fasciata* Handmann, 1882, non Gassies, 1874. Wenz (1929: 2741) considered the taxon as a junior synonym of *Melanopsis haueri* Handmann, 1882.

#### † Melanopsis vitzoui

Taxon classificationAnimaliaSorbeoconchaMelanopsidae

Porumbaru, 1881

##### Original source.

Porumbaru 1881: 27, pl. 8, fig. 4.

##### Type horizon.

Early Romanian, Pliocene.

##### Type locality.

“Bucovatzu” [Bucovăț], Romania.

##### Remarks.

The name “*vitzui*” as mentioned in Pallary (1926a: 93) is an incorrect subsequent spelling.

#### Melanopsis vondeli

Taxon classificationAnimaliaSorbeoconchaMelanopsidae

Pallary, 1928

##### Original source.

Pallary 1928b: 16, pl. 2, figs 9–14.

##### Type locality.

“Beni Mellal, dans l’oued Taguenout” [Beni Mellal, in the Oued Taguenout (?)], Morocco.

#### † Melanopsis vrcinensis

Taxon classificationAnimaliaSorbeoconchaMelanopsidae

Neubauer, Harzhauser, Georgopoulou, Mandic & Kroh, 2014

##### Original source.

Neubauer et al. 2014a: 457.

##### Type horizon.

Middle Pannonian, late Miocene.

##### Type locality.

“У Карагачу” (Pavlović 1927: 60) [from Karagača near Vrčin], Serbia.

##### Types.

Milošević (1962: 23) indicated that the syntype illustrated by Pavlović is stored in the Natural History Museum, Belgrade, coll. no. 209.

##### Remarks.

Replacement name for *Melanopsis glabra* Pavlović, 1927, non Brusina, 1874 (see Note 1).

#### Melanopsis wagneri

Taxon classificationAnimaliaSorbeoconchaMelanopsidae

Roth, 1839

##### Original source.

Roth 1839: 24, pl. 2, fig. 11.

##### Type locality.

“Smyrnae” [Izmir], Turkey.

##### Remarks.

Martens (1874: 32) considered the taxon as a junior synonym of *Melanopsis praemorsa* (Linnaeus, 1758).

#### Melanopsis waitaraensis

Taxon classificationAnimaliaSorbeoconchaMelanopsidae

Marwick, 1926

##### Original source.

Marwick 1926: 317, pl. 73, figs 1–2.

##### Type locality.

“Coast south side Wai-iti Stream”, New Zealand.

#### † Melanopsis wilhelmi

Taxon classificationAnimaliaSorbeoconchaMelanopsidae

Esu & Girotti, 2015

##### Original source.

Esu and Girotti 2015b: 150, figs 1–2.

##### Type horizon.

Late early Pleistocene.

##### Type locality.

“Stirone river section, between Laurano and S. Nicomede (Emilia)”, Italy.

##### Types.

Senckenberg Forschungsinstitut und Naturmuseum Frankfurt, coll. no. SMF 345834.

#### † Melanopsis wolfgangfischeri

Taxon classificationAnimaliaSorbeoconchaMelanopsidae

Neubauer, Harzhauser, Kroh, Georgopoulou & Mandic, 2014

##### Original source.

Neubauer et al. 2014c: 17.

##### Type horizon.

Pannonian, zone B–D, late Miocene.

##### Type locality.

“Leobersdorf” (Handmann 1887), Austria.

##### Types.

See statement for *Melanopsis martiniana rugosa* Handmann, 1887.

##### Remarks.

Replacement name for *Melanopsis martiniana rugosa* Handmann, 1887, non Matheron, 1842 (see Note 1).

#### † Melanopsis wuenschmanni

Taxon classificationAnimaliaSorbeoconchaMelanopsidae

Mertin, 1939

##### Original source.

Mertin 193: 206, pl. 6, fig. 3.

##### Type horizon.

Heidelberg Formation, late Santonian, late Cretaceous.

##### Type locality.

“Flugplatz Quedlinburg” [airfield at Quedlinburg], Germany.

#### † Fagotia wuesti

Taxon classificationAnimaliaSorbeoconchaMelanopsidae

Meijer, 1990

##### Original source.

Meijer 1990: 152, pl. 2, figs 1–2.

##### Type horizon.

Bavelian Complex, Pleistocene.

##### Type locality.

“Clay-pit North of the village of Bavel (province of Noord Brabant, The Netherlands)”, Netherlands.

##### Types.

Rijks Geologische Dienst, Haarlem, The Netherlands; no number indicated.

##### Remarks.

Glöer (2002: 79) assigned the species to Esperiana (Microcolpia).

#### Melanopsis zarudnyi

Taxon classificationAnimaliaSorbeoconchaMelanopsidae

Izzatullaev & Starobogatov, 1984

##### Original source.

Izzatullaev and Starobogatov 1984: 1479, fig. 1 (12), fig. 3 (6).

##### Type locality.

“Золотой ключ около Ашхабада” [Zolotoy spring near Ashgabat], Turkmenistan.

##### Types.

Zoological Institute of Russian Academy of Sciences, St.-Petersburg; no number indicated.

#### † ? Melanopsis zea

Taxon classificationAnimaliaSorbeoconchaMelanopsidae

Pezant, 1908

##### Original source.

Pezant 1908: 200, pl. 7, figs 23–24.

##### Type horizon.

Bartonian, Eocene.

##### Type locality.

“Monneville”, France.

##### Remarks.

Probably not a Melanopsidae.

#### Melanopsis zebra

Taxon classificationAnimaliaSorbeoconchaMelanopsidae

De Cristofori & Jan, 1832

##### Original source.

De Cristofori and Jan 1832: 4.

##### Type locality.

“Guinea”, indicated in the previous part of the same work (“Conchylia terrestria et fluviatilia [...]”, p. 7; there, the name is a nomen nudum).

##### Remarks.

Probably not a Melanopsidae.

#### Melanopsis fasensis var. zebrina

Taxon classificationAnimaliaSorbeoconchaMelanopsidae

Pallary, 1920

##### Original source.

Pallary 1920c: 148.

##### Type locality.

“Sefrou”, Morocco.

#### Melanopsis zelandica

Taxon classificationAnimaliaSorbeoconchaMelanopsidae

Gould, 1847

##### Original source.

Gould 1847: 225.

##### Type locality.

“New Zealand” [no locality indicated].

##### Remarks.

Suter (1893: 139) considered the taxon as a junior synonym of *Zemelanopsis trifasciata* (Gray in Dieffenbach, 1843). The name “*zealandica*” as mentioned in Hutton (1883: 67) is an incorrect subsequent spelling.

#### † Melanopsis (Canthidomus) zitteli

Taxon classificationAnimaliaSorbeoconchaMelanopsidae

Neumayr, 1869

##### Original source.

Neumayr 1869: 357, pl. 11, figs 4–5.

##### Type horizon.

Langhian, middle Miocene.

##### Type locality.

“Miocic” [Miočić], Croatia.

##### Types.

Illustrated syntypes are stored at the Geological Survey Austria, Vienna, coll. no. 1869/01/5/1-2.

#### Melanopsis maroccana var. zonata

Taxon classificationAnimaliaSorbeoconchaMelanopsidae

Gassies, 1856

##### Original source.

Gassies 1856: 12, figs 5–6.

##### Type locality.

“L’Aïn-Kadra, sur les hauts plateaux de l’Atlas, à deux mètres des Chots” [in the ‘Aïn Khadr, in the highlands of the Atlas, two meters in the Chott (probably he meant the lake Chott el Hodna)], Algeria.

#### Melanopsis praemorsa var. zonata

Taxon classificationAnimaliaSorbeoconchaMelanopsidae

Bourguignat, 1864 [invalid]

##### Original source.

Bourguignat 1864: 264.

##### Type locality.

“Biskra”, Algeria.

##### Remarks.

Junior homonym of *Melanopsis maroccana zonata* Gassies, 1856 (see Note 1), which Bourguignat listed as well (p. 260–261).

#### Melanella afra var. zonata

Taxon classificationAnimaliaSorbeoconchaMelanopsidae

Bourguignat, 1884

##### Original source.

Bourguignat 1884: 22.

##### Type locality.

“Aus einem Mühlbache bei Nassenfuss in Unterkrain” [from a mill creek near Mokronog], Slovenia.

##### Remarks.

Introduced for a specimen of Melania holandri var. laevigata figured in Rossmässler (1839: 38, pl. 50, fig. 666).

#### Melanopsis maroccana var. zonatosubcostata

Taxon classificationAnimaliaSorbeoconchaMelanopsidae

Paladilhe, 1875

##### Original source.

Paladilhe 1875: 94.

##### Type locality.

“Meknès”, Morocco.

##### Remarks.

Described originally as “*zonato-subcostata*”.

#### Melanopsis zonites

Taxon classificationAnimaliaSorbeoconchaMelanopsidae

Gassies, 1870

##### Original source.

Gassies 1870: 147.

##### Type locality.

“Prope Saint-Vincent” [near Saint-Vincent], New Caledonia.

##### Remarks.

Brot (1879: 457) considered the species as a junior synonym of *Melanopsis brevis* Morelet, 1857 (which is a junior homonym of *Melanopsis brevis* Sowerby, 1826; see *Melanopsis moreleti* Pallary, 1916).

#### Melanopsis praerosa [sic] var. zonulata

Taxon classificationAnimaliaSorbeoconchaMelanopsidae

Paetel, 1888

##### Original source.

Paetel 1888: 403.

##### Type locality.

“Persia” (Nevill 1884: 208), Iran.

##### Remarks.

Originally introduced as infrasubspecific taxon (“subvariety”) by Nevill (1884), but made available by Paetel (1888) who treated it as variety (Art. 45.5.1). Paetel clearly referred to the description of Nevill.

#### † Melanopsis zujovici

Taxon classificationAnimaliaSorbeoconchaMelanopsidae

Brusina, 1893

##### Original source.

Brusina 1893: 200 (Serbian part), 33 (Italian part), pl. 2, fig. 5.

##### Type horizon.

Pannonian, zone D–E, late Miocene.

##### Type locality.

“Ripanj”, Serbia.

##### Types.

Milan et al. (1974: 99) indicated a holotype, but it is uncertain whether the specimen was the only one Brusina had at hand (holotype by monotypy, Art. 73.1.2). The specimen is stored in the Croatian Natural History Museum, Zagreb, coll. no. 2531-177/1.
